# A bearing fault diagnosis method based on hybrid artificial intelligence models

**DOI:** 10.1371/journal.pone.0327646

**Published:** 2025-07-31

**Authors:** Lijie Sun, Xin Tao, Yanping Lu

**Affiliations:** 1 School of Art and Design, Taizhou University, Taizhou, Zhejiang, China; 2 School of Electronics and Information Engineering, Taizhou University, Taizhou, Zhejiang, China; 3 School of Information, Liaoning University, Shenyang, Liaoning, China; VIT-AP Campus, INDIA

## Abstract

The working state of rolling bearing severely affects the performance of industrial equipment. Addressing the issue of that the difficulty of incipient weak signals feature extraction influences the rolling bearing diagnosis accuracy, an efficient bearing fault diagnostic technique, a proposition is forwarded for hybrid artificial intelligence models, which integrates Improved Harris Hawks Optimization (IHHO) into the optimization of Deep Belief Networks and Extreme Learning Machines (DBN-ELM). The process employs Maximum Second-order Cyclostationary Blind Deconvolution (CYCBD) to filter out noise from the vibration signals emitted by bearings; secondly, considering the issue with the conventional Harris Hawks Optimization (HHO) algorithm which tends to prematurely converge to local optima, the differential evolution mutation operator is introduced and the escape energy factor is improved from linear to nonlinear in IHHO; then, a double-layer network model based on DBN-ELM is proposed, to avoid the number of hidden layer nodes of DBN from human experience interference, and IHHO is used to optimize DBN structure, which is denoted as IHHO-DBN-ELM method; with the optimal structure is obtained by using a combined IHHO optimized DBN and ELM; in conclusion, the proposed IHHO-DBN-ELM approach is applied to the bearing fault detection using the Western Reserve University’s bearing fault dataset. The outcome of the experiments demonstrates that IHHO-DBN-ELM technique successfully extracts fault characteristics from the raw time-domain signals, thereby offering enhanced diagnostic accuracy and superior generalization capabilities.

## Introduction

With the continuous advancement of modern industrial production systems, ensuring the safety and reliability of mechanical equipment has become increasingly critical. When a machine or component fails, it disrupts production processes, leading to significant financial losses. Moreover, severe malfunctions may endanger human lives and result in irreversible consequences. As key components of industrial rotating machinery, rolling bearings often operate under extreme conditions, such as high temperatures and heavy loads, making them particularly prone to failure. Therefore, timely and accurate detection of bearing faults is essential for maintaining efficient and safe industrial operations [1].

The early symptoms of rolling bearing faults typically appear as vibrations exhibiting nonlinear and non-stationary characteristics. These fault-induced signals are often embedded in background noise, making it challenging to extract meaningful diagnostic features. To address this issue, many researchers employ blind deconvolution algorithms to enhance signal quality by suppressing noise and recovering underlying fault signatures. A fault diagnostic approach was introduced by Zhang et al. [2], which integrated Continuous Vibration Separation (CVS) methodology with the application of Minimum Entropy Deconvolution (MED). The MCKD (Maximum Correlated Kurtosis Deconvolution) algorithm was introduced by MCDONALD et al. [3], with its primary focus on utilizing correlation kurtosis for optimization, thus emphasizing concealed fault characteristics amidst noise. In a subsequent study, Cui et al. [4] merged Variational Mode Decomposition (VMD) with MCKD to enhance fault detection. This approach strengthened the faint pulse signals of rolling element faults through the application of MCKD. However, MCKD has strict requirements on several input parameters such as filter length, maximum number of iterations and shift order, and is less effective in extracting shock components. These achievements have achieved certain results in signal denoising, but there are also fatal drawbacks. The CYCBD algorithm, which stands for Maximum Second-order Cyclostationarity Blind Deconvolution, was introduced by BUZZONI and collaborators in [5]. This method relies on the principle of maximizing the second-order cyclostationary statistic. Compared with other deconvolution algorithms, the proposed method demonstrates superior performance in extracting impulses under noisy conditions, effectively enhancing fault-related features in the signal. Given these advantages, this study employs the CYCBD to refine raw bearing vibration signals and suppress noise interference.

After ideal noise reduction effect is obtained for the bearing vibration signal, many scholars further extract the features and input them into the intelligent diagnosis model, which includes capsule network [6], ANN [7], BPNN [8]. However, these shallow models have poor fitting ability during dealing with complex nonlinear data, which resulting in low diagnostic accuracy [9–12].

In recent years, advanced deep learning architectures, including Convolutional Neural Networks (CNNs), Deep Belief Networks (DBNs), Autoencoders (AEs), and Recurrent Neural Networks (RNNs), have demonstrated remarkable success in bearing fault detection applications [13–17]. Among these, DBNs exhibit particular strengths in learning complex nonlinear relationships from input data through their unique unsupervised layer-wise training mechanism. This architecture endows DBNs with exceptional nonlinear mapping capabilities and robust adaptability. As a result, DBNs have proven particularly effective at extracting subtle fault features from mechanical vibration signals, enabling highly accurate identification of incipient bearing defects. A hybrid Multi model fusion method for fault diagnosis, incorporating deep learning, which utilized different DBN combinations to achieve decision-level multimodal fusion, and was suggested by Che et al. [18]. An inventive Optimized Adaptive Deep Belief Network (OABN) was put forward by Gao et al. [19]. OABN undergoes initial training through minimal batch stochastic gradient descent. Subsequently, it is refined with the combination of backpropagation (BP) and conjugate gradient descent, thereby exhibiting superior classification capabilities. In reference [20], Yan and collaborators introduced a technique for unbalanced fault diagnosis utilizing Deep Belief Network (DBN). The results showed that a cracking classification accuracy was obtained in unbalanced data. Simultaneously, Extreme Learning Machine (ELM) possesses the distinct virtue of neural networks approximating nonlinear functions compared to SVM. Moreover, ELM has a fast learning rate and powerful generalization ability. It is currently widely utilized in fault classification fields and achieved ideal results [21–23]. Therefore, combining the advantages of both DBN and ELM algorithms, so lots of scholars have established the DBN-ELM model, Gao et al. [24] utilized the DBN-ELM for power quality disturbance (PQD) classification. DBN extracted features from the denoised PQD signal, which improved the PQD classification efficiency through the ELM classifier. In the work by Cui et al. [25], a technique was introduced for the fault detection in gas turbines utilizing DBN-ELM architecture. The method was verified by experiments that DBN-ELM was more accurate than using DBN or ELM respectively. The DBN-ELM exhibits exceptional capabilities in data feature extraction and model classification efficiency, which led to the development of a fault diagnostic model that leverages these strengths.

The aforementioned approaches offer valuable insights for bearing feature extraction using Deep Belief Networks (DBNs). However, the performance of DBNs is critically dependent on the number of neurons in their hidden layers. Achieving optimal trade-offs between model stability and predictive accuracy, without resorting to empirical guesswork, remains a significant challenge in practical applications. Currently, swarm intelligence optimization algorithms have been extensively studied by many scholars [26–31], which is adopted in the selection of the optimal parameters of the models. Referring to Zhang et al.‘s work [32], they introduced an innovative approach for diagnosing gearbox bearing faults. This approach fuses a Single-Dimensional Convolutional Neural Network (1DCNN) alongside a Support Vector Machine (SVM) for an integrated solution, where the SVM component is meticulously fine-tuned using Particle Swarm Optimization (PSO). Then, the experiments showed that the proposed 1DCNN-PSO-SVM improved the diagnostic accuracy. In the work of Ke et al. [33], they introduced a strategy that harnesses Genetic Algorithm (GA) to fine-tune the hyperparameters of Deep Convolutional Neural Networks (DCNNs), demonstrating exceptional efficiency in the context of modular multilevel converters (MMCs). Separately, in reference [34], Jin and colleagues employed Deep Belief Network (DBN) refined by an enhanced Grey Wolf Optimization (GWO) algorithm for the purpose of identifying faint faults in bearings. Their study confirmed the effective diagnosis of subtle faults in axle box bearings.

In light of the preceding research, this study introduces a technique forbearing fault diagnosis, which harnesses the improved Harris Hawks Optimizer (IHHO) to refine Deep Belief Networks (DBN). Firstly, CYCBD method is applied to denoise the raw bearing vibration signals, followed by time-domain feature dimension reduction using Principal Component Analysis (PCA). Secondly, Harris Hawks Optimizer is improved by incorporating both a differential evolution mutation strategy and a nonlinear escape energy mechanism, namely IHHO. This dual modification significantly improves the algorithm’s global exploration capability while effectively preventing premature convergence to local optima. The optimized IHHO algorithm then determines the optimal number of hidden units in the DBN-ELM framework. Finally, the selected time-domain features are fed into the optimally configured DBN-ELM model for fault classification. Experimental results demonstrate that our hybrid AI model achieves superior diagnostic accuracy with excellent generalization capability, though requiring longer training times due to its numerous parameters.

## Related works

### Harris hawks optimizer

Harris Hawk Optimizer (HHO), introduced by Heidari et al. [35] in 2019, is an innovative optimization strategy that mimics the hunting dynamics of hawks pursuing their prey. HHO follows three indispensable stages: exploration, the shift from exploration to exploitation, and finally, exploitation.

**Exploration:** In the exploration stage, Harris hawks foraging includes two mechanisms, randomly perching in tall trees or hunting according to other members and the location of the prey. A mathematical formulation is constructed as depicted in equation (1).


$$X_{i}\left(t+1\right)=\left\{\begin{array}{c} X_{rand}(t)-r_{1}\left|X_{rand}(t)-2r_{2}X_{i}(t)\right|\;\;\;\;\;\;,q\geq 0.5\{X_{rabbit}\}(t)-X_{m}(t))-r_{3}(lb+r_{4}(ub-lb);,q<0.5 \end{array}\right\}$$ (1)

Here, $X_{i}\left(t+1\right)$ and $X_{i}\left(t\right)$ denote the location of an individual at the present and subsequent iterations, correspondingly. $X_{m}\left(t\right)$ signifies the average strategic location of every participant, $X_{rabbit}\left(t\right)$ symbolizes the prey’s position, $X_{rand}\left(t\right)$ represents a selectively chosen individual’s position, t denotes the total number of iterations, and $r_{1},r_{2},r_{3},r_{4},q$ are random values that lie within the range of 0 and 1.

**The shift from exploration to exploitation:** The shift of Harris’ hawks from discovery to utilization primarily revolves around the prey’s escape energy, as depicted in equation (2).


$$E=2E_{0}(1-\frac{t}{T})$$ (2)

Here, $E_{0}$ represents the initial escape energy of the prey, t is the present iteration count, and T is upper limit for iterations.

**Exploitation:** Harris hawks exhibit a range of four strategic hunting techniques: soft besiege, hard besiege, soft besiege with progressive rapid dives and hard besiege with progressive rapid dives.

(1) Soft besiege: In the scenario where $\begin{array}{c} \text{r}>0.5,|\text{E}|<0.5 \end{array}$, the prey, despite possessing adequate energy to evade, succumbs to the pursuit. Harris hawk skillfully encircles its target before executing an unexpected assault. The position is updated as follows:

$$X_{i}(t+1)=X_{rabbit}(t)-X_{i}(t)-E|JX_{rabbit}(t)-X_{i}(t)|$$ (3)

$$J=2(1-r_{5})$$ (4)

where, ${\text{r}}_{\text{5}}\in [0,1]$, and J is the jumping distance during the escape of the rabbit.

(2) Hard besiege: In the scenario where $\begin{array}{c} \text{r}\geq 0.5,|\text{E}|<0.5 \end{array}$, the prey is depleted of energy and cannot escape. So, in HHO, the prey is exhausted and can attack it with little need to surround the prey. The update of position is shown in (5).

$$X_{i}(t+1)=X_{rabbit}(t)-E|X_{rabbit}(t)-X_{i}(t)|$$ (5)

(3) Soft besiege with progressive rapid dives: In the scenario where $\begin{array}{c} \text{r}<0.5,|\text{E}|\geq 0.5 \end{array}$, it is possible for prey to escape because of sufficient energy. Harris hawks dives quickly around the prey several times, and always adjust its position to attack it. The formula is as follows:

$$X_{i}(t+1)=\{\begin{array}{cc} {}*20cY, & if\;F(Y)<F(X_{i}(t)),\\ Z, & if\;F(Z)<F(X_{i}(t)) \end{array}$$ (6)

$$Y=X_{rabbit}(t)-E|JX_{rabbit}(t)-X_{i}(t)|$$ (7)

Z=Y+S×LF(D) (8)

Here, D represents the dimension, S is the random vector by D×1 inside (0,1), while LF denotes the Levy flight function.

(4) Hard besiege with progressive rapid dives: In the scenario where $\begin{array}{c} \text{r}<0.5,|\text{E}|<0.5 \end{array}$, the prey has insufficient energy. A hard encirclement to raid the prey is formed in HHO, and the position is updated as (9).

$$X_{i}(t+1)=\{\begin{array}{cc} {}*20cX_{rabbit}(t)-E\left|JX_{rabbit}(t)-X_{i}(t)\right|, & if\;F(Y)<F(X_{i}(t)),\\ Y+S\times LF(D), & if\;F(Z)<F(X_{i}(t)) \end{array}$$ (9)

### Deep belief network (DBN)

**Fig 1:**
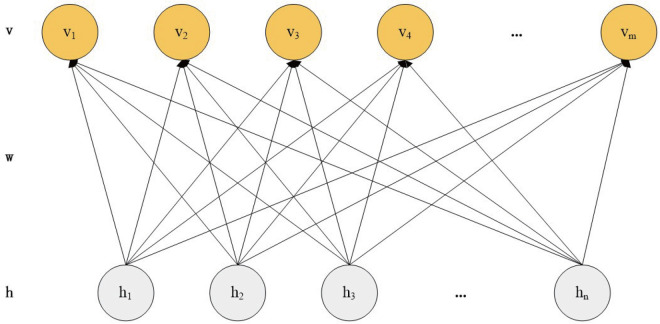
The structure of RBM.

DBN is constructed with multiple strata of Restricted Boltzmann Machines (RBMs). Within a RBM architecture, you’ll find a visible unit denoted as ‘v’ and a concealed unit referred to as ‘h’. The role of the visible unit v is to process incoming data, whereas the hidden unit h undertakes the task of extracting high-level features. The architectural layout of an RBM is depicted in Fig 1.

The connection weights and biases between RBM layers are initialized in an unsupervised greedy way. Then, each layer of RBM is trained from bottom to top. The energy defined between layers is as (10).


$$E(v,h|\theta )=-\sum_{i=1}^{n}a_{i}v_{i}-\sum_{j=1}^{m}b_{j}h_{j}-\sum_{i=1}^{n}\sum_{j=1}^{m}v_{i}W_{ij}h_{j}$$ (10)

Here, $\theta =\{W_{ij},a_{i},b_{j}\}$ denotes the parameter of the RBM, with a and b denoting the biases for the visible and hidden layers individually, while W represents the weight matrix. The joint probability distribution function can be calculated by (10) as shown in (11).

The connection weights and biases between RBM layers are initialized in an unsupervised greedy way. Then, each layer of RBM is trained from bottom to top. The energy defined between layers is as (11).


$$p(v,h|\theta )=\frac{e^{-E(v,h|\theta )}}{\sum_{v}\sum_{h}e^{-E(v,h|\theta )}}$$ (11)

The probabilities governing the activation of each visible layer element and hidden layer unit are depicted in equations (12) and (13) respectively.


$$p(v_{i}=1|h)=\sigma (a_{i}+\sum_{j}w_{ij}h_{j})$$ (12)

$$p(h_{j}=1|v)=\sigma (b_{j}+\sum_{i}w_{ij}v_{i})$$ (13)

Here, σ(x) is the Logistic function.

The parameters of RBM are refined through optimizing the maximum likelihood estimation equation derived from the training dataset, thus proceeding as equations (14)-(16).


$$\Delta W_{ij}=\varepsilon (<v_{i}h_{j}{>}_{data}-<v_{i}h_{j}{>}_{recon})$$ (14)

$$\Delta a_{i}=\varepsilon (<v_{i}{>}_{data}-<v_{i}{>}_{recon})$$ (15)

$$\Delta b_{j}=\varepsilon (<h_{j}{>}_{data}-<h_{j}{>}_{recon})$$ (16)

In this context, ε denotes the Learning rate, ${<\cdot >}_{data}$ signifies the anticipated values for the training dataset, while ${<\cdot >}_{recon}$ stands for the mathematical expectation of the reconstructed model.

### Extreme learning machine

Extreme Learning Machine (ELM) represents an efficient machine learning algorithm specifically designed for Single Hidden Layer Feedforward Neural Networks (SLFNs). Unlike conventional approaches, ELM employs a unique batch training methodology characterized by randomly initialized hidden layer weights and biases.ELM’s training phase concludes through the determination of the hidden layer’s output weights. As mathematically formulated in Equation (17), it expresses the network output.


$$\sum_{i=1}^{N}g(w_{i}\cdot x_{j}+b_{i})\beta_{i}=y_{j},j=1,2,\cdots ,N$$ (17)

Here, g(x) is the activation function of the hidden layers, $w_{i}$ and $\beta_{i}$ are the weights for the input and output, respectively. $b_{i}$ is the bias of the hidden layer i, and $y_{j}$ represents the target vector,j is the sequence number of the target vector. The ELM training process can be simplified as shown in (18).


Hβ=Y (18)

Where, H denotes the output from the nodes in hidden layers, and Y represents the desired output. β can be obtained from (19).


$$\beta =H^{+}Y$$ (19)

Here, $H^{+}$ represents the generalized inverse of H.

## The proposed method

### IHHO

During iterative optimization, the conventional Harris Hawks Optimizer (HHO) exhibits progressive loss of population diversity, significantly impairing its capacity to avoid local optima convergence. To mitigate this limitation, a mutation mechanism is integrated during the exploitation phase, adopting the well-known DE/best/2 mutation operator from [36] to supplant the initial position update approach outlined in the equation. The enhanced position update formulation is depicted in (20).


$$X_{i}\left(t+1\right)=X_{best}\left(t\right)+F\left(X_{r1}\left(t\right)-X_{r2}\left(t\right))+F(X_{r3}\left(t\right)-X_{r4}\left(t\right)\right)$$ (20)

$$F=F_{0}\times 2^{\lambda }$$ (21)

$$\lambda =e^{1-\frac{T}{T+1-t}}$$ (22)

Here, $r_{1}$, $r_{2}$,$r_{3}$ and $r_{4}$ are distinct integers chosen from [1,N]. In order to avoid the phenomenon of premature maturity, the factorF with adaptive variation is increased, and the initial value of F is set to 0.5. Therefore, the position update in the exploration phase is changed from (1) to (23):


$$X_{i}\left(t+1\right)=\left\{\begin{array}{c} X_{best}\left(t\right)+F\left(X_{r1}\left(t\right)-X_{r2}\left(t\right))+F(X_{r3}\left(t\right)-X_{r4}\left(t\right)\right)\;\;\;\;\;q\geq 0.5\\ (X_{rabbit}-X_{m}\left(t\right))-rand1\times (lb+rand2\times (ub-lb))\;\;\;\;\;\;\;\;\;\;\;\;\;\;\;\;\;\;\;\;\;\;\;\;\;\;\;\;\;\;\;\;\;\;\;\;\;\;\;\;\;\;\;\;\;\;\;\;\;\;\;\;\;\;\;\;\;\;q<0.5 \end{array}\right\}$$ (23)

The escape energy factor E of the prey plays a crucial role in balancing the global search and local development phases.

Upon the condition that |E|≥1, the algorithm initiates the exploration phase at the outset of the iterative process, hawks increase the global search range. When |E|≤1, hawks narrow the search for local development during the development phase.

During the exploration phase of |E|≥1, the algorithm expands the comprehensive search territory, whereas in the development phase of |E|<1, the hawks refine their focus for localized progress. With the progressive rise in iterations, the value E in (3) decreases linearly with the change of 1−(\raise0.7ext/tT\nulldelimiterspace\lower0.7exT) in the conventional HHO algorithm. It is noteworthy that the algorithm typically exhibits robust global search capabilities during the initial phases of its iterative process, while the later iterations focus more on the local search capability. Therefore, the transition parameter E is improved as shown in (24).


E=2E0a(t) (24)

$$a(t)=1-(\frac{e^{\frac{t}{T}}-1}{e-1}{)}^{2}$$ (25)

**Fig 2:**
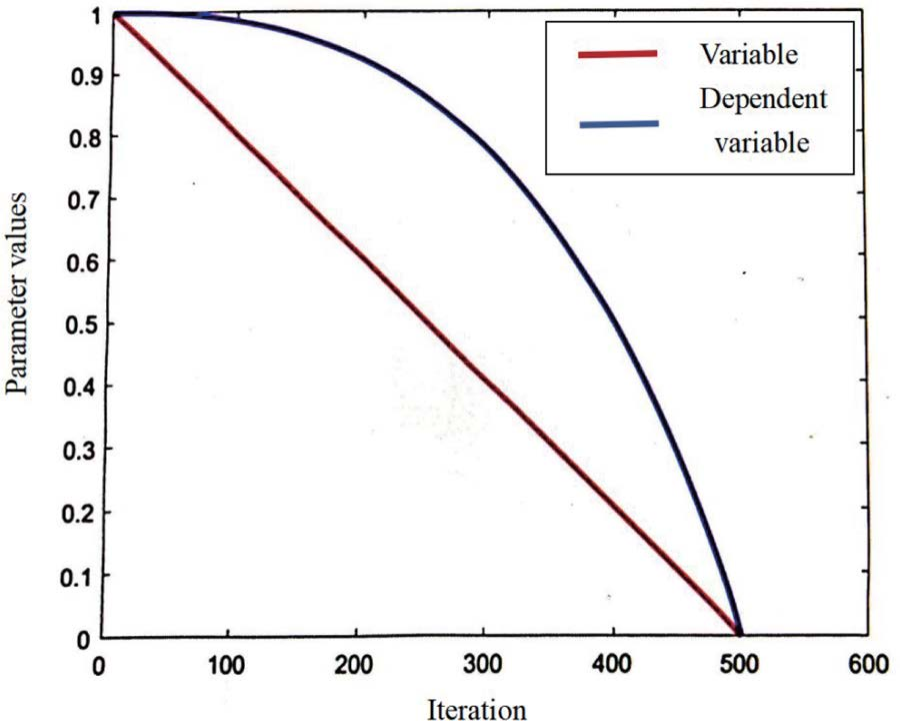
The change curves of 1-(t/T) and a(t).

As the number of iterations progresses, the convergence factor a(t) diminishes non-linearly from 1 to 0. This evolution of 1−(\raise0.7ext/tT\nulldelimiterspace\lower0.7exT) and a(t) is graphically depicted in Fig 2. Diverging from the linear trajectory of 1−(\raise0.7ext/tT\nulldelimiterspace\lower0.7exT), the non-linear transformation of a(t) facilitates a broader exploratory scope during the initial iteration stages, thereby augmenting the algorithm’s global search capabilities to prevent premature localization into a suboptimal solution. As the iterations progress, the parameter value declines at an accelerated pace, facilitating precise local searches and enhancing the algorithm’s convergence velocity. Consequently, the enhanced non-linear escape energy facilitates a more stable transition between the early exploration phase and the subsequent exploitation phase, thereby being more beneficial for enhancing both the convergence velocity and solution precision of HHO.

### Performance analysis of IHHO algorithm

To rigorously evaluate the performance enhancement of our proposed Improved Harris Hawks Optimizer (IHHO), comparative experiments are conducted using Harris Hawk Optimizer (HHO), Whale Optimization Algorithm (WOA) [37,38], Grey Wolf Optimizer (GWO) and Genetic Algorithm (GA). These independent replication trials are executed on a set of six benchmark functions detailed in Table 1, sourced from the CEC (International Conference on Evolutionary Computing) Test Suite. These functions are relatively straightforward and facilitate convenient implementation. In addition, $f_{1}$ ~$f_{3}$ are single-maxima benchmark functions, and $f_{4}$ ~$f_{6}$ are versus complex multi-maxima counterparts. A single-maximum function serves to gauge the localized search capability of a swarm algorithm, whereas a multi-maxima function challenges its global exploration capacity.

**Table 1:** Description of test functions.

Function	Specific formula	Range
$f_{1}$	$f_{1}(x)=\sum_{i=1}^{n-1}\left[100{\left(x_{i+1}-x_{i}^{2}\right)}^{2}+{\left(x_{i}-1\right)}^{2}\right]$	$[-30,30{]}^{n}$
$f_{2}$	$f_{2}(x)=\sum_{i=1}^{n}{\left([x_{i}+0.5]\right)}^{2}$	$[-100,100{]}^{n}$
$f_{3}$	$f_{3}(x)=\sum_{i=1}^{n}ix\begin{array}{c} {}*20c4\\ i \end{array}+random[0,1)$	$[-1.28,1.28{]}^{n}$
$f_{4}$	$f_{4}(x)=\sum_{i=1}^{n}-x_{i}\sin(\sqrt{\left|x_{i}\right|})$	$[-500,500{]}^{n}$
$f_{5}$	$\begin{array}{c} f_{5}(x)=\frac{\pi }{n}\left\{\begin{array}{c} 10\sin(\pi y_{1})+\\ \sum_{i=1}^{n-1}{\left(y_{i}-1\right)}^{2}[1+10\sin^{2}(\pi y_{i+1})]+{\left(y_{n}-1\right)}^{2} \end{array}\right\}\\ +\sum_{i=1}^{n}u(x_{i},10,100,4) \end{array}$	$[-50,50{]}^{n}$
$f_{6}$	$\begin{array}{c} f_{6}(x)=0.1\{\sin^{2}(3\pi x_{1})+\sum_{i=1}^{n}{\left(x_{i}-1\right)}^{2}[1+\sin^{2}(3\pi x_{i}+1)]\\ +(x_{n}-1{)}^{2}[1+\sin^{2}(2\pi x_{n})]\}+\sum_{i=1}^{n}u(x_{i},5,100,4) \end{array}$	$[-50,50{]}^{n}$

The experimental configuration entails setting particular parameters such as a population size of 30 for each algorithm and a maximum iteration limit of 300. The logarithmic spiral parameter b in WOA is fixed at 1, while the search factor is configured to diminish linearly from a value of 2 down to zero.

**Fig 3:**
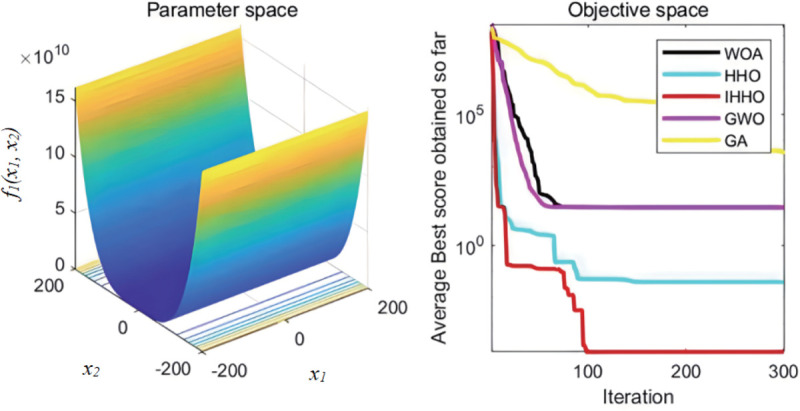
The curve of change of $f_{1}$ fitness.

**Fig 4:**
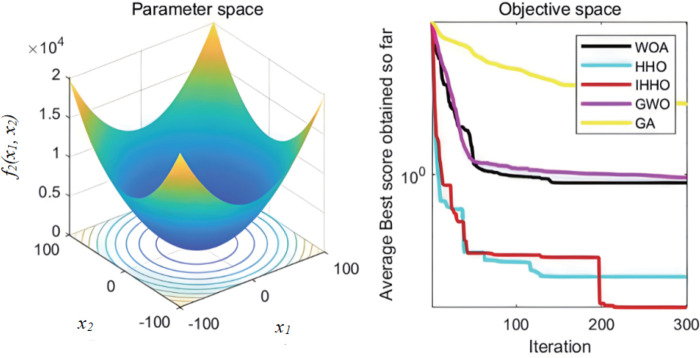
The curve of change of $f_{2}$ fitness.

**Fig 5:**
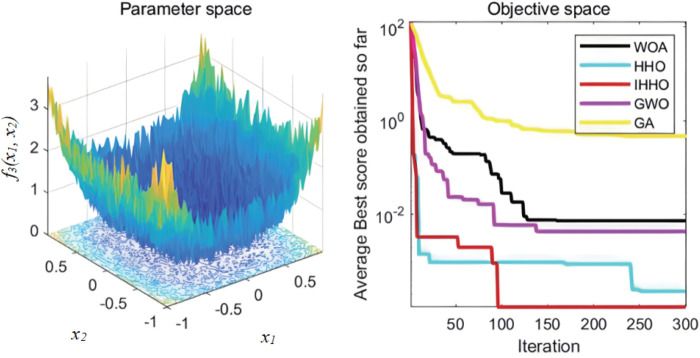
The curve of change of $f_{3}$ fitness.

**Fig 6:**
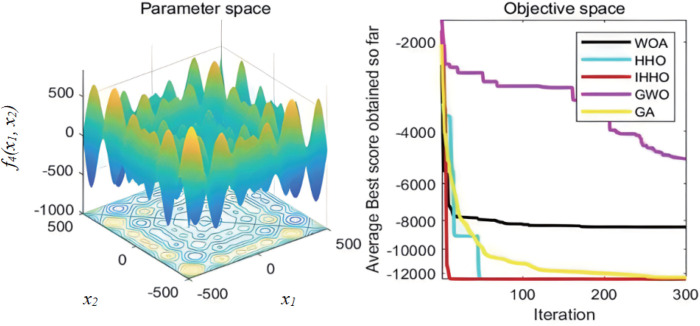
The curve of change of $f_{4}$ fitness.

**Fig 7:**
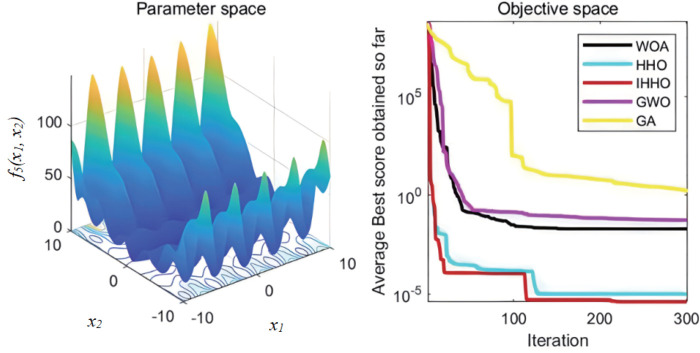
The curve of change of $f_{5}$ fitness.

**Fig 8:**
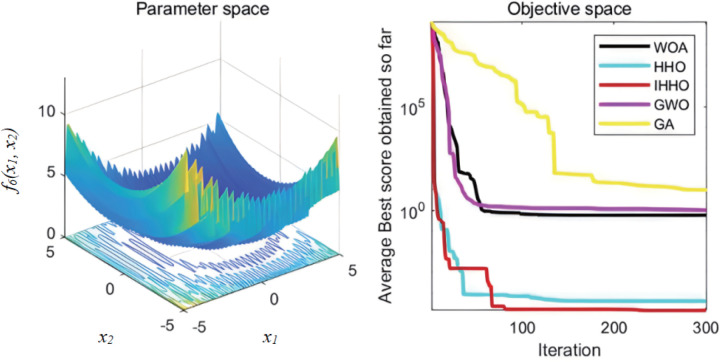
The curve of change of $f_{6}$ fitness.

The collaborative dynamics of GWO are influenced by a cooperative factor vector, along with its convergence attributes. The genetic algorithm’s mutation rate is configured at 0.1, while the probabilities for crossover and selection also play their roles. Each experimental function undergoes 30 isolated evaluations. The graphical representation of the fitness progression for these test functions can be observed in Figs 3–8.

From Figs 3–8, the following analysis and conclusions can be drawn:

(1) The convergence curves of the functions show that GA has the weakest overall optimization capability and the optimization accuracy under most of the test functions is extremely low.
(2) HHO and IHHO both have better convergence speed and optimization accuracy than WOA, GWO, and GA algorithms, proving the advantages of HHO and IHHO.
(3) In terms of convergence precision, IHHO outperforms all other algorithms and demonstrates the swiftest rate of convergence for some of the test functions. For test function$f_{1}$ and$f_{2}$, it can be seen that the convergence accuracy of IHHO is significantly improved. Besides, only the optimal value of IHHO is 0 for multi-peaked function$f_{4}$ ~$f_{6}$, especially the function $f_{4}$ converges to 0 quickly, $f_{5}$ and $f_{6}$ reaches the optimal value in about 100 generations, which shows that the convergence speed becomes faster. $f_{3}$ can intuitively see that there are multiple inflection points in the whole iteration, which proves that the improved algorithm can prevent falling into local optimum.

After executing 30 times for each of the five algorithms, a comparison is made regarding the best outcome, mean value, and standard deviation derived from each test function. The standard deviation serves as an indicator of the algorithm’s stability, whereas the mean assesses the functional convergence precision. These experimental findings are counted as Table 2.

**Table 2:** Comparison of optimization results.

Test functions	Algorithms	Optimum	Average	Standard Deviation
$f_{1}$	WOA	27.8227	28.29417	0.419099
	GWO	27.0108	27.60028	0.7986325
	GA	3575.2695	6288.461	4385.801
	HHO	0.038813	0.03540173	0.05246807
	IHHO	**8.2643 × 10−5**	**0.01422109**	**0.02357525**
$f_{2}$	WOA	0.54815	0.8921187	0.3596076
	GWO	0.80971	1.054148	0.3945553
	GA	176.7767	151.3395	54.7391
	HHO	0.00054998	0.0005130475	**0.000539072**
	IHHO	**0.00005812**	**0.0003281827**	0.0009174721
$f_{3}$	WOA	0.0073638	0.005669792	0.00571261
	GWO	0.0043532	0.003490628	0.001456826
	GA	0.4719	0.4117278	0.143302
	HHO	0.00023228	**0.0002167377**	**0.0003088344**
	IHHO	**0.00010656**	0.0003098534	0.0003299915
$f_{4}$	WOA	−8392.8047	−9989.828	1770.619
	GWO	−4942.8979	−5676.406	1003.224
	GA	−12429.146	−12281.83	89.51645
	HHO	**−12568.907**	−12561.07	**39.779**
	IHHO	−12569.2373	**−12044.86**	1445.467
$f_{5}$	WOA	0.019845	0.04590174	0.03556084
	GWO	0.05444	0.05750024	0.03137766
	GA	1.742	1.656298	0.6448184
	HHO	1.0028 × 10^−5^	1.849791 × 10^−5^	1.990142 × 10^−5^
	IHHO	**3.93 × 10−6**	**8.391934 × 10−6**	**1.047471 × 10−5**
$f_{6}$	WOA	0.57774	0.809324	0.2791133
	GWO	1.0223	0.7895038	0.3014514
	GA	9.5485	7.077904	2.863825
	HHO	4.1368 × 10^−5^	0.0002031807	0.0002027408
	IHHO	**1.4453 × 10−5**	**0.0001186725**	**0.0001496747**

Compared to other algorithms, as revealed in Table 2, IHHO demonstrates the most superior optimization capability. The optimal value of function $f_{1}$,$f_{2}$,$f_{3}$,$f_{5}$,$f_{6}$ is close to 0, and the optimization effect is the best. Similarly, the average value in the middle is the lowest, indicating that IHHO has high convergence accuracy, especially, IHHO standard deviation of $f_{5}$ and $f_{6}$ is very low, indicating that it has high robustness. Obviously, the optimization effect and convergence speed of IHHO are higher than those of the other four algorithms.

### DBN-ELM optimized by IHHO algorithm

**Fig 9:**
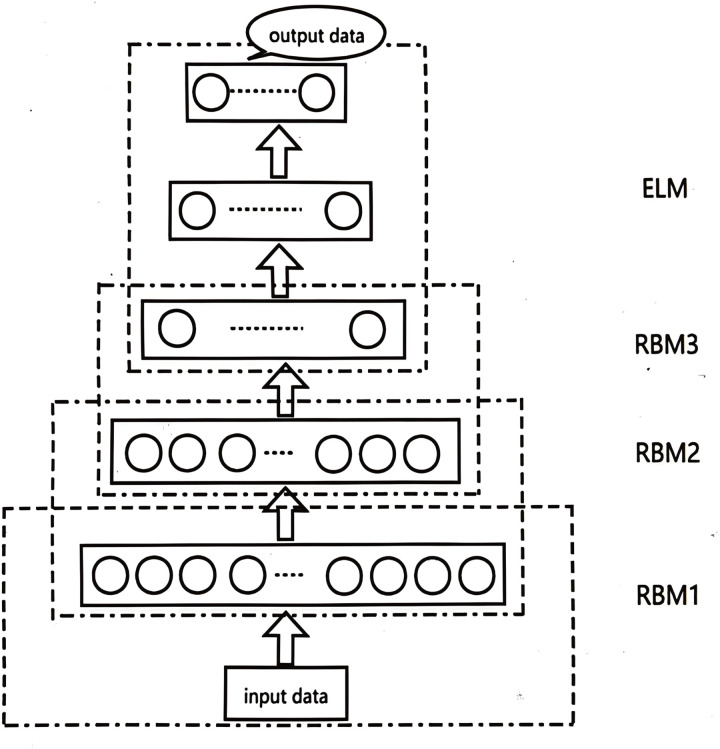
The network structure of DBN-ELM.

The construction of DBN-ELM model involves ELM and DBN, as depicted in Fig 9. The bottom DBN comprises three RBMs, which undergo unsupervised, greedy training for feature extraction. The visual layer, denoted as v, takes in the training set data with the neuron count equivalent to the dataset’s dimension. The top layer is the ELM classification stage, where the input layer is fed by the concealed layer’s output of the third RBM configuration. This innovative DBN-ELM hybrid architecture synergistically combines the complementary advantages of both components: the Deep Belief Network (DBN) demonstrates exceptional capability in hierarchical feature extraction, while the ELM contributes remarkable learning efficiency and robust generalization performance.The integrated framework significantly enhances both classification accuracy and computational efficiency during model training.

The diagnostic performance of the DBN-ELM model is critically dependent on the optimal configuration of hidden layer neurons. Conventional approaches typically determine this hyperparameter through empirical estimation, often resulting in suboptimal model performance and inconsistent diagnostic outcomes. To address this limitation and achieve enhanced model accuracy with accelerated convergence, we implement the Improved Harris Hawks Optimizer (IHHO) for systematic optimization of the hidden layer architecture. The optimization process proceeds through the following key steps:

(1) The optimization process begins with parameter initialization for IHHO, which involves configuring three critical operational parameters: the population size, the maximum iteration limit, and the boundaries. Initialize the positions of Harris hawks population randomly, designating these positions $\left(h_{1},h_{2},h_{3},h_{4}\right)$ as the neuron count in each concealed layer of the model.
(2) The feature vector obtained after data preprocessing is input into DBN for training, and the cross-entropy loss is computed by back-propagation. DBN is fine-tuned and saved. Then, the parameters of DBN are utilized to initialize the feature space of the ELM, and the output weight vector β for ELM is computed.
(3) Utilize the mean precision deviation of the 10-fold cross-validation on the genuine labels and the categorical designations of DBN-ELM as the IHHO fitness function.
(4) Update prey energyE. Utilize the energy E of the prey and a random value r to select and implement the appropriate updating tactic. Subsequently, revise the positioning of the hawk population.
(5) Evaluate if the present iteration counts have attained the terminating criterion; if met, conclude the loop and yield the outcome; otherwise, proceed back to stage 2) for further execution.
(6) Return the optimal individual position prey position and its fitness value. Set the optimal individual position prey position as the structure parameter of DBN-ELM to construct the model.

### Overview of the proposed method

**Fig 10:**
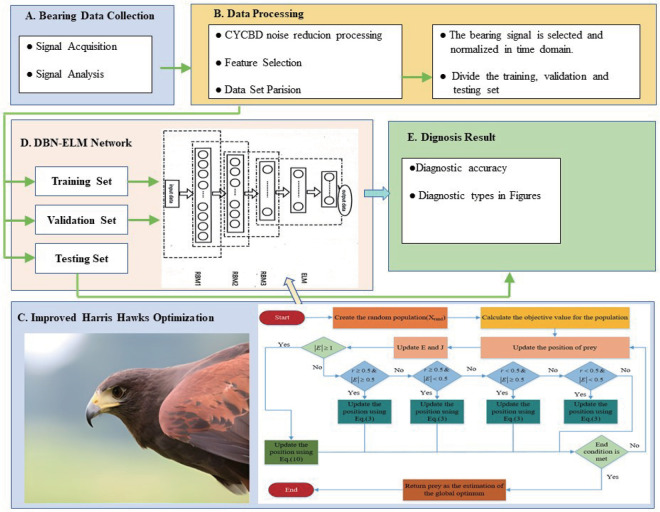
The overall framework of IHHO-DBN-ELM.

The proposed IHHO-DBN-ELM fault detection strategy’s comprehensive architectural progression is depicted in Fig 10. This approach encompasses two distinctive phases: pre-operational training and real-time diagnosis. The pre-operational training segment focuses on optimizing the DBN architecture for heightened effectiveness and efficiency. The online diagnosis part is tested with the model obtained by offline training, and the bearing diagnosis result is obtained. The specific operation process is completed by the following four steps:

(1) Obtain the original signal.
(2) Adopt CYCBD algorithm to denoise the data, and the bearing signal is selected and normalized in time domain. Proceed with segmenting the dataset into separate training and testing sets.
(3) Construct IHHO-DBN-ELM framework, applying IHHO technique to optimize the architectural configuration of DBN-ELM network. Employ the training subset to train the model, thereby preserving the optimal parameters.
(4) Validate the performance of the trained DBN-ELM with the test set.

## Data preprocessing and feature extraction

### Experimental device

**Fig 11:**
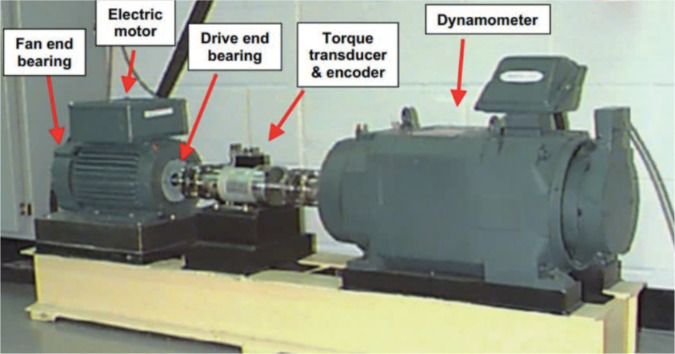
Experiment device.

The proposed IHHO-DBN-ELM algorithm is verified by the bearing data set published of Case Western Reserve University. Experimental device consists of five parts: 1.5 kw electric motor, fan and bearing, drive end bearing torque transducer & encoder, dynamometer, which is shown in Fig 11. In the experiment, single-point faults such as inner ring, outer ring, and rolling body with a diameter of 0.007 mm were caused by electric sparks, and the acceleration and vibration data at the sampling frequency of 12khz were collected. The load is 0 ~ 2HP. Table 3 shows the detailed information of the experimental data on load 0HP.

**Table 3:** The description of fault and sample.

Fault diameter	Fault state	Label	Training/testing
0.007	Normal	1	120/30
0.007	Inner fault	2	120/30
0.007	Rolling fault	3	120/30
0.007	Outer fault	4	120/30

### Data preprocessing

Owing to the intricate operational conditions, the pulse components caused by rotating machinery faults are often interfered by random pulses, periodic harmonics, etc., which affect the fault diagnosis and classification. Therefore, CYCBD algorithm is selected to perform noise reduction preprocessing on the experimental data.

**Fig 12:**
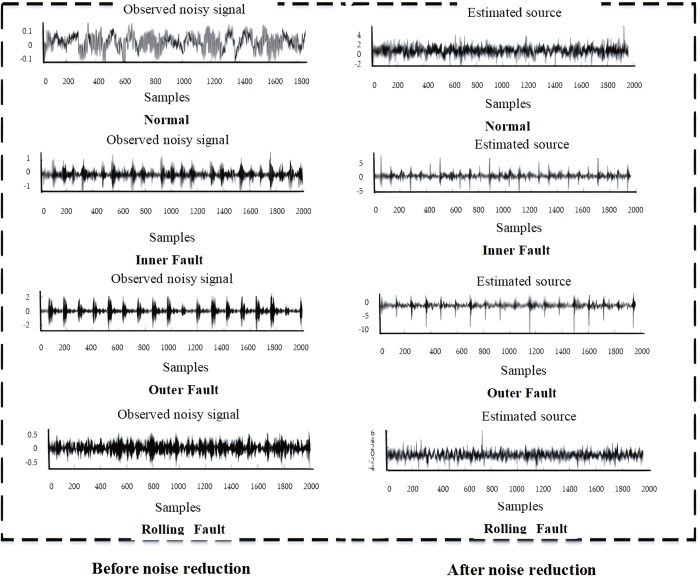
Original signal and noise reduction signal.

CYCBD approach introduces a novel blind deconvolution technique that involves iterative resolution of the feature vector h, to optimize the filter and thereby diminish noise disturbances while emphasizing fault characteristics. The dataset of 2000 samples from four distinct fault scenarios (normal, inner fault, rolling fault, and outer fault) under 0 load was selected. These were subjected to noise reduction treatment using the CYCBD method. As depicted in Fig 12, the left side displays the observed noisy signal after noisy reduction, while the right side exhibits the estimated source. It is demonstrates enhanced periodic patterns and pulse clarity, significantly mitigating noise interference on the right side of Fig 12, which is more conducive to extracting fault features.

### Feature selection

Time domain features are often applied in fault diagnosis because of their simplicity and easy availability. Therefore, temporal features are selected to train the proposed IHHO-DBN-ELM. The time domain statistical method mainly consists of waveform observation and time domain numerical calculation. Table 4 exhibits the details of common time-domain characteristic formulas.

**Table 4:** The description of time domain features.

Time domain feature name	Formula
**Mean value**	$\mu_{x}=\frac{1}{n}\sum_{i=1}^{n}\left|x_{i}\right|$
**Peak value** $x_{p}$	$x_{p}=\max\left|x_{i}\right|$
**Root mean square value**	$x_{rms}=\sqrt{\frac{1}{N}\sum_{i=1}^{n}x_{i}^{2}}$
**Variance**	$S^{2}=\frac{1}{N-1}\sum {\mathrm{\backslash nolimits}}_{n=1}^{N}{\left(x\left(n\right)-\bar{X}\right)}^{2}$
**Root amplitude**	$X_{r}=(\frac{1}{N}\sum_{n=1}^{N}\sqrt{\left|x(n)\right|}{)}^{2}$
**Peak-to-peak value**	$X_{ppv}=\max(x(n))-\min(x(n))$
**Skewness**	$\alpha =\frac{1}{N}\sum_{n=1}^{N}x^{3}(n)$
**Kurtosis**	$\beta =\frac{1}{N}\sum_{n=1}^{N}x_{4}(n)$
**Waveform index**	$S_{f}=\frac{\sqrt{\frac{\sum {\mathrm{\backslash nolimits}}_{n=1}^{N}\left(x{\left(n\right)}^{2}\right)}{N}}}{\bar{X}}$
**Peak indicator**	$I_{p}=\frac{X_{p}}{\sqrt{\frac{\sum {\mathrm{\backslash nolimits}}_{n=1}^{N}\left(x{\left(n\right)}^{2}\right)}{N}}}$
**Pulse indicator**	$C_{f}=\frac{X_{p}}{\bar{X}}$
**Abundance index**	$C_{e}=\frac{X_{p}}{X_{r}}$
**Deviation index**	$P={\frac{\frac{1}{N}\sum {\mathrm{\backslash nolimits}}_{n=1}^{N}\left(x\left(n\right)-\bar{X}\right)}{{\left(\sqrt{\frac{\sum {\mathrm{\backslash nolimits}}_{n=1}^{N}\left(x{\left(n\right)}^{2}\right)}{N}}\right)}^{3}}}^{2}$
**Kurtosis index**	$K_{v}=\frac{\beta }{(\sqrt{\frac{\sum {\mathrm{\backslash nolimits}}_{n=1}^{N}\left(x{\left(n\right)}^{2}\right)}{N}{)}^{4}}}$

If 14 time-domain features in Table 4 are formed into a feature matrix, the amount of computation will increase and the classification efficiency will be affected. Hence, the dataset’s dimensionality is reduced through the utilization of Principal Component Analysis (PCA), and four kinds of time-domain characteristic parameters whose cumulative contribution rate is greater than 95% are selected. The four time-domain features are mean, peak, standard deviation, and amplitude sum of squares. That is, the four parameters preserved through PCA dimensionality reduction retain 95% of the important information in all parameters, but can greatly improve computational efficiency.

## Experiments

### Bearing fault diagnosis based on IHHO-DBN-ELM

**Fig 13:**
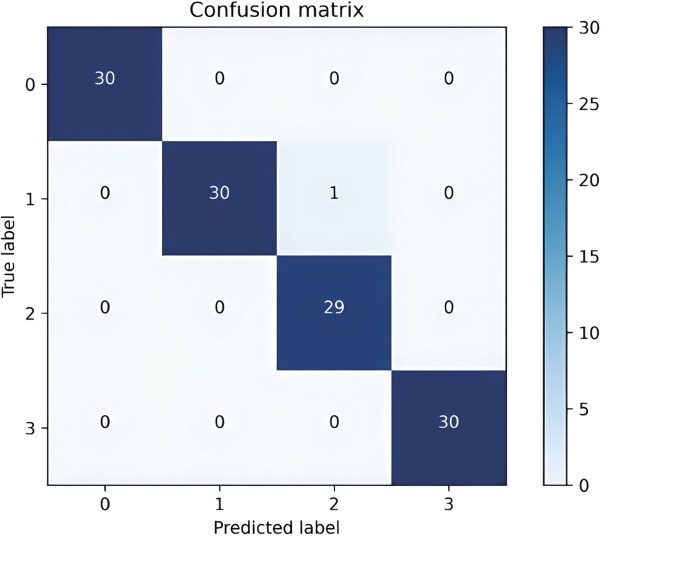
IHHO-DBN-ELM confusion matrix.

To comprehensively evaluate the performance of the proposed IHHO-DBN-ELM framework, we established an experimental protocol using 240,000 preprocessed bearing vibration records collected under no-load conditions (0 HP). The dataset was strategically partitioned with an 80:20 ratio for training and testing purposes, respectively. The optimization parameters were configured as follows: population size is 30, maximum iterations is 300, and searching space boundaries belong to [100, 500] for hidden neuron counts. The visibility layer count is fixed at 400. Through IHHO optimization, we identified an optimal DBN-ELM architecture of 400-129-375-375-165-4. The classification performance is quantitatively demonstrated through the confusion matrix presented in Fig 13, which reveals the model’s diagnostic accuracy across different fault categories.

As depicted in Fig 13, the proposed IHHO-DBN showcases remarkable performance with commendable precision in its identification capabilities. Notably, a solitary misclassification occurred where a test specimen was misidentified, assigning it to a rolling fault instead of the intended inner fault. The recognition accuracy of the entire test set reached 99.17%, which shows that the fault types can be effectively identified.

**Fig 14:**
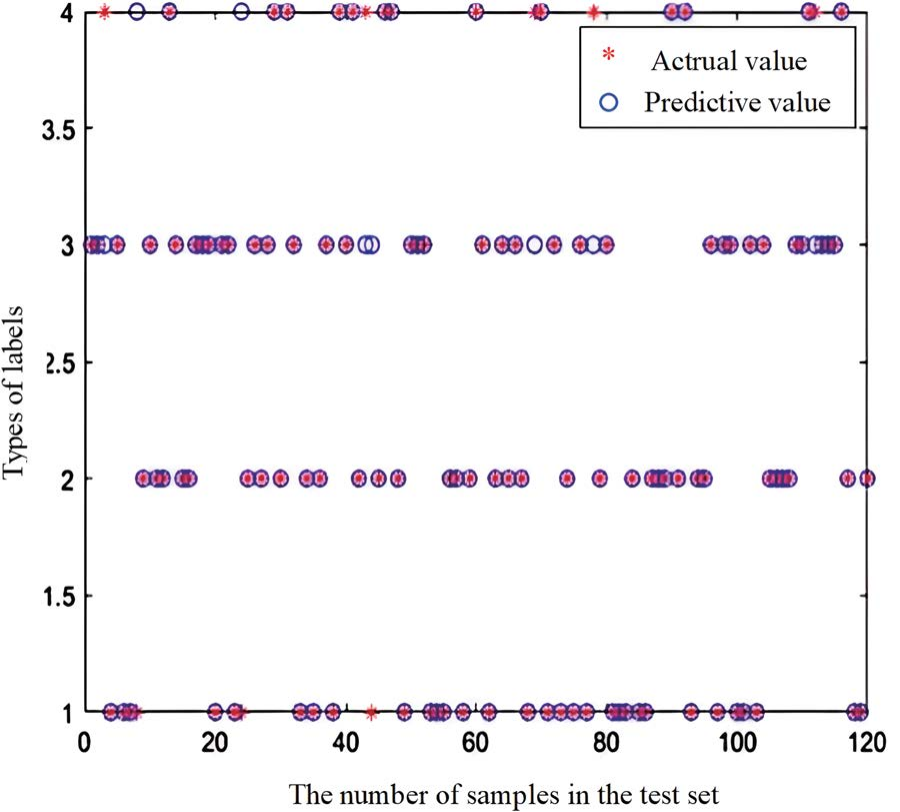
HHO-DBN-ELM diagnostic accuracy.

**Fig 15:**
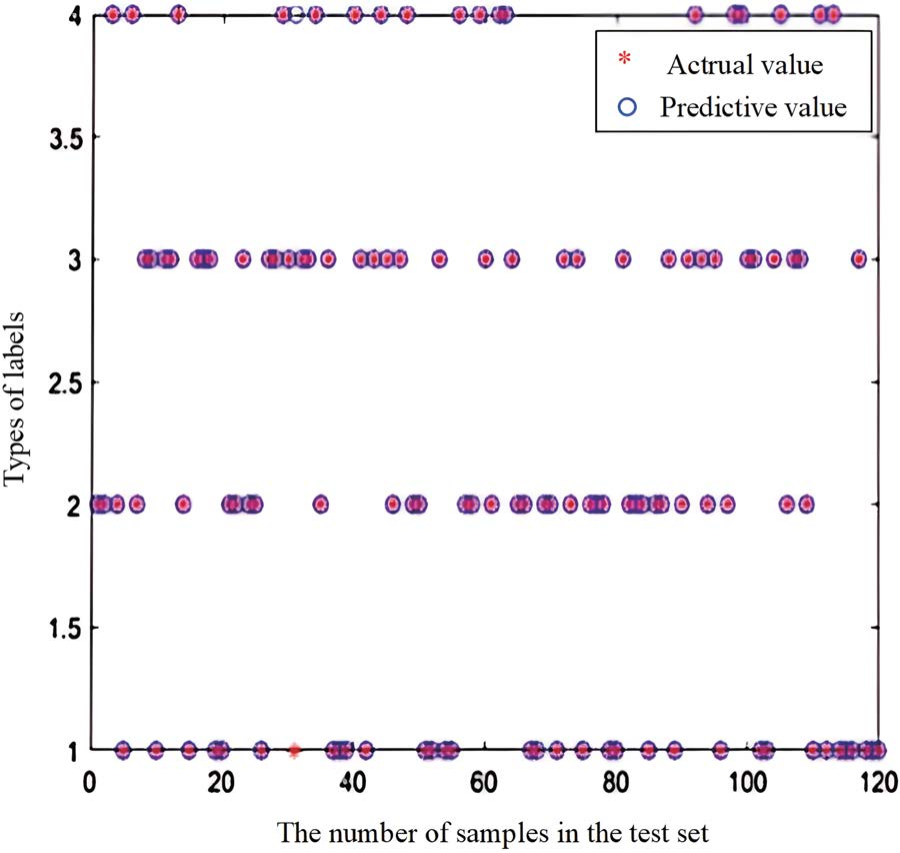
IHHO-DBN-ELM diagnostic accuracy.

To demonstrate the effectiveness performance of the proposed method, identical datasets are utilized for both training and testing phases for IHHO-DBN-ELM and HHO-DBN-ELM. The examination of the test set outcomes can be observed in Figs 14 and 15. The diagnostic accuracy of HHO-DBN-ELM is 93.33%, while the diagnostic accuracy of IHHO-DBN-ELM reached 99.17%. Observations reveal that IHHO-DBN-ELM has effectively pinpointed optimal hyperparameters through tuning the quantity of hidden layer neurons and enhancing the training approach, thereby maximizing the exceptional capabilities of DBN-ELM.

### Comparison experiments of optimized DBN-ELM models

To substantiate the model’s credibility further, the mean diagnostic precision was ascertained through five replicate tests employing IHHO-DBN-ELM, HHO-DBN-ELM, GWO-DBN-ELM, and DBN-ELM frameworks. These comparisons are depicted in Table 5. Observations reveal that the initial DBN-ELM’s average accuracy falls below 90%. Conversely, the swarm algorithm-optimized models exhibit notably superior diagnostic precision compared to the base DBN-ELM. Specifically, the IHHO-DBN-ELM achieves an average accuracy of 97.53%, consistently outperforming HHO-DBN-ELM across all experiments, demonstrating that the IHHO algorithm optimizes DBN-ELM’s performance more effectively than its baseline counterpart. Consequently, the proposed IHHO-DBN-ELM method demonstrates the highest diagnostic efficacy.

**Table 5:** The accuracy under different models.

	Accuracy			
Experiment No.	DBN-ELM	GWO-DBN-ELM	HHO-DBN-ELM	IHHO-DBN-ELM
1	89.75%	93.87%	93.33%	**97.13%**
2	87.67%	95.09%	95.87%	**98.16%**
3	90.12%	92.37%	94.12%	**96.58%**
4	89.36%	94.53%	95.00%	**96.67%**
5	92.68%	93.50%	93.87%	**99.12%**
Mean	89.92%	93.87%	94.44%	**97.53%**

### Comparison experiment of common AI diagnostic models

As shown in Table 6, compared with BP neural network, SVM model, RBF model and CNN model proposed by scholars in recent years, the experimental results demonstrate significant performance advantages of the proposed deep learning framework over conventional shallow models. Specifically, our IHHO-DBN-ELM architecture achieves a remarkable classification accuracy of 96.73%, representing a substantial improvement over traditional shallow networks which typically attain only 85–88% accuracy on similar diagnostic tasks. While maintaining competitive computational efficiency with an average inference time of 0.73 seconds per test sample, well within the sub-second requirement for real-time industrial applications, the proposed method exhibits superior diagnostic precision compared to both individual deep networks and shallow models. These findings suggest that the IHHO-DBN-ELM framework not only provides enhanced classification performance but also maintains practical applicability, indicating strong potential for implementation in industrial fault diagnosis systems.

**Table 6:** Experimental comparison results of common AI diagnostic models.

Model	Accuracy	Test time
**BPNN**	86.71%	**0.10**
**SVM**	88.75%	0.17
**RBF**	85.32%	0.19
**CNN**	95.12%	0.58
**IHHO-DBN-ELM**	**96.73%**	0.73

### Comparative experiments under different data sets

Accuracy is an important indicator for evaluating IHHO-DBN-ELM intelligent fault diagnosis. The higher the accuracy, the better the diagnosis effect. In order to enrich the coverage of sample selection, this paper adjusts the sampling frequency, truncates the original bearing data into a sample length according to different points, and obtains 3000, 6000, and 10000 pieces of data to form a test set for the next operation. The ratio of the number of healthy and unhealthy data is 1:3, and as depicted in Table 7, it is evident that Table 7 that the IHHO-DBN intelligent fault diagnosis system has a high classification accuracy under different test set quantities, meeting the expected goals.

**Table 7:** Accuracy test of artificial intelligence diagnosis model.

Test No.	Number of total samples	Number of normal samples	Number of fault samples	Accuracy
**1**	3000	750	2250	95.83%
**2**	6000	1500	4500	97.78%
**3**	10000	2500	7500	98.18%

## Conclusions

This study proposes an advanced bearing fault detection method that integrates an Improved Harris Hawks Optimization (IHHO) algorithm with a Deep Belief Network-Extreme Learning Machine (DBN-ELM) architecture. The proposed framework addresses two critical challenges in rotating machinery fault diagnosis: (1) noise contamination that masks incipient fault features in vibration signals; (2) suboptimal network architecture in conventional deep learning approaches. The main work of this paper includes two parts: methodology and experimental validation, which are described as follows:

In aspect of the methodology:(1) Signal Preprocessing: The raw vibration signals are first enhanced using Maximum Correlated Kurtosis Deconvolution (CYCBD) to amplify fault-related periodic impulses while suppressing noise interference. (2) Feature Learning: The denoised time-domain signals serve as input to the DBN, which automatically extracts discriminative fault features through its hierarchical structure of Restricted Boltzmann Machines. (3) Model Optimization: IHHO algorithm determines the optimal DBN-ELM architecture by introducing a differential evolution mutation operator to preserve population diversity and implementing a nonlinear escape energy factor to dynamically balance exploration and exploitation. (4) Fault Classification: The optimized DBN-ELM model performs final fault pattern recognition.

In aspect of experimental validation: Evaluation on the Case Western Reserve University (CWRU) bearing dataset demonstrates superior performance: 99.17% classification accuracy, 97.53% average precision, which has significant improvement over conventional methods.

While achieving excellent diagnostic performance, the method presents two limitations: strong dependency on effective noise removal during preprocessing and increased computational requirements compared to traditional techniques. Future work will focus on optimizing computational efficiency for real-time applications and enhancing generalization capability under varying operational conditions.

## Supporting information

S1 File Data. (PDF)
