# Supplementary material for: A bearing fault diagnosis method based on hybrid artificial intelligence models
Source: PLoS One. 2025 Jul 31;20(7):e0327646. doi: 10.1371/journal.pone.0327646 (PMC12312945; doi:10.1371/journal.pone.0327646)
Supplement: S1 File — (PDF) [file pone.0327646.s001.pdf]

| Time serises | nomal(gm/s²) | Inner fault (gm/s²) | Outer fault(gm/s²) | Rolling fault(gm/s²) |
|---|---|---|---|---|
| 1 | 0.0461040000000000 | -0.277601636726547 | -0.110455888223553 | -0.0721211976047904 |
| 2 | -0.0371335384615385 | -0.0443447904191617 | 0.215632634730539 | 0.303103952095808 |
| 3 | -0.0894960000000000 | 0.117603033932136 | 0.0799993013972056 | 0.0331367664670659 |
| 4 | -0.0849064615384615 | -0.145054570858283 | -0.317966766467066 | -0.207267225548902 |
| 5 | -0.0385938461538462 | -0.111430499001996 | 0.0231470059880240 | 0.0969737724550898 |
| 6 | 0.0254510769230769 | 0.130922714570858 | 0.177460379241517 | 0.174455329341317 |
| 7 | 0.0406800000000000 | 0.0328118962075848 | 0.0998976047904192 | -0.0869027944111777 |
| 8 | 0.0425575384615385 | -0.197033812375250 | -0.231876147704591 | -0.0550655089820359 |
| 9 | 0.0381766153846154 | -0.0748825948103793 | -0.0621314371257485 | 0.130922714570858 |
| 10 | 0.0440178461538462 | 0.00958367265469062 | 0.0117765469061876 | 0.00438574850299401 |
| 11 | 0.0421403076923077 | -0.128161317365269 | -0.0678166666666667 | -0.0972986427145709 |
| 12 | -0.0114738461538462 | 0.178678642714571 | 0.0276139720558882 | 0.0578269061876247 |
| 13 | -0.0767704615384615 | 0.392280838323353 | -0.155125548902196 | 0.0639994411177645 |
| 14 | -0.147073846153846 | 0.0484056686626747 | 0.0564462075848303 | -0.0857657485029940 |
| 15 | -0.152915076923077 | 0.0604258682634731 | 0.267205788423154 | -0.0628623952095808 |
| 16 | -0.0955458461538462 | 0.116953293413174 | 0.191267365269461 | 0.0948621157684631 |
| 17 | -0.0308750769230769 | -0.0735831137724551 | -0.240403992015968 | 0.0909636726546906 |
| 18 | 0.0289975384615385 | 0.0531162874251497 | -0.0609131736526946 | 0.00795932135728543 |
| 19 | 0.0312923076923077 | 0.234556327345309 | 0.174617764471058 | 0.0186800399201597 |
| 20 | 0.0185667692307692 | 0.176729421157685 | -0.0393905189620759 | 0.0419082634730539 |
| 21 | 0.00146030769230769 | -0.0800805189620758 | -0.150658582834331 | -0.0146191616766467 |
| 22 | -0.00521538461538462 | 0.00568522954091816 | -0.128729840319361 | 0.00519792415169661 |
| 23 | -0.0168978461538462 | 0.124100439121756 | 0.125075049900200 | 0.0323245908183633 |
| 24 | -0.0909563076923077 | -0.341925948103792 | 0.0714714570858283 | -0.0922631536926148 |
| 25 | -0.137060307692308 | -0.655263313373254 | -0.0889332335329341 | -0.0602634331337325 |
| 26 | -0.145613538461538 | -0.185663353293413 | -0.0572583832335329 | 0.112567544910180 |
| 27 | -0.0990923076923077 | 0.296444111776447 | 0.208729141716567 | 0.103958483033932 |
| 28 | -0.0118910769230769 | 0.114354331337325 | 0.0568522954091816 | -0.0703344111776447 |
| 29 | 0.0438092307692308 | 0.0305378043912176 | -0.216038722554890 | -0.0778064271457086 |
| 30 | 0.0657138461538462 | 0.0575020359281437 | 0.0479183632734531 | 0.128648622754491 |
| 31 | 0.0319181538461538 | 0.0328118962075848 | 0.164871656686627 | 0.183064391217565 |
| 32 | 0.0123083076923077 | 0.183876566866267 | -0.0771566866267465 | -0.0833292215568862 |
| 33 | 0.0114738461538462 | 0.0266393612774451 | -0.139694211576846 | -0.173318283433134 |
| 34 | -0.0131427692307692 | -0.273053453093812 | 0.0609131736526946 | 0.0740704191616766 |
| 35 | -0.0638363076923077 | 0.00568522954091816 | 0.114110678642715 | 0.0690349301397206 |
| 36 | -0.148116923076923 | 0.463102554890220 | 0.0698471057884232 | -0.113542155688623 |
| 37 | -0.198810461538462 | 0.377824111776447 | 0.0312687624750499 | -0.103146307385230 |
| 38 | -0.190883076923077 | 0.163247305389222 | 0.0820297405189621 | -0.0302129341317365 |
| 39 | -0.127881230769231 | 0.00828419161676647 | 0.136851596806387 | 0.00649740518962076 |
| 40 | -0.0552830769230769 | -0.0644867465069860 | 0.176242115768463 | 0.0693598003992016 |
| 41 | -0.0256596923076923 | 0.0319997205588822 | 0.0446696606786427 | 0.00552279441117765 |
| 42 | -0.0148116923076923 | -0.107856926147705 | -0.127917664670659 | -0.111268063872255 |
| 43 | -0.0179409230769231 | -0.223510738522954 | 0.111268063872255 | -0.000487305389221557 |

| | | | | |
|---|---|---|---|---|
| 44 | -0.00375507692307692 | -0.0919382834331337 | 0.210353493013972 | 0.115491377245509 |
| 45 | 0.00146030769230769 | -0.0251774451097804 | -0.0211165668662675 | -0.0105582834331337 |
| 46 | -0.0396369230769231 | -0.0540908982035928 | -0.108019361277445 | -0.172181237524950 |
| 47 | -0.0828203076923077 | 0.0144567265469062 | 0.114922854291417 | -0.0142942914171657 |
| 48 | -0.137060307692308 | 0.0605883033932136 | 0.189643013972056 | 0.174617764471058 |
| 49 | -0.150828923076923 | -0.247876007984032 | -0.0450757485029940 | -0.0292383233532934 |
| 50 | -0.109523076923077 | -0.318535289421158 | -0.00852784431137725 | -0.163409740518962 |
| 51 | -0.0563261538461538 | 0.127024271457086 | -0.0162435129740519 | 0.0859281836327345 |
| 52 | -0.00312923076923077 | 0.198495728542914 | -0.0759384231536926 | 0.164384351297405 |
| 53 | -0.0135600000000000 | 0.00227409181636727 | -0.160404690618762 | -0.0493802794411178 |
| 54 | -0.0306664615384615 | 0.150577365269461 | -0.167714271457086 | -0.0391468662674651 |
| 55 | -0.0327526153846154 | 0.237480159680639 | -0.121826347305389 | 0.0347611177644711 |
| 56 | -0.0283716923076923 | 0.0281012774451098 | -0.216444810379242 | -0.0229033532934132 |
| 57 | -0.0154375384615385 | 0.0198170858283433 | -0.0747201596806387 | -0.0190049101796407 |
| 58 | -0.0540313846153846 | 0.0583142115768463 | 0.0958367265469062 | -0.00665984031936128 |
| 59 | -0.0801083076923077 | -0.250799840319361 | -0.00934001996007984 | -0.0940499401197605 |
| 60 | -0.0884529230769231 | -0.224647784431138 | -0.119389820359281 | -0.0743952894211577 |
| 61 | -0.0429747692307692 | 0.260870818363273 | 0.610350000000000 | 0.183226826347305 |
| 62 | 0.0509021538461538 | 0.190211536926148 | 0.233500499001996 | 0.109318842315369 |
| 63 | 0.0926252307692308 | -0.266718483033932 | -1.18333992015968 | -0.254210978043912 |
| 64 | 0.110983384615385 | -0.0882022754491018 | -0.0255835329341317 | -0.0856033133732535 |
| 65 | 0.0805255384615385 | 0.0841413972055888 | 1.86597355289421 | 0.320159640718563 |
| 66 | 0.0490246153846154 | -0.231470059880240 | -0.456442714570858 | 0.0474310578842315 |
| 67 | 0.0552830769230769 | -0.221074211576846 | -1.98536337325349 | -0.290758882235529 |
| 68 | 0.0490246153846154 | -0.0259896207584830 | 1.53866676646707 | 0.0706592814371258 |
| 69 | 0.0379680000000000 | -0.0194922155688623 | 1.66252355289421 | 0.307977005988024 |
| 70 | -0.0187753846153846 | 0.0313499800399202 | -1.82780129740519 | -0.0323245908183633 |
| 71 | -0.0569520000000000 | 0.249987664670659 | -1.03308742514970 | -0.159836167664671 |
| 72 | -0.0388024615384615 | 0.302941516966068 | 1.85013612774451 | 0.177704031936128 |
| 73 | 0.0100135384615385 | 0.0596136926147705 | 0.265987524950100 | 0.141480998003992 |
| 74 | 0.0888701538461539 | -0.0862530538922156 | -1.58699121756487 | -0.161947824351297 |
| 75 | 0.107645538461538 | -0.0609131736526946 | 0.447508782435130 | -0.0342738123752495 |
| 76 | 0.0853236923076923 | -0.0461315768463074 | 0.630654391217565 | 0.143917524950100 |
| 77 | 0.0475643076923077 | -0.0729333732534930 | -0.911667165668663 | -0.0696846706586826 |
| 78 | 0.0216960000000000 | -0.0334616367265469 | 0.0950245508982036 | -0.162922435129741 |
| 79 | 0.0456867692307692 | 0.0711465868263473 | 0.746795508982036 | 0.0830043512974052 |
| 80 | 0.0390110769230769 | 0.100709780439122 | -0.966895109780439 | 0.0259896207584830 |
| 81 | 0.0106393846153846 | 0.0190049101796407 | -0.464564471057884 | -0.182739520958084 |
| 82 | -0.0229476923076923 | 0.00227409181636727 | 1.45054570858283 | 0.00519792415169661 |
| 83 | -0.0354646153846154 | 0.0230657884231537 | 0.0190861277445110 | 0.160973213572854 |
| 84 | 0.0116824615384615 | -0.169419840319361 | -1.49643363273453 | -0.105745269461078 |
| 85 | 0.0636276923076923 | -0.202881477045908 | 0.338677245508982 | -0.112729980039920 |
| 86 | 0.0940855384615385 | 0.0713090219560878 | 1.61379301397206 | 0.203368782435130 |
| 87 | 0.0732240000000000 | 0.119227385229541 | -0.482026247504990 | 0.100547345309381 |

| | | | | |
|---|---|---|---|---|
| 88 | 0.0289975384615385 | -0.134658722554890 | -1.54191546906188 | -0.228708662674651 |
| 89 | 0.00479815384615385 | -0.130110538922156 | 0.579081237524950 | -0.121988782435130 |
| 90 | 0.00375507692307692 | -0.243815129740519 | 1.05907704590818 | 0.205805309381238 |
| 91 | 0.0160633846153846 | -0.216038722554890 | -0.650552694610779 | 0.0566898602794411 |
| 92 | -0.0110566153846154 | 0.846124590818363 | -0.760196407185629 | -0.128811057884232 |
| 93 | -0.0548658461538462 | 0.987280718562874 | 0.534411576846307 | 0.115653812375249 |
| 94 | -0.0869926153846154 | 0.143755089820359 | 0.488117564870260 | 0.188912055888224 |
| 95 | -0.0759360000000000 | -0.237642594810379 | -0.428016566866267 | -0.0506797604790419 |
| 96 | -0.0127255384615385 | -0.325357564870259 | -0.0897454091816367 | 0.00178678642714571 |
| 97 | 0.0329612307692308 | 0.0313499800399202 | 0.358981636726547 | 0.180790299401198 |
| 98 | 0.0463126153846154 | 0.127998882235529 | -0.0235530938123753 | 0.0212790019960080 |
| 99 | 0.0162720000000000 | -0.513132574850299 | -0.0804053892215569 | -0.0828419161676647 |
| 100 | -0.0196098461538462 | -0.834104391217565 | 0.272484930139721 | 0.00454818363273453 |
| 101 | -0.0129341538461538 | 0.369539920159681 | 0.0198983033932136 | 0.0472686227544910 |
| 102 | 0.00625846153846154 | 1.04738171656687 | -0.199795209580838 | -0.0167308183632735 |
| 103 | 0.0154375384615385 | -0.313824670658683 | 0.319591117764471 | -0.0344362475049900 |
| 104 | -0.0250338461538462 | -0.768805469061876 | 0.356951197604790 | 0.0665984031936128 |
| 105 | -0.0744756923076923 | 0.352646666666667 | -0.239997904191617 | 0.0820297405189621 |
| 106 | -0.0703033846153846 | 0.376849500998004 | -0.316342415169661 | -0.0142942914171657 |
| 107 | -0.0233649230769231 | -0.409823832335329 | 0.160810778443114 | -0.0112080239520958 |
| 108 | 0.0394283076923077 | -0.237805029940120 | 0.147003792415170 | 0.0386595608782435 |
| 109 | 0.0504849230769231 | 0.0136445508982036 | -0.488117564870260 | -0.0243652694610778 |
| 110 | 0.0273286153846154 | -0.0178678642714571 | -0.295225848303393 | -0.0349235528942116 |
| 111 | 0.000625846153846154 | 0.432727185628742 | 0.435326147704591 | -0.0144567265469062 |
| 112 | -0.00542400000000000 | 0.564786946107785 | 0.00162435129740519 | -0.0206292614770459 |
| 113 | 0.0502763076923077 | -0.198658163672655 | -0.509234131736527 | -0.0886895808383234 |
| 114 | 0.0821944615384615 | -0.174292894211577 | 0.0515731536926148 | -0.0469437524950100 |
| 115 | 0.0673827692307692 | 0.486980518962076 | 0.579893413173653 | 0.0921007185628743 |
| 116 | 0.0310836923076923 | 0.00568522954091816 | -0.137663772455090 | -0.00129948103792415 |
| 117 | 0.00146030769230769 | -0.542858203592814 | -0.516137624750499 | -0.0963240319361277 |
| 118 | 0.0196098461538462 | -0.0588015169660679 | 0.310657185628743 | 0.0680603193612775 |
| 119 | 0.0536141538461538 | 0.291733493013972 | 0.417864371257485 | 0.188262315369261 |
| 120 | 0.0915821538461538 | -0.159511297405190 | -0.224972654690619 | -0.0305378043912176 |
| 121 | 0.0761446153846154 | -0.213927065868264 | -0.302941516966068 | -0.169907145708583 |
| 122 | 0.0444350769230769 | 0.192323193612774 | 0.320403293413174 | -0.0354108582834331 |
| 123 | 0.0415144615384615 | 0.0617253493013972 | 0.205480439121756 | 0.0326494610778443 |
| 124 | 0.0515280000000000 | -0.235206067864271 | -0.0958367265469062 | -0.112892415169661 |
| 125 | 0.0828203076923077 | -0.0347611177644711 | -0.0178678642714571 | -0.0974610778443114 |
| 126 | 0.0638363076923077 | 0.105907704590818 | -0.0540096806387226 | 0.0682227544910180 |
| 127 | 0.0440178461538462 | -0.113704590818363 | 0.108831536926148 | 0.0173805588822355 |
| 128 | 0.0310836923076923 | -0.0287510179640719 | 0.136445508982036 | 0.0125075049900200 |
| 129 | 0.0310836923076923 | 0.107369620758483 | 0.0483244510978044 | 0.0573396007984032 |
| 130 | 0.0840720000000000 | 0.153338762475050 | -0.269642315369262 | 0.0199795209580838 |
| 131 | 0.114947076923077 | 0.199795209580838 | -0.0397966067864271 | -0.0419082634730539 |

| | | | | |
|---|---|---|---|---|
| 132 | 0.109731692307692 | 0.0141318562874252 | 0.332585928143713 | 0.0350859880239521 |
| 133 | 0.0719723076923077 | -0.204018522954092 | 0.0974610778443114 | 0.138719600798403 |
| 134 | 0.0344215384615385 | 0.00665984031936128 | -0.164465568862275 | 0.0224160479041916 |
| 135 | 0.0406800000000000 | 0.270779361277445 | -0.0263957085828343 | -0.0339489421157685 |
| 136 | 0.0709292307692308 | 0.00406087824351297 | 0.437356586826347 | 0.0492178443113772 |
| 137 | 0.0834461538461538 | -0.188262315369261 | 0.0986793413173653 | 0.0274515369261477 |
| 138 | 0.0579950769230769 | -0.0959991616766467 | -0.313499800399202 | 0.00714714570858283 |
| 139 | 0.000208615384615385 | -0.0661110978043912 | 0.0263957085828343 | 0.106232574850299 |
| 140 | -0.0398455384615385 | -0.131085149700599 | 0.295225848303393 | -0.0177054291417166 |
| 141 | -0.0229476923076923 | -0.0116953293413174 | -0.247713572854291 | -0.119389820359281 |
| 142 | 0.0285803076923077 | 0.0730958083832335 | -0.394311277445110 | 0.118577644710579 |
| 143 | 0.0569520000000000 | -0.172830978043912 | 0.177460379241517 | 0.140506387225549 |
| 144 | 0.0267027692307692 | -0.0817048702594810 | 0.0698471057884232 | -0.177379161676647 |
| 145 | -0.0125169230769231 | 0.216363592814371 | -0.285073652694611 | -0.198495728542914 |
| 146 | -0.0210701538461538 | 0.00194922155688623 | -0.114516766467066 | 0.159348862275449 |
| 147 | 0.00584123076923077 | -0.177379161676647 | 0.174211676646707 | 0.0929128942115768 |
| 148 | 0.0375507692307692 | 0.142942914171657 | 0.0950245508982036 | -0.341925948103792 |
| 149 | 0.0385938461538462 | 0.159348862275449 | -0.0613192614770459 | -0.218312814371257 |
| 150 | 0.00897046153846154 | -0.205318003992016 | -0.159592514970060 | 0.223023433133733 |
| 151 | -0.0244080000000000 | -0.0654613572854292 | -0.0434513972055888 | 0.122638522954092 |
| 152 | -0.0164806153846154 | 0.177054291417166 | 0.0873088822355290 | -0.210678363273453 |
| 153 | 0.00876184615384615 | -0.134983592814371 | 0.101115868263473 | -0.101359520958084 |
| 154 | 0.0285803076923077 | -0.0786186027944112 | -0.188424750499002 | 0.137582554890220 |
| 155 | 0.0143944615384615 | 0.327144351297405 | -0.213602195608782 | 0.0854408782435130 |
| 156 | -0.0277458461538462 | 0.114841636726547 | 0.305378043912176 | -0.0177054291417166 |
| 157 | -0.0398455384615385 | -0.332179840319361 | 0.292789321357285 | -0.0103958483033932 |
| 158 | -0.0237821538461538 | -0.186475528942116 | -0.122232435129741 | 0.0118577644710579 |
| 159 | 0.0319181538461538 | 0.198495728542914 | -0.235124850299401 | 0.111592934131737 |
| 160 | 0.0573692307692308 | -0.0188424750499002 | 0.177054291417166 | 0.172018802395210 |
| 161 | 0.0237821538461538 | -0.104120918163673 | 0.219287425149701 | -0.00633497005988024 |
| 162 | 0.00125169230769231 | 0.00389844311377246 | -0.151876846307385 | -0.112242674650699 |
| 163 | -0.00208615384615385 | -0.164059481037924 | -0.242840518962076 | 0.0914509780439122 |
| 164 | 0.0256596923076923 | -0.254048542914172 | 0.0625375249500998 | 0.195571896207585 |
| 165 | 0.0492332307692308 | -0.145541876247505 | 0.182333433133733 | -0.0440199201596806 |
| 166 | 0.0369249230769231 | 0.222048822355289 | -0.118577644710579 | -0.183876566866267 |
| 167 | -0.00500676923076923 | 0.558939281437126 | -0.0629436127744511 | 0.0550655089820359 |
| 168 | -0.0640449230769231 | 0.378148982035928 | 0.0414209580838323 | 0.244952175648703 |
| 169 | -0.0613329230769231 | 0.0245277045908184 | -0.00203043912175649 | 0.0422331337325349 |
| 170 | 0.00166892307692308 | 0.110455888223553 | -0.0657862275449102 | -0.138069860279441 |
| 171 | 0.0506935384615385 | -0.407874610778443 | -0.000406087824351297 | -0.000974610778443114 |
| 172 | 0.0531969230769231 | -0.257134810379242 | 0.0596949101796407 | 0.0555528143712575 |
| 173 | 0.0189840000000000 | 0.524340598802395 | 0.457660978043912 | -0.0433701796407186 |
| 174 | 0.00625846153846154 | -0.403813732534930 | 0.0272078842315369 | -0.0310251097804391 |
| 175 | -0.00292061538461538 | -0.304728303393214 | -1.46475878243513 | -0.0294007584830339 |

| | | | | |
|---|---|---|---|---|
| 176 | 0.00375507692307692 | 0.956905349301397 | 0.0828419161676647 | -0.100709780439122 |
| 177 | 0.0337956923076923 | 0.287835049900200 | 1.93866327345309 | -0.00990854291417166 |
| 178 | 0.0267027692307692 | -0.736318443113773 | -0.739485928143713 | 0.125887225548902 |
| 179 | 0.000417230769230769 | 0.0164059481037924 | -2.21155429141717 | -0.0300504990019960 |
| 180 | -0.0308750769230769 | 0.495752015968064 | 1.66414790419162 | -0.149440319361277 |
| 181 | -0.0289975384615385 | -0.537660279441118 | 2.05196177644711 | 0.0909636726546906 |
| 182 | -0.00312923076923077 | -0.543995249500998 | -2.04424610778443 | 0.183389261477046 |
| 183 | -0.00834461538461538 | 0.436788063872256 | -1.03024481037924 | -0.0979483832335329 |
| 184 | -0.0283716923076923 | 0.145541876247505 | 1.75389331337325 | -0.170881756487026 |
| 185 | -0.0630018461538462 | -0.273540758483034 | 0.363854690618762 | 0.0256647504990020 |
| 186 | -0.0613329230769231 | 0.440524071856287 | -1.13582764471058 | 0.0103958483033932 |
| 187 | -0.00208615384615385 | 0.231957365269461 | 0.408930439121757 | -0.109643712574850 |
| 188 | 0.0496504615384615 | -0.599548063872256 | 0.330149401197605 | -0.0474310578842315 |
| 189 | 0.0604984615384615 | 0.197033812375250 | -0.891768862275449 | 0.00763445109780439 |
| 190 | 0.0133513846153846 | 0.684014331337325 | 0.902327145708583 | -0.105257964071856 |
| 191 | -0.0319181538461538 | -0.385945868263473 | 0.706186726546906 | -0.0146191616766467 |
| 192 | -0.0421403076923077 | -0.430777964071856 | -1.54557025948104 | 0.146191616766467 |
| 193 | -0.0273286153846154 | 0.713739960079840 | -0.365479041916168 | 0.0332992015968064 |
| 194 | -0.000208615384615385 | 0.436950499001996 | 1.66820878243513 | -0.0261520558882236 |
| 195 | -0.0212787692307692 | -0.512970139720559 | 0.0397966067864271 | 0.0898266267465070 |
| 196 | -0.0659224615384615 | -0.0123450698602794 | -1.66252355289421 | 0.149765189620759 |
| 197 | -0.0947113846153846 | 0.398940678642715 | 0.177866467065868 | -0.0173805588822355 |
| 198 | -0.0811513846153846 | -0.158861556886228 | 1.47206836327345 | -0.0761820758483034 |
| 199 | -0.0310836923076923 | -0.162110259481038 | -0.0921819361277445 | 0.124587744510978 |
| 200 | 0.00709292307692308 | 0.0756947704590818 | -1.12405109780439 | 0.148628143712575 |
| 201 | 0.0273286153846154 | -0.262982475049900 | 0.329743313373253 | -0.0181927345309381 |
| 202 | 0.0168978461538462 | -0.139694211576846 | 0.935626347305389 | 0.0318372854291417 |
| 203 | 0.0127255384615385 | 0.210678363273453 | -0.315124151696607 | 0.167145748502994 |
| 204 | 0.0292061538461538 | -0.0360605988023952 | -0.278982335329341 | 0.0157562075848303 |
| 205 | 0.0465212307692308 | -0.156749900199601 | 0.321215469061876 | -0.0807302594810379 |
| 206 | 0.0565347692307692 | -0.0115328942115768 | 0.0369539920159681 | 0.00162435129740519 |
| 207 | 0.0239907692307692 | -0.00747201596806387 | -0.295631936127745 | -0.0268017964071856 |
| 208 | -0.0112652307692308 | -0.138882035928144 | 0.0422331337325349 | -0.137744990019960 |
| 209 | -0.0229476923076923 | 0.135958203592814 | 0.205886526946108 | -0.125562355289421 |
| 210 | -0.0158547692307692 | 0.318372854291417 | -0.362230339321357 | -0.00942123752495010 |
| 211 | 0.00980492307692308 | -0.159348862275449 | -0.121420259481038 | 0.00129948103792415 |
| 212 | 0.0156461538461538 | -0.156425029940120 | 0.284667564870260 | -0.0430453093812375 |
| 213 | 0.00208615384615385 | 0.213764630738523 | -0.0844662674650699 | -0.0362230339321357 |
| 214 | -0.00751015384615385 | -0.0484056686626747 | -0.216850898203593 | -0.0246901397205589 |
| 215 | 0.0223218461538462 | -0.181277604790419 | 0.180709081836327 | -0.0328118962075848 |
| 216 | 0.0748929230769231 | 0.188912055888224 | 0.214414371257485 | 0.0305378043912176 |
| 217 | 0.0838633846153846 | 0.0895017564870260 | -0.281824950099800 | 0.0294007584830339 |
| 218 | 0.0511107692307692 | -0.177054291417166 | -0.170556886227545 | 0.0204668263473054 |
| 219 | 0.00584123076923077 | 0.0726085029940120 | 0.228221357285429 | 0.143267784431138 |

| | | | | |
|---|---|---|---|---|
| 220 | -0.0112652307692308 | 0.155937724550898 | 0.0974610778443114 | 0.0683851896207585 |
| 221 | -0.00125169230769231 | -0.252424191616766 | -0.257865768463074 | -0.0802429540918164 |
| 222 | 0.0346301538461538 | -0.217175768463074 | -0.103552395209581 | 0.0755323353293413 |
| 223 | 0.0671741538461538 | 0.224810219560878 | 0.374412974051896 | 0.164221916167665 |
| 224 | 0.0588295384615385 | 0.00665984031936128 | 0.166089920159681 | -0.127511576846307 |
| 225 | 0.0440178461538462 | -0.468950219560878 | -0.340301596806387 | -0.149927624750499 |
| 226 | 0.0277458461538462 | -0.0164059481037924 | -0.108831536926148 | 0.132709500998004 |
| 227 | 0.0333784615384615 | 0.271429101796407 | 0.380910379241517 | 0.0488929740518962 |
| 228 | 0.0450609230769231 | -0.218475249500998 | 0.0795932135728543 | -0.180302994011976 |
| 229 | 0.0273286153846154 | -0.149115449101796 | -0.393905189620759 | -0.0813800000000000 |
| 230 | -0.000208615384615385 | 0.174942634730539 | -0.135227245508982 | 0.143755089820359 |
| 231 | -0.0179409230769231 | -0.00308626746506986 | 0.294819760479042 | -0.0274515369261477 |
| 232 | 0.00438092307692308 | -0.159998602794411 | -0.00730958083832335 | -0.259084031936128 |
| 233 | 0.0546572307692308 | 0.121663912175649 | -0.188018662674651 | 0.0181927345309381 |
| 234 | 0.0786480000000000 | 0.0886895808383234 | 0.0369539920159681 | 0.232607105788423 |
| 235 | 0.0655052307692308 | -0.0456442714570858 | 0.0848723552894212 | -0.0484056686626747 |
| 236 | 0.0421403076923077 | 0.222211257485030 | 0.118983732534930 | -0.0901514970059880 |
| 237 | 0.0327526153846154 | 0.162760000000000 | -0.0479183632734531 | 0.189724231536926 |
| 238 | 0.0475643076923077 | -0.175592375249501 | -0.0929941117764471 | 0.0795932135728543 |
| 239 | 0.0669655384615385 | 0.155937724550898 | -0.0215226546906188 | -0.0667608383233533 |
| 240 | 0.0715550769230769 | 0.449458003992016 | 0.121826347305389 | 0.0620502195608782 |
| 241 | 0.0486073846153846 | -0.205480439121756 | 0.0178678642714571 | 0.0484056686626747 |
| 242 | 0.0123083076923077 | -0.642430938123752 | -0.265175349301397 | -0.0521416766467066 |
| 243 | 0.0104307692307692 | -0.198820598802395 | -0.126293313373253 | 0.0672481437125748 |
| 244 | 0.0563261538461538 | 0.334129061876248 | 0.176648203592814 | 0.132222195608782 |
| 245 | 0.100761230769231 | 0.0964864670658683 | 0.110861976047904 | -0.0680603193612775 |
| 246 | 0.103473230769231 | -0.0173805588822355 | -0.193297804391218 | -0.123613133732535 |
| 247 | 0.0690516923076923 | 0.0690349301397206 | -0.0804053892215569 | 0.0700095409181637 |
| 248 | 0.0323353846153846 | 0.171531497005988 | 0.181115169660679 | 0.0433701796407186 |
| 249 | 0.0221132307692308 | 0.704156287425150 | 0.214820459081836 | -0.262007864271457 |
| 250 | 0.0454781538461538 | 0.145866746506986 | -0.180709081836327 | -0.145217005988024 |
| 251 | 0.0832375384615385 | -0.667283512974052 | -0.346799001996008 | 0.187287704590818 |
| 252 | 0.0876184615384615 | -0.115816247504990 | 0.140100299401198 | -0.00211165668662675 |
| 253 | 0.0559089230769231 | 0.559264151696607 | 0.211165668662675 | -0.150577365269461 |
| 254 | 0.0302492307692308 | 0.0360605988023952 | -0.237561377245509 | 0.146678922155689 |
| 255 | 0.0400541538461538 | -0.478371457085828 | -0.227409181636727 | 0.186800399201597 |
| 256 | 0.0740584615384615 | 0.209541317365269 | 0.208729141716567 | -0.0833292215568862 |
| 257 | 0.0972147692307692 | 0.487467824351297 | 0.152689021956088 | -0.0445072255489022 |
| 258 | 0.0974233846153846 | 0.109481277445110 | -0.247307485029940 | 0.134171417165669 |
| 259 | 0.0709292307692308 | -0.140019081836327 | -0.103958483033932 | 0.0432077445109780 |
| 260 | 0.0469384615384615 | -0.252424191616766 | 0.291977145708583 | -0.0765069461077844 |
| 261 | 0.0604984615384615 | -0.402839121756487 | 0.0824358283433134 | 0.0807302594810379 |
| 262 | 0.0765618461538462 | -0.376687065868263 | -0.163653393213573 | 0.154313373253493 |
| 263 | 0.0627932307692308 | 0.0513295009980040 | 0.0584766467065868 | -0.0456442714570858 |

| | | | | |
|---|---|---|---|---|
| 264 | 0.0260769230769231 | 0.0537660279441118 | 0.197764770459082 | -0.0302129341317365 |
| 265 | 0.00563261538461538 | 0.0380098203592814 | 0.0231470059880240 | 0.104933093812375 |
| 266 | 0.0189840000000000 | 0.379448463073852 | -0.101928043912176 | -0.0159186427145709 |
| 267 | 0.0450609230769231 | 0.345337085828343 | 0.0406087824351297 | -0.0898266267465070 |
| 268 | 0.0872012307692308 | 0.0355732934131737 | 0.140912475049900 | 0.0620502195608782 |
| 269 | 0.0830289230769231 | 0.0791059081836327 | 0.144973353293413 | 0.129948103792415 |
| 270 | 0.0473556923076923 | 0.109643712574850 | -0.0129948103792415 | 0.0441823552894212 |
| 271 | 0.0333784615384615 | -0.126699401197605 | -0.0869027944111777 | 0.0315124151696607 |
| 272 | 0.0319181538461538 | -0.165521397205589 | 0.0332992015968064 | 0.0391468662674651 |
| 273 | 0.0763532307692308 | 0.148953013972056 | 0.100709780439122 | -0.0539284630738523 |
| 274 | 0.100969846153846 | 0.254860718562874 | -0.0519792415169661 | -0.0523041117764471 |
| 275 | 0.0878270769230769 | -0.0875525349301397 | -0.211165668662675 | 0.0680603193612775 |
| 276 | 0.0680086153846154 | -0.00990854291417166 | -0.0312687624750499 | -0.0594512574850299 |
| 277 | 0.0542400000000000 | 0.0448320958083832 | 0.116547205588822 | -0.218312814371257 |
| 278 | 0.0863667692307692 | -0.135146027944112 | 0.00771566866267465 | 0.00503548902195609 |
| 279 | 0.108897230769231 | -0.193460239520958 | -0.0592888223552894 | 0.132547065868263 |
| 280 | 0.0957544615384615 | -0.311388143712575 | -0.0174617764471058 | -0.0547406387225549 |
| 281 | 0.0323353846153846 | -0.289296966067864 | 0.0503548902195609 | -0.145379441117764 |
| 282 | -0.0627932307692308 | -0.0557152495009980 | 0.157562075848303 | 0.0609131736526946 |
| 283 | -0.103890461538462 | 0.204505828343313 | -0.0783749500998004 | 0.134821157684631 |
| 284 | -0.0913735384615385 | 0.0384971257485030 | 0.109643712574850 | -0.175592375249501 |
| 285 | -0.0579950769230769 | -0.108019361277445 | -0.0755323353293413 | -0.210678363273453 |
| 286 | -0.0429747692307692 | 0.134333852295409 | 0.278982335329341 | 0.103796047904192 |
| 287 | -0.0417230769230769 | 0.208566706586826 | 0.639182235528942 | 0.133359241516966 |
| 288 | -0.0342129230769231 | 0.00958367265469062 | -0.501112375249501 | -0.121014171656687 |
| 289 | -0.0260769230769231 | 0.0633497005988024 | -0.883241017964072 | -0.168120359281437 |
| 290 | -0.00813600000000000 | 0.156912335329341 | 0.564462075848303 | 0.0891768862275449 |
| 291 | -0.0221132307692308 | -0.0781312974051896 | 1.40262734530938 | 0.181764910179641 |
| 292 | -0.0659224615384615 | -0.0198170858283433 | -1.47166227544910 | -0.0886895808383234 |
| 293 | -0.118284923076923 | 0.124587744510978 | -0.919788922155689 | -0.145866746506986 |
| 294 | -0.150203076923077 | -0.0237155289421158 | 1.59633123752495 | 0.170556886227545 |
| 295 | -0.123083076923077 | -0.187937445109780 | 0.350859880239521 | 0.211815409181637 |
| 296 | -0.0717636923076923 | -0.133359241516966 | -1.56506247504990 | -0.0696846706586826 |
| 297 | -0.0279544615384615 | -0.0204668263473054 | -0.390656487025948 | -0.119389820359281 |
| 298 | -0.0244080000000000 | -0.0981108183632735 | 1.77176117764471 | 0.0298880638722555 |
| 299 | -0.0392196923076923 | -0.0334616367265469 | -0.518168063872255 | -0.0371976447105788 |
| 300 | -0.0388024615384615 | -0.0123450698602794 | -0.676542315369262 | -0.0893393213572854 |
| 301 | -0.0298320000000000 | -0.121339041916168 | 0.571365568862275 | 0.0555528143712575 |
| 302 | -0.0146030769230769 | 0.0123450698602794 | 0.0483244510978044 | -0.00454818363273453 |
| 303 | -0.0342129230769231 | 0.100872215568862 | -0.201013473053892 | -0.145866746506986 |
| 304 | -0.0878270769230769 | -0.0714714570858283 | 0.289540618762475 | 0.0756947704590818 |
| 305 | -0.142901538461538 | -0.0701719760479042 | 0.257053592814371 | 0.290434011976048 |
| 306 | -0.160008000000000 | 0.238292335329341 | -1.02902654690619 | -0.0138069860279441 |
| 307 | -0.122457230769231 | 0.283124431137725 | 0.497863672654691 | -0.244302435129741 |

| | | | | |
|---|---|---|---|---|
| 308 | -0.0748929230769231 | 0.0459691417165669 | 0.875931437125749 | 0.0930753293413174 |
| 309 | -0.0440178461538462 | -0.0298880638722555 | -0.776033832335329 | 0.240241556886228 |
| 310 | -0.0488160000000000 | 0.0256647504990020 | -0.873900998003992 | -0.161135648702595 |
| 311 | -0.0665483076923077 | 0.115978682634731 | 0.795526047904192 | -0.218962554890220 |
| 312 | -0.0511107692307692 | 0.0368727744510978 | 1.00547345309381 | 0.191835888223553 |
| 313 | -0.0125169230769231 | -0.0789434730538922 | -1.19389820359281 | 0.192160758483034 |
| 314 | -0.00625846153846154 | -0.104120918163673 | -0.706186726546906 | -0.0729333732534930 |
| 315 | -0.0573692307692308 | 0.315936327345309 | 0.729333732534930 | -0.0714714570858283 |
| 316 | -0.114321230769231 | 0.432889620758483 | 0.484056686626747 | 0.0352484231536926 |
| 317 | -0.147491076923077 | -0.918895528942116 | -0.704968463073852 | 0.0256647504990020 |
| 318 | -0.142692923076923 | -1.10845732534930 | -0.510046307385230 | 0.0586390818363273 |
| 319 | -0.109314461538462 | 0.269155009980040 | 0.572989920159681 | 0.0927504590818363 |
| 320 | -0.0970061538461539 | 0.566248862275449 | 0.152282934131737 | -0.147815968063872 |
| 321 | -0.0905390769230769 | 0.0107207185628743 | -0.205886526946108 | -0.190373972055888 |
| 322 | -0.0811513846153846 | -0.118090339321357 | -0.0596949101796407 | 0.139206906187625 |
| 323 | -0.0565347692307692 | 0.112567544910180 | 0.216444810379242 | 0.0696846706586826 |
| 324 | 0.000834461538461538 | 0.438574850299401 | -0.0142130738522954 | -0.259571337325349 |
| 325 | 0.0302492307692308 | 0.542695768463074 | 0.113298502994012 | -0.0328118962075848 |
| 326 | 0.00792738461538461 | -0.631222914171657 | 0.172181237524950 | 0.240566427145709 |
| 327 | -0.0700947692307692 | -1.01408251497006 | -0.383346906187625 | -0.00324870259481038 |
| 328 | -0.138520615384615 | 0.701070019960080 | -0.0978671656686627 | -0.107369620758483 |
| 329 | -0.118076307692308 | 1.07580786427146 | 0.389032135728543 | 0.168445229540918 |
| 330 | -0.0563261538461538 | -0.356382674650699 | 0.212383932135729 | 0.208404271457086 |
| 331 | 0.0183581538461538 | -0.341113772455090 | -0.485274950099800 | -0.0196546506986028 |
| 332 | 0.0623760000000000 | 0.647791297405190 | -0.170556886227545 | 0.0196546506986028 |
| 333 | 0.0644621538461539 | 0.385620998003992 | 0.382940818363274 | 0.172506107784431 |
| 334 | 0.0709292307692308 | -0.230820319361277 | 0.107207185628743 | 0.0691973652694611 |
| 335 | 0.0755187692307692 | -0.309763792415170 | -0.287510179640719 | -0.0578269061876247 |
| 336 | 0.0872012307692308 | -0.468462914171657 | 0.0190861277445110 | 0.0898266267465070 |
| 337 | 0.0686344615384615 | -0.353946147704591 | 0.381722554890220 | 0.202231736526946 |
| 338 | 0.00396369230769231 | 0.385620998003992 | -0.195328243512974 | 0.0661110978043912 |
| 339 | -0.0559089230769231 | 0.151389540918164 | -0.183551696606786 | -0.0550655089820359 |
| 340 | -0.0951286153846154 | -0.524503033932136 | 0.168932534930140 | -0.0427204391217565 |
| 341 | -0.0884529230769231 | 0.112242674650699 | 0.182739520958084 | 0.0412585229540918 |
| 342 | -0.0381766153846154 | 0.571446786427146 | -0.223348303393214 | -0.0113704590818363 |
| 343 | 0.0148116923076923 | -0.172181237524950 | -0.106395009980040 | -0.142130738522954 |
| 344 | 0.0500676923076923 | -0.402514251497006 | 0.370352095808383 | -0.0687100598802395 |
| 345 | 0.0721809230769231 | 0.312850059880240 | 0.0739079840319361 | 0.0207916966067864 |
| 346 | 0.108688615384615 | 0.194434850299401 | -0.129948103792415 | -0.115491377245509 |
| 347 | 0.157087384615385 | -0.278576247504990 | -0.0365479041916168 | -0.157562075848303 |
| 348 | 0.184416000000000 | 0.174130459081836 | 0.235124850299401 | 0.0248525748502994 |
| 349 | 0.158964923076923 | 0.372463752495010 | 0.00730958083832335 | 0.0505173253493014 |
| 350 | 0.0882443076923077 | -0.0752074650698603 | -0.0726897205588822 | -0.101034650698603 |
| 351 | 0.0392196923076923 | -0.00844662674650699 | -0.0499488023952096 | -0.0823546107784431 |

| | | | | |
|---|---|---|---|---|
| 352 | 0.0302492307692308 | 0.354920758483034 | -0.159186427145709 | 0.107856926147705 |
| 353 | 0.0490246153846154 | 0.0207916966067864 | 0.174617764471058 | 0.0423955688622755 |
| 354 | 0.0757273846153846 | -0.244464870259481 | 0.183145608782435 | -0.157562075848303 |
| 355 | 0.0569520000000000 | -0.0711465868263473 | -0.0345174650698603 | 0.00909636726546906 |
| 356 | 0.0102221538461538 | -0.224322914171657 | -0.195328243512974 | 0.193135369261477 |
| 357 | -0.0137686153846154 | -0.155287984031936 | 0.101928043912176 | 0.00178678642714571 |
| 358 | 0.0264941538461538 | 0.290434011976048 | 0.168120359281437 | -0.157237205588822 |
| 359 | 0.0869926153846154 | 0.0781312974051896 | -0.0787810379241517 | 0.0256647504990020 |
| 360 | 0.0849064615384615 | -0.476422235528942 | -0.110455888223553 | 0.110455888223553 |
| 361 | 0.0394283076923077 | -0.0818673053892216 | -0.0633497005988024 | -0.0768318163672655 |
| 362 | -0.0262855384615385 | 0.396666586826347 | 0.194516067864271 | -0.0675730139720559 |
| 363 | -0.0486073846153846 | -0.0363854690618763 | -0.0142130738522954 | 0.0644867465069860 |
| 364 | -0.0160633846153846 | -0.257297245508982 | -0.278982335329341 | -0.00714714570858283 |
| 365 | 0.0319181538461538 | 0.0290758882235529 | 0.0203043912175649 | -0.0371976447105788 |
| 366 | 0.0767704615384615 | -0.0901514970059880 | 0.305378043912176 | 0.0740704191616766 |
| 367 | 0.0638363076923077 | -0.238292335329341 | -0.0812175648702595 | 0.0453194011976048 |
| 368 | 0.0319181538461538 | 0.138069860279441 | -0.221317864271457 | -0.0232282235528942 |
| 369 | 0.0114738461538462 | 0.109806147704591 | 0.273703193612774 | 0.0545782035928144 |
| 370 | 0.00208615384615385 | -0.124750179640719 | 0.127511576846307 | 0.0937250698602794 |
| 371 | 0.00125169230769231 | 0.147653532934132 | -0.284261477045908 | -0.0602634331337325 |
| 372 | -0.0433920000000000 | 0.329418443113772 | -0.0816236526946108 | -0.0882022754491018 |
| 373 | -0.0963803076923077 | -0.0290758882235529 | 0.203856087824351 | 0.112567544910180 |
| 374 | -0.113695384615385 | -0.240079121756487 | -0.106395009980040 | 0.0852784431137725 |
| 375 | -0.0830289230769231 | 0.116628423153693 | -0.147003792415170 | -0.140506387225549 |
| 376 | -0.00542400000000000 | 0.0588015169660679 | 0.0345174650698603 | -0.0797556487025948 |
| 377 | 0.0594553846153846 | -0.217825508982036 | -0.0312687624750499 | 0.0979483832335329 |
| 378 | 0.0890787692307692 | 0.0328118962075848 | 0.0523853293413174 | -0.0472686227544910 |
| 379 | 0.0872012307692308 | 0.184039001996008 | 0.136851596806387 | -0.122638522954092 |
| 380 | 0.0719723076923077 | -0.0341113772455090 | -0.0730958083832335 | 0.0753699001996008 |
| 381 | 0.0813600000000000 | -0.0735831137724551 | -0.242434431137725 | 0.0349235528942116 |
| 382 | 0.0761446153846154 | 0.240566427145709 | 0.238373552894212 | -0.153501197604790 |
| 383 | 0.0377593846153846 | 0.189561796407186 | 0.178678642714571 | -0.0248525748502994 |
| 384 | -0.0183581538461538 | -0.240728862275449 | -0.277357984031936 | 0.123775568862275 |
| 385 | -0.0688430769230769 | -0.288322355289421 | -0.264769261477046 | -0.0487305389221557 |
| 386 | -0.0627932307692308 | -0.0523041117764471 | 0.157155988023952 | -0.138394730538922 |
| 387 | -0.0187753846153846 | 0.0743952894211577 | 0.209947405189621 | 0.0589639520958084 |
| 388 | 0.0323353846153846 | 0.0946996806387226 | -0.193297804391218 | 0.0984356886227545 |
| 389 | 0.0652966153846154 | 0.0756947704590818 | -0.0726897205588822 | -0.119714690618762 |
| 390 | 0.0725981538461538 | 0.174942634730539 | 0.172181237524950 | -0.109643712574850 |
| 391 | 0.0557003076923077 | 0.240241556886228 | 0.188424750499002 | 0.103796047904192 |
| 392 | 0.0425575384615385 | -0.147491097804391 | -0.112486327345309 | 0.0446696606786427 |
| 393 | 0.0413058461538462 | -0.384808822355289 | -0.0312687624750499 | -0.0683851896207585 |
| 394 | 0.0175236923076923 | -0.114354331337325 | 0.0268017964071856 | 0.118252774451098 |
| 395 | -0.0244080000000000 | 0.185825788423154 | -0.0901514970059880 | 0.124587744510978 |

| | | | | |
|---|---|---|---|---|
| 396 | -0.0824030769230769 | 0.259246467065868 | -0.0913697604790419 | -0.111430499001996 |
| 397 | -0.109105846153846 | 0.0701719760479042 | 0.139694211576846 | 0.0560401197604790 |
| 398 | -0.0784393846153846 | -0.0430453093812375 | -0.102740219560878 | 0.239591816367265 |
| 399 | -0.0102221538461538 | 0.0630248303393214 | 0.194109980039920 | -0.0584766467065868 |
| 400 | 0.0557003076923077 | -0.0151064670658683 | 0.548218562874252 | -0.176729421157685 |
| 401 | 0.0763532307692308 | -0.408199481037924 | -0.673699700598802 | 0.171369061876248 |
| 402 | 0.0776049230769231 | -0.362717644710579 | -1.01197085828343 | 0.255023153692615 |
| 403 | 0.0955458461538462 | 0.259733772455090 | 0.740298103792415 | -0.0946996806387226 |
| 404 | 0.119536615384615 | 0.440036766467066 | 1.48912405189621 | -0.0701719760479042 |
| 405 | 0.107645538461538 | 0.0256647504990020 | -1.65440179640719 | 0.262495169660679 |
| 406 | 0.0448523076923077 | -0.0607507385229541 | -1.00750389221557 | 0.138719600798403 |
| 407 | -0.0456867692307692 | 0.228058922155689 | 1.94881546906188 | -0.127511576846307 |
| 408 | -0.113486769230769 | 0.193622674650699 | 0.618065668662675 | 0.0722836327345309 |
| 409 | -0.121622769230769 | -0.0363854690618763 | -1.71044191616766 | 0.153663632734531 |
| 410 | -0.0751015384615385 | -0.153501197604790 | -0.565274251497006 | -0.136445508982036 |
| 411 | -0.0104307692307692 | -0.199957644710579 | 1.75917245508982 | -0.115004071856287 |
| 412 | 0.0244080000000000 | 0.129298363273453 | -0.0617253493013972 | 0.0820297405189621 |
| 413 | 0.0210701538461538 | 0.496889061876248 | -0.428422654690619 | -0.0300504990019960 |
| 414 | 0.0219046153846154 | 0.00877149700598802 | 0.196952594810379 | -0.189074491017964 |
| 415 | 0.0446436923076923 | -0.520929461077844 | -0.200201297405190 | -0.0204668263473054 |
| 416 | 0.0604984615384615 | -0.0207916966067864 | 0.155531636726547 | 0.0901514970059880 |
| 417 | 0.0415144615384615 | 0.288647225548902 | 0.622532634730539 | -0.108181796407186 |
| 418 | -0.0168978461538462 | -0.207754530938124 | -0.162435129740519 | -0.135795768463074 |
| 419 | -0.0644621538461539 | -0.325844870259481 | -1.42293173652695 | 0.0691973652694611 |
| 420 | -0.0642535384615385 | 0.0719587624750499 | 0.503142814371258 | 0.0704968463073852 |
| 421 | -0.0289975384615385 | 0.133684111776447 | 1.27511576846307 | -0.0802429540918164 |
| 422 | 0.0375507692307692 | -0.0503548902195609 | -0.535223752495010 | -0.0142942914171657 |
| 423 | 0.0938769230769231 | 0.105095528942116 | -1.13826417165669 | 0.139369341317365 |
| 424 | 0.102221538461538 | -0.0419082634730539 | 0.517761976047904 | 0.0847911377245509 |
| 425 | 0.0930424615384616 | -0.366128782435130 | 1.27917664670659 | -0.0615629141716567 |
| 426 | 0.0869926153846154 | 0.0272891017964072 | -0.608319560878244 | 0.0300504990019960 |
| 427 | 0.0901218461538462 | 0.376199760479042 | -0.962428143712575 | 0.136283073852295 |
| 428 | 0.0788566153846154 | 0.0510046307385230 | 0.327712874251497 | -0.00519792415169661 |
| 429 | -0.00250338461538462 | -0.103958483033932 | 0.670450998003992 | -0.0462940119760479 |
| 430 | -0.0909563076923077 | 0.176566986027944 | -0.273297105788423 | 0.0480807984031936 |
| 431 | -0.120579692307692 | 0.204830698602794 | -0.458879241516966 | 0.0717963273453094 |
| 432 | -0.0913735384615385 | 0.0518168063872256 | 0.407306087824351 | -0.00194922155688623 |
| 433 | -0.00542400000000000 | -0.0659486626746507 | 0.176648203592814 | -0.0534411576846307 |
| 434 | 0.0600812307692308 | -0.0870652295409182 | -0.194516067864271 | 0.00633497005988024 |
| 435 | 0.0892873846153846 | -0.0695222355289421 | 0.117765469061876 | 0.0188424750499002 |
| 436 | 0.103473230769231 | -0.0669232734530938 | 0.257053592814371 | -0.0594512574850299 |
| 437 | 0.110983384615385 | 0.0726085029940120 | -0.158374251497006 | -0.0573396007984032 |
| 438 | 0.135182769230769 | 0.0912885429141717 | -0.0657862275449102 | -0.0147815968063872 |
| 439 | 0.144779076923077 | -0.0345986826347305 | 0.281824950099800 | -0.00552279441117765 |

| | | | | |
|---|---|---|---|---|
| 440 | 0.113904000000000 | -0.0529538522954092 | -0.114516766467066 | -0.0217663073852295 |
| 441 | 0.0317095384615385 | -0.0433701796407186 | -0.134821157684631 | -0.126699401197605 |
| 442 | -0.0486073846153846 | -0.139369341317365 | 0.233906586826347 | -0.0555528143712575 |
| 443 | -0.0748929230769231 | -0.109318842315369 | 0.181927345309381 | 0.0791059081836327 |
| 444 | -0.0325440000000000 | 0.0289134530938124 | -0.261114471057884 | -0.00795932135728543 |
| 445 | 0.0308750769230769 | 0.0745577245508982 | -0.108019361277445 | -0.0142942914171657 |
| 446 | 0.0498590769230769 | 0.140019081836327 | 0.322839820359281 | 0.108181796407186 |
| 447 | 0.0438092307692308 | 0.204343393213573 | 0.0702531936127745 | 0.0258271856287425 |
| 448 | 0.0152289230769231 | 0.110780758483034 | -0.363854690618762 | -0.117115728542914 |
| 449 | 0.00667569230769231 | 0.00536035928143713 | -0.0345174650698603 | 0.0378473852295409 |
| 450 | 0.0164806153846154 | -0.0500300199600798 | 0.361824251497006 | 0.112080239520958 |
| 451 | 0.00479815384615385 | -0.197521117764471 | -0.203450000000000 | -0.111917804391218 |
| 452 | -0.0469384615384615 | -0.0831667864271457 | -0.551873353293413 | -0.224160479041916 |
| 453 | -0.129550153846154 | 0.0651364870259481 | -0.147815968063872 | 0.0549030738522954 |
| 454 | -0.166892307692308 | -0.0713090219560878 | 0.340707684630739 | 0.174455329341317 |
| 455 | -0.141232615384615 | -0.0138069860279441 | -0.164465568862275 | -0.166333572854291 |
| 456 | -0.0894960000000000 | 0.0912885429141717 | -0.510452395209581 | -0.140343952095808 |
| 457 | -0.0446436923076923 | -0.0204668263473054 | 0.148222055888224 | 0.177054291417166 |
| 458 | -0.0271200000000000 | -0.0794307784431138 | 0.368321656686627 | 0.103146307385230 |
| 459 | -0.0312923076923077 | -0.0919382834331337 | -0.0929941117764471 | -0.134983592814371 |
| 460 | -0.0152289230769231 | -0.156912335329341 | -0.316748502994012 | -0.0420706986027944 |
| 461 | 0.000417230769230769 | -0.216201157684631 | 0.200607385229541 | 0.0615629141716567 |
| 462 | 0.000834461538461538 | -0.0264769261477046 | 0.136851596806387 | -0.0373600798403194 |
| 463 | -0.0352560000000000 | 0.211815409181637 | -0.138069860279441 | -0.00877149700598802 |
| 464 | -0.117450461538462 | 0.0160810778443114 | -0.0475122754491018 | 0.0401214770459082 |
| 465 | -0.167935384615385 | -0.160485908183633 | 0.0828419161676647 | 0.0206292614770459 |
| 466 | -0.174819692307692 | -0.122963393213573 | 0.156343812375250 | 0.0471061876247505 |
| 467 | -0.125795076923077 | 0.322433732534930 | 0.136039421157685 | 0.186637964071856 |
| 468 | -0.0565347692307692 | 0.674593093812375 | 0.162029041916168 | 0.137744990019960 |
| 469 | -0.0154375384615385 | 0.300992295409182 | -0.118983732534930 | -0.0271266666666667 |
| 470 | -0.00125169230769231 | 0.00194922155688623 | -0.0235530938123753 | 0.0839789620758483 |
| 471 | -0.00166892307692308 | -0.148140838323353 | 0.185582135728543 | 0.192648063872255 |
| 472 | 0.0171064615384615 | -0.104770658682635 | 0.0730958083832335 | -0.0469437524950100 |
| 473 | 0.0392196923076923 | 0.234718762475050 | -0.248525748502994 | -0.185013612774451 |
| 474 | 0.0388024615384615 | 0.0139694211576846 | -0.105582834331337 | 0.0976235129740519 |
| 475 | -0.00292061538461538 | -0.837353093812375 | 0.328118962075848 | 0.0410960878243513 |
| 476 | -0.0554916923076923 | -0.381072814371258 | 0.0186800399201597 | -0.243977564870259 |
| 477 | -0.0692603076923077 | 0.913210299401198 | -0.348829441117764 | -0.0394717365269461 |
| 478 | -0.0423489230769231 | 0.223998043912176 | 0.0324870259481038 | 0.236343113772455 |
| 479 | 0.00125169230769231 | -0.805190938123753 | 0.433701796407186 | -0.00519792415169661 |
| 480 | 0.0223218461538462 | 0.0321621556886228 | -0.166089920159681 | -0.184688742514970 |
| 481 | 0.0148116923076923 | 0.503061596806387 | -0.255835329341317 | 0.131897325349301 |
| 482 | -0.000834461538461538 | -0.239429381237525 | 0.292789321357285 | 0.161135648702595 |
| 483 | 0.00751015384615385 | -0.327794091816367 | 0.198983033932136 | -0.217987944111776 |

| | | | | |
|---|---|---|---|---|
| 484 | 0.0383852307692308 | 0.0638370059880239 | -0.293601497005988 | -0.224160479041916 |
| 485 | 0.0423489230769231 | -0.114354331337325 | -0.278170159680639 | 0.0836540918163673 |
| 486 | 0.0175236923076923 | 0.0352484231536926 | 0.232282235528942 | -0.00633497005988024 |
| 487 | -0.0275372307692308 | 0.662410459081836 | 0.0613192614770459 | -0.141480998003992 |
| 488 | -0.0486073846153846 | 0.252911497005988 | -0.231063972055888 | 0.0597761277445110 |
| 489 | -0.0292061538461538 | -0.244464870259481 | -0.0938062874251497 | 0.117440598802395 |
| 490 | 0.0123083076923077 | 0.256160199600798 | 0.168526447105788 | -0.0524665469061876 |
| 491 | 0.0690516923076923 | 0.291895928143713 | 0.155531636726547 | 0.0154313373253493 |
| 492 | 0.0844892307692308 | -0.250312534930140 | -0.0832480039920160 | 0.206455049900200 |
| 493 | 0.0659224615384615 | -0.194109980039920 | -0.0714714570858283 | -0.0183551696606786 |
| 494 | 0.0440178461538462 | 0.178678642714571 | 0.0479183632734531 | -0.138557165668663 |
| 495 | 0.0423489230769231 | -0.00162435129740519 | 0.223348303393214 | 0.107207185628743 |
| 496 | 0.0673827692307692 | -0.261682994011976 | 0.108831536926148 | 0.122638522954092 |
| 497 | 0.0596640000000000 | 0.0381722554890220 | -0.0670044910179641 | -0.152039281437126 |
| 498 | 0.0173150769230769 | 0.161947824351297 | -0.0211165668662675 | -0.0885271457085828 |
| 499 | -0.0196098461538462 | -0.199632774451098 | 0.184769960079840 | 0.241703473053892 |
| 500 | -0.0221132307692308 | -0.196708942115768 | 0.335022455089820 | 0.0883647105788423 |
| 501 | 0.0168978461538462 | -0.00730958083832335 | -0.0365479041916168 | -0.173480718562874 |
| 502 | 0.0630018461538462 | -0.0173805588822355 | -0.122638522954092 | 0.0310251097804391 |
| 503 | 0.0997181538461539 | 0.0882022754491018 | 0.127917664670659 | 0.257622115768463 |
| 504 | 0.111609230769231 | 0.125237485029940 | 0.123450698602794 | -0.00958367265469062 |
| 505 | 0.0945027692307692 | 0.0831667864271457 | -0.109237624750499 | -0.196546506986028 |
| 506 | 0.0888701538461539 | 0.162597564870259 | -0.260302295409182 | 0.0466188822355289 |
| 507 | 0.110357538461538 | 0.123450698602794 | -0.117359381237525 | 0.179978123752495 |
| 508 | 0.119328000000000 | -0.0594512574850299 | -0.0909636726546906 | -0.0341113772455090 |
| 509 | 0.0853236923076923 | -0.0971362075848303 | -0.0633497005988024 | -0.202394171656687 |
| 510 | 0.0306664615384615 | 0.0266393612774451 | -0.0162435129740519 | 0.00568522954091816 |
| 511 | 0.00458953846153846 | 0.124425309381238 | -0.0755323353293413 | 0.122476087824351 |
| 512 | 0.0350473846153846 | 0.0779688622754491 | 0.0450757485029940 | -0.0307002395209581 |
| 513 | 0.0897046153846154 | -0.0519792415169661 | 0.556746407185629 | -0.101197085828343 |
| 514 | 0.113278153846154 | -0.138069860279441 | -0.388219960079840 | 0.123288263473054 |
| 515 | 0.104516307692308 | -0.128323752495010 | -1.34739940119760 | 0.157074770459082 |
| 516 | 0.0759360000000000 | -0.0287510179640719 | 1.03836656686627 | -0.111268063872255 |
| 517 | 0.0596640000000000 | -0.0363854690618763 | 1.41115518962076 | -0.0703344111776447 |
| 518 | 0.0913735384615385 | -0.175105069860279 | -1.95856157684631 | 0.164059481037924 |
| 519 | 0.129758769230769 | -0.0843038323353293 | -0.981108183632735 | 0.0831667864271457 |
| 520 | 0.133305230769231 | 0.00617253493013972 | 2.82434081836327 | -0.242353213572854 |
| 521 | 0.0974233846153846 | -0.0242028343313373 | 0.126293313373253 | -0.0635121357285429 |
| 522 | 0.0584123076923077 | 0.128486187624751 | -2.85195479041916 | 0.292708103792415 |
| 523 | 0.0567433846153846 | 0.118252774451098 | 0.779688622754491 | 0.00860906187624751 |
| 524 | 0.0882443076923077 | -0.0571771656686627 | 2.10556536926148 | -0.291895928143713 |
| 525 | 0.118910769230769 | -0.0804053892215569 | -1.17927904191617 | 0.0185176047904192 |
| 526 | 0.121622769230769 | -0.0281012774451098 | -1.28892275449102 | 0.140019081836327 |
| 527 | 0.108271384615385 | 0.0175429940119760 | 1.38191686626747 | -0.197033812375250 |

| | | | | |
|---|---|---|---|---|
| 528 | 0.0970061538461539 | 0.0295631936127745 | 0.0418270459081836 | -0.116953293413174 |
| 529 | 0.0855323076923077 | -0.0877149700598802 | -1.07207185628743 | 0.105420399201597 |
| 530 | 0.0838633846153846 | -0.0311875449101796 | 1.05095528942116 | -0.0812175648702595 |
| 531 | 0.0844892307692308 | 0.281824950099800 | 0.186394311377246 | -0.156100159680639 |
| 532 | 0.0538227692307692 | 0.110131017964072 | -1.31044540918164 | 0.204505828343313 |
| 533 | -0.00667569230769231 | -0.300017684630739 | 0.789028642714571 | 0.246901397205589 |
| 534 | -0.0379680000000000 | -0.0917758483033932 | 1.64506177644711 | -0.201257125748503 |
| 535 | -0.00250338461538462 | 0.180465429141717 | -1.18171556886228 | -0.112567544910180 |
| 536 | 0.0509021538461538 | -0.0461315768463074 | -1.19958343313373 | 0.369215049900200 |
| 537 | 0.0711378461538462 | -0.119552255489022 | 1.73358892215569 | 0.157074770459082 |
| 538 | 0.0575778461538462 | -0.0639994411177645 | 0.924255888223553 | -0.325844870259481 |
| 539 | 0.0452695384615385 | -0.139206906187625 | -1.70556886227545 | 0.0235530938123753 |
| 540 | 0.0481901538461539 | -0.293682714570858 | -0.763851197604790 | 0.382047425149701 |
| 541 | 0.0738498461538462 | -0.315286586826347 | 1.42861696606786 | -0.0843038323353293 |
| 542 | 0.0951286153846154 | 0.450270179640719 | 0.246901397205589 | -0.299043073852295 |
| 543 | 0.0615415384615385 | 0.903626626746507 | -1.21988782435130 | 0.130922714570858 |
| 544 | -0.00292061538461538 | 0.439062155688623 | 0.0507609780439122 | 0.154963113772455 |
| 545 | -0.0515280000000000 | 0.141643433133733 | 0.762226846307385 | -0.267530658682635 |
| 546 | -0.0590381538461538 | -0.195571896207585 | -0.154719461077844 | -0.121663912175649 |
| 547 | -0.0283716923076923 | -0.587852734530938 | -0.514513273453094 | 0.226272135728543 |
| 548 | 0.00146030769230769 | -0.0690349301397206 | 0.256647504990020 | -0.107694491017964 |
| 549 | -0.00897046153846154 | 0.318697724550898 | 0.145785528942116 | -0.369864790419162 |
| 550 | -0.0527796923076923 | -0.821596886227545 | -0.355326846307385 | 0.0891768862275449 |
| 551 | -0.0851150769230769 | -0.483731816367266 | 0.0714714570858283 | 0.200932255489022 |
| 552 | -0.0855323076923077 | 1.39742942115768 | 0.0873088822355290 | -0.277926506986028 |
| 553 | -0.0640449230769231 | 0.436300758483034 | -0.162435129740519 | -0.191023712574850 |
| 554 | -0.0590381538461538 | -1.16108630738523 | 0.0869027944111777 | 0.251287145708583 |
| 555 | -0.0897046153846154 | -0.0112080239520958 | 0.362636427145709 | 0.0227409181636727 |
| 556 | -0.105350769230769 | 0.799343273453094 | 0.0162435129740519 | -0.230657884231537 |
| 557 | -0.104099076923077 | -0.600035369261477 | -0.208729141716567 | 0.189074491017964 |
| 558 | -0.0757273846153846 | -0.846124590818363 | 0.0605070858283433 | 0.300829860279441 |
| 559 | -0.0150203076923077 | 0.388382395209581 | 0.220099600798403 | -0.118902514970060 |
| 560 | 0.0143944615384615 | 0.142455608782435 | -0.194922155688623 | -0.124912614770459 |
| 561 | 0.0114738461538462 | -0.214251936127745 | -0.340707684630739 | 0.243165389221557 |
| 562 | -0.0141858461538462 | 0.635771097804391 | 0.265987524950100 | 0.0747201596806387 |
| 563 | -0.000208615384615385 | 0.293032974051896 | 0.393499101796407 | -0.241053732534930 |
| 564 | 0.0477729230769231 | -0.593213093812375 | -0.220911776447106 | 0.0644867465069860 |
| 565 | 0.0448523076923077 | 0.383184471057884 | -0.380504291417166 | 0.275165109780439 |
| 566 | -0.00125169230769231 | 0.755323353293413 | 0.451163572854291 | 0.00194922155688623 |
| 567 | -0.0713464615384615 | -0.576969580838323 | 0.452381836327345 | -0.0882022754491018 |
| 568 | -0.111817846153846 | -0.574045748502994 | -0.333398103792415 | 0.123613133732535 |
| 569 | -0.0959630769230769 | 0.572258962075848 | -0.321621556886228 | 0.0765069461077844 |
| 570 | -0.0531969230769231 | 0.359793812375250 | 0.404869560878244 | -0.0190049101796407 |
| 571 | -0.0146030769230769 | -0.437275369261477 | 0.287104091816367 | 0.108669101796407 |

| | | | | |
|---|---|---|---|---|
| 572 | -0.00667569230769231 | -0.00341113772455090 | -0.434107884231537 | 0.0138069860279441 |
| 573 | -0.0187753846153846 | 0.325844870259481 | -0.188018662674651 | -0.148628143712575 |
| 574 | -0.0139772307692308 | -0.0956742914171657 | 0.121014171656687 | 0.0428828742514970 |
| 575 | 0.0166892307692308 | -0.00422331337325349 | 0.0938062874251497 | 0.189399361277445 |
| 576 | 0.0465212307692308 | 0.0906388023952096 | -0.0349235528942116 | -0.0324870259481038 |
| 577 | 0.0492332307692308 | -0.263307345309381 | -0.147815968063872 | -0.121826347305389 |
| 578 | 0.00521538461538462 | -0.141805868263473 | -0.0804053892215569 | 0.108506666666667 |
| 579 | -0.0515280000000000 | 0.149927624750499 | 0.166089920159681 | 0.109643712574850 |
| 580 | -0.0600812307692308 | -0.0279388423153693 | 0.277764071856287 | -0.126536966067864 |
| 581 | -0.0327526153846154 | 0.0501924550898204 | -0.215226546906188 | -0.0612380439121757 |
| 582 | -0.00855323076923077 | 0.246738962075848 | -0.335022455089820 | 0.0674105788423154 |
| 583 | -0.0120996923076923 | -0.0985981237524950 | 0.105988922155689 | -0.108669101796407 |
| 584 | -0.0509021538461538 | -0.178353772455090 | 0.165277744510978 | -0.102009261477046 |
| 585 | -0.0863667692307692 | 0.362392774451098 | -0.151470758483034 | 0.0885271457085828 |
| 586 | -0.104724923076923 | 0.295307065868264 | -0.201825648702595 | -0.0552279441117765 |
| 587 | -0.0990923076923077 | -0.319672335329341 | 0.159998602794411 | -0.185176047904192 |
| 588 | -0.0890787692307692 | -0.195084590818363 | 0.283449301397206 | 0.0255023153692615 |
| 589 | -0.126420923076923 | 0.177704031936128 | -0.0531975049900200 | 0.122151217564870 |
| 590 | -0.171690461538462 | -0.0475934930139721 | -0.265581437125749 | -0.0445072255489022 |
| 591 | -0.171899076923077 | -0.147978403193613 | 0.0864967065868263 | -0.0565274251497006 |
| 592 | -0.116824615384615 | 0.177541596806387 | 0.207916966067864 | 0.0946996806387226 |
| 593 | -0.0454781538461538 | 0.0199795209580838 | -0.224566566866267 | 0.0948621157684631 |
| 594 | -0.00166892307692308 | -0.208241836327345 | -0.223348303393214 | 0.0188424750499002 |
| 595 | 0.0216960000000000 | 0.123775568862275 | 0.178272554890220 | 0.0492178443113772 |
| 596 | 0.0214873846153846 | 0.0802429540918164 | 0.226597005988024 | 0.00860906187624751 |
| 597 | 0.0210701538461538 | -0.435163712574850 | -0.0913697604790419 | -0.0968113373253493 |
| 598 | 0.0352560000000000 | -0.190698842315369 | -0.109643712574850 | 0.0929128942115768 |
| 599 | 0.0442264615384615 | 0.492340878243513 | 0.214008283433134 | 0.203043912175649 |
| 600 | 0.00542400000000000 | 0.00211165668662675 | 0.222130039920160 | -0.102171696606786 |
| 601 | -0.0892873846153846 | -0.458554371257485 | -0.0580705588822355 | -0.196221636726547 |
| 602 | -0.171273230769231 | 0.185176047904192 | -0.157562075848303 | 0.204993133732535 |
| 603 | -0.197558769230769 | 0.277276766467066 | 0.0198983033932136 | 0.219124990019960 |
| 604 | -0.170021538461538 | -0.195084590818363 | 0.147003792415170 | -0.320159640718563 |
| 605 | -0.123708923076923 | -0.0979483832335329 | -0.0962428143712575 | -0.221074211576846 |
| 606 | -0.0965889230769231 | 0.244627305389222 | -0.315530239520958 | 0.304240998003992 |
| 607 | -0.0846978461538462 | 0.101846826347305 | -0.0174617764471058 | 0.148953013972056 |
| 608 | -0.0604984615384615 | -0.160485908183633 | 0.273297105788423 | -0.304403433133733 |
| 609 | -0.00792738461538461 | 0.131572455089820 | 0.0304565868263473 | -0.117603033932136 |
| 610 | 0.0362990769230769 | 0.216363592814371 | -0.203450000000000 | 0.229845708582834 |
| 611 | 0.0442264615384615 | -0.112729980039920 | -0.0576644710578842 | -0.00324870259481038 |
| 612 | 0.0302492307692308 | -0.0149440319361277 | 0.190049101796407 | -0.229033532934132 |
| 613 | 0.00521538461538462 | 0.0181927345309381 | 0.115328942115768 | -0.0112080239520958 |
| 614 | 0.00458953846153846 | -0.243490259481038 | -0.257865768463074 | 0.104933093812375 |
| 615 | 0.0417230769230769 | -0.214901676646707 | -0.119795908183633 | -0.0972986427145709 |

| | | | | |
|---|---|---|---|---|
| 616 | 0.0801083076923077 | 0.0246901397205589 | 0.117765469061876 | -0.0500300199600798 |
| 617 | 0.0913735384615385 | -0.0342738123752495 | -0.00243652694610778 | 0.0903139321357285 |
| 618 | 0.0634190769230769 | 0.0753699001996008 | -0.117765469061876 | -0.0555528143712575 |
| 619 | 0.0279544615384615 | 0.237480159680639 | -0.118577644710579 | -0.0612380439121757 |
| 620 | 0.0171064615384615 | -0.192810499001996 | 0.124262874251497 | 0.138882035928144 |
| 621 | 0.0250338461538462 | -0.212465149700599 | 0.0150252495009980 | 0.0479183632734531 |
| 622 | 0.0271200000000000 | 0.0485681037924152 | 0.00324870259481038 | -0.196059201596806 |
| 623 | -0.00688430769230769 | -0.0423955688622755 | -0.132790718562874 | -0.00308626746506986 |
| 624 | -0.0467298461538462 | -0.0998976047904192 | 0.106395009980040 | 0.140343952095808 |
| 625 | -0.0650880000000000 | 0.175917245508982 | 0.602228243512974 | -0.118577644710579 |
| 626 | -0.0444350769230769 | 0.445884431137725 | -0.495021057884232 | -0.0948621157684631 |
| 627 | 0.0221132307692308 | 0.287510179640719 | -1.06760489021956 | 0.240079121756487 |
| 628 | 0.0725981538461538 | 0.111592934131737 | 0.733394610778443 | 0.128486187624751 |
| 629 | 0.0963803076923077 | -0.151227105788423 | 1.34496287425150 | -0.204180958083832 |
| 630 | 0.105768000000000 | -0.217500638722555 | -1.47612924151697 | 0.0617253493013972 |
| 631 | 0.111817846153846 | -0.0190049101796407 | -0.830043512974052 | 0.309276487025948 |
| 632 | 0.141441230769231 | 0.0998976047904192 | 2.22617345309381 | -0.0558776846307385 |
| 633 | 0.159382153846154 | 0.0220911776447106 | 0.369946007984032 | -0.218800119760479 |
| 634 | 0.143944615384615 | -0.116303552894212 | -1.65521397205589 | 0.243490259481038 |
| 635 | 0.0876184615384615 | 0.145704311377246 | 0.262738822355289 | 0.265256566866268 |
| 636 | 0.0225304615384615 | 0.108831536926148 | 1.34090199600798 | -0.294332455089820 |
| 637 | 0.00584123076923077 | -0.109643712574850 | -0.617253493013972 | -0.221236646706587 |
| 638 | 0.0150203076923077 | -0.349722834331337 | -0.652989221556886 | 0.202394171656687 |
| 639 | 0.0248252307692308 | -0.518655369261477 | 0.662329241516966 | 0.0414209580838323 |
| 640 | 0.0302492307692308 | -0.0207916966067864 | -0.393499101796407 | -0.246414091816367 |
| 641 | 0.0354646153846154 | 0.593537964071856 | -0.488929740518962 | -0.00958367265469062 |
| 642 | 0.0385938461538462 | 0.249987664670659 | 0.829637425149701 | 0.190536407185629 |
| 643 | 0.0613329230769231 | -0.136445508982036 | -0.000812175648702595 | -0.0279388423153693 |
| 644 | 0.102221538461538 | 0.307652135728543 | -1.05826487025948 | -0.125887225548902 |
| 645 | 0.109940307692308 | 0.337702634730539 | 0.338271157684631 | 0.138394730538922 |
| 646 | 0.0723895384615385 | 0.0760196407185629 | 1.14435548902196 | 0.112567544910180 |
| 647 | 0.0254510769230769 | -0.221074211576846 | -0.479995808383234 | -0.163897045908184 |
| 648 | 0.0150203076923077 | -0.142455608782435 | -0.953088123752495 | 0.0558776846307385 |
| 649 | 0.0592467692307692 | -0.0250150099800399 | 0.516543712574850 | 0.217500638722555 |
| 650 | 0.130801846153846 | 0.0149440319361277 | 0.959179441117764 | -0.0550655089820359 |
| 651 | 0.171690461538462 | 0.369539920159681 | -0.488117564870260 | -0.103471177644711 |
| 652 | 0.173359384615385 | 0.145541876247505 | -0.827200898203593 | 0.147003792415170 |
| 653 | 0.149994461538462 | -0.249662794411178 | 0.589639520958084 | 0.100222475049900 |
| 654 | 0.120162461538462 | -0.117115728542914 | 0.513295009980040 | -0.117927904191617 |
| 655 | 0.105976615384615 | 0.139369341317365 | -0.373600798403194 | -0.0695222355289421 |
| 656 | 0.0832375384615385 | -0.298718203592814 | -0.346392914171657 | 0.0410960878243513 |
| 657 | 0.0413058461538462 | -0.435488582834331 | 0.306190219560878 | 0.0373600798403194 |
| 658 | -0.0164806153846154 | 0.00665984031936128 | 0.116547205588822 | -0.0664359680638723 |
| 659 | -0.0644621538461539 | 0.0605883033932136 | -0.406087824351297 | -0.0550655089820359 |

| | | | | |
|---|---|---|---|---|
| 660 | -0.0590381538461538 | 0.0753699001996008 | 0.0678166666666667 | 0.0120201996007984 |
| 661 | -0.00938769230769231 | 0.266393612774451 | 0.0755323353293413 | -0.0355732934131737 |
| 662 | 0.0467298461538462 | 0.0940499401197605 | -0.279388423153693 | -0.0212790019960080 |
| 663 | 0.0659224615384615 | -0.375550019960080 | 0.0142130738522954 | 0.0628623952095808 |
| 664 | 0.0609156923076923 | -0.0566898602794411 | 0.364260778443114 | 0.0206292614770459 |
| 665 | 0.0619587692307692 | 0.246901397205589 | -0.0950245508982036 | -0.0378473852295409 |
| 666 | 0.0736412307692308 | -0.0672481437125748 | -0.187206487025948 | 0.0854408782435130 |
| 667 | 0.101595692307692 | -0.106395009980040 | 0.361012075848303 | 0.145379441117764 |
| 668 | 0.109731692307692 | 0.224322914171657 | 0.306190219560878 | 0.0562025548902196 |
| 669 | 0.0861581538461538 | 0.349397964071856 | -0.174211676646707 | 0.0102334131736527 |
| 670 | 0.0394283076923077 | -0.0388219960079840 | -0.191673453093812 | 0.107369620758483 |
| 671 | 0.0102221538461538 | -0.165358962075848 | 0.221317864271457 | 0.0908012375249501 |
| 672 | 0.0296233846153846 | -0.0696846706586826 | 0.0215226546906188 | -0.164871656686627 |
| 673 | 0.0429747692307692 | -0.154800678642715 | -0.255023153692615 | -0.0955118562874252 |
| 674 | 0.0212787692307692 | -0.110293453093812 | -0.105988922155689 | 0.196871377245509 |
| 675 | -0.0319181538461538 | 0.0412585229540918 | 0.181927345309381 | -0.0157562075848303 |
| 676 | -0.0773963076923077 | 0.0808926946107784 | -0.00527914171656687 | -0.285073652694611 |
| 677 | -0.0901218461538462 | 0.0617253493013972 | -0.302941516966068 | 0.0285885828343313 |
| 678 | -0.0675913846153846 | 0.102496566866267 | 0.0601009980039920 | 0.210840798403194 |
| 679 | -0.00792738461538461 | 0.0456442714570858 | 0.243246606786427 | -0.149277884231537 |
| 680 | 0.0104307692307692 | -0.102334131736527 | -0.125887225548902 | -0.228383792415170 |
| 681 | -0.0120996923076923 | -0.217825508982036 | -0.414615668662675 | 0.147653532934132 |
| 682 | -0.0304578461538462 | -0.109643712574850 | 0.173805588822355 | 0.143105349301397 |
| 683 | -0.0235735384615385 | 0.129298363273453 | 0.281012774451098 | -0.274190499001996 |
| 684 | 0.0175236923076923 | 0.125562355289421 | -0.210759580838323 | -0.162597564870259 |
| 685 | 0.0250338461538462 | -0.0750450299401198 | -0.131572455089820 | 0.230170578842315 |
| 686 | 0.0125169230769231 | 0.0472686227544910 | 0.164871656686627 | -0.0238779640718563 |
| 687 | -0.0233649230769231 | 0.322433732534930 | 0.240810079840319 | -0.291408622754491 |
| 688 | -0.0786480000000000 | 0.0734206786427146 | -0.0150252495009980 | 0.0258271856287425 |
| 689 | -0.0684258461538462 | -0.184363872255489 | -0.0426392215568862 | 0.159348862275449 |
| 690 | -0.0444350769230769 | -0.0274515369261477 | -0.0950245508982036 | -0.172830978043912 |
| 691 | -0.0500676923076923 | 0.0506797604790419 | 0.0328931137724551 | -0.105257964071856 |
| 692 | -0.0830289230769231 | 0.228383792415170 | 0.216038722554890 | 0.250150099800399 |
| 693 | -0.123291692307692 | 0.448970698602794 | -0.0970549900199601 | 0.0782937325349302 |
| 694 | -0.115155692307692 | -0.404138602794411 | -0.361824251497006 | -0.188749620758483 |
| 695 | -0.0740584615384615 | -0.924255888223553 | -0.0341113772455090 | 0.000649740518962076 |
| 696 | -0.0179409230769231 | 0.129785668662675 | 0.283449301397206 | 0.206942355289421 |
| 697 | -0.0191926153846154 | 0.474960319361277 | -0.0158374251497006 | -0.0396341716566866 |
| 698 | -0.0728067692307692 | -0.0891768862275449 | -0.220505688622755 | -0.148628143712575 |
| 699 | -0.114947076923077 | -0.0563649900199601 | -0.0726897205588822 | 0.148953013972056 |
| 700 | -0.126420923076923 | -0.00601009980039920 | 0.259490119760479 | 0.122476087824351 |
| 701 | -0.0748929230769231 | 0.243002954091816 | 0.187206487025948 | -0.0563649900199601 |
| 702 | -0.0310836923076923 | 0.832804910179641 | -0.296038023952096 | 0.111268063872255 |
| 703 | -0.0123083076923077 | -0.163084870259481 | -0.164059481037924 | 0.147815968063872 |

| | | | | |
|---|---|---|---|---|
| 704 | -0.00751015384615385 | -1.14403061876248 | 0.250150099800399 | -0.0742328542914172 |
| 705 | -0.0183581538461538 | 0.230982754491018 | 0.0381722554890220 | -0.00324870259481038 |
| 706 | -0.00584123076923077 | 1.09042702594810 | -0.234312674650699 | 0.135958203592814 |
| 707 | 0.0231563076923077 | -0.201744431137725 | 0.0629436127744511 | -0.0860906187624751 |
| 708 | 0.0377593846153846 | -0.545944471057884 | 0.259896207584830 | -0.217825508982036 |
| 709 | -0.00104307692307692 | 0.573883313373254 | -0.0690349301397206 | 0.0584766467065868 |
| 710 | -0.0619587692307692 | 0.441336247504990 | -0.103958483033932 | 0.208566706586826 |
| 711 | -0.0819858461538462 | -0.134821157684631 | 0.281012774451098 | -0.0302129341317365 |
| 712 | -0.0636276923076923 | -0.0469437524950100 | 0.196952594810379 | -0.0758572055888224 |
| 713 | -0.0248252307692308 | -0.365966347305389 | -0.141724650698603 | 0.121663912175649 |
| 714 | 0.0244080000000000 | -0.663222634730539 | -0.0881210578842315 | 0.0813800000000000 |
| 715 | 0.0569520000000000 | 0.0497051497005988 | 0.0917758483033932 | -0.0368727744510978 |
| 716 | 0.0617501538461538 | 0.341276207584830 | -0.00527914171656687 | 0.0472686227544910 |
| 717 | 0.0452695384615385 | -0.496401756487026 | -0.100709780439122 | 0.0430453093812375 |
| 718 | 0.0400541538461538 | -0.211328103792415 | -0.0759384231536926 | -0.0807302594810379 |
| 719 | 0.0423489230769231 | 0.633659441117765 | -0.0174617764471058 | 0.0399590419161677 |
| 720 | 0.00146030769230769 | 0.169257405189621 | 0.154313373253493 | 0.124425309381238 |
| 721 | -0.0394283076923077 | -0.449782874251497 | 0.126699401197605 | -0.0402839121756487 |
| 722 | -0.0582036923076923 | 0.0183551696606786 | -0.113298502994012 | -0.0406087824351297 |
| 723 | -0.0521538461538462 | 0.301317165668663 | -0.175023852295409 | 0.105095528942116 |
| 724 | 0.00375507692307692 | -0.271266666666667 | 0.0938062874251497 | 0.0255023153692615 |
| 725 | 0.0529883076923077 | -0.192648063872255 | 0.150658582834331 | -0.121501477045908 |
| 726 | 0.0636276923076923 | 0.317398243512974 | -0.135227245508982 | -0.0375225149700599 |
| 727 | 0.0458953846153846 | 0.234393892215569 | -0.250556187624750 | 0.0294007584830339 |
| 728 | 0.0212787692307692 | -0.0402839121756487 | 0.0828419161676647 | -0.0992478642714571 |
| 729 | 0.0231563076923077 | 0.0519792415169661 | 0.195734331337325 | -0.0906388023952096 |
| 730 | 0.0267027692307692 | 0.0615629141716567 | 0.0284261477045908 | 0.0505173253493014 |
| 731 | 0.00584123076923077 | -0.0977859481037924 | -0.0174617764471058 | -0.00454818363273453 |
| 732 | -0.0369249230769231 | -0.267530658682635 | 0.00812175648702595 | -0.0678978842315369 |
| 733 | -0.0815686153846154 | -0.365966347305389 | 0.208323053892216 | 0.119552255489022 |
| 734 | -0.0869926153846154 | -0.0193297804391218 | -0.0263957085828343 | 0.203693652694611 |
| 735 | -0.0479815384615385 | 0.130760279441118 | 0.108831536926148 | -0.0230657884231537 |
| 736 | 0.0191926153846154 | 0.0449945309381238 | -0.181115169660679 | -0.0683851896207585 |
| 737 | 0.0698861538461539 | -0.0604258682634731 | 0.294413672654691 | 0.0581517764471058 |
| 738 | 0.0803169230769231 | -0.0680603193612775 | 0.459691417165669 | -0.0250150099800399 |
| 739 | 0.0769790769230769 | 0.108181796407186 | -0.758165968063872 | -0.0227409181636727 |
| 740 | 0.0788566153846154 | 0.122638522954092 | -0.872276646706587 | 0.0560401197604790 |
| 741 | 0.0905390769230769 | 0.100384910179641 | 0.595730838323353 | -0.107532055888224 |
| 742 | 0.0696775384615385 | 0.0445072255489022 | 1.43389610778443 | -0.0771566866267465 |
| 743 | 0.0158547692307692 | -0.0167308183632735 | -1.55612854291417 | 0.207916966067864 |
| 744 | -0.0471470769230769 | 0.0597761277445110 | -0.674511876247505 | 0.0778064271457086 |
| 745 | -0.0965889230769231 | 0.227896487025948 | 1.86028832335329 | -0.197358682634731 |
| 746 | -0.0746843076923077 | 0.0133196806387226 | 0.491366267465070 | 0.00601009980039920 |
| 747 | -0.0143944615384615 | -0.169907145708583 | -1.64587395209581 | 0.221561516966068 |

| | | | | |
|---|---|---|---|---|
| 748 | 0.0258683076923077 | 0.0797556487025948 | -0.480807984031936 | -0.0588015169660679 |
| 749 | 0.0496504615384615 | 0.0963240319361277 | 1.82252215568862 | -0.292545668662675 |
| 750 | 0.0565347692307692 | -0.0916134131736527 | -0.585578642714571 | -0.0713090219560878 |
| 751 | 0.0619587692307692 | -0.105095528942116 | -0.605883033932136 | 0.0134821157684631 |
| 752 | 0.0824030769230769 | 0.0921007185628743 | 0.325276347305389 | -0.136120638722555 |
| 753 | 0.0974233846153846 | -0.0451569660678643 | -0.0739079840319361 | -0.0319997205588822 |
| 754 | 0.0740584615384615 | -0.298555768463074 | -0.0934001996007984 | 0.000812175648702595 |
| 755 | 0.00229476923076923 | 0.0175429940119760 | 0.456442714570858 | -0.0974610778443114 |
| 756 | -0.0592467692307692 | 0.158861556886228 | 0.100709780439122 | 0.0989229940119761 |
| 757 | -0.0744756923076923 | -0.192485628742515 | -1.44851526946108 | 0.160323473053892 |
| 758 | -0.0413058461538462 | -0.0672481437125748 | 0.592482135728543 | -0.122476087824351 |
| 759 | 0.0206529230769231 | 0.303266387225549 | 1.09318842315369 | -0.126861836327345 |
| 760 | 0.0567433846153846 | 0.245764351297405 | -0.802429540918164 | 0.205642874251497 |
| 761 | 0.0655052307692308 | -0.0300504990019960 | -1.21582694610778 | 0.168445229540918 |
| 762 | 0.0552830769230769 | -0.0813800000000000 | 0.739485928143713 | -0.170556886227545 |
| 763 | 0.0663396923076923 | -0.0410960878243513 | 1.28283143712575 | -0.00763445109780439 |
| 764 | 0.102430153846154 | -0.0552279441117765 | -1.03389960079840 | 0.288322355289421 |
| 765 | 0.100344000000000 | 0.126049660678643 | -0.616847405189621 | 0.0609131736526946 |
| 766 | 0.0563261538461538 | 0.0630248303393214 | 0.686288423153693 | -0.106232574850299 |
| 767 | 0.00625846153846154 | 0.298230898203593 | 0.610350000000000 | 0.145866746506986 |
| 768 | -0.0146030769230769 | 0.00958367265469062 | -0.466594910179641 | 0.185013612774451 |
| 769 | -0.0112652307692308 | -0.764257285429142 | -0.294007584830339 | -0.0503548902195609 |
| 770 | 0.0187753846153846 | -0.238454770459082 | 0.542939421157685 | 0.0493802794411178 |
| 771 | 0.0644621538461539 | 0.415509061876248 | -0.223348303393214 | 0.176891856287425 |
| 772 | 0.0821944615384615 | 0.238292335329341 | -0.159592514970060 | -0.125399920159681 |
| 773 | 0.0803169230769231 | -0.198170858283433 | 0.0198983033932136 | -0.196708942115768 |
| 774 | 0.0824030769230769 | 0.0625375249500998 | 0.0588827345309381 | 0.155450419161677 |
| 775 | 0.0970061538461539 | 0.409986267465070 | -0.178272554890220 | 0.0417458283433134 |
| 776 | 0.0999267692307692 | 0.158861556886228 | 0.105176746506986 | -0.350859880239521 |
| 777 | 0.0504849230769231 | -0.300342554890220 | 0.332179840319361 | -0.0807302594810379 |
| 778 | -0.0342129230769231 | -0.625537684630738 | -0.321215469061876 | 0.331205229540918 |
| 779 | -0.0945027692307692 | 0.0758572055888224 | 0.116141117764471 | 0 |
| 780 | -0.0794824615384615 | 0.807465029940120 | 0.355732934131737 | -0.330068183632735 |
| 781 | -0.00980492307692308 | 0.195734331337325 | 0.114110678642715 | 0.111105628742515 |
| 782 | 0.0686344615384615 | -0.367753133732535 | -0.441823552894212 | 0.365479041916168 |
| 783 | 0.116616000000000 | 0.180140558882236 | -0.0397966067864271 | -0.111430499001996 |
| 784 | 0.109523076923077 | 0.556665189620759 | 0.421925249500998 | -0.209378882235529 |
| 785 | 0.0957544615384615 | 0.136445508982036 | -0.106395009980040 | 0.260058642714571 |
| 786 | 0.0965889230769231 | -0.351996926147705 | -0.304159780439122 | 0.148790578842315 |
| 787 | 0.105350769230769 | -0.409823832335329 | 0.0889332335329341 | -0.304890738522954 |
| 788 | 0.0917907692307692 | -0.173643153692615 | 0.530350698602794 | -0.0233906586826347 |
| 789 | 0.0264941538461538 | 0.251774451097804 | -0.219287425149701 | 0.382047425149701 |
| 790 | -0.0465212307692308 | 0.259896207584830 | -0.382534730538922 | -0.0352484231536926 |
| 791 | -0.0909563076923077 | -0.527264431137725 | 0.113704590818363 | -0.416158802395210 |

| | | | | |
|---|---|---|---|---|
| 792 | -0.0778135384615385 | -0.369052614770459 | 0.283043213572854 | 0.0682227544910180 |
| 793 | -0.0250338461538462 | 0.547731257485030 | -0.194109980039920 | 0.344362475049900 |
| 794 | 0.0285803076923077 | 0.186637964071856 | -0.298880638722555 | -0.192972934131737 |
| 795 | 0.0621673846153846 | -0.641293892215569 | 0.260302295409182 | -0.323408343313373 |
| 796 | 0.0703033846153846 | -0.307164830339321 | 0.170962974051896 | 0.229033532934132 |
| 797 | 0.0888701538461539 | 0.442798163672655 | 0.0649740518962076 | 0.219449860279441 |
| 798 | 0.115781538461538 | 0.134658722554890 | -0.0235530938123753 | -0.295469500998004 |
| 799 | 0.121414153846154 | -0.244140000000000 | 0.122638522954092 | -0.0964864670658683 |
| 800 | 0.0725981538461538 | 0.100709780439122 | 0.0889332335329341 | 0.314474411177645 |
| 801 | -0.00688430769230769 | 0.0968113373253493 | 0.144973353293413 | -0.0271266666666667 |
| 802 | -0.0548658461538462 | 0.00438574850299401 | 0.00893393213572854 | -0.266880918163673 |
| 803 | -0.0588295384615385 | 0.293357844311377 | -0.294007584830339 | 0.174455329341317 |
| 804 | -0.0246166153846154 | 0.310413532934132 | 0.0674105788423154 | 0.291733493013972 |
| 805 | 0.0241993846153846 | -0.0914509780439122 | 0.167714271457086 | -0.157399640718563 |
| 806 | 0.0546572307692308 | -0.131572455089820 | -0.122232435129741 | -0.146516487025948 |
| 807 | 0.0506935384615385 | 0.157724510978044 | -0.327712874251497 | 0.260870818363273 |
| 808 | 0.0473556923076923 | 0.0467813173652695 | 0.0503548902195609 | 0.133034371257485 |
| 809 | 0.0644621538461539 | -0.0633497005988024 | 0.194922155688623 | -0.271429101796407 |
| 810 | 0.0657138461538462 | 0.0266393612774451 | -0.0475122754491018 | -0.0834916566866268 |
| 811 | 0.0350473846153846 | -0.119227385229541 | -0.134008982035928 | 0.170719321357285 |
| 812 | -0.0383852307692308 | -0.270129620758483 | 0.0259896207584830 | -0.102334131736527 |
| 813 | -0.123291692307692 | -0.0501924550898204 | 0.268017964071856 | -0.192972934131737 |
| 814 | -0.142275692307692 | 0.101846826347305 | -0.0536035928143713 | 0.0326494610778443 |
| 815 | -0.0945027692307692 | -0.167145748502994 | -0.370352095808383 | -0.0102334131736527 |
| 816 | -0.0252424615384615 | -0.259896207584830 | -0.145785528942116 | -0.126536966067864 |
| 817 | 0.0233649230769231 | -0.0168932534930140 | 0.280606686626747 | 0.0571771656686627 |
| 818 | 0.0381766153846154 | 0.0430453093812375 | 0.0158374251497006 | 0.147003792415170 |
| 819 | 0.0477729230769231 | -0.129948103792415 | -0.227003093812375 | -0.0537660279441118 |
| 820 | 0.0477729230769231 | -0.121501477045908 | 0.175023852295409 | -0.105907704590818 |
| 821 | 0.0523624615384615 | 0.0765069461077844 | 0.277764071856287 | 0.127998882235529 |
| 822 | 0.0306664615384615 | 0.199145469061876 | -0.0730958083832335 | 0.161785389221557 |
| 823 | -0.0388024615384615 | 0.213927065868264 | -0.299286726546906 | -0.0878774051896208 |
| 824 | -0.108062769230769 | 0.0581517764471058 | 0.0389844311377246 | -0.155775289421158 |
| 825 | -0.152706461538462 | -0.176242115768463 | -0.0198983033932136 | 0.0419082634730539 |
| 826 | -0.129967384615385 | 0.0121826347305389 | -0.198983033932136 | 0.148790578842315 |
| 827 | -0.0788566153846154 | 0.279875728542914 | -0.103958483033932 | -0.0138069860279441 |
| 828 | -0.0317095384615385 | -0.0880398403193613 | -0.0580705588822355 | -0.166333572854291 |
| 829 | -0.00751015384615385 | -0.216688463073852 | 0.0633497005988024 | -0.0625375249500998 |
| 830 | -0.0256596923076923 | 0.117115728542914 | 0.0174617764471058 | 0.196708942115768 |
| 831 | -0.0214873846153846 | 0.140181516966068 | 0.0381722554890220 | 0.172343672654691 |
| 832 | -0.00438092307692308 | -0.00568522954091816 | -0.0223348303393214 | -0.105420399201597 |
| 833 | -0.00479815384615385 | -0.0167308183632735 | 0.168526447105788 | -0.114841636726547 |
| 834 | -0.0371335384615385 | 0.0695222355289421 | 0.170962974051896 | 0.144729700598802 |
| 835 | -0.103890461538462 | -0.0485681037924152 | -0.105988922155689 | 0.169419840319361 |

| | | | | |
|---|---|---|---|---|
| 836 | -0.149577230769231 | -0.177866467065868 | -0.122638522954092 | -0.115491377245509 |
| 837 | -0.144570461538462 | -0.0253398802395210 | -0.00974610778443114 | -0.109156407185629 |
| 838 | -0.0832375384615385 | -0.0410960878243513 | 0.119389820359281 | 0.0924255888223553 |
| 839 | 0.00876184615384615 | -0.0350859880239521 | -0.141724650698603 | 0.0367103393213573 |
| 840 | 0.0582036923076923 | 0.205967744510978 | -0.0747201596806387 | -0.0474310578842315 |
| 841 | 0.0675913846153846 | 0.209541317365269 | 0.117765469061876 | 0.0339489421157685 |
| 842 | 0.0707206153846154 | -0.0990854291417166 | 0.155125548902196 | 0.0536035928143713 |
| 843 | 0.0794824615384615 | -0.134171417165669 | 0.0117765469061876 | -0.00698471057884232 |
| 844 | 0.0924166153846154 | 0.441823552894212 | 0.0194922155688623 | 0.0604258682634731 |
| 845 | 0.0748929230769231 | 0.323895648702595 | 0.148628143712575 | 0.0930753293413174 |
| 846 | 0.0139772307692308 | -0.0804053892215569 | -0.00649740518962076 | -0.0592888223552894 |
| 847 | -0.0705120000000000 | 0.110131017964072 | -0.0357357285429142 | -0.166983313373254 |
| 848 | -0.121831384615385 | 0.0336240718562874 | -0.0540096806387226 | 0.0466188822355289 |
| 849 | -0.115155692307692 | 0.0152689021956088 | 0.0170556886227545 | 0.158861556886228 |
| 850 | -0.0611243076923077 | 0.0256647504990020 | -0.152689021956088 | -0.133684111776447 |
| 851 | 0.00855323076923077 | -0.399265548902196 | 0.408524351297405 | -0.173155848303393 |
| 852 | 0.0429747692307692 | -0.592400918163673 | 0.290352794411178 | 0.127674011976048 |
| 853 | 0.0438092307692308 | 0.0974610778443114 | -1.07004141716567 | 0.178353772455090 |
| 854 | 0.0446436923076923 | 0.444422514970060 | -0.451975748502994 | -0.0851160079840319 |
| 855 | 0.0417230769230769 | -0.140993692614770 | 1.71612714570858 | -0.149440319361277 |
| 856 | 0.0300406153846154 | -0.238454770459082 | 0.199389121756487 | 0.125399920159681 |
| 857 | 0.00479815384615385 | 0.192648063872255 | -2.29358403193613 | 0.156749900199601 |
| 858 | -0.0634190769230769 | 0.232931976047904 | 0.696034530938124 | -0.189886666666667 |
| 859 | -0.108897230769231 | -0.0846287025948104 | 1.98089640718563 | -0.177216726546906 |
| 860 | -0.0857409230769231 | -0.231145189620758 | -1.19471037924152 | 0.170069580838323 |
| 861 | -0.0135600000000000 | -0.136283073852295 | -1.70232015968064 | 0.0576644710578842 |
| 862 | 0.0567433846153846 | 0.0846287025948104 | 1.31978542914172 | -0.191186147704591 |
| 863 | 0.0571606153846154 | 0.392768143712575 | 1.16019291417166 | 0.0206292614770459 |
| 864 | 0.0285803076923077 | 0.360443552894212 | -1.20811127744511 | 0.191348582834331 |
| 865 | -0.00250338461538462 | 0.0240403992015968 | 0.0856845309381238 | -0.0513295009980040 |
| 866 | -0.00813600000000000 | 0.0324870259481038 | 0.745171157684631 | -0.124750179640719 |
| 867 | 0.00542400000000000 | 0.151551976047904 | -0.387001696606786 | 0.153826067864271 |
| 868 | 0.000834461538461538 | -0.0589639520958084 | 0.0523853293413174 | 0.156425029940120 |
| 869 | -0.0158547692307692 | -0.152526586826347 | 0.761008582834331 | -0.155287984031936 |
| 870 | -0.0611243076923077 | -0.0825170459081836 | -0.666796207584830 | -0.0781312974051896 |
| 871 | -0.0705120000000000 | -0.0823546107784431 | -0.799180838323353 | 0.221074211576846 |
| 872 | -0.0344215384615385 | -0.0691973652694611 | 1.34861766467066 | 0.0446696606786427 |
| 873 | 0.0183581538461538 | 0.0609131736526946 | 0.478371457085828 | -0.163572175648703 |
| 874 | 0.0700947692307692 | 0.135958203592814 | -1.31775499001996 | 0.104770658682635 |
| 875 | 0.0669655384615385 | -0.161622954091816 | -0.175836027944112 | 0.235855808383234 |
| 876 | 0.0467298461538462 | -0.262170299401198 | 1.60485908183633 | -0.0690349301397206 |
| 877 | 0.0267027692307692 | -0.0279388423153693 | 0.211571756487026 | -0.152526586826347 |
| 878 | 0.0296233846153846 | 0.0789434730538922 | -1.55937724550898 | 0.0573396007984032 |
| 879 | 0.0481901538461539 | 0.0134821157684631 | 0.0714714570858283 | 0.0297256287425150 |

| | | | | |
|---|---|---|---|---|
| 880 | 0.0269113846153846 | -0.00519792415169661 | 1.12770588822355 | -0.167633053892216 |
| 881 | -0.0148116923076923 | 0.0566898602794411 | -0.193703892215569 | -0.0934001996007984 |
| 882 | -0.0400541538461538 | 0.137582554890220 | -1.03552395209581 | 0.0969737724550898 |
| 883 | 0.00312923076923077 | 0.203856087824351 | 0.128729840319361 | -0.0212790019960080 |
| 884 | 0.0803169230769231 | -0.0672481437125748 | 0.695628443113773 | -0.0966489021956088 |
| 885 | 0.118910769230769 | -0.175592375249501 | -0.401620858283433 | 0.149440319361277 |
| 886 | 0.110148923076923 | 0.0391468662674651 | -0.387813872255489 | 0.123775568862275 |
| 887 | 0.0550744615384615 | 0.0823546107784431 | 0.327306786427146 | -0.211652974051896 |
| 888 | 0.0129341538461538 | 0.0297256287425150 | 0.0994915169660679 | -0.0968113373253493 |
| 889 | 0.00980492307692308 | 0.0584766467065868 | -0.368321656686627 | 0.188262315369261 |
| 890 | 0.0227390769230769 | -0.0393093013972056 | 0.0702531936127745 | -0.0514919361277445 |
| 891 | 0.0273286153846154 | -0.115491377245509 | 0.109643712574850 | -0.264444391217565 |
| 892 | -0.0175236923076923 | 0.0139694211576846 | -0.400808682634731 | -0.0206292614770459 |
| 893 | -0.0579950769230769 | -0.0838165269461078 | 0.0211165668662675 | 0.136445508982036 |
| 894 | -0.0504849230769231 | -0.257297245508982 | 0.449539221556886 | -0.0146191616766467 |
| 895 | -0.000208615384615385 | -0.143592654690619 | -0.0194922155688623 | -0.0363854690618763 |
| 896 | 0.0815686153846154 | 0.0609131736526946 | -0.375631237524950 | 0.109806147704591 |
| 897 | 0.116407384615385 | 0.00178678642714571 | 0.343550299401198 | 0.0756947704590818 |
| 898 | 0.103681846153846 | 0.0745577245508982 | 0.544563772455090 | 0.0539284630738523 |
| 899 | 0.0669655384615385 | 0.230333013972056 | -0.259084031936128 | 0.0895017564870260 |
| 900 | 0.0168978461538462 | 0.0328118962075848 | -0.285885828343313 | -0.0373600798403194 |
| 901 | 0.0106393846153846 | -0.128648622754491 | 0.395123453093812 | -0.128486187624751 |
| 902 | 0.0168978461538462 | -0.0341113772455090 | 0.162841217564870 | 0.0631872654690619 |
| 903 | 0.0260769230769231 | 0.0644867465069860 | -0.546188123752495 | 0.108181796407186 |
| 904 | 0.0160633846153846 | -0.0722836327345309 | -0.268017964071856 | -0.166496007984032 |
| 905 | -0.0114738461538462 | -0.0475934930139721 | 0.365479041916168 | -0.0243652694610778 |
| 906 | 0.00521538461538462 | 0.102496566866267 | 0.0889332335329341 | 0.246089221556886 |
| 907 | 0.0331698461538462 | 0.121339041916168 | -0.500300199600798 | 0.0427204391217565 |
| 908 | 0.0613329230769231 | 0.0945372455089820 | 0.0548218562874252 | -0.170556886227545 |
| 909 | 0.0406800000000000 | -0.0477559281437126 | 0.533193313373254 | 0.0516543712574850 |
| 910 | 0.00563261538461538 | -0.0497051497005988 | -0.110455888223553 | 0.223835608782435 |
| 911 | 0.00604984615384615 | 0.0414209580838323 | -0.403245209580838 | -0.0464564471057884 |
| 912 | 0.0154375384615385 | -0.00665984031936128 | 0.131166367265469 | -0.148303273453094 |
| 913 | 0.0546572307692308 | -0.0870652295409182 | 0.222536127744511 | 0.0674105788423154 |
| 914 | 0.0598726153846154 | 0.00211165668662675 | -0.270860578842315 | 0.0776439920159681 |
| 915 | 0.0315009230769231 | -0.100709780439122 | -0.214008283433134 | -0.132222195608782 |
| 916 | 0.0141858461538462 | -0.158699121756487 | 0.0714714570858283 | -0.0157562075848303 |
| 917 | 0.0183581538461538 | -0.256972375249501 | 0.0596949101796407 | 0.127186706586826 |
| 918 | 0.0619587692307692 | -0.356057804391218 | 0.0385783433133733 | 0.00471061876247505 |
| 919 | 0.0813600000000000 | 0.718613013972056 | 0.0686288423153693 | 0.0753699001996008 |
| 920 | 0.0648793846153846 | 1.12794954091816 | -0.104770658682635 | 0.154475808383234 |
| 921 | 0.0310836923076923 | 0.414534451097804 | -0.218881337325349 | -0.0310251097804391 |
| 922 | -0.00667569230769231 | -0.256322634730539 | 0.0775627744510978 | -0.131247584830339 |
| 923 | -0.0146030769230769 | -0.573558443113772 | 0.0649740518962076 | 0.109968582834331 |

| | | | | |
|---|---|---|---|---|
| 924 | -0.0143944615384615 | -0.0391468662674651 | -0.342332035928144 | 0.0193297804391218 |
| 925 | -0.00250338461538462 | 0.139531776447106 | -0.212790019960080 | -0.285073652694611 |
| 926 | -0.00458953846153846 | -0.455792974051896 | 0.276545808383234 | -0.0633497005988024 |
| 927 | -0.0354646153846154 | -0.931565469061876 | 0.187206487025948 | 0.159186427145709 |
| 928 | -0.0292061538461538 | 0.246251656686627 | -0.224566566866267 | -0.0597761277445110 |
| 929 | 0.0100135384615385 | 1.24262874251497 | 0.00690349301397206 | -0.114191896207585 |
| 930 | 0.0531969230769231 | -0.145704311377246 | 0.587609081836327 | 0.225947265469062 |
| 931 | 0.0707206153846154 | -0.877961876247505 | 0.155937724550898 | 0.178841077844311 |
| 932 | 0.0496504615384615 | 0.256485069860279 | -0.280200598802395 | -0.148953013972056 |
| 933 | 0.0241993846153846 | 0.441336247504990 | 0.225378742514970 | -0.00909636726546906 |
| 934 | -0.000834461538461538 | -0.492340878243513 | 0.450351397205589 | 0.239104510978044 |
| 935 | 0.00125169230769231 | -0.458067065868263 | -0.156749900199601 | -0.0620502195608782 |
| 936 | 0.00354646153846154 | 0.0524665469061876 | -0.434107884231537 | -0.267043353293413 |
| 937 | -0.0250338461538462 | 0.158049381237525 | 0.297662375249501 | 0.140993692614770 |
| 938 | -0.0529883076923077 | 0.345986826347305 | 0.259896207584830 | 0.268017964071856 |
| 939 | -0.0531969230769231 | 0.578593932135729 | -0.216038722554890 | -0.0982732534930140 |
| 940 | -0.00312923076923077 | -0.0204668263473054 | -0.183957784431138 | -0.191511017964072 |
| 941 | 0.0506935384615385 | -0.158049381237525 | 0.157155988023952 | 0.101521956087824 |
| 942 | 0.0611243076923077 | 0.671181956087824 | 0.155125548902196 | 0.140831257485030 |
| 943 | 0.0327526153846154 | 0.173155848303393 | -0.118983732534930 | -0.111917804391218 |
| 944 | -0.0127255384615385 | -0.689861996007984 | -0.0357357285429142 | -0.163409740518962 |
| 945 | -0.0458953846153846 | -0.126049660678643 | -0.155125548902196 | 0.0102334131736527 |
| 946 | -0.0292061538461538 | 0.435163712574850 | 0.0661923153692615 | 0.0597761277445110 |
| 947 | 0.00980492307692308 | -0.0760196407185629 | 0.179084730538922 | -0.0334616367265469 |
| 948 | 0.0258683076923077 | -0.342900558882236 | 0.0101521956087824 | -0.0776439920159681 |
| 949 | 0.00375507692307692 | 0.0844662674650699 | -0.271266666666667 | -0.0852784431137725 |
| 950 | -0.0354646153846154 | 0.0903139321357285 | -0.0158374251497006 | 0.00146191616766467 |
| 951 | -0.0327526153846154 | -0.101197085828343 | 0.484056686626747 | 0.0773191217564870 |
| 952 | -0.000625846153846154 | -0.0415833932135729 | -0.00446696606786427 | 0.0198170858283433 |
| 953 | 0.0285803076923077 | -0.0683851896207585 | -0.328118962075848 | -0.129460798403194 |
| 954 | 0.0267027692307692 | -0.143105349301397 | -0.00893393213572854 | -0.0523041117764471 |
| 955 | 0.00688430769230769 | -0.0674105788423154 | 0.233906586826347 | 0.184363872255489 |
| 956 | -0.0179409230769231 | 0.0550655089820359 | -0.106801097804391 | 0.126212095808383 |
| 957 | -0.0287889230769231 | 0.0839789620758483 | -0.307002395209581 | -0.117927904191617 |
| 958 | 0.00730153846153846 | 0.240079121756487 | -0.0450757485029940 | -0.130110538922156 |
| 959 | 0.0289975384615385 | 0.103796047904192 | 0.150252495009980 | 0.101684391217565 |
| 960 | 0.0133513846153846 | -0.283774171656687 | -0.0345174650698603 | 0.0383346906187625 |
| 961 | -0.0350473846153846 | 0.0501924550898204 | -0.0353296407185629 | -0.149602754491018 |
| 962 | -0.0690516923076923 | 0.350859880239521 | 0.169744710578842 | -0.0251774451097804 |
| 963 | -0.0504849230769231 | -0.0678978842315369 | 0.114516766467066 | 0.131085149700599 |
| 964 | -0.0181495384615385 | -0.252261756487026 | 0.380504291417166 | -0.00438574850299401 |
| 965 | 0.00354646153846154 | -0.0155937724550898 | 0.165683832335329 | -0.0995727345309381 |
| 966 | -0.0210701538461538 | -0.0680603193612775 | -1.18537035928144 | 0.117440598802395 |
| 967 | -0.0496504615384615 | -0.210515928143713 | -0.234718762475050 | 0.135470898203593 |

| | | | | |
|---|---|---|---|---|
| 968 | -0.0415144615384615 | 0.0344362475049900 | 1.89602405189621 | -0.125399920159681 |
| 969 | -0.0162720000000000 | 0.0687100598802395 | -0.458473153692615 | -0.0851160079840319 |
| 970 | 0.0221132307692308 | -0.225135089820359 | -2.19855948103792 | 0.280850339321357 |
| 971 | 0.0337956923076923 | 0.0190049101796407 | 1.42699261477046 | 0.138232295409182 |
| 972 | -0.00375507692307692 | 0.259896207584830 | 2.17460029940120 | -0.286698003992016 |
| 973 | -0.0421403076923077 | -0.177541596806387 | -1.94028762475050 | -0.00113704590818363 |
| 974 | -0.0369249230769231 | -0.271591536926148 | -1.34780548902196 | 0.406900000000000 |
| 975 | 0.0108480000000000 | 0.220099600798403 | 2.01338343313373 | 0.0103958483033932 |
| 976 | 0.0688430769230769 | 0.266393612774451 | 0.471061876247505 | -0.443285469061876 |
| 977 | 0.0844892307692308 | -0.288972095808383 | -1.49480928143713 | -0.0282637125748503 |
| 978 | 0.0627932307692308 | -0.137582554890220 | 0.162841217564870 | 0.321296686626747 |
| 979 | 0.0296233846153846 | 0.326007305389222 | 0.591263872255489 | -0.126049660678643 |
| 980 | 0.00458953846153846 | -0.0329743313373254 | -0.903139321357286 | -0.332992015968064 |
| 981 | -0.00438092307692308 | -0.204018522954092 | 0.400402594810379 | 0.154638243512974 |
| 982 | -0.0154375384615385 | 0.233581716566866 | 0.881616666666667 | 0.279225988023952 |
| 983 | -0.0319181538461538 | 0.308626746506986 | -1.30232365269461 | -0.174292894211577 |
| 984 | -0.0613329230769231 | -0.241378602794411 | -0.418270459081836 | -0.151714411177645 |
| 985 | -0.0698861538461539 | -0.227734051896208 | 1.74089850299401 | 0.240079121756487 |
| 986 | -0.0415144615384615 | 0.246251656686627 | 0.179084730538922 | 0.0552279441117765 |
| 987 | -0.00271200000000000 | 0.0882022754491018 | -1.85013612774451 | -0.229520838323353 |
| 988 | 0.0116824615384615 | -0.180140558882236 | 0.000812175648702595 | 0.109643712574850 |
| 989 | -0.0164806153846154 | -0.0622126546906188 | 1.56384421157685 | 0.238292335329341 |
| 990 | -0.0429747692307692 | -0.0285885828343313 | -0.370758183632735 | -0.129948103792415 |
| 991 | -0.0381766153846154 | -0.251774451097804 | -1.49846407185629 | -0.125399920159681 |
| 992 | -0.0146030769230769 | -0.271916407185629 | 0.270860578842315 | 0.173805588822355 |
| 993 | -0.000417230769230769 | 0.169907145708583 | 1.11024411177645 | 0.104608223552894 |
| 994 | -0.0108480000000000 | 0.525315209580838 | -0.336646806387226 | -0.178678642714571 |
| 995 | -0.0440178461538462 | 0.189561796407186 | -0.492990618762475 | -0.0914509780439122 |
| 996 | -0.0805255384615385 | 0.0971362075848303 | 0.596949101796407 | 0.166171137724551 |
| 997 | -0.0782307692307692 | 0.130435409181637 | 0.343550299401198 | 0.0555528143712575 |
| 998 | -0.0511107692307692 | -0.591751177644711 | -0.356139021956088 | -0.124100439121756 |
| 999 | -0.0254510769230769 | -0.293845149700599 | 0.0503548902195609 | 0.0165683832335329 |
| 1000 | -0.0258683076923077 | 0.633009700598802 | 0.372788622754491 | 0.0503548902195609 |
| 1001 | -0.0690516923076923 | -0.201094690618762 | -0.277764071856287 | -0.100872215568862 |
| 1002 | -0.100761230769231 | -0.564137205588822 | -0.330555489021956 | -0.0513295009980040 |
| 1003 | -0.0976320000000000 | 0.870327425149701 | 0.211165668662675 | 0.0282637125748503 |
| 1004 | -0.0748929230769231 | 0.699608103792415 | 0.0227409181636727 | 0.00812175648702595 |
| 1005 | -0.0740584615384615 | -0.570797045908184 | -0.134415069860279 | 0.0199795209580838 |
| 1006 | -0.100344000000000 | -0.173480718562874 | 0.150252495009980 | 0.0540908982035928 |
| 1007 | -0.106602461538462 | 0.501437245508982 | 0.128729840319361 | 0.00828419161676647 |
| 1008 | -0.0857409230769231 | -0.178191337325349 | -0.149034231536926 | 0.0307002395209581 |
| 1009 | -0.0469384615384615 | -0.490229221556886 | -0.0698471057884232 | 0.124587744510978 |
| 1010 | -0.0110566153846154 | 0.256160199600798 | 0.198170858283433 | -0.0112080239520958 |
| 1011 | 0.0152289230769231 | 0.0414209580838323 | -0.129948103792415 | -0.109318842315369 |

| | | | | |
|---|---|---|---|---|
| 1012 | 0.0175236923076923 | -0.269967185628743 | -0.344768562874252 | 0.0497051497005988 |
| 1013 | -0.00229476923076923 | 0.465863952095808 | -0.0207104790419162 | 0.115491377245509 |
| 1014 | 0.00458953846153846 | 0.199145469061876 | 0.365479041916168 | -0.0435326147704591 |
| 1015 | 0.0227390769230769 | -0.717313532934132 | 0.0958367265469062 | -0.111430499001996 |
| 1016 | 0.0241993846153846 | 0.0968113373253493 | -0.398778243512974 | 0.0812175648702595 |
| 1017 | -0.0123083076923077 | 0.823870978043912 | 0.0101521956087824 | 0.153988502994012 |
| 1018 | -0.0675913846153846 | -0.187125269461078 | 0.509640219560878 | -0.133359241516966 |
| 1019 | -0.0832375384615385 | -0.488604870259481 | 0.245277045908184 | -0.206617485029940 |
| 1020 | -0.0630018461538462 | 0.417458283433134 | -0.501112375249501 | 0.0622126546906188 |
| 1021 | -0.00250338461538462 | 0.477721716566866 | -0.179084730538922 | 0.110131017964072 |
| 1022 | 0.0479815384615385 | -0.311388143712575 | 0.444260079840319 | -0.0316748502994012 |
| 1023 | 0.0461040000000000 | -0.253236367265469 | -0.0235530938123753 | -0.0867403592814371 |
| 1024 | 0.0189840000000000 | 0.154638243512974 | -0.376443413173653 | 0.0596136926147705 |
| 1025 | -0.00292061538461538 | -0.0373600798403194 | -0.131166367265469 | 0.114191896207585 |
| 1026 | 0.0198184615384615 | 0.00503548902195609 | 0.234312674650699 | -0.0677354491017964 |
| 1027 | 0.0456867692307692 | 0.0735831137724551 | 0.117765469061876 | -0.0469437524950100 |
| 1028 | 0.0559089230769231 | -0.290921317365269 | 0.00284261477045908 | 0.122638522954092 |
| 1029 | 0.0523624615384615 | -0.172018802395210 | -0.0389844311377246 | 0.0750450299401198 |
| 1030 | 0.0454781538461538 | 0.160648343313373 | 0.0649740518962076 | -0.0138069860279441 |
| 1031 | 0.0596640000000000 | -0.0612380439121757 | 0.325682435129741 | 0.0786186027944112 |
| 1032 | 0.0746843076923077 | -0.141805868263473 | 0.0686288423153693 | 0.105257964071856 |
| 1033 | 0.104307692307692 | -0.0490554091816367 | -0.272891017964072 | 0.0180302994011976 |
| 1034 | 0.0855323076923077 | -0.0534411576846307 | -0.187612574850299 | -0.0100709780439122 |
| 1035 | 0.0340043076923077 | -0.0714714570858283 | 0.216444810379242 | 0.0165683832335329 |
| 1036 | 0.00521538461538462 | 0.175592375249501 | 0.0986793413173653 | -0.0436950499001996 |
| 1037 | -0.0158547692307692 | 0.331367664670659 | -0.217663073852295 | -0.152689021956088 |
| 1038 | 0.00771876923076923 | -0.0945372455089820 | -0.0247713572854291 | -0.0422331337325349 |
| 1039 | 0.00917907692307692 | -0.132059760479042 | 0.303347604790419 | 0.0657862275449102 |
| 1040 | -0.0143944615384615 | 0.117603033932136 | 0.187206487025948 | -0.0479183632734531 |
| 1041 | -0.0302492307692308 | 0.0812175648702595 | -0.236343113772455 | -0.107694491017964 |
| 1042 | -0.0379680000000000 | -0.134171417165669 | -0.161216866267465 | -0.0284261477045908 |
| 1043 | 0.00625846153846154 | 0.0594512574850299 | 0.257865768463074 | 0.0144567265469062 |
| 1044 | 0.0588295384615385 | 0.158049381237525 | 0.0410148702594810 | -0.0539284630738523 |
| 1045 | 0.0936683076923077 | -0.112567544910180 | -0.365479041916168 | -0.0672481437125748 |
| 1046 | 0.0984664615384615 | 0.0206292614770459 | -0.0458879241516966 | 0.0243652694610778 |
| 1047 | 0.0763532307692308 | 0.0687100598802395 | 0.317154590818363 | 0.0583142115768463 |
| 1048 | 0.0665483076923077 | -0.138557165668663 | 0.0288322355289421 | 0.00909636726546906 |
| 1049 | 0.0821944615384615 | -0.151064670658683 | -0.250150099800399 | 0.0198170858283433 |
| 1050 | 0.104516307692308 | 0.153338762475050 | 0.110861976047904 | 0.0756947704590818 |
| 1051 | 0.0892873846153846 | -0.0380098203592814 | 0.190455189620759 | 0.0344362475049900 |
| 1052 | 0.0423489230769231 | -0.226759441117764 | -0.00649740518962076 | -0.0505173253493014 |
| 1053 | 0.0106393846153846 | -0.0167308183632735 | -0.164059481037924 | -0.00665984031936128 |
| 1054 | 0.0175236923076923 | 0.0607507385229541 | -0.0986793413173653 | 0.0985981237524950 |
| 1055 | 0.0531969230769231 | -0.0516543712574850 | 0.00934001996007984 | -0.0698471057884232 |

| | | | | |
|---|---|---|---|---|
| 1056 | 0.0796910769230769 | -0.138557165668663 | -0.0726897205588822 | -0.198008423153693 |
| 1057 | 0.0707206153846154 | 0.0289134530938124 | -0.0852784431137725 | 0.0898266267465070 |
| 1058 | 0.0431833846153846 | 0.0397966067864271 | -0.138475948103792 | 0.171369061876248 |
| 1059 | 0.00959630769230769 | 0.0734206786427146 | 0.0897454091816367 | -0.179978123752495 |
| 1060 | 0.0187753846153846 | -0.0371976447105788 | 0.159998602794411 | -0.237967465069860 |
| 1061 | 0.0415144615384615 | -0.0729333732534930 | 0.106801097804391 | 0.0869027944111777 |
| 1062 | 0.0467298461538462 | 0.0516543712574850 | -0.101928043912176 | 0.0771566866267465 |
| 1063 | 0.0375507692307692 | 0.187937445109780 | 0.00934001996007984 | -0.193135369261477 |
| 1064 | 0.00166892307692308 | 0.0362230339321357 | 0.239591816367265 | -0.0305378043912176 |
| 1065 | 0.0104307692307692 | -0.237805029940120 | -0.0869027944111777 | 0.276627025948104 |
| 1066 | 0.0467298461538462 | 0.0795932135728543 | -0.197358682634731 | 0.0644867465069860 |
| 1067 | 0.0919993846153846 | 0.244140000000000 | -0.0434513972055888 | -0.143755089820359 |
| 1068 | 0.114738461538462 | 0.406575129740519 | 0.118171556886228 | 0.169094970059880 |
| 1069 | 0.0940855384615385 | 0.0199795209580838 | -0.174617764471058 | 0.228546227544910 |
| 1070 | 0.0728067692307692 | -0.779363752495010 | -0.167308183632735 | -0.144892135728543 |
| 1071 | 0.0500676923076923 | -0.442310858283433 | 0.0125887225548902 | -0.00909636726546906 |
| 1072 | 0.0498590769230769 | 0.345012215568862 | 0.121420259481038 | 0.312037884231537 |
| 1073 | 0.0425575384615385 | 0.343387864271457 | -0.0381722554890220 | 0.00422331337325349 |
| 1074 | 0.0143944615384615 | -0.111592934131737 | -0.0414209580838323 | -0.249337924151697 |
| 1075 | -0.0229476923076923 | -0.0997351696606787 | 0.0491366267465070 | 0.0675730139720559 |
| 1076 | -0.0392196923076923 | 0.190698842315369 | -0.0442635728542914 | 0.209054011976048 |
| 1077 | 0.00292061538461538 | 0.868703073852295 | 0.334616367265469 | -0.0789434730538922 |
| 1078 | 0.0598726153846154 | 0.469762395209581 | -0.318778942115768 | -0.137257684630739 |
| 1079 | 0.0917907692307692 | -0.893393213572854 | -0.451163572854291 | 0.0501924550898204 |
| 1080 | 0.0640449230769231 | -0.405925389221557 | 0.228221357285429 | 0.0396341716566866 |
| 1081 | 0.0120996923076923 | 0.730958083832335 | 0.786186027944112 | -0.0657862275449102 |
| 1082 | -0.0235735384615385 | 0.363692255489022 | -0.490960179640719 | 0.0558776846307385 |
| 1083 | -0.0375507692307692 | -0.467488303393214 | -0.608319560878244 | 0.0752074650698603 |
| 1084 | -0.0104307692307692 | 0.0711465868263473 | 1.20770518962076 | -0.112405109780439 |
| 1085 | -0.000208615384615385 | 0.627974211576846 | 0.223348303393214 | 0.00129948103792415 |
| 1086 | -0.00876184615384615 | 0.193947544910180 | -0.712684131736527 | 0.106070139720559 |
| 1087 | -0.0114738461538462 | -0.119227385229541 | -0.288728443113772 | -0.0209541317365269 |
| 1088 | -0.0202356923076923 | -0.318372854291417 | 0.722430239520958 | -0.0396341716566866 |
| 1089 | 0.00584123076923077 | -0.620989500998004 | -0.243652694610778 | -0.0172181237524950 |
| 1090 | 0.0492332307692308 | -0.493153053892216 | -0.599385628742515 | 0.0146191616766467 |
| 1091 | 0.0655052307692308 | 0.189074491017964 | 0.329743313373253 | 0.0985981237524950 |
| 1092 | 0.0454781538461538 | 0.0646491816367266 | -0.287104091816367 | -0.0412585229540918 |
| 1093 | 0.00834461538461538 | -0.244140000000000 | 0.0219287425149701 | -0.184526307385230 |
| 1094 | -0.00458953846153846 | 0.310738403193613 | 0.351672055888224 | 0.0423955688622755 |
| 1095 | -0.00187753846153846 | 0.539447065868263 | 0.188830838323353 | 0.188424750499002 |
| 1096 | -0.0125169230769231 | -0.0812175648702595 | -0.477153193612775 | -0.0677354491017964 |
| 1097 | -0.0302492307692308 | -0.275814850299401 | 0.266799700598802 | -0.205642874251497 |
| 1098 | -0.0390110769230769 | 0.249662794411178 | 0.776033832335329 | 0.173643153692615 |
| 1099 | -0.0356732307692308 | 0.154963113772455 | -0.416646107784431 | 0.327794091816367 |

| | | | | |
|---|---|---|---|---|
| 1100 | -0.00354646153846154 | -0.275652415169661 | -0.488117564870260 | -0.103308742514970 |
| 1101 | 0.0354646153846154 | -0.121339041916168 | 0.235124850299401 | -0.175917245508982 |
| 1102 | 0.0350473846153846 | 0.447021477045908 | 0.611974351297405 | 0.216363592814371 |
| 1103 | -0.00438092307692308 | 0.349073093812375 | -0.441011377245509 | 0.177054291417166 |
| 1104 | -0.0648793846153846 | -0.119227385229541 | -0.503142814371258 | -0.242840518962076 |
| 1105 | -0.0899132307692308 | 0.00763445109780439 | 0.333804191616766 | -0.176566986027944 |
| 1106 | -0.0682172307692308 | 0.0791059081836327 | 0.339895508982036 | 0.202881477045908 |
| 1107 | -0.0285803076923077 | -0.193297804391218 | -0.113298502994012 | 0.0862530538922156 |
| 1108 | 0.00396369230769231 | -0.419407504990020 | -0.312687624750499 | -0.158861556886228 |
| 1109 | 0.00709292307692308 | -0.252586626746507 | 0.253804890219561 | -0.00584766467065868 |
| 1110 | 0.0102221538461538 | -0.0562025548902196 | 0.0467000998003992 | 0.102171696606786 |
| 1111 | 0.0310836923076923 | -0.0250150099800399 | -0.101115868263473 | -0.101197085828343 |
| 1112 | 0.0680086153846154 | 0.0779688622754491 | 0.0462940119760479 | -0.179328383233533 |
| 1113 | 0.101178461538462 | -0.0514919361277445 | 0.0174617764471058 | 0.0240403992015968 |
| 1114 | 0.0993009230769231 | -0.210028622754491 | 0.0450757485029940 | 0.0755323353293413 |
| 1115 | 0.0634190769230769 | 0.00779688622754491 | 0.168526447105788 | -0.0839789620758483 |
| 1116 | 0.00938769230769231 | 0.257459680638723 | 0.239997904191617 | -0.0412585229540918 |
| 1117 | -0.0227390769230769 | 0.153013892215569 | -0.0840601796407186 | 0.0984356886227545 |
| 1118 | -0.0135600000000000 | -0.0358981636726547 | 0.0519792415169661 | 0.0157562075848303 |
| 1119 | 0.00104307692307692 | 0.0268017964071856 | 0.218475249500998 | -0.0251774451097804 |
| 1120 | -0.00521538461538462 | 0.200769820359281 | 0.0759384231536926 | 0.0472686227544910 |
| 1121 | -0.0191926153846154 | 0.135795768463074 | -0.235530938123752 | 0.00162435129740519 |
| 1122 | -0.00855323076923077 | -0.0721211976047904 | -0.190455189620759 | 0.00341113772455090 |
| 1123 | 0.0333784615384615 | -0.189399361277445 | -0.0198983033932136 | 0.0318372854291417 |
| 1124 | 0.0742670769230769 | -0.0791059081836327 | -0.124262874251497 | -0.132222195608782 |
| 1125 | 0.0765618461538462 | 0.0989229940119761 | -0.183551696606786 | -0.111917804391218 |
| 1126 | 0.0350473846153846 | -0.125562355289421 | -0.179490818363273 | 0.0968113373253493 |
| 1127 | -0.00292061538461538 | -0.222048822355289 | 0.109237624750499 | 0.0648116167664671 |
| 1128 | -0.0177323076923077 | 0.0336240718562874 | -0.0142130738522954 | -0.0864154890219561 |
| 1129 | -0.0160633846153846 | -0.0539284630738523 | -0.0795932135728543 | -0.0656237924151697 |
| 1130 | -0.0104307692307692 | -0.198170858283433 | 0.0198983033932136 | 0.0869027944111777 |
| 1131 | -0.0187753846153846 | 0.0505173253493014 | 0.192485628742515 | 0.0813800000000000 |
| 1132 | -0.0331698461538462 | 0.00276139720558882 | 0.0296444111776447 | -0.0183551696606786 |
| 1133 | -0.0375507692307692 | -0.257297245508982 | -0.272078842315369 | 0.0441823552894212 |
| 1134 | -0.0189840000000000 | 0.0510046307385230 | 0.150658582834331 | 0.134333852295409 |
| 1135 | 0.000625846153846154 | 0.392605708582834 | 0.136445508982036 | -0.0222536127744511 |
| 1136 | 0.00438092307692308 | 0.133034371257485 | -0.0873088822355290 | -0.100384910179641 |
| 1137 | -0.0308750769230769 | -0.232282235528942 | -0.167714271457086 | 0.0389844311377246 |
| 1138 | -0.0869926153846154 | 0.0586390818363273 | 0.106395009980040 | -0.00454818363273453 |
| 1139 | -0.112235076923077 | 0.238779640718563 | 0.184363872255489 | -0.0636745708582834 |
| 1140 | -0.100969846153846 | 0.00893393213572854 | -0.0308626746506986 | 0.0872276646706587 |
| 1141 | -0.0469384615384615 | 0.107044750499002 | -0.0824358283433134 | 0.115004071856287 |
| 1142 | -0.00625846153846154 | 0.176079680638723 | -0.154719461077844 | -0.0419082634730539 |
| 1143 | -0.00876184615384615 | 0.384646387225549 | 0.0958367265469062 | -0.0235530938123753 |

| | | | | |
|---|---|---|---|---|
| 1144 | -0.0100135384615385 | -0.0337865069860280 | -0.0113704590818363 | 0.0518168063872256 |
| 1145 | -0.00771876923076923 | -0.874875608782435 | -0.158374251497006 | -0.0212790019960080 |
| 1146 | -0.00855323076923077 | -0.539771936127745 | -0.211571756487026 | -0.0500300199600798 |
| 1147 | -0.0158547692307692 | 0.230008143712575 | -0.00649740518962076 | 0.00763445109780439 |
| 1148 | -0.0492332307692308 | 0.475122754491018 | 0.159186427145709 | -0.0709841516966068 |
| 1149 | -0.103681846153846 | 0.0394717365269461 | 0.0588827345309381 | -0.141318562874252 |
| 1150 | -0.147491076923077 | -0.0601009980039920 | 0.0341113772455090 | 0.108993972055888 |
| 1151 | -0.134974153846154 | 0.288484790419162 | -0.0406087824351297 | 0.201094690618762 |
| 1152 | -0.0728067692307692 | 0.429316047904192 | 0.286291916167665 | -0.153338762475050 |
| 1153 | -0.0114738461538462 | -0.0709841516966068 | 0.134008982035928 | -0.176566986027944 |
| 1154 | 0.0219046153846154 | -0.946996806387226 | -0.283449301397206 | 0.256160199600798 |
| 1155 | 0.0267027692307692 | -0.204505828343313 | -0.183145608782435 | 0.178191337325349 |
| 1156 | 0.0423489230769231 | 1.03406203592814 | 0.217663073852295 | -0.263144910179641 |
| 1157 | 0.0657138461538462 | 0.327794091816367 | -0.0247713572854291 | -0.113054850299401 |
| 1158 | 0.0824030769230769 | -0.507934650698603 | -0.402839121756487 | 0.281175209580838 |
| 1159 | 0.0767704615384615 | 0.328118962075848 | 0.157155988023952 | 0.149277884231537 |
| 1160 | 0.0120996923076923 | 0.676217445109780 | 0.215632634730539 | -0.221074211576846 |
| 1161 | -0.0738498461538462 | -0.0146191616766467 | -0.150658582834331 | -0.123938003992016 |
| 1162 | -0.120162461538462 | -0.328606267465070 | -0.0174617764471058 | 0.235693373253493 |
| 1163 | -0.0945027692307692 | -0.442310858283433 | 0.288728443113772 | 0.166496007984032 |
| 1164 | -0.0179409230769231 | -0.428991177644711 | 0.0901514970059880 | -0.116953293413174 |
| 1165 | 0.0588295384615385 | 0.147166227544910 | -0.155125548902196 | -0.0384971257485030 |
| 1166 | 0.0984664615384615 | 0.319509900199601 | 0.00690349301397206 | 0.220586906187625 |
| 1167 | 0.102012923076923 | -0.458716806387226 | -0.00771566866267465 | 0.173643153692615 |
| 1168 | 0.117450461538462 | -0.159836167664671 | -0.0255835329341317 | -0.0558776846307385 |
| 1169 | 0.136017230769231 | 0.687912774451098 | 0.0438574850299401 | -0.0456442714570858 |
| 1170 | 0.137894769230769 | 0.0882022754491018 | 0.00243652694610778 | 0.196871377245509 |
| 1171 | 0.112860923076923 | -0.659161756487026 | -0.0101521956087824 | 0.0972986427145709 |
| 1172 | 0.0467298461538462 | -0.0656237924151697 | 0.156343812375250 | -0.178191337325349 |
| 1173 | -0.00208615384615385 | 0.500787504990020 | 0.207916966067864 | 0.0225784830339321 |
| 1174 | -0.00333784615384615 | -0.110943193612774 | -0.0125887225548902 | 0.149602754491018 |
| 1175 | 0.0388024615384615 | -0.269804750499002 | -0.163653393213573 | -0.0940499401197605 |
| 1176 | 0.0988836923076923 | 0.278738682634731 | 0.0462940119760479 | -0.118415209580838 |
| 1177 | 0.126420923076923 | 0.195084590818363 | 0.127917664670659 | 0.0929128942115768 |
| 1178 | 0.136851692307692 | 0.00763445109780439 | -0.0645679640718563 | -0.0289134530938124 |
| 1179 | 0.144361846153846 | 0.236343113772455 | -0.227815269461078 | -0.250799840319361 |
| 1180 | 0.148116923076923 | 0.132384630738523 | 0.0613192614770459 | -0.0113704590818363 |
| 1181 | 0.140815384615385 | -0.127186706586826 | 0.227815269461078 | 0.132222195608782 |
| 1182 | 0.0909563076923077 | -0.0118577644710579 | 0.0998976047904192 | -0.158536686626747 |
| 1183 | 0.00834461538461538 | -0.0566898602794411 | 0.112080239520958 | -0.198008423153693 |
| 1184 | -0.0755187692307692 | -0.230657884231537 | 0.136039421157685 | 0.120201996007984 |
| 1185 | -0.0967975384615385 | 0.114191896207585 | 0.150658582834331 | 0.122638522954092 |
| 1186 | -0.0342129230769231 | 0.282474690618762 | -0.0657862275449102 | -0.173805588822355 |
| 1187 | 0.0554916923076923 | -0.164871656686627 | 0.102334131736527 | -0.148465708582834 |

| | | | | |
|---|---|---|---|---|
| 1188 | 0.131844923076923 | -0.321946427145709 | -0.249744011976048 | 0.0601009980039920 |
| 1189 | 0.147491076923077 | 0.0614004790419162 | 0.321215469061876 | -0.0441823552894212 |
| 1190 | 0.123917538461538 | 0.238129900199601 | 0.136851596806387 | -0.0427204391217565 |
| 1191 | 0.101178461538462 | -0.0497051497005988 | -1.14394940119760 | 0.157724510978044 |
| 1192 | 0.0869926153846154 | -0.109481277445110 | -0.786592115768463 | -0.0103958483033932 |
| 1193 | 0.0703033846153846 | -0.0305378043912176 | 1.10171626746507 | -0.142130738522954 |
| 1194 | 0.0221132307692308 | -0.103471177644711 | 0.977453393213573 | 0.191673453093812 |
| 1195 | -0.0448523076923077 | -0.0682227544910180 | -2.00241906187625 | 0.268667704590818 |
| 1196 | -0.0951286153846154 | -0.0683851896207585 | 0.0255835329341317 | -0.186150658682635 |
| 1197 | -0.0751015384615385 | -0.115166506986028 | 1.93582065868263 | -0.255835329341317 |
| 1198 | -0.0106393846153846 | -0.00422331337325349 | 0.0422331337325349 | 0.239754251497006 |
| 1199 | 0.0496504615384615 | 0.145379441117764 | -1.66374181636727 | 0.241541037924152 |
| 1200 | 0.0798996923076923 | 0.0341113772455090 | -0.00243652694610778 | -0.287997485029940 |
| 1201 | 0.0811513846153846 | -0.177541596806387 | 1.46881966067864 | -0.233906586826347 |
| 1202 | 0.0788566153846154 | -0.0696846706586826 | -0.626999600798403 | 0.251936886227545 |
| 1203 | 0.0815686153846154 | 0.139531776447106 | -0.144161177644711 | 0.159998602794411 |
| 1204 | 0.0821944615384615 | 0.00633497005988024 | -0.00893393213572854 | -0.239266946107784 |
| 1205 | 0.0398455384615385 | -0.117765469061876 | -0.192891716566866 | -0.0950245508982036 |
| 1206 | -0.0506935384615385 | 0.0979483832335329 | 0.362636427145709 | 0.178353772455090 |
| 1207 | -0.130176000000000 | 0.214414371257485 | 0.703344111776447 | 0.00406087824351297 |
| 1208 | -0.138312000000000 | 0.0422331337325349 | -0.460503592814371 | -0.105745269461078 |
| 1209 | -0.0794824615384615 | 0.0194922155688623 | -1.33602894211577 | 0.0475934930139721 |
| 1210 | 0.00938769230769231 | 0.208891576846307 | 0.959585528942116 | 0.0542533333333333 |
| 1211 | 0.0769790769230769 | 0.0487305389221557 | 0.821109580838323 | -0.0360605988023952 |
| 1212 | 0.0978406153846154 | -0.191835888223553 | -1.05785878243513 | 0.0212790019960080 |
| 1213 | 0.0867840000000000 | -0.148303273453094 | -1.16872075848303 | 0.0521416766467066 |
| 1214 | 0.0894960000000000 | -0.131572455089820 | 0.836947005988024 | -0.0204668263473054 |
| 1215 | 0.119328000000000 | 0.00243652694610778 | 1.04445788423154 | 0.0246901397205589 |
| 1216 | 0.107019692307692 | 0.184688742514970 | -0.893799301397206 | 0.109643712574850 |
| 1217 | 0.0271200000000000 | 0.0909636726546906 | -0.472686227544910 | -0.0136445508982036 |
| 1218 | -0.0700947692307692 | 0.0436950499001996 | 0.638776147704591 | -0.0911261077844311 |
| 1219 | -0.123917538461538 | 0.0527914171656687 | 0.681415369261477 | 0.123938003992016 |
| 1220 | -0.113486769230769 | -0.129785668662675 | -0.309845009980040 | 0.169419840319361 |
| 1221 | -0.0513193846153846 | -0.154963113772455 | -0.188424750499002 | -0.105745269461078 |
| 1222 | 0.0283716923076923 | -0.00779688622754491 | 0.237561377245509 | -0.101846826347305 |
| 1223 | 0.0834461538461538 | 0.217175768463074 | -0.231876147704591 | 0.111268063872255 |
| 1224 | 0.0790652307692308 | 0.179165948103792 | -0.00487305389221557 | -0.0212790019960080 |
| 1225 | 0.0621673846153846 | -0.0901514970059880 | 0.0402026946107784 | -0.139694211576846 |
| 1226 | 0.0644621538461539 | 0.0103958483033932 | 0.00284261477045908 | 0.0579893413173653 |
| 1227 | 0.0552830769230769 | 0.00893393213572854 | -0.255835329341317 | 0.104608223552894 |
| 1228 | 0.0146030769230769 | -0.348098483033932 | 0.0548218562874252 | -0.0729333732534930 |
| 1229 | -0.0778135384615385 | -0.378636287425150 | 0.190455189620759 | -0.0196546506986028 |
| 1230 | -0.144570461538462 | 0.0428828742514970 | -0.195734331337325 | 0.175267504990020 |
| 1231 | -0.149785846153846 | 0.291408622754491 | -0.0308626746506986 | 0.0118577644710579 |

| | | | | |
|---|---|---|---|---|
| 1232 | -0.109731692307692 | 0.192648063872255 | 0.115328942115768 | -0.160485908183633 |
| 1233 | -0.0379680000000000 | 0.0123450698602794 | 0.0369539920159681 | 0.120526866267465 |
| 1234 | 0.00229476923076923 | 0.0196546506986028 | -0.326900698602794 | 0.282312255489022 |
| 1235 | 0.0125169230769231 | 0.147166227544910 | 0.0479183632734531 | -0.0422331337325349 |
| 1236 | 0.00834461538461538 | 0.0349235528942116 | 0.311469361277445 | -0.119714690618762 |
| 1237 | 0.00751015384615385 | -0.130272974051896 | -0.0548218562874252 | 0.219124990019960 |
| 1238 | 0.0300406153846154 | -0.158374251497006 | -0.175836027944112 | 0.124262874251497 |
| 1239 | 0.0394283076923077 | 0.0384971257485030 | 0.367509481037924 | -0.220424471057884 |
| 1240 | -0.00479815384615385 | 0.445884431137725 | 0.435732235528942 | -0.0350859880239521 |
| 1241 | -0.0744756923076923 | 0.447508782435130 | -0.205074351297405 | 0.233256846307385 |
| 1242 | -0.108897230769231 | -0.115491377245509 | -0.254210978043912 | -0.0596136926147705 |
| 1243 | -0.0932510769230769 | -0.312525189620759 | 0.139288123752495 | -0.329093572854291 |
| 1244 | -0.0377593846153846 | 0.143755089820359 | 0.308220658682635 | 0.0198170858283433 |
| 1245 | 0.0200270769230769 | 0.100060039920160 | -0.351265968063872 | 0.249662794411178 |
| 1246 | 0.0440178461538462 | -0.341438642714571 | -0.294413672654691 | -0.165034091816367 |
| 1247 | 0.0463126153846154 | -0.199470339321357 | 0.181927345309381 | -0.308464311377246 |
| 1248 | 0.0373421538461538 | 0.150252495009980 | 0.264769261477046 | 0.134658722554890 |
| 1249 | 0.0333784615384615 | 0.0828419161676647 | 0.0832480039920160 | 0.178841077844311 |
| 1250 | 0.0304578461538462 | -0.0727709381237525 | -0.121014171656687 | -0.214576806387226 |
| 1251 | -0.00166892307692308 | 0.0747201596806387 | 0.141318562874252 | -0.116953293413174 |
| 1252 | -0.0402627692307692 | -0.110293453093812 | 0.130760279441118 | 0.231957365269461 |
| 1253 | -0.0778135384615385 | -0.337052894211577 | 0.111674151696607 | 0.0345986826347305 |
| 1254 | -0.0740584615384615 | 0.0826794810379242 | -0.237155289421158 | -0.223998043912176 |
| 1255 | -0.0108480000000000 | 0.311388143712575 | -0.140100299401198 | 0.0597761277445110 |
| 1256 | 0.0565347692307692 | -0.0180302994011976 | 0.147409880239521 | 0.264931696606786 |
| 1257 | 0.101178461538462 | 0.00617253493013972 | 0.0418270459081836 | -0.0378473852295409 |
| 1258 | 0.100761230769231 | 0.338839680638723 | -0.0917758483033932 | -0.151714411177645 |
| 1259 | 0.0945027692307692 | 0.170069580838323 | -0.283855389221557 | 0.158374251497006 |
| 1260 | 0.0861581538461538 | -0.0453194011976048 | 0.118983732534930 | 0.146516487025948 |
| 1261 | 0.0561175384615385 | -0.00357357285429142 | 0.108425449101796 | -0.198820598802395 |
| 1262 | 0.0137686153846154 | -0.0846287025948104 | -0.0491366267465070 | -0.159836167664671 |
| 1263 | -0.0717636923076923 | -0.207104790419162 | -0.185988223552894 | 0.0906388023952096 |
| 1264 | -0.144987692307692 | 0.0334616367265469 | -0.0101521956087824 | -0.0167308183632735 |
| 1265 | -0.154375384615385 | 0.230657884231537 | 0.237155289421158 | -0.185663353293413 |
| 1266 | -0.103473230769231 | -0.0332992015968064 | -0.137663772455090 | -0.0623750898203593 |
| 1267 | -0.0377593846153846 | -0.0867403592814371 | -0.216038722554890 | 0.0839789620758483 |
| 1268 | -0.00625846153846154 | 0.0591263872255489 | -0.0268017964071856 | 0.0552279441117765 |
| 1269 | -0.00146030769230769 | -0.0620502195608782 | 0.351265968063872 | 0.0371976447105788 |
| 1270 | -0.0233649230769231 | -0.276464590818363 | 0.0560401197604790 | 0.0748825948103793 |
| 1271 | -0.0196098461538462 | -0.107207185628743 | -0.0832480039920160 | 0.0342738123752495 |
| 1272 | 0.00333784615384615 | 0.0722836327345309 | 0.282231037924152 | 0.0165683832335329 |
| 1273 | 0.00751015384615385 | -0.0402839121756487 | 0.333804191616766 | 0.0505173253493014 |
| 1274 | -0.0198184615384615 | 0.122476087824351 | -0.0247713572854291 | 0.0410960878243513 |
| 1275 | -0.0890787692307692 | 0.286373133732535 | -0.330961576846307 | -0.0891768862275449 |

| | | | | |
|---|---|---|---|---|
| 1276 | -0.130384615384615 | 0.0756947704590818 | 0.0869027944111777 | -0.0901514970059880 |
| 1277 | -0.131427692307692 | -0.0860906187624751 | 0.0276139720558882 | 0.182739520958084 |
| 1278 | -0.111400615384615 | -0.0279388423153693 | -0.194109980039920 | 0.121339041916168 |
| 1279 | -0.0880356923076923 | -0.0717963273453094 | -0.282231037924152 | -0.189236926147705 |
| 1280 | -0.0917907692307692 | -0.100709780439122 | 0.000406087824351297 | -0.0461315768463074 |
| 1281 | -0.109314461538462 | -0.00617253493013972 | 0.198576946107784 | 0.270129620758483 |
| 1282 | -0.111817846153846 | 0.0102334131736527 | -0.0795932135728543 | 0.0854408782435130 |
| 1283 | -0.0719723076923077 | 0.0375225149700599 | -0.0706592814371258 | -0.241865908183633 |
| 1284 | -0.0337956923076923 | 0.0536035928143713 | 0.0617253493013972 | -0.119877125748503 |
| 1285 | -0.0423489230769231 | -0.0129948103792415 | 0.239591816367265 | 0.0756947704590818 |
| 1286 | -0.0696775384615385 | -0.0802429540918164 | -0.0779688622754491 | -0.0482432335329341 |
| 1287 | -0.0972147692307692 | -0.143917524950100 | -0.195328243512974 | -0.196546506986028 |
| 1288 | -0.0949200000000000 | -0.182252215568862 | -0.219693512974052 | -0.0388219960079840 |
| 1289 | -0.0529883076923077 | -0.121339041916168 | -0.134821157684631 | 0.0875525349301397 |
| 1290 | -0.0196098461538462 | 0.0557152495009980 | 0.0134008982035928 | -0.0302129341317365 |
| 1291 | -0.0306664615384615 | 0.108181796407186 | -0.141318562874252 | -0.00454818363273453 |
| 1292 | -0.0709292307692308 | -0.0313499800399202 | -0.0483244510978044 | 0.131085149700599 |
| 1293 | -0.0907476923076923 | -0.0942123752495010 | -0.00446696606786427 | 0.110455888223553 |
| 1294 | -0.0778135384615385 | -0.261358123752495 | 0.205074351297405 | 0.0207916966067864 |
| 1295 | -0.0557003076923077 | 0.161135648702595 | 0.0962428143712575 | 0.00129948103792415 |
| 1296 | -0.0448523076923077 | 0.904763672654691 | -0.0377661676646707 | 0.0393093013972056 |
| 1297 | -0.0728067692307692 | 0.465863952095808 | -0.0215226546906188 | 0.0363854690618763 |
| 1298 | -0.0976320000000000 | -0.151551976047904 | 0.0194922155688623 | -0.0823546107784431 |
| 1299 | -0.0863667692307692 | -0.192972934131737 | 0.121014171656687 | -0.107044750499002 |
| 1300 | -0.0333784615384615 | -0.155775289421158 | -0.0454818363273453 | 0.122313652694611 |
| 1301 | 0.0527796923076923 | 0.0310251097804391 | 0.0994915169660679 | 0.132222195608782 |
| 1302 | 0.0863667692307692 | 0.00698471057884232 | -0.0735018962075848 | -0.131085149700599 |
| 1303 | 0.0761446153846154 | -0.687100598802395 | 0.356951197604790 | -0.0701719760479042 |
| 1304 | 0.0463126153846154 | -0.508421956087824 | 0.769130339321357 | 0.220911776447106 |
| 1305 | 0.0133513846153846 | 0.903139321357286 | -0.777658183632735 | 0.126049660678643 |
| 1306 | 0.0273286153846154 | 0.644055289421158 | -0.901921057884232 | -0.214739241516966 |
| 1307 | 0.0498590769230769 | -0.747201596806387 | 1.53298153692615 | -0.0893393213572854 |
| 1308 | 0.0436006153846154 | -0.280363033932136 | 0.901514970059880 | 0.225622395209581 |
| 1309 | 0.00500676923076923 | 0.665821596806387 | -2.32931976047904 | -0.00519792415169661 |
| 1310 | -0.0231563076923077 | -0.0376849500998004 | -0.117359381237525 | -0.282799560878244 |
| 1311 | 0.00208615384615385 | -0.563974770459082 | 2.51408972055888 | 0.112405109780439 |
| 1312 | 0.0531969230769231 | -0.0802429540918164 | -1.07166576846307 | 0.336890459081836 |
| 1313 | 0.0909563076923077 | 0.0271266666666667 | -2.15754461077844 | -0.128323752495010 |
| 1314 | 0.0888701538461539 | 0.0441823552894212 | 1.41643433133733 | -0.186637964071856 |
| 1315 | 0.0609156923076923 | 0.564624510978044 | 1.42942914171657 | 0.235855808383234 |
| 1316 | 0.0467298461538462 | 0.438574850299401 | -1.65765049900200 | 0.189561796407186 |
| 1317 | 0.0755187692307692 | -0.329905748502994 | -0.392280838323353 | -0.203368782435130 |
| 1318 | 0.119119384615385 | 0.0490554091816367 | 1.52039281437126 | -0.103146307385230 |
| 1319 | 0.128507076923077 | 0.590939001996008 | -0.669232734530938 | 0.211165668662675 |

| | | | | |
|---|---|---|---|---|
| 1320 | 0.0980492307692308 | -0.183551696606786 | -0.599791716566866 | -0.0204668263473054 |
| 1321 | 0.0496504615384615 | -0.631872654690619 | 1.08059970059880 | -0.255510459081836 |
| 1322 | 0.0400541538461538 | 0.201744431137725 | -0.398372155688623 | 0.0357357285429142 |
| 1323 | 0.0801083076923077 | 0.387082914171657 | -1.08222405189621 | 0.160485908183633 |
| 1324 | 0.141024000000000 | -0.359631377245509 | 1.19998952095808 | -0.132709500998004 |
| 1325 | 0.164806153846154 | -0.193947544910180 | 0.864154890219561 | -0.0550655089820359 |
| 1326 | 0.130384615384615 | 0.287672614770459 | -1.63206696606786 | 0.207754530938124 |
| 1327 | 0.0936683076923077 | -0.0735831137724551 | -0.294007584830339 | 0.0573396007984032 |
| 1328 | 0.0682172307692308 | -0.374412974051896 | 1.92607455089820 | -0.156425029940120 |
| 1329 | 0.0813600000000000 | -0.0445072255489022 | 0.100303692614770 | -0.0412585229540918 |
| 1330 | 0.103056000000000 | 0.0914509780439122 | -1.94313023952096 | 0.118577644710579 |
| 1331 | 0.0842806153846154 | -0.0946996806387226 | 0.103552395209581 | -0.00324870259481038 |
| 1332 | 0.0557003076923077 | -0.00129948103792415 | 1.67714271457086 | -0.102496566866267 |
| 1333 | 0.0327526153846154 | 0.246901397205589 | -0.399590419161677 | 0.0594512574850299 |
| 1334 | 0.0504849230769231 | 0.165358962075848 | -1.15085289421158 | 0.124425309381238 |
| 1335 | 0.0872012307692308 | 0.0276139720558882 | 0.600197804391218 | -0.0142942914171657 |
| 1336 | 0.105142153846154 | 0.0443447904191617 | 0.856033133732535 | 0.0722836327345309 |
| 1337 | 0.0861581538461538 | -0.0929128942115768 | -0.414209580838323 | 0.133359241516966 |
| 1338 | 0.0375507692307692 | -0.0862530538922156 | -0.319997205588822 | -0.123288263473054 |
| 1339 | 0.0342129230769231 | 0.188912055888224 | 0.447914870259481 | -0.159836167664671 |
| 1340 | 0.0600812307692308 | 0.147003792415170 | -0.0881210578842315 | 0.140506387225549 |
| 1341 | 0.100969846153846 | -0.139369341317365 | -0.283043213572854 | 0.0802429540918164 |
| 1342 | 0.117867692307692 | -0.0285885828343313 | 0.289540618762475 | -0.212140279441118 |
| 1343 | 0.0801083076923077 | 0.0268017964071856 | -0.125481137724551 | -0.0246901397205589 |
| 1344 | 0.0579950769230769 | -0.0774815568862275 | -0.496239321357285 | 0.210678363273453 |
| 1345 | 0.0661310769230769 | 0.0110455888223553 | 0.293195409181637 | 0.0115328942115768 |
| 1346 | 0.107436923076923 | -0.101359520958084 | 0.505173253493014 | -0.103471177644711 |
| 1347 | 0.146865230769231 | -0.166008702594810 | -0.276545808383234 | 0.142780479041916 |
| 1348 | 0.147491076923077 | -0.00162435129740519 | -0.343956387225549 | 0.0609131736526946 |
| 1349 | 0.110566153846154 | 0.0752074650698603 | 0.315124151696607 | -0.209703752495010 |
| 1350 | 0.0515280000000000 | -0.0134821157684631 | 0.222536127744511 | 0.0238779640718563 |
| 1351 | 0.0166892307692308 | -0.00730958083832335 | -0.357763373253493 | 0.149927624750499 |
| 1352 | 0.0204443076923077 | 0.129460798403194 | -0.241622255489022 | -0.213927065868264 |
| 1353 | 0.0323353846153846 | -0.00438574850299401 | 0.210759580838323 | -0.216688463073852 |
| 1354 | 0.0127255384615385 | -0.214251936127745 | 0.0946184630738523 | 0.220262035928144 |
| 1355 | -0.0241993846153846 | 0.0316748502994012 | -0.471874051896208 | 0.0932377644710579 |
| 1356 | -0.0369249230769231 | 0.152851457085828 | -0.0657862275449102 | -0.301317165668663 |
| 1357 | -0.0164806153846154 | -0.197196247504990 | 0.478777544910180 | -0.114191896207585 |
| 1358 | 0.0219046153846154 | -0.00438574850299401 | 0.0584766467065868 | 0.198170858283433 |
| 1359 | 0.0398455384615385 | 0.336565588822355 | -0.376849500998004 | 0.0300504990019960 |
| 1360 | 0.0152289230769231 | -0.0115328942115768 | 0.0170556886227545 | -0.132547065868263 |
| 1361 | -0.00688430769230769 | -0.298230898203593 | 0.495021057884232 | 0.0711465868263473 |
| 1362 | -0.0133513846153846 | 0.0332992015968064 | -0.118577644710579 | 0.0773191217564870 |
| 1363 | 0.00458953846153846 | 0.102496566866267 | -0.346799001996008 | -0.0628623952095808 |

| | | | | |
|---|---|---|---|---|
| 1364 | 0.0256596923076923 | -0.194109980039920 | 0.00690349301397206 | 0.0217663073852295 |
| 1365 | -0.0135600000000000 | 0.0198170858283433 | 0.219287425149701 | 0.0713090219560878 |
| 1366 | -0.0815686153846154 | -0.0203043912175649 | 0.0795932135728543 | -0.0601009980039920 |
| 1367 | -0.149994461538462 | -0.286373133732535 | -0.0722836327345309 | 0.0316748502994012 |
| 1368 | -0.171064615384615 | -0.148790578842315 | -0.0150252495009980 | 0.213602195608782 |
| 1369 | -0.138103384615385 | -0.113704590818363 | 0.0779688622754491 | 0.0261520558882236 |
| 1370 | -0.116616000000000 | 0.0565274251497006 | 0.319997205588822 | -0.125237485029940 |
| 1371 | -0.115155692307692 | 0.630735608782435 | 0.0491366267465070 | 0.0643243113772455 |
| 1372 | -0.141649846153846 | 0.650877564870260 | -0.250150099800399 | 0.137257684630739 |
| 1373 | -0.158964923076923 | -0.00357357285429142 | -0.201419560878244 | -0.118252774451098 |
| 1374 | -0.133096615384615 | -0.173318283433134 | 0.112486327345309 | -0.170719321357285 |
| 1375 | -0.0922080000000000 | -0.316098762475050 | -0.0247713572854291 | 0.0324870259481038 |
| 1376 | -0.0598726153846154 | -0.349397964071856 | -0.307408483033932 | 0.0404463473053892 |
| 1377 | -0.0700947692307692 | 0.353296407185629 | -0.118577644710579 | -0.0911261077844311 |
| 1378 | -0.110774769230769 | -0.176079680638723 | 0.0954306387225549 | -0.0630248303393214 |
| 1379 | -0.130384615384615 | -0.665171856287425 | 0.248525748502994 | 0.0609131736526946 |
| 1380 | -0.107436923076923 | 0.834429261477046 | -0.0588827345309381 | 0.0422331337325349 |
| 1381 | -0.0621673846153846 | 0.814937045908184 | -0.0357357285429142 | -0.0420706986027944 |
| 1382 | -0.0362990769230769 | -0.749800558882236 | 0.299692814371258 | -0.0259896207584830 |
| 1383 | -0.0408886153846154 | -0.427854131736527 | 0.163653393213573 | 0.0274515369261477 |
| 1384 | -0.0565347692307692 | 0.639019800399202 | -0.209541317365269 | 0.0342738123752495 |
| 1385 | -0.0563261538461538 | -0.0987605588822355 | -0.0670044910179641 | 0.0251774451097804 |
| 1386 | -0.0381766153846154 | -0.814774610778443 | 0.234312674650699 | -0.0513295009980040 |
| 1387 | -0.0271200000000000 | 0.205155568862275 | -0.144161177644711 | -0.0321621556886228 |
| 1388 | -0.0383852307692308 | 0.364991736526946 | -0.188018662674651 | 0.0994102994011976 |
| 1389 | -0.0696775384615385 | -0.246901397205589 | 0.0722836327345309 | 0.0685476247504990 |
| 1390 | -0.0886615384615385 | 0.343225429141717 | 0.124668962075848 | -0.0703344111776447 |
| 1391 | -0.0678000000000000 | 0.437600239520958 | 0.00162435129740519 | -0.0219287425149701 |
| 1392 | -0.0279544615384615 | -0.468138043912176 | -0.0722836327345309 | 0.137582554890220 |
| 1393 | 0.00125169230769231 | -0.0282637125748503 | -0.0284261477045908 | 0.121501477045908 |
| 1394 | -0.00104307692307692 | 0.774328263473054 | -0.0519792415169661 | 0.0222536127744511 |
| 1395 | -0.0112652307692308 | -0.155450419161677 | 0.151064670658683 | -0.0505173253493014 |
| 1396 | -0.00584123076923077 | -0.614004790419162 | -0.0487305389221557 | 0.0181927345309381 |
| 1397 | 0.0246166153846154 | 0.370027225548902 | -0.277764071856287 | 0.123775568862275 |
| 1398 | 0.0544486153846154 | 0.446371736526946 | 0.0913697604790419 | 0.0633497005988024 |
| 1399 | 0.0473556923076923 | -0.349235528942116 | 0.361824251497006 | -0.0717963273453094 |
| 1400 | 0.00458953846153846 | -0.239591816367265 | 0.0304565868263473 | -0.130435409181637 |
| 1401 | -0.0521538461538462 | 0.183226826347305 | -0.266393612774451 | 0.0510046307385230 |
| 1402 | -0.0694689230769231 | -0.0914509780439122 | 0.177866467065868 | 0.217663073852295 |
| 1403 | -0.0463126153846154 | -0.102171696606786 | 0.282637125748503 | 0.0142942914171657 |
| 1404 | -0.0116824615384615 | 0.131572455089820 | -0.0572583832335329 | -0.186475528942116 |
| 1405 | -0.00354646153846154 | -0.154313373253493 | -0.199389121756487 | 0.0445072255489022 |
| 1406 | -0.0241993846153846 | -0.160323473053892 | -0.0223348303393214 | 0.192160758483034 |
| 1407 | -0.0346301538461538 | 0.172993413173653 | 0.122232435129741 | -0.0896641916167665 |

| | | | | |
|---|---|---|---|---|
| 1408 | -0.0127255384615385 | 0.0318372854291417 | -0.202637824351297 | -0.199307904191617 |
| 1409 | 0.0323353846153846 | -0.0123450698602794 | -0.168526447105788 | 0.0412585229540918 |
| 1410 | 0.0465212307692308 | 0.154963113772455 | -0.0345174650698603 | 0.112567544910180 |
| 1411 | 0.0283716923076923 | -0.0958367265469062 | 0.0393905189620759 | -0.112567544910180 |
| 1412 | -0.0141858461538462 | -0.0943748103792415 | -0.0527914171656687 | -0.112567544910180 |
| 1413 | -0.0433920000000000 | 0.281500079840319 | -0.00527914171656687 | 0.103958483033932 |
| 1414 | -0.0196098461538462 | 0.252911497005988 | 0.102334131736527 | 0.0251774451097804 |
| 1415 | 0.0160633846153846 | -0.115816247504990 | -0.0556340319361277 | -0.117440598802395 |
| 1416 | 0.0425575384615385 | -0.139369341317365 | 0.578675149700599 | 0.0188424750499002 |
| 1417 | 0.0360904615384615 | 0.247713572854291 | -0.269642315369262 | 0.168282794411178 |
| 1418 | 0.0319181538461538 | 0.0513295009980040 | -1.22760349301397 | 0.0185176047904192 |
| 1419 | 0.0584123076923077 | -0.194434850299401 | 0.733394610778443 | -0.104283353293413 |
| 1420 | 0.0878270769230769 | 0.164221916167665 | 1.48546926147705 | 0.148303273453094 |
| 1421 | 0.103473230769231 | 0.147653532934132 | -1.41521606786427 | 0.220749341317365 |
| 1422 | 0.0765618461538462 | -0.163247305389222 | -1.29501407185629 | -0.0804053892215569 |
| 1423 | 0.0248252307692308 | -0.106557445109780 | 2.41500429141717 | -0.125399920159681 |
| 1424 | -0.0164806153846154 | 0.109643712574850 | 0.575020359281437 | 0.0459691417165669 |
| 1425 | -0.0123083076923077 | -0.277439201596806 | -2.09703752495010 | 0.0123450698602794 |
| 1426 | 0.0498590769230769 | -0.388057524950100 | 0.172181237524950 | -0.0648116167664671 |
| 1427 | 0.106185230769231 | 0.193947544910180 | 1.40059690618762 | -0.0656237924151697 |
| 1428 | 0.118076307692308 | 0.0591263872255489 | -0.536848103792415 | -0.0735831137724551 |
| 1429 | 0.104516307692308 | -0.374900279441118 | -0.957149001996008 | -0.0310251097804391 |
| 1430 | 0.0769790769230769 | -0.110455888223553 | 0.804866067864271 | 0.0985981237524950 |
| 1431 | 0.0782307692307692 | 0.270779361277445 | -0.317154590818363 | 0.0480807984031936 |
| 1432 | 0.102638769230769 | -0.0633497005988024 | -0.641618762475050 | -0.234718762475050 |
| 1433 | 0.110774769230769 | -0.291733493013972 | 1.20242604790419 | -0.120689301397206 |
| 1434 | 0.0940855384615385 | 0.202069301397206 | 0.157968163672655 | 0.221561516966068 |
| 1435 | 0.0596640000000000 | 0.326494610778443 | -1.19389820359281 | 0.0266393612774451 |
| 1436 | 0.0573692307692308 | -0.165196526946108 | 0.407712175648703 | -0.248363313373253 |
| 1437 | 0.0874098461538462 | -0.0748825948103793 | 1.37501337325349 | 0.0144567265469062 |
| 1438 | 0.126838153846154 | 0.366778522954092 | -0.560807285429142 | 0.228546227544910 |
| 1439 | 0.145822153846154 | 0.0388219960079840 | -1.13136067864271 | -0.0316748502994012 |
| 1440 | 0.116198769230769 | -0.0575020359281437 | 0.451975748502994 | -0.128323752495010 |
| 1441 | 0.0640449230769231 | 0.195734331337325 | 0.890550598802395 | 0.121339041916168 |
| 1442 | 0.0438092307692308 | -0.147978403193613 | -0.557964670658683 | 0.0615629141716567 |
| 1443 | 0.0602898461538462 | -0.119877125748503 | -0.893393213572854 | -0.155450419161677 |
| 1444 | 0.0630018461538462 | 0.187775009980040 | 0.571771656686627 | 0.0527914171656687 |
| 1445 | 0.0325440000000000 | 0.0185176047904192 | 0.595324750499002 | 0.159511297405190 |
| 1446 | -0.00980492307692308 | -0.283449301397206 | -0.387813872255489 | -0.156587465069860 |
| 1447 | -0.0204443076923077 | -0.232931976047904 | -0.218475249500998 | -0.108993972055888 |
| 1448 | 0.0156461538461538 | 0.0589639520958084 | 0.370758183632735 | 0.201094690618762 |
| 1449 | 0.0807341538461538 | -0.0230657884231537 | 0.0556340319361277 | -0.00373600798403194 |
| 1450 | 0.135391384615385 | -0.167308183632735 | -0.339083333333333 | -0.331367664670659 |
| 1451 | 0.144153230769231 | -0.0709841516966068 | 0.0661923153692615 | -0.0154313373253493 |

| | | | | |
|---|---|---|---|---|
| 1452 | 0.112860923076923 | 0.177054291417166 | 0.103552395209581 | 0.290434011976048 |
| 1453 | 0.0863667692307692 | 0.506472734530938 | -0.360605988023952 | -0.108181796407186 |
| 1454 | 0.0882443076923077 | 0.391306227544910 | -0.0288322355289421 | -0.255348023952096 |
| 1455 | 0.0999267692307692 | -0.161622954091816 | 0.322839820359281 | 0.187450139720559 |
| 1456 | 0.0922080000000000 | -0.127674011976048 | -0.0621314371257485 | 0.217500638722555 |
| 1457 | 0.0550744615384615 | 0.305053173652695 | -0.0962428143712575 | -0.207267225548902 |
| 1458 | 0.0112652307692308 | 0.136445508982036 | 0.376037325349301 | -0.0969737724550898 |
| 1459 | -0.00250338461538462 | -0.349885269461078 | 0.235937025948104 | 0.268992574850299 |
| 1460 | 0.0308750769230769 | -0.0357357285429142 | -0.245683133732535 | 0.0519792415169661 |
| 1461 | 0.0761446153846154 | 0.376199760479042 | -0.143755089820359 | -0.185500918163673 |
| 1462 | 0.0955458461538462 | 0.00568522954091816 | 0.172993413173653 | 0.133359241516966 |
| 1463 | 0.0715550769230769 | -0.177541596806387 | -0.00527914171656687 | 0.291408622754491 |
| 1464 | 0.0515280000000000 | -0.101846826347305 | -0.352078143712575 | -0.127998882235529 |
| 1465 | 0.0561175384615385 | -0.207267225548902 | -0.108425449101796 | -0.172830978043912 |
| 1466 | 0.0657138461538462 | -0.540908982035928 | 0.300504990019960 | 0.214576806387226 |
| 1467 | 0.0878270769230769 | -0.285723393213573 | 0.0901514970059880 | 0.112405109780439 |
| 1468 | 0.0625846153846154 | 0.324707824351297 | -0.298880638722555 | -0.185176047904192 |
| 1469 | 0.0237821538461538 | 0.272078842315369 | 0.0406087824351297 | -0.0139694211576846 |
| 1470 | 0.0248252307692308 | 0.0766693812375250 | 0.389438223552894 | 0.153338762475050 |
| 1471 | 0.0515280000000000 | 0.175592375249501 | -0.00893393213572854 | -0.0326494610778443 |
| 1472 | 0.109940307692308 | 0.362067904191617 | -0.387407784431138 | 0.00812175648702595 |
| 1473 | 0.131427692307692 | 0.229033532934132 | 0.00324870259481038 | 0.144892135728543 |
| 1474 | 0.106602461538462 | -0.103146307385230 | 0.214820459081836 | -0.0804053892215569 |
| 1475 | 0.0700947692307692 | -0.169419840319361 | -0.193297804391218 | -0.189886666666667 |
| 1476 | 0.0385938461538462 | -0.0636745708582834 | -0.147815968063872 | 0.105257964071856 |
| 1477 | 0.0369249230769231 | 0.148953013972056 | 0.129948103792415 | 0.201906866267465 |
| 1478 | 0.0229476923076923 | 0.368078003992016 | 0.176648203592814 | -0.152851457085828 |
| 1479 | 0.00646707692307692 | 0.132709500998004 | 0.111268063872255 | -0.167145748502994 |
| 1480 | -0.0183581538461538 | -0.0958367265469062 | 0.0544157684630739 | 0.291246187624751 |
| 1481 | -0.0394283076923077 | 0.0414209580838323 | 0.0580705588822355 | 0.252911497005988 |
| 1482 | -0.00292061538461538 | 0.0807302594810379 | 0.109643712574850 | -0.157237205588822 |
| 1483 | 0.0548658461538462 | -0.187287704590818 | 0.143755089820359 | -0.106882315369261 |
| 1484 | 0.117867692307692 | -0.520279720558882 | -0.0751262475049900 | 0.151389540918164 |
| 1485 | 0.125377846153846 | -0.324870259481038 | -0.312687624750499 | 0.106070139720559 |
| 1486 | 0.0659224615384615 | 0.0472686227544910 | -0.0605070858283433 | -0.129785668662675 |
| 1487 | 0.0166892307692308 | 0.0826794810379242 | 0.187612574850299 | -0.138394730538922 |
| 1488 | -0.0123083076923077 | 0.0963240319361277 | -0.0954306387225549 | 0.0531162874251497 |
| 1489 | -0.00897046153846154 | 0.0839789620758483 | -0.248119660678643 | 0.0332992015968064 |
| 1490 | -0.00438092307692308 | 0.00990854291417166 | 0.0341113772455090 | -0.0727709381237525 |
| 1491 | -0.0202356923076923 | 0.0804053892215569 | 0.257053592814371 | -0.0209541317365269 |
| 1492 | -0.0404713846153846 | 0.0940499401197605 | 0.0556340319361277 | 0.0219287425149701 |
| 1493 | -0.0584123076923077 | -0.0724460678642715 | -0.300098902195609 | -0.0506797604790419 |
| 1494 | -0.0463126153846154 | -0.0930753293413174 | -0.0816236526946108 | -0.0300504990019960 |
| 1495 | -0.0285803076923077 | 0.0773191217564870 | 0.281418862275449 | 0.0997351696606787 |

| | | | | |
|---|---|---|---|---|
| 1496 | -0.0410972307692308 | 0.106232574850299 | -0.0328931137724551 | 0.0295631936127745 |
| 1497 | -0.0853236923076923 | 0.0323245908183633 | -0.276545808383234 | -0.115166506986028 |
| 1498 | -0.123083076923077 | -0.0110455888223553 | 0.0633497005988024 | -0.0220911776447106 |
| 1499 | -0.117867692307692 | -0.129460798403194 | 0.273297105788423 | 0.0888520159680639 |
| 1500 | -0.0759360000000000 | -0.161135648702595 | -0.00852784431137725 | -0.0316748502994012 |
| 1501 | -0.0335870769230769 | -0.00389844311377246 | -0.115328942115768 | -0.138394730538922 |
| 1502 | -0.0275372307692308 | 0.0203043912175649 | 0.119389820359281 | -0.00324870259481038 |
| 1503 | -0.0440178461538462 | -0.121014171656687 | 0.116141117764471 | 0.110618323353293 |
| 1504 | -0.0500676923076923 | 0.0147815968063872 | -0.0893393213572854 | 0.000974610778443114 |
| 1505 | -0.0214873846153846 | 0.191835888223553 | -0.140912475049900 | -0.0300504990019960 |
| 1506 | 0.0294147692307692 | 0.0194922155688623 | -0.0154313373253493 | 0.0568522954091816 |
| 1507 | 0.0406800000000000 | -0.102496566866267 | -0.0377661676646707 | -0.0159186427145709 |
| 1508 | -0.000208615384615385 | 0.0181927345309381 | -0.132790718562874 | -0.0555528143712575 |
| 1509 | -0.0579950769230769 | -0.0445072255489022 | -0.148222055888224 | 0.0142942914171657 |
| 1510 | -0.117450461538462 | -0.0336240718562874 | -0.0381722554890220 | -0.0691973652694611 |
| 1511 | -0.128715692307692 | 0.208404271457086 | 0.149034231536926 | -0.137095249500998 |
| 1512 | -0.0990923076923077 | 0.0319997205588822 | 0.112080239520958 | 0.0201419560878244 |
| 1513 | -0.0725981538461538 | -0.160648343313373 | -0.0377661676646707 | 0.0898266267465070 |
| 1514 | -0.0569520000000000 | 0.137744990019960 | -0.00934001996007984 | -0.00682227544910180 |
| 1515 | -0.0638363076923077 | 0.227571616766467 | 0.241622255489022 | -0.00341113772455090 |
| 1516 | -0.0465212307692308 | -0.0688724950099800 | 0.129948103792415 | 0.0838165269461078 |
| 1517 | 0.00542400000000000 | -0.164059481037924 | -0.180302994011976 | 0.0224160479041916 |
| 1518 | 0.0467298461538462 | 0.133359241516966 | -0.127105489021956 | -0.0813800000000000 |
| 1519 | 0.0602898461538462 | 0.537660279441118 | 0.149440319361277 | 0.127998882235529 |
| 1520 | 0.0321267692307692 | -0.133521676646707 | 0.0198983033932136 | 0.112080239520958 |
| 1521 | -0.0112652307692308 | -0.968763113772455 | -0.0714714570858283 | -0.258271856287425 |
| 1522 | -0.0300406153846154 | -0.278901117764471 | 0.0259896207584830 | -0.0997351696606787 |
| 1523 | -0.0146030769230769 | 0.381072814371258 | 0.0503548902195609 | 0.252586626746507 |
| 1524 | 0.00834461538461538 | 0.270941796407186 | 0.0747201596806387 | -0.0123450698602794 |
| 1525 | -0.00229476923076923 | 0.267043353293413 | -0.00487305389221557 | -0.290921317365269 |
| 1526 | -0.0348387692307692 | -0.0695222355289421 | 0.145785528942116 | 0.0724460678642715 |
| 1527 | -0.0596640000000000 | -0.252749061876248 | -0.349235528942116 | 0.230333013972056 |
| 1528 | -0.0529883076923077 | 0.736643313373254 | 0.389844311377246 | -0.159348862275449 |
| 1529 | -0.00938769230769231 | 0.387245349301397 | 0.324870259481038 | -0.0579893413173653 |
| 1530 | 0.00438092307692308 | -1.18675105788423 | -1.01846826347305 | 0.369377485029940 |
| 1531 | -0.0379680000000000 | -0.402514251497006 | -1.00872215568862 | 0.0929128942115768 |
| 1532 | -0.0855323076923077 | 1.13818295409182 | 1.12933023952096 | -0.254535848303393 |
| 1533 | -0.110774769230769 | 0.273865628742515 | 1.36648552894212 | 0.167308183632735 |
| 1534 | -0.0909563076923077 | -0.785698722554890 | -2.08607315369261 | 0.353133972055888 |
| 1535 | -0.0423489230769231 | 0.290921317365269 | -0.192485628742515 | -0.168932534930140 |
| 1536 | 0.00208615384615385 | 0.700420279441118 | 2.14982894211577 | -0.206455049900200 |
| 1537 | 0.0146030769230769 | -0.244140000000000 | 0.310657185628743 | 0.309276487025948 |
| 1538 | -0.000208615384615385 | -0.255023153692615 | -2.05886526946108 | 0.234393892215569 |
| 1539 | 0.00166892307692308 | -0.0245277045908184 | 0.00446696606786427 | -0.184526307385230 |

| | | | | |
|---|---|---|---|---|
| 1540 | 0.0202356923076923 | -0.624238203592814 | 1.96465289421158 | -0.0664359680638723 |
| 1541 | 0.0365076923076923 | -0.330555489021956 | -1.04039700598802 | 0.0685476247504990 |
| 1542 | 0.00417230769230769 | 0.563000159680639 | -0.585984730538922 | -0.103958483033932 |
| 1543 | -0.0665483076923077 | -0.135308463073852 | 0.406900000000000 | -0.0656237924151697 |
| 1544 | -0.112235076923077 | -0.356382674650699 | 0.190861277445110 | -0.00357357285429142 |
| 1545 | -0.126629538461538 | 0.592076047904192 | -0.147409880239521 | -0.146678922155689 |
| 1546 | -0.0955458461538462 | 0.267043353293413 | 0.635933532934132 | -0.0753699001996008 |
| 1547 | -0.0413058461538462 | -0.472523792415170 | 0.166089920159681 | 0.147003792415170 |
| 1548 | -0.0158547692307692 | -0.00406087824351297 | -1.16100508982036 | 0.0141318562874252 |
| 1549 | -0.00625846153846154 | 0.350047704590818 | 1.01278303393214 | -0.198170858283433 |
| 1550 | 0.00792738461538461 | -0.168120359281437 | 0.826794810379242 | 0.0440199201596806 |
| 1551 | 0.0398455384615385 | -0.265094131736527 | -1.03755439121756 | 0.270941796407186 |
| 1552 | 0.0604984615384615 | 0.219124990019960 | -1.22394870259481 | -0.0841413972055888 |
| 1553 | 0.0258683076923077 | 0.306190219560878 | 1.03349351297405 | -0.259733772455090 |
| 1554 | -0.0315009230769231 | 0.00211165668662675 | 1.02577784431138 | 0.126861836327345 |
| 1555 | -0.0888701538461539 | 0.0818673053892216 | -1.44932744510978 | 0.197196247504990 |
| 1556 | -0.112860923076923 | 0.116628423153693 | -0.514513273453094 | -0.102334131736527 |
| 1557 | -0.0728067692307692 | -0.0648116167664671 | 1.03389960079840 | -0.0417458283433134 |
| 1558 | 0.000208615384615385 | -0.0196546506986028 | 0.797556487025948 | 0.164384351297405 |
| 1559 | 0.0598726153846154 | -0.273540758483034 | -0.684257984031936 | 0.00211165668662675 |
| 1560 | 0.0849064615384615 | -0.407874610778443 | -0.136851596806387 | -0.135308463073852 |
| 1561 | 0.0984664615384615 | 0.162110259481038 | 0.634309181636727 | 0.0367103393213573 |
| 1562 | 0.113904000000000 | 0.268667704590818 | -0.144973353293413 | 0.0573396007984032 |
| 1563 | 0.142275692307692 | -0.241216167664671 | -0.171775149700599 | -0.139856646706587 |
| 1564 | 0.159590769230769 | -0.139856646706587 | -0.229033532934132 | -0.0341113772455090 |
| 1565 | 0.123917538461538 | 0.236180678642715 | 0.0150252495009980 | 0.127998882235529 |
| 1566 | 0.0509021538461538 | 0.132709500998004 | -0.178678642714571 | -0.0497051497005988 |
| 1567 | -0.0187753846153846 | -0.00990854291417166 | 0.105582834331337 | -0.0886895808383234 |
| 1568 | -0.0277458461538462 | 0.101684391217565 | 0.120608083832335 | 0.0862530538922156 |
| 1569 | 0.0112652307692308 | -0.0404463473053892 | -0.298880638722555 | 0.0566898602794411 |
| 1570 | 0.0607070769230769 | -0.223510738522954 | 0.183145608782435 | -0.0425580039920160 |
| 1571 | 0.0984664615384615 | 0.0597761277445110 | 0.348829441117764 | 0.0729333732534930 |
| 1572 | 0.0965889230769231 | 0.119552255489022 | 0.0625375249500998 | 0.118740079840319 |
| 1573 | 0.101387076923077 | -0.139856646706587 | -0.513295009980040 | -0.0402839121756487 |
| 1574 | 0.128715692307692 | -0.0605883033932136 | -0.0942123752495010 | 0.0311875449101796 |
| 1575 | 0.148951384615385 | 0.163897045908184 | 0.280200598802395 | 0.159998602794411 |
| 1576 | 0.139980923076923 | 0.0240403992015968 | -0.138069860279441 | -0.0298880638722555 |
| 1577 | 0.0794824615384615 | -0.147815968063872 | -0.438168762475050 | -0.209703752495010 |
| 1578 | 0.0168978461538462 | 0.0713090219560878 | 0.170962974051896 | -0.0129948103792415 |
| 1579 | -0.000417230769230769 | 0.0901514970059880 | 0.549030738522954 | 0.169094970059880 |
| 1580 | 0.00834461538461538 | -0.180302994011976 | -0.185582135728543 | -0.110131017964072 |
| 1581 | 0.0246166153846154 | -0.0987605588822355 | -0.214008283433134 | -0.220262035928144 |
| 1582 | 0.0277458461538462 | 0.179328383233533 | 0.350453792415170 | 0.0349235528942116 |
| 1583 | 0.0198184615384615 | 0.0670857085828343 | 0.414209580838323 | 0.0792683433133733 |

| | | | | |
|---|---|---|---|---|
| 1584 | 0.0321267692307692 | -0.0929128942115768 | -0.405681736526946 | -0.112567544910180 |
| 1585 | 0.0765618461538462 | 0.115166506986028 | -0.306190219560878 | -0.104120918163673 |
| 1586 | 0.125795076923077 | 0.292058363273453 | 0.194109980039920 | 0.0828419161676647 |
| 1587 | 0.125586461538462 | 0.104933093812375 | 0.0162435129740519 | 0.0298880638722555 |
| 1588 | 0.0607070769230769 | -0.179328383233533 | -0.224566566866267 | -0.0529538522954092 |
| 1589 | -0.0323353846153846 | -0.107369620758483 | -0.188018662674651 | 0.0103958483033932 |
| 1590 | -0.108271384615385 | 0.0396341716566866 | 0.142536826347305 | 0.0659486626746507 |
| 1591 | -0.118910769230769 | 0.0579893413173653 | 0.0596949101796407 | 0.0748825948103793 |
| 1592 | -0.0638363076923077 | 0.141480998003992 | 0.132384630738523 | 0.0423955688622755 |
| 1593 | -0.00229476923076923 | 0.0355732934131737 | -0.0901514970059880 | 0.00259896207584830 |
| 1594 | 0.0431833846153846 | 0.0334616367265469 | -0.171775149700599 | 0.0185176047904192 |
| 1595 | 0.0680086153846154 | 0.309926227544910 | 0.102334131736527 | 0.0575020359281437 |
| 1596 | 0.0728067692307692 | -0.194597285429142 | 0.172587325349301 | 0.0157562075848303 |
| 1597 | 0.0715550769230769 | -0.755648223552894 | -0.219287425149701 | 0.0245277045908184 |
| 1598 | 0.0471470769230769 | -0.189236926147705 | -0.464564471057884 | 0.0614004790419162 |
| 1599 | -0.00125169230769231 | 0.405275648702595 | 0.0869027944111777 | 0.0409336526946108 |
| 1600 | -0.0713464615384615 | 0.168282794411178 | 0.207510878243513 | -0.0287510179640719 |
| 1601 | -0.141649846153846 | -0.198495728542914 | -0.0121826347305389 | -0.00129948103792415 |
| 1602 | -0.157921846153846 | 0.0203043912175649 | -0.171369061876248 | 0.106070139720559 |
| 1603 | -0.109523076923077 | 0.232931976047904 | 0.169338622754491 | -0.0337865069860280 |
| 1604 | -0.0362990769230769 | 0.000324870259481038 | 0.354920758483034 | -0.112080239520958 |
| 1605 | 0.00876184615384615 | -0.392768143712575 | -0.0873088822355290 | 0.0826794810379242 |
| 1606 | 0.0156461538461538 | -0.398290938123753 | -0.308220658682635 | 0.110780758483034 |
| 1607 | 0.0158547692307692 | 0.321946427145709 | 0.0767505988023952 | -0.0958367265469062 |
| 1608 | 0.0304578461538462 | 0.692460958083832 | 0.248525748502994 | -0.0698471057884232 |
| 1609 | 0.0458953846153846 | 0.0964864670658683 | -0.287916267465070 | 0.132059760479042 |
| 1610 | 0.0406800000000000 | -0.198983033932136 | -0.101115868263473 | 0.0448320958083832 |
| 1611 | -0.0150203076923077 | 0.256160199600798 | 0.387001696606786 | -0.118902514970060 |
| 1612 | -0.108688615384615 | 0.425092734530938 | 0.209135229540918 | 0.0155937724550898 |
| 1613 | -0.160633846153846 | -0.0102334131736527 | -0.106395009980040 | 0.119877125748503 |
| 1614 | -0.141858461538462 | -0.295631936127745 | 0.0345174650698603 | -0.0768318163672655 |
| 1615 | -0.0807341538461538 | -0.129623233532934 | 0.221723952095808 | -0.0943748103792415 |
| 1616 | -0.0216960000000000 | 0.119552255489022 | 0.0357357285429142 | 0.153176327345309 |
| 1617 | 0.00208615384615385 | 0.305702914171657 | -0.0844662674650699 | 0.158211816367265 |
| 1618 | 0.00959630769230769 | 0.000649740518962076 | -0.185582135728543 | -0.100709780439122 |
| 1619 | 0.0300406153846154 | -0.548380998003992 | -0.0925880239520958 | -0.136283073852295 |
| 1620 | 0.0682172307692308 | -0.163897045908184 | 0.0901514970059880 | 0.106719880239521 |
| 1621 | 0.0838633846153846 | 0.338189940119761 | 0.0657862275449102 | 0.101521956087824 |
| 1622 | 0.0544486153846154 | -0.170394451097804 | -0.0970549900199601 | -0.0779688622754491 |
| 1623 | -0.00876184615384615 | -0.518655369261477 | -0.0219287425149701 | -0.0402839121756487 |
| 1624 | -0.0688430769230769 | 0.00617253493013972 | 0.368321656686627 | 0.0302129341317365 |
| 1625 | -0.0826116923076923 | 0.283124431137725 | 0.263144910179641 | 0.0243652694610778 |
| 1626 | -0.0536141538461538 | -0.0558776846307385 | -0.169744710578842 | -0.000162435129740519 |
| 1627 | -0.00897046153846154 | -0.0337865069860280 | -0.201013473053892 | -0.0553903792415170 |

| | | | | |
|---|---|---|---|---|
| 1628 | 0.00792738461538461 | 0.0458067065868264 | 0.207104790419162 | -0.0386595608782435 |
| 1629 | 0.0116824615384615 | -0.165358962075848 | 0.157968163672655 | -0.0295631936127745 |
| 1630 | 0.0317095384615385 | 0.0185176047904192 | -0.296038023952096 | -0.0324870259481038 |
| 1631 | 0.0536141538461538 | 0.330393053892216 | -0.138882035928144 | 0.0510046307385230 |
| 1632 | 0.0713464615384615 | 0.174130459081836 | 0.166089920159681 | 0.0212790019960080 |
| 1633 | 0.0442264615384615 | -0.0769942514970060 | 0.130760279441118 | -0.0243652694610778 |
| 1634 | -0.0333784615384615 | 0.0696846706586826 | -0.0938062874251497 | 0.0607507385229541 |
| 1635 | -0.106811076923077 | 0.127349141716567 | -0.0726897205588822 | 0.0654613572854292 |
| 1636 | -0.138729230769231 | -0.0886895808383234 | 0.195734331337325 | 0.00211165668662675 |
| 1637 | -0.102847384615385 | -0.0615629141716567 | -0.109643712574850 | -0.0466188822355289 |
| 1638 | -0.0444350769230769 | -0.00958367265469062 | -0.0954306387225549 | -0.0407712175648703 |
| 1639 | -0.00250338461538462 | -0.224810219560878 | -0.0629436127744511 | 0.0484056686626747 |
| 1640 | 0.0196098461538462 | -0.0870652295409182 | -0.117765469061876 | 0.00990854291417166 |
| 1641 | 0.0125169230769231 | 0.220262035928144 | 0.432483532934132 | -0.0841413972055888 |
| 1642 | 0.0133513846153846 | 0.0500300199600798 | 0.0170556886227545 | -0.00438574850299401 |
| 1643 | 0.0325440000000000 | -0.108669101796407 | -1.23125828343313 | 0.0570147305389222 |
| 1644 | 0.0319181538461538 | -0.0375225149700599 | -0.731364171656687 | -0.0307002395209581 |
| 1645 | -0.0252424615384615 | -0.0755323353293413 | 2.23388912175649 | -0.0942123752495010 |
| 1646 | -0.112235076923077 | -0.145379441117764 | 0.610350000000000 | -0.0138069860279441 |
| 1647 | -0.154166769230769 | -0.108019361277445 | -2.55835329341317 | -0.00860906187624751 |
| 1648 | -0.121622769230769 | -0.0539284630738523 | 0.551873353293413 | -0.109968582834331 |
| 1649 | -0.0425575384615385 | 0.0511670658682635 | 2.73256497005988 | -0.0514919361277445 |
| 1650 | 0.0394283076923077 | 0.156100159680639 | -0.746795508982036 | 0.0531162874251497 |
| 1651 | 0.0869926153846154 | 0.218312814371257 | -2.78738682634731 | -0.00747201596806387 |
| 1652 | 0.0769790769230769 | 0.116628423153693 | 1.06232574850299 | -0.0992478642714571 |
| 1653 | 0.0579950769230769 | -0.113054850299401 | 1.57521467065868 | 0.0227409181636727 |
| 1654 | 0.0665483076923077 | -0.0250150099800399 | -1.17643642714571 | 0.182414650698603 |
| 1655 | 0.0661310769230769 | 0.0479183632734531 | -0.423549600798403 | 0.00942123752495010 |
| 1656 | 0.0398455384615385 | -0.0713090219560878 | 0.513701097804391 | -0.209703752495010 |
| 1657 | -0.0304578461538462 | 0.0344362475049900 | -0.162435129740519 | 0.0422331337325349 |
| 1658 | -0.0815686153846154 | 0.0963240319361277 | 0.0730958083832335 | 0.246414091816367 |
| 1659 | -0.0738498461538462 | 0.0665984031936128 | 1.01197085828343 | -0.0659486626746507 |
| 1660 | -0.0369249230769231 | 0.0800805189620758 | -0.972580339321357 | -0.187287704590818 |
| 1661 | 0.0302492307692308 | -0.0243652694610778 | -1.14313722554890 | 0.0750450299401198 |
| 1662 | 0.0584123076923077 | -0.0675730139720559 | 1.39003862275449 | 0.132547065868263 |
| 1663 | 0.0569520000000000 | -0.0454818363273453 | 0.699689321357286 | -0.0755323353293413 |
| 1664 | 0.0571606153846154 | -0.0992478642714571 | -1.50171277445110 | -0.0207916966067864 |
| 1665 | 0.0665483076923077 | -0.182901956087824 | -0.895829740518962 | 0.202069301397206 |
| 1666 | 0.0878270769230769 | -0.00129948103792415 | 1.72100019960080 | 0.0289134530938124 |
| 1667 | 0.0646707692307692 | 0.245277045908184 | 0.745983333333333 | -0.127674011976048 |
| 1668 | 0.00250338461538462 | 0.0823546107784431 | -1.46557095808383 | 0.154963113772455 |
| 1669 | -0.0751015384615385 | -0.0641618762475050 | -0.430453093812375 | 0.197845988023952 |
| 1670 | -0.119745230769231 | -0.164546786427146 | 1.26577574850299 | -0.202719041916168 |
| 1671 | -0.0990923076923077 | 0.131897325349301 | 0.496645409181637 | -0.0820297405189621 |

| | | | | |
|---|---|---|---|---|
| 1672 | -0.0369249230769231 | 0.584441596806387 | -1.02699610778443 | 0.376524630738523 |
| 1673 | 0.0271200000000000 | 0.231632495009980 | -0.0905575848303393 | 0.0859281836327345 |
| 1674 | 0.0490246153846154 | 0.00389844311377246 | 0.671669261477046 | -0.337540199600798 |
| 1675 | 0.0279544615384615 | -0.0272891017964072 | -0.0491366267465070 | 0.0648116167664671 |
| 1676 | 0.00751015384615385 | -0.116790858283433 | -0.417458283433134 | 0.369539920159681 |
| 1677 | 0.0152289230769231 | 0.190536407185629 | 0.274109281437126 | -0.138719600798403 |
| 1678 | 0.0248252307692308 | 0.0144567265469062 | 0.372788622754491 | -0.319672335329341 |
| 1679 | 0.00855323076923077 | -0.775140439121757 | -0.295631936127745 | 0.150902235528942 |
| 1680 | -0.0296233846153846 | -0.431427704590818 | 0.148628143712575 | 0.190698842315369 |
| 1681 | -0.0619587692307692 | 0.721374411177645 | 0.321215469061876 | -0.271429101796407 |
| 1682 | -0.0600812307692308 | 0.367103393213573 | -0.184769960079840 | -0.249500359281437 |
| 1683 | -0.0152289230769231 | -0.497701237524950 | -0.171775149700599 | 0.160810778443114 |
| 1684 | 0.0446436923076923 | -0.0501924550898204 | 0.360605988023952 | 0.0662735329341317 |
| 1685 | 0.0734326153846154 | 0.380423073852295 | -0.0649740518962076 | -0.154150938123753 |
| 1686 | 0.0638363076923077 | -0.0717963273453094 | -0.621314371257485 | 0.0376849500998004 |
| 1687 | 0.0419316923076923 | -0.311875449101796 | -0.00365479041916168 | 0.157074770459082 |
| 1688 | 0.0383852307692308 | -0.175267504990020 | 0.340707684630739 | 0.0235530938123753 |
| 1689 | 0.0490246153846154 | -0.220749341317365 | -0.179896906187625 | 0.0146191616766467 |
| 1690 | 0.0433920000000000 | 0.00958367265469062 | -0.422737425149701 | 0.0136445508982036 |
| 1691 | -0.00667569230769231 | 0.551142395209581 | 0.339083333333333 | -0.0402839121756487 |
| 1692 | -0.0632104615384615 | 0.306190219560878 | 0.330961576846307 | 0.0307002395209581 |
| 1693 | -0.0753101538461539 | -0.175592375249501 | -0.415833932135729 | 0.0367103393213573 |
| 1694 | -0.0300406153846154 | 0.155287984031936 | -0.449539221556886 | -0.0912885429141717 |
| 1695 | 0.0552830769230769 | 0.293682714570858 | 0.361012075848303 | -0.0951869860279441 |
| 1696 | 0.111817846153846 | -0.177704031936128 | 0.416240019960080 | 0.126861836327345 |
| 1697 | 0.127881230769231 | -0.292058363273453 | -0.504361077844311 | 0.104608223552894 |
| 1698 | 0.110357538461538 | 0.0864154890219561 | -0.157562075848303 | -0.0895017564870260 |
| 1699 | 0.0947113846153846 | 0.0862530538922156 | 0.737861576846307 | -0.0462940119760479 |
| 1700 | 0.105768000000000 | -0.171044191616766 | 0.490554091816367 | 0.101521956087824 |
| 1701 | 0.103681846153846 | 0.0113704590818363 | -0.340301596806387 | 0.0511670658682635 |
| 1702 | 0.0767704615384615 | 0.150414930139721 | -0.0219287425149701 | -0.0882022754491018 |
| 1703 | 0.0246166153846154 | -0.0776439920159681 | 0.488523652694611 | -0.0129948103792415 |
| 1704 | 0.00375507692307692 | -0.209216447105788 | 0.0397966067864271 | 0.0129948103792415 |
| 1705 | 0.0340043076923077 | -0.0498675848303393 | -0.318372854291417 | -0.0207916966067864 |
| 1706 | 0.0784393846153846 | 0.0872276646706587 | -0.267611876247505 | 0.0501924550898204 |
| 1707 | 0.108897230769231 | 0.0990854291417166 | 0.0353296407185629 | 0.0812175648702595 |
| 1708 | 0.0984664615384615 | 0.112242674650699 | 0.107613273453094 | 0.00324870259481038 |
| 1709 | 0.0684258461538462 | -0.00617253493013972 | 0.0726897205588822 | 0.0295631936127745 |
| 1710 | 0.0438092307692308 | 0.0204668263473054 | -0.144567265469062 | 0.0471061876247505 |
| 1711 | 0.0461040000000000 | 0.113054850299401 | -0.203043912175649 | -0.100222475049900 |
| 1712 | 0.0763532307692308 | -0.134496287425150 | 0.140506387225549 | -0.105257964071856 |
| 1713 | 0.0796910769230769 | -0.211652974051896 | 0.165277744510978 | 0.0505173253493014 |
| 1714 | 0.0454781538461538 | -0.000487305389221557 | -0.118983732534930 | 0.0419082634730539 |
| 1715 | 0.0173150769230769 | 0.103146307385230 | -0.309845009980040 | -0.129135928143713 |

| | | | | |
|---|---|---|---|---|
| 1716 | 0.0139772307692308 | 0.155450419161677 | 0.0113704590818363 | -0.00893393213572854 |
| 1717 | 0.0342129230769231 | 0.0652989221556886 | 0.261520558882236 | 0.129460798403194 |
| 1718 | 0.0538227692307692 | -0.0682227544910180 | -0.0986793413173653 | -0.0339489421157685 |
| 1719 | 0.0634190769230769 | -0.0357357285429142 | -0.417864371257485 | -0.0480807984031936 |
| 1720 | 0.0517366153846154 | -0.0214414371257485 | 0.129135928143713 | 0.100384910179641 |
| 1721 | 0.0312923076923077 | -0.0638370059880239 | 0.444260079840319 | -0.00471061876247505 |
| 1722 | 0.0471470769230769 | -0.118252774451098 | -0.244464870259481 | -0.0854408782435130 |
| 1723 | 0.0824030769230769 | -0.0925880239520958 | -0.229033532934132 | 0.132059760479042 |
| 1724 | 0.106393846153846 | -0.0506797604790419 | 0.383346906187625 | 0.0568522954091816 |
| 1725 | 0.0851150769230769 | 0.0644867465069860 | 0.147815968063872 | -0.229033532934132 |
| 1726 | 0.0277458461538462 | 0.194109980039920 | -0.590857784431138 | -0.0615629141716567 |
| 1727 | -0.00396369230769231 | 0.0878774051896208 | -0.264769261477046 | 0.234069021956088 |
| 1728 | 0.00521538461538462 | -0.0207916966067864 | 0.421925249500998 | 0.0209541317365269 |
| 1729 | 0.0486073846153846 | -0.118252774451098 | 0.0341113772455090 | -0.233094411177645 |
| 1730 | 0.0824030769230769 | -0.119714690618762 | -0.416240019960080 | 0.0639994411177645 |
| 1731 | 0.0730153846153846 | 0.0232282235528942 | 0.0146191616766467 | 0.277114331337325 |
| 1732 | 0.0454781538461538 | 0.0118577644710579 | 0.594512574850299 | -0.00487305389221557 |
| 1733 | 0.0239907692307692 | -0.0799180838323353 | 0.215226546906188 | -0.0644867465069860 |
| 1734 | 0.0244080000000000 | -0.00730958083832335 | -0.152282934131737 | 0.110943193612774 |
| 1735 | 0.0273286153846154 | 0.196546506986028 | -0.0259896207584830 | -0.0245277045908184 |
| 1736 | 0.0152289230769231 | 0.0784561676646707 | 0.212790019960080 | -0.116790858283433 |
| 1737 | -0.0204443076923077 | -0.208241836327345 | 0.214008283433134 | 0.0930753293413174 |
| 1738 | -0.0542400000000000 | -0.0475934930139721 | -0.175429940119760 | 0.0454818363273453 |
| 1739 | -0.0317095384615385 | 0.190049101796407 | -0.378067764471058 | -0.178353772455090 |
| 1740 | 0.0216960000000000 | -0.0224160479041916 | -0.241622255489022 | 0.113704590818363 |
| 1741 | 0.0579950769230769 | -0.0393093013972056 | 0.196140419161677 | 0.268992574850299 |
| 1742 | 0.0481901538461539 | 0.0748825948103793 | 0.108425449101796 | -0.0885271457085828 |
| 1743 | 0.000208615384615385 | -0.186313093812375 | -0.251368363273453 | -0.129785668662675 |
| 1744 | -0.0300406153846154 | -0.390981357285429 | -0.170556886227545 | 0.163409740518962 |
| 1745 | -0.0221132307692308 | -0.367103393213573 | 0.209541317365269 | 0.0691973652694611 |
| 1746 | 0.0100135384615385 | 0.481295289421158 | 0.228221357285429 | -0.243327824351297 |
| 1747 | 0.0219046153846154 | 1.03406203592814 | -0.194109980039920 | -0.0500300199600798 |
| 1748 | -0.00855323076923077 | 0.459528982035928 | -0.235937025948104 | 0.121339041916168 |
| 1749 | -0.0531969230769231 | 0.00292383233532934 | -0.0158374251497006 | -0.121501477045908 |
| 1750 | -0.0690516923076923 | -0.326494610778443 | 0.0739079840319361 | -0.141318562874252 |
| 1751 | -0.0371335384615385 | -0.382209860279441 | 0.0121826347305389 | 0.211490538922156 |
| 1752 | 0.0133513846153846 | -0.0138069860279441 | -0.0706592814371258 | 0.114029461077844 |
| 1753 | 0.0417230769230769 | 0.0579893413173653 | 0.114516766467066 | -0.243490259481038 |
| 1754 | 0.0254510769230769 | -0.864154890219561 | 0.586796906187625 | 0.0430453093812375 |
| 1755 | -0.00792738461538461 | -0.327956526946108 | 0.154719461077844 | 0.210515928143713 |
| 1756 | -0.00751015384615385 | 1.52867700598802 | -1.23775568862275 | -0.114679201596806 |
| 1757 | 0.0196098461538462 | 0.442473293413174 | -0.0369539920159681 | -0.126861836327345 |
| 1758 | 0.0354646153846154 | -1.12161457085828 | 1.98414510978044 | 0.150252495009980 |
| 1759 | 0.0264941538461538 | 0.0430453093812375 | -0.694410179640719 | -0.0282637125748503 |

| | | | | |
|---|---|---|---|---|
| 1760 | -0.00959630769230769 | 0.734694091816367 | -2.24485349301397 | -0.223835608782435 |
| 1761 | -0.0279544615384615 | -0.582979680638723 | 1.69298013972056 | 0.126374530938124 |
| 1762 | -0.0104307692307692 | -0.800480319361278 | 2.17906726546906 | 0.175105069860279 |
| 1763 | 0.0315009230769231 | 0.120039560878244 | -2.29926926147705 | -0.216850898203593 |
| 1764 | 0.0705120000000000 | 0.0471061876247505 | -1.35024201596806 | -0.116465988023952 |
| 1765 | 0.0346301538461538 | 0.0472686227544910 | 2.51530798403194 | 0.359956247504990 |
| 1766 | -0.0302492307692308 | 0.662410459081836 | 0.512076746506986 | 0.104770658682635 |
| 1767 | -0.0609156923076923 | 0.250799840319361 | -1.72627934131737 | -0.285398522954092 |
| 1768 | -0.0438092307692308 | -0.410148702594810 | 0.441823552894212 | 0.0536035928143713 |
| 1769 | -0.0100135384615385 | 0.477884151696607 | 0.999788223552894 | 0.210191057884232 |
| 1770 | -0.00980492307692308 | 0.557639800399202 | -1.03674221556886 | -0.0778064271457086 |
| 1771 | -0.0308750769230769 | -0.683202155688623 | 0.304565868263473 | -0.0826794810379242 |
| 1772 | -0.0611243076923077 | -0.504361077844311 | 1.00709780439122 | 0.0599385628742515 |
| 1773 | -0.0607070769230769 | 0.520279720558882 | -1.47247445109780 | -0.0789434730538922 |
| 1774 | -0.0131427692307692 | 0.182739520958084 | -0.529944610778443 | -0.0160810778443114 |
| 1775 | 0.0442264615384615 | -0.478371457085828 | 1.91429800399202 | 0.208404271457086 |
| 1776 | 0.0615415384615385 | 0.0678978842315369 | 0.175429940119760 | -0.0506797604790419 |
| 1777 | 0.0258683076923077 | 0.312525189620759 | -2.15632634730539 | -0.333804191616766 |
| 1778 | -0.0185667692307692 | -0.157886946107784 | 0.278170159680639 | 0.0760196407185629 |
| 1779 | -0.0452695384615385 | -0.132222195608782 | 1.99795209580838 | 0.319347465069860 |
| 1780 | -0.0390110769230769 | 0.0617253493013972 | -0.562837724550898 | -0.224972654690619 |
| 1781 | -0.0273286153846154 | -0.0990854291417166 | -1.77825858283433 | -0.405600518962076 |
| 1782 | -0.0423489230769231 | -0.106557445109780 | 0.555528143712575 | 0.168282794411178 |
| 1783 | -0.0725981538461538 | 0.00487305389221557 | 1.32303413173653 | 0.313337365269461 |
| 1784 | -0.0809427692307692 | 0.0791059081836327 | -0.721211976047904 | -0.239104510978044 |
| 1785 | -0.0394283076923077 | 0.237480159680639 | -0.605070858283433 | -0.197196247504990 |
| 1786 | 0.0148116923076923 | 0.143105349301397 | 0.812987824351298 | 0.268017964071856 |
| 1787 | 0.0600812307692308 | -0.170394451097804 | 0.419082634730539 | 0.0607507385229541 |
| 1788 | 0.0719723076923077 | -0.0810551297405190 | -0.547000299401198 | -0.201257125748503 |
| 1789 | 0.0421403076923077 | 0.286535568862276 | -0.101521956087824 | 0.0675730139720559 |
| 1790 | 0.0108480000000000 | 0.161135648702595 | 0.415833932135729 | 0.173643153692615 |
| 1791 | -0.0116824615384615 | -0.189399361277445 | -0.238373552894212 | -0.100222475049900 |
| 1792 | -0.0198184615384615 | -0.163734610778443 | -0.345986826347305 | -0.103146307385230 |
| 1793 | -0.0442264615384615 | 0.0352484231536926 | 0.180302994011976 | 0.198008423153693 |
| 1794 | -0.0955458461538462 | 0.000974610778443114 | -0.0856845309381238 | 0.192648063872255 |
| 1795 | -0.0940855384615385 | -0.0271266666666667 | -0.185582135728543 | -0.106070139720559 |
| 1796 | -0.0431833846153846 | 0.00503548902195609 | 0.409742614770459 | -0.0648116167664671 |
| 1797 | 0.0202356923076923 | -0.187612574850299 | 0.269642315369262 | 0.188424750499002 |
| 1798 | 0.0742670769230769 | -0.124100439121756 | -0.287104091816367 | 0.152039281437126 |
| 1799 | 0.0865753846153846 | 0.0924255888223553 | -0.0353296407185629 | 0.0219287425149701 |
| 1800 | 0.0725981538461538 | -0.0719587624750499 | 0.524259381237525 | -0.0256647504990020 |
| 1801 | 0.0223218461538462 | -0.283449301397206 | -0.0190861277445110 | -0.0157562075848303 |
| 1802 | 0.00250338461538462 | -0.0402839121756487 | -0.544969860279441 | 0.160323473053892 |
| 1803 | 0.0379680000000000 | 0.295469500998004 | -0.0852784431137725 | 0.155287984031936 |

| | | | | |
|---|---|---|---|---|
| 1804 | 0.0444350769230769 | 0.0326494610778443 | 0.413803493013972 | -0.219612295409182 |
| 1805 | 0.0127255384615385 | -0.226109700598802 | 0.000812175648702595 | -0.207754530938124 |
| 1806 | -0.0494418461538462 | 0.0532787225548902 | -0.657862275449102 | 0.208241836327345 |
| 1807 | -0.0809427692307692 | 0.141318562874252 | -0.145379441117764 | 0.154475808383234 |
| 1808 | -0.0678000000000000 | -0.0782937325349302 | 0.462127944111777 | -0.241053732534930 |
| 1809 | -0.0333784615384615 | -0.00925880239520958 | 0.112486327345309 | -0.179328383233533 |
| 1810 | -0.00354646153846154 | 0.168445229540918 | -0.518574151696607 | 0.196708942115768 |
| 1811 | -0.0287889230769231 | 0.0594512574850299 | -0.164059481037924 | 0.0669232734530938 |
| 1812 | -0.0571606153846154 | -0.144892135728543 | 0.425173952095808 | -0.287022874251497 |
| 1813 | -0.0567433846153846 | -0.00990854291417166 | 0.0596949101796407 | -0.0823546107784431 |
| 1814 | -0.0323353846153846 | 0.190049101796407 | -0.352890319361277 | 0.219287425149701 |
| 1815 | -0.0183581538461538 | 0.000324870259481038 | -0.172993413173653 | 0.00292383233532934 |
| 1816 | -0.0469384615384615 | -0.0643243113772455 | 0.190455189620759 | -0.157562075848303 |
| 1817 | -0.0824030769230769 | -0.0358981636726547 | 0.224566566866267 | 0.0935626347305389 |
| 1818 | -0.111400615384615 | -0.0703344111776447 | -0.0288322355289421 | 0.159511297405190 |
| 1819 | -0.0922080000000000 | -0.174292894211577 | -0.124262874251497 | -0.0235530938123753 |
| 1820 | -0.0348387692307692 | -0.113054850299401 | 0.196546506986028 | -0.0172181237524950 |
| 1821 | -0.00521538461538462 | 0.475934930139721 | 0.348017265469062 | 0.0675730139720559 |
| 1822 | -0.0150203076923077 | 0.333316886227545 | -0.0215226546906188 | -0.0706592814371258 |
| 1823 | -0.0584123076923077 | -0.0985981237524950 | -0.377255588822355 | -0.132222195608782 |
| 1824 | -0.0853236923076923 | 0.142618043912176 | -0.152282934131737 | 0.0602634331337325 |
| 1825 | -0.0740584615384615 | -0.145541876247505 | 0.0885271457085828 | 0.0563649900199601 |
| 1826 | -0.0454781538461538 | -0.467650738522954 | -0.133602894211577 | -0.133359241516966 |
| 1827 | -0.0162720000000000 | 0.155450419161677 | -0.269236227544910 | -0.0760196407185629 |
| 1828 | -0.0275372307692308 | 0.303428822355289 | -0.115328942115768 | 0.110618323353293 |
| 1829 | -0.0636276923076923 | -0.302779081836327 | 0.240810079840319 | 0.0173805588822355 |
| 1830 | -0.0625846153846154 | 0.243652694610778 | 0.325276347305389 | -0.153663632734531 |
| 1831 | -0.0183581538461538 | 0.699770538922156 | -0.0812175648702595 | -0.0157562075848303 |
| 1832 | 0.0458953846153846 | -0.200120079840319 | -0.184769960079840 | 0.107856926147705 |
| 1833 | 0.0811513846153846 | -0.414047145708583 | 0.395935628742515 | -0.0558776846307385 |
| 1834 | 0.0767704615384615 | 0.153338762475050 | 0.335834630738523 | -0.0536035928143713 |
| 1835 | 0.0421403076923077 | 0.0727709381237525 | -0.338271157684631 | 0.133034371257485 |
| 1836 | 0.00917907692307692 | -0.407874610778443 | -0.168120359281437 | 0.0566898602794411 |
| 1837 | 0.0137686153846154 | -0.0263144910179641 | 0.293195409181637 | -0.0753699001996008 |
| 1838 | 0.0212787692307692 | 0.235855808383234 | 0.100303692614770 | -0.00227409181636727 |
| 1839 | -0.00104307692307692 | -0.234231457085828 | -0.342332035928144 | 0.0763445109780439 |
| 1840 | -0.0540313846153846 | 0.139206906187625 | -0.101928043912176 | 0.0349235528942116 |
| 1841 | -0.0627932307692308 | 0.347773612774451 | 0.325682435129741 | 0.0125075049900200 |
| 1842 | -0.0198184615384615 | -0.491366267465070 | 0.0759384231536926 | 0.0579893413173653 |
| 1843 | 0.0189840000000000 | -0.228871097804391 | -0.205886526946108 | 0.0445072255489022 |
| 1844 | 0.0429747692307692 | 0.818510618762475 | -0.0661923153692615 | 0.0441823552894212 |
| 1845 | 0.0390110769230769 | 0.228546227544910 | 0.116953293413174 | 0.130110538922156 |
| 1846 | 0.0137686153846154 | -0.463589860279441 | -0.00609131736526946 | 0.0467813173652695 |
| 1847 | -0.00354646153846154 | 0.221561516966068 | -0.188424750499002 | -0.158211816367265 |

| | | | | |
|---|---|---|---|---|
| 1848 | 0.0202356923076923 | 0.544969860279441 | -0.149846407185629 | -0.0347611177644711 |
| 1849 | 0.0673827692307692 | -0.0142942914171657 | 0.140100299401198 | 0.196546506986028 |
| 1850 | 0.0907476923076923 | -0.442960598802395 | 0.248525748502994 | -0.0188424750499002 |
| 1851 | 0.0728067692307692 | -0.0527914171656687 | -0.0280200598802395 | -0.288484790419162 |
| 1852 | 0.0458953846153846 | 0.0518168063872256 | -0.0657862275449102 | -0.0484056686626747 |
| 1853 | 0.0490246153846154 | -0.0994102994011976 | 0.243246606786427 | 0.189561796407186 |
| 1854 | 0.0826116923076923 | 0.203206347305389 | 0.263144910179641 | -0.0662735329341317 |
| 1855 | 0.111192000000000 | -0.105095528942116 | -0.0527914171656687 | -0.255023153692615 |
| 1856 | 0.106185230769231 | -0.334778802395210 | -0.129948103792415 | 0.000649740518962076 |
| 1857 | 0.0696775384615385 | 0.0786186027944112 | 0.0385783433133733 | 0.173643153692615 |
| 1858 | 0.0337956923076923 | 0.175429940119760 | 0.0783749500998004 | -0.0310251097804391 |
| 1859 | 0.0315009230769231 | -0.272241277445110 | -0.184769960079840 | -0.156262594810379 |
| 1860 | 0.0454781538461538 | -0.275814850299401 | -0.123856786427146 | 0.00714714570858283 |
| 1861 | 0.0588295384615385 | 0.0547406387225549 | -0.0162435129740519 | 0.0946996806387226 |
| 1862 | 0.0523624615384615 | -0.00795932135728543 | 0.136851596806387 | 0.00633497005988024 |
| 1863 | 0.0298320000000000 | -0.00211165668662675 | 0.0467000998003992 | -0.0232282235528942 |
| 1864 | 0.0179409230769231 | 0.216850898203593 | 0.0243652694610778 | 0.0826794810379242 |
| 1865 | 0.0410972307692308 | 0.185176047904192 | 0.108831536926148 | 0.122963393213573 |
| 1866 | 0.0899132307692308 | -0.152526586826347 | -0.0280200598802395 | 0.0248525748502994 |
| 1867 | 0.112235076923077 | 0.0410960878243513 | 0.389032135728543 | -0.00633497005988024 |
| 1868 | 0.106393846153846 | 0.219449860279441 | -0.235124850299401 | 0.133196806387226 |
| 1869 | 0.0796910769230769 | -0.0178678642714571 | -0.433701796407186 | 0.201094690618762 |
| 1870 | 0.0784393846153846 | -0.0615629141716567 | 0.125887225548902 | -0.0513295009980040 |
| 1871 | 0.0840720000000000 | 0.144404830339321 | 0.587609081836327 | -0.154963113772455 |
| 1872 | 0.0723895384615385 | 0.103796047904192 | -0.377661676646707 | 0.0774815568862275 |
| 1873 | 0.0611243076923077 | -0.169907145708583 | -0.648116167664671 | 0.0407712175648703 |
| 1874 | 0.0273286153846154 | -0.0963240319361277 | 0.935220259481038 | -0.214901676646707 |
| 1875 | 0.00792738461538461 | -0.157074770459082 | 0.180302994011976 | -0.187937445109780 |
| 1876 | 0.0183581538461538 | -0.201094690618762 | -0.525071556886228 | 0.0332992015968064 |
| 1877 | 0.0636276923076923 | 0.00341113772455090 | -0.311875449101796 | 0.0575020359281437 |
| 1878 | 0.0967975384615385 | 0.0729333732534930 | 0.496645409181637 | -0.0813800000000000 |
| 1879 | 0.0821944615384615 | -0.0808926946107784 | -0.0625375249500998 | -0.109156407185629 |
| 1880 | 0.0340043076923077 | -0.00698471057884232 | -0.622532634730539 | 0.00942123752495010 |
| 1881 | -0.0202356923076923 | 0.220424471057884 | 0.293195409181637 | 0.100709780439122 |
| 1882 | -0.0248252307692308 | 0.0134821157684631 | -0.180302994011976 | 0.0755323353293413 |
| 1883 | 0 | -0.0802429540918164 | -0.0962428143712575 | 0.00454818363273453 |
| 1884 | 0.0125169230769231 | -0.0164059481037924 | 0.262332734530938 | -0.0617253493013972 |
| 1885 | -0.0114738461538462 | 0.0271266666666667 | 0.235124850299401 | 0.0485681037924152 |
| 1886 | -0.0354646153846154 | 0.166658443113772 | -0.308220658682635 | 0.139694211576846 |
| 1887 | -0.0177323076923077 | 0.0703344111776447 | 0.0268017964071856 | -0.0633497005988024 |
| 1888 | 0.0125169230769231 | -0.104283353293413 | 0.645273552894212 | -0.143430219560878 |
| 1889 | 0.0519452307692308 | 0.0165683832335329 | -0.213602195608782 | 0.0682227544910180 |
| 1890 | 0.0705120000000000 | 0.243652694610778 | -0.557558582834331 | 0.102496566866267 |
| 1891 | 0.0461040000000000 | 0.120039560878244 | -0.0869027944111777 | -0.0794307784431138 |

| | | | | |
|---|---|---|---|---|
| 1892 | 0.0112652307692308 | -0.282961996007984 | 0.481214071856287 | -0.0805678243512974 |
| 1893 | -0.0219046153846154 | -0.161947824351297 | -0.272484930139721 | 0.0583142115768463 |
| 1894 | -0.0198184615384615 | 0.214901676646707 | -0.613192614770459 | 0.0216038722554890 |
| 1895 | -0.0173150769230769 | 0.294982195608782 | 0.218475249500998 | -0.0714714570858283 |
| 1896 | -0.0383852307692308 | 0.210191057884232 | 0.411773053892216 | 0.0534411576846307 |
| 1897 | -0.0617501538461538 | -0.526452255489022 | -0.0129948103792415 | 0.0506797604790419 |
| 1898 | -0.0890787692307692 | -0.795444830339321 | -0.273297105788423 | -0.109481277445110 |
| 1899 | -0.0719723076923077 | 0.0445072255489022 | 0.195734331337325 | 0.0251774451097804 |
| 1900 | -0.0325440000000000 | 0.333154451097804 | 0.129948103792415 | 0.220911776447106 |
| 1901 | 0.00104307692307692 | 0.00373600798403194 | -0.124262874251497 | 0.0662735329341317 |
| 1902 | 0.00208615384615385 | 0.0222536127744511 | -0.101928043912176 | -0.135308463073852 |
| 1903 | -0.0325440000000000 | 0.0430453093812375 | -0.0637557884231537 | 0.0852784431137725 |
| 1904 | -0.0279544615384615 | 0.439224590818363 | -0.0698471057884232 | 0.225135089820359 |
| 1905 | -0.00813600000000000 | 1.00644806387226 | -0.0247713572854291 | -0.100222475049900 |
| 1906 | 0.0152289230769231 | -0.181115169660679 | 0.141724650698603 | -0.133684111776447 |
| 1907 | -0.00292061538461538 | -0.972499121756487 | -0.0609131736526946 | 0.161622954091816 |
| 1908 | -0.0788566153846154 | 0.444747385229541 | -0.0247713572854291 | 0.0500300199600798 |
| 1909 | -0.126212307692308 | 0.901352534930140 | 0.179490818363273 | -0.183714131736527 |
| 1910 | -0.158756307692308 | -0.401702075848303 | 0.237155289421158 | -0.00601009980039920 |
| 1911 | -0.133722461538462 | -0.488117564870260 | -0.0840601796407186 | 0.199795209580838 |
| 1912 | -0.0776049230769231 | 0.626187425149701 | -0.180709081836327 | 0.0570147305389222 |
| 1913 | -0.0713464615384615 | 0.369864790419162 | 0.0966489021956088 | -0.101359520958084 |
| 1914 | -0.0759360000000000 | -0.319185029940120 | -0.00446696606786427 | 0.0641618762475050 |
| 1915 | -0.0990923076923077 | -0.121988782435130 | -0.171369061876248 | 0.134983592814371 |
| 1916 | -0.0945027692307692 | -0.359144071856287 | -0.118171556886228 | -0.0589639520958084 |
| 1917 | -0.0596640000000000 | -0.797231616766467 | 0.216038722554890 | -0.0536035928143713 |
| 1918 | -0.0321267692307692 | -0.0834916566866268 | 0.0897454091816367 | 0.0298880638722555 |
| 1919 | -0.000625846153846154 | 0.299530379241517 | -0.0552279441117765 | 0.0242028343313373 |
| 1920 | -0.0158547692307692 | -0.339814291417166 | 0.0966489021956088 | 0.0100709780439122 |
| 1921 | -0.0273286153846154 | -0.0919382834331337 | 0.227815269461078 | -0.0579893413173653 |
| 1922 | 0.0123083076923077 | 0.626024990019960 | 0.0430453093812375 | -0.0898266267465070 |
| 1923 | 0.0500676923076923 | 0.216363592814371 | -0.248931836327345 | -0.0138069860279441 |
| 1924 | 0.0584123076923077 | -0.367915568862275 | 0.0596949101796407 | 0.000487305389221557 |
| 1925 | 0.0216960000000000 | 0.134821157684631 | 0.0791871257485030 | -0.0584766467065868 |
| 1926 | -0.0154375384615385 | 0.372301317365269 | -0.0747201596806387 | -0.0713090219560878 |
| 1927 | -0.0423489230769231 | -0.152689021956088 | -0.167714271457086 | -0.00389844311377246 |
| 1928 | -0.0375507692307692 | -0.163084870259481 | 0.0661923153692615 | 0.0620502195608782 |
| 1929 | 0.00938769230769231 | 0.318047984031936 | 0.322839820359281 | -0.0662735329341317 |
| 1930 | 0.0221132307692308 | 0.355570499001996 | 0.0511670658682635 | -0.00178678642714571 |
| 1931 | 0.00730153846153846 | 0.00276139720558882 | 0.0231470059880240 | 0.202394171656687 |
| 1932 | -0.0275372307692308 | 0.0175429940119760 | 0.0816236526946108 | 0.0659486626746507 |
| 1933 | -0.0202356923076923 | 0.0602634331337325 | 0.144161177644711 | -0.0492178443113772 |
| 1934 | 0.0271200000000000 | -0.118252774451098 | 0.0645679640718563 | 0.140343952095808 |
| 1935 | 0.0461040000000000 | -0.357357285429142 | -0.0730958083832335 | 0.203043912175649 |

| | | | | |
|---|---|---|---|---|
| 1936 | 0.0327526153846154 | -0.440686506986028 | -0.168932534930140 | -0.0774815568862275 |
| 1937 | -0.0367163076923077 | -0.148953013972056 | -0.179896906187625 | -0.138069860279441 |
| 1938 | -0.0659224615384615 | 0.0300504990019960 | 0.0150252495009980 | 0.114029461077844 |
| 1939 | -0.0356732307692308 | 0.0121826347305389 | -0.00893393213572854 | 0.0878774051896208 |
| 1940 | 0.00438092307692308 | -0.0852784431137725 | -0.176648203592814 | -0.220911776447106 |
| 1941 | 0.0602898461538462 | -0.0924255888223553 | -0.129948103792415 | -0.192648063872255 |
| 1942 | 0.0694689230769231 | 0.0901514970059880 | 0.226597005988024 | 0.0758572055888224 |
| 1943 | 0.0408886153846154 | 0.134171417165669 | 0.186394311377246 | 0.0552279441117765 |
| 1944 | 0.0146030769230769 | 0.134171417165669 | -0.194109980039920 | -0.0852784431137725 |
| 1945 | 0.0118910769230769 | 0.0635121357285429 | -0.0580705588822355 | -0.119877125748503 |
| 1946 | 0.0356732307692308 | -0.117765469061876 | 0.289134530938124 | -0.0261520558882236 |
| 1947 | 0.0331698461538462 | 0.0274515369261477 | 0.0511670658682635 | 0.0345986826347305 |
| 1948 | 0.00208615384615385 | 0.228546227544910 | -0.244058782435130 | -0.00942123752495010 |
| 1949 | -0.0394283076923077 | -0.00276139720558882 | 0.0633497005988024 | -0.0958367265469062 |
| 1950 | -0.0515280000000000 | -0.121663912175649 | 0.253804890219561 | -0.102334131736527 |
| 1951 | -0.0225304615384615 | -0.00438574850299401 | -0.0832480039920160 | 0.0748825948103793 |
| 1952 | 0.000625846153846154 | 0.0831667864271457 | -0.169338622754491 | 0.0614004790419162 |
| 1953 | 0.0227390769230769 | 0.0313499800399202 | 0.285885828343313 | -0.0701719760479042 |
| 1954 | 0.0271200000000000 | -0.136607944111776 | 0.235937025948104 | 0.0289134530938124 |
| 1955 | 0.0425575384615385 | 0.0654613572854292 | -0.245277045908184 | 0.0885271457085828 |
| 1956 | 0.0851150769230769 | 0.0599385628742515 | -0.138069860279441 | -0.0344362475049900 |
| 1957 | 0.121622769230769 | -0.250637405189621 | 0.126699401197605 | -0.00568522954091816 |
| 1958 | 0.140606769230769 | -0.0397966067864271 | -0.0893393213572854 | 0.0633497005988024 |
| 1959 | 0.0926252307692308 | 0.162435129740519 | -0.300098902195609 | -0.0430453093812375 |
| 1960 | 0.0306664615384615 | -0.187287704590818 | -0.0990854291417166 | -0.0591263872255489 |
| 1961 | -0.00563261538461538 | -0.181927345309381 | -0.00893393213572854 | 0.0373600798403194 |
| 1962 | -0.000625846153846154 | 0.352809101796407 | -0.0552279441117765 | 0.0895017564870260 |
| 1963 | 0.0644621538461539 | 0.244627305389222 | 0.103958483033932 | -0.00714714570858283 |
| 1964 | 0.124126153846154 | -0.154963113772455 | 0.0527914171656687 | -0.0687100598802395 |
| 1965 | 0.147699692307692 | -0.0360605988023952 | -0.0824358283433134 | 0.0332992015968064 |
| 1966 | 0.119328000000000 | 0.122638522954092 | 0.0268017964071856 | 0.0771566866267465 |
| 1967 | 0.0930424615384616 | -0.0765069461077844 | 0.231876147704591 | 0.0948621157684631 |
| 1968 | 0.0993009230769231 | 0.107532055888224 | -0.0296444111776447 | 0.0591263872255489 |
| 1969 | 0.0986750769230769 | 0.273540758483034 | -0.261520558882236 | -0.0497051497005988 |
| 1970 | 0.0788566153846154 | 0.0334616367265469 | -0.00649740518962076 | 0.0449945309381238 |
| 1971 | 0.0283716923076923 | 0.287347744510978 | 0.153095109780439 | 0.211328103792415 |
| 1972 | -0.00438092307692308 | -0.208241836327345 | -0.0609131736526946 | 0.0513295009980040 |
| 1973 | 0.0120996923076923 | -0.838490139720559 | -0.172993413173653 | -0.207104790419162 |
| 1974 | 0.0700947692307692 | -0.342250818363273 | 0.0665984031936128 | -0.0506797604790419 |
| 1975 | 0.146865230769231 | 0.469924830339321 | 0.144567265469062 | 0.199307904191617 |
| 1976 | 0.190048615384615 | 0.451732095808383 | -0.0243652694610778 | -0.0196546506986028 |
| 1977 | 0.204234461538462 | -0.242353213572854 | -0.0385783433133733 | -0.276627025948104 |
| 1978 | 0.196098461538462 | 0.0579893413173653 | 0.0828419161676647 | -0.0183551696606786 |
| 1979 | 0.187962461538462 | 0.475934930139721 | -0.183145608782435 | 0.220099600798403 |

| | | | | |
|---|---|---|---|---|
| 1980 | 0.177948923076923 | 0.227246746506986 | 0.469031437125748 | -0.00129948103792415 |
| 1981 | 0.117241846153846 | -0.516056407185629 | 0.110049800399202 | -0.0623750898203593 |
| 1982 | 0.0248252307692308 | -0.693922874251497 | -1.05664051896208 | 0.0812175648702595 |
| 1983 | -0.0617501538461538 | 0.347286307385230 | -0.668420558882236 | -0.0506797604790419 |
| 1984 | -0.0932510769230769 | 0.830693253493014 | 1.42293173652695 | -0.0800805189620758 |
| 1985 | -0.0609156923076923 | -0.0506797604790419 | 0.868215768463074 | 0.221074211576846 |
| 1986 | -0.0150203076923077 | -0.340951337325349 | -1.98170858283433 | 0.0994102994011976 |
| 1987 | 0.0325440000000000 | 0.444422514970060 | 0.300911077844311 | -0.320809381237525 |
| 1988 | 0.0486073846153846 | 0.424280558882236 | 1.98333293413174 | -0.00389844311377246 |
| 1989 | 0.0490246153846154 | -0.0776439920159681 | -0.138882035928144 | 0.409336526946108 |
| 1990 | 0.0809427692307692 | -0.412097924151697 | -1.91835888223553 | -0.0107207185628743 |
| 1991 | 0.109523076923077 | -0.479670938123753 | 0.358575548902196 | -0.383834211576846 |
| 1992 | 0.121831384615385 | -0.136932814371257 | 1.32628283433134 | 0.0259896207584830 |
| 1993 | 0.0792738461538462 | 0.428016566866267 | -0.903545409181637 | 0.340301596806387 |
| 1994 | -0.0100135384615385 | 0.124912614770459 | -0.159186427145709 | 0.0365479041916168 |
| 1995 | -0.0692603076923077 | -0.572583832335329 | 0.167714271457086 | -0.138394730538922 |
| 1996 | -0.0721809230769231 | 0.0737455489021956 | 0.0235530938123753 | 0.0557152495009980 |
| 1997 | -0.0181495384615385 | 0.629436127744511 | 0.205074351297405 | 0.156100159680639 |
| 1998 | 0.0337956923076923 | -0.103633612774451 | 0.854408782435130 | 0.0449945309381238 |
| 1999 | 0.0548658461538462 | -0.652177045908184 | -0.422331337325349 | -0.0219287425149701 |
| 2000 | 0.0513193846153846 | -0.0164059481037924 | -1.07694491017964 | -0.0791059081836327 |
| 2001 | 0.0519452307692308 | 0.414534451097804 | 1.19146167664671 | -0.0628623952095808 |
| 2002 | 0.0740584615384615 | -0.0440199201596806 | 0.619283932135729 | 0.149927624750499 |
| 2003 | 0.0717636923076923 | -0.155937724550898 | -1.19998952095808 | 0.166171137724551 |
| 2004 | 0.0479815384615385 | 0.158211816367265 | -0.943748103792415 | -0.124262874251497 |
| 2005 | -0.0216960000000000 | 0.107694491017964 | 1.15531986027944 | -0.153501197604790 |
| 2006 | -0.103473230769231 | 0.0752074650698603 | 0.577050798403194 | 0.129948103792415 |
| 2007 | -0.118284923076923 | 0.213764630738523 | -1.15207115768463 | 0.0987605588822355 |
| 2008 | -0.0838633846153846 | 0.0852784431137725 | -0.213602195608782 | -0.135795768463074 |
| 2009 | -0.0118910769230769 | -0.0916134131736527 | 0.789434730538922 | -0.127024271457086 |
| 2010 | 0.0400541538461538 | -0.141968303393214 | 0.489741916167665 | 0.0383346906187625 |
| 2011 | 0.0429747692307692 | -0.0768318163672655 | -0.497051497005988 | 0.0167308183632735 |
| 2012 | 0.0337956923076923 | 0.0438574850299401 | 0.0182739520958084 | -0.118577644710579 |
| 2013 | 0.0285803076923077 | 0.0992478642714571 | 0.322433732534930 | -0.0146191616766467 |
| 2014 | 0.0442264615384615 | -0.0363854690618763 | -0.175023852295409 | 0.0427204391217565 |
| 2015 | 0.0423489230769231 | -0.147166227544910 | -0.135227245508982 | -0.118740079840319 |
| 2016 | -0.00813600000000000 | -0.0797556487025948 | -0.0483244510978044 | -0.102496566866267 |
| 2017 | -0.0911649230769231 | 0.0929128942115768 | 0.0491366267465070 | 0.0586390818363273 |
| 2018 | -0.155835692307692 | 0.0969737724550898 | -0.218069161676647 | 0.0367103393213573 |
| 2019 | -0.140815384615385 | -0.0760196407185629 | 0.206292614770459 | -0.0792683433133733 |
| 2020 | -0.0636276923076923 | -0.0662735329341317 | 0.205480439121756 | -0.0238779640718563 |
| 2021 | 0.0137686153846154 | -0.0930753293413174 | -0.118983732534930 | 0.0826794810379242 |
| 2022 | 0.0486073846153846 | -0.0959991616766467 | 0.198983033932136 | 0.0519792415169661 |
| 2023 | 0.0502763076923077 | -0.0511670658682635 | 0.186394311377246 | 0.00763445109780439 |

| | | | | |
|---|---|---|---|---|
| 2024 | 0.0544486153846154 | -0.125075049900200 | -0.179490818363273 | -0.0540908982035928 |
| 2025 | 0.0692603076923077 | -0.0714714570858283 | -0.347205089820359 | -0.128811057884232 |
| 2026 | 0.0846978461538462 | 0.147815968063872 | 0.0596949101796407 | 0.00601009980039920 |
| 2027 | 0.0477729230769231 | 0.132059760479042 | 0.0207104790419162 | 0.139856646706587 |
| 2028 | -0.0304578461538462 | -0.140343952095808 | -0.232282235528942 | -0.0862530538922156 |
| 2029 | -0.0907476923076923 | -0.0813800000000000 | -0.105176746506986 | -0.233256846307385 |
| 2030 | -0.109105846153846 | 0.135470898203593 | 0.291977145708583 | 0.0901514970059880 |
| 2031 | -0.0611243076923077 | 0.0139694211576846 | 0.167714271457086 | 0.289621836327345 |
| 2032 | -0.00271200000000000 | -0.123288263473054 | -0.263957085828343 | -0.0596136926147705 |
| 2033 | 0.0354646153846154 | 0.0186800399201597 | -0.0966489021956088 | -0.272403712574850 |
| 2034 | 0.0431833846153846 | 0.117115728542914 | 0.218069161676647 | 0.127511576846307 |
| 2035 | 0.0244080000000000 | 0.0438574850299401 | 0.169744710578842 | 0.282474690618762 |
| 2036 | 0.0352560000000000 | -0.00974610778443114 | -0.380098203592814 | -0.106719880239521 |
| 2037 | 0.0521538461538462 | 0.103471177644711 | -0.244464870259481 | -0.0856033133732535 |
| 2038 | 0.0342129230769231 | 0.151227105788423 | 0.158780339321357 | 0.234556327345309 |
| 2039 | -0.0410972307692308 | -0.103308742514970 | 0.105582834331337 | 0.0805678243512974 |
| 2040 | -0.118910769230769 | -0.153338762475050 | -0.190455189620759 | -0.118090339321357 |
| 2041 | -0.132888000000000 | -0.0307002395209581 | -0.0771566866267465 | 0.0667608383233533 |
| 2042 | -0.102847384615385 | -0.0495427145708583 | 0.274921457085828 | 0.118415209580838 |
| 2043 | -0.0400541538461538 | 0.152039281437126 | 0.0495427145708583 | -0.108506666666667 |
| 2044 | 0.0158547692307692 | 0.274190499001996 | 0.0982732534930140 | -0.0765069461077844 |
| 2045 | 0.0296233846153846 | 0.0778064271457086 | -0.0629436127744511 | 0.196221636726547 |
| 2046 | 0.00125169230769231 | 0.0289134530938124 | -0.0836540918163673 | 0.0295631936127745 |
| 2047 | -0.0271200000000000 | -0.0446696606786427 | 0.161216866267465 | -0.290921317365269 |
| 2048 | -0.00980492307692308 | -0.0540908982035928 | 0.0418270459081836 | -0.0227409181636727 |
| 2049 | 0.00292061538461538 | -0.0227409181636727 | -0.309438922155689 | 0.256322634730539 |
| 2050 | -0.0264941538461538 | -0.0116953293413174 | -0.315530239520958 | -0.0474310578842315 |
| 2051 | -0.0988836923076923 | 0.219937165668663 | 0.164059481037924 | -0.262982475049900 |
| 2052 | -0.160216615384615 | -0.0173805588822355 | 0.0718775449101796 | 0.0639994411177645 |
| 2053 | -0.146030769230769 | -0.204343393213573 | -0.114922854291417 | 0.204018522954092 |
| 2054 | -0.0955458461538462 | 0.118090339321357 | -0.103552395209581 | -0.0698471057884232 |
| 2055 | -0.0421403076923077 | -0.118090339321357 | 0.157155988023952 | -0.0742328542914172 |
| 2056 | -0.0135600000000000 | -0.652826786427146 | 0.161216866267465 | 0.114841636726547 |
| 2057 | -0.00479815384615385 | -0.294332455089820 | -0.121420259481038 | 0.00909636726546906 |
| 2058 | 0.0187753846153846 | 0.400077724550898 | -0.0998976047904192 | -0.100384910179641 |
| 2059 | 0.0465212307692308 | 0.284748782435130 | 0.0531975049900200 | 0.0563649900199601 |
| 2060 | 0.0857409230769231 | -0.0371976447105788 | 0.145785528942116 | 0.0781312974051896 |
| 2061 | 0.0959630769230769 | 0.0240403992015968 | -0.125887225548902 | -0.0961615968063872 |
| 2062 | 0.0448523076923077 | 0.220099600798403 | 0.00934001996007984 | -0.0592888223552894 |
| 2063 | -0.0150203076923077 | 0.220099600798403 | 0.268017964071856 | 0.172668542914172 |
| 2064 | -0.0362990769230769 | -0.0344362475049900 | 0.109643712574850 | 0.0791059081836327 |
| 2065 | 0.0100135384615385 | -0.214901676646707 | -0.0710653692614771 | -0.161622954091816 |
| 2066 | 0.0778135384615385 | -0.0555528143712575 | -0.00893393213572854 | 0.00243652694610778 |
| 2067 | 0.101595692307692 | 0.368890179640719 | 0.127511576846307 | 0.165846267465070 |

| | | | | |
|---|---|---|---|---|
| 2068 | 0.0876184615384615 | 0.519467544910180 | 0.00690349301397206 | 0.0417458283433134 |
| 2069 | 0.0519452307692308 | 0.186150658682635 | -0.0219287425149701 | -0.0108831536926148 |
| 2070 | 0.0423489230769231 | -0.120851736526946 | -0.0580705588822355 | 0.0571771656686627 |
| 2071 | 0.0559089230769231 | -0.0235530938123753 | -0.0170556886227545 | 0.0110455888223553 |
| 2072 | 0.0538227692307692 | 0.0834916566866268 | 0.185988223552894 | -0.0164059481037924 |
| 2073 | 0.0196098461538462 | -0.187125269461078 | 0.144161177644711 | 0.0804053892215569 |
| 2074 | -0.0609156923076923 | -0.307814570858283 | -0.138882035928144 | -0.00324870259481038 |
| 2075 | -0.105768000000000 | -0.0818673053892216 | 0.0300504990019960 | -0.176079680638723 |
| 2076 | -0.0761446153846154 | -0.00259896207584830 | 0.389438223552894 | -0.0456442714570858 |
| 2077 | -0.00125169230769231 | -0.0302129341317365 | 0.103958483033932 | 0.137744990019960 |
| 2078 | 0.0719723076923077 | -0.0250150099800399 | -0.166902095808383 | -0.0774815568862275 |
| 2079 | 0.0884529230769231 | 0.0417458283433134 | -0.000406087824351297 | -0.199145469061876 |
| 2080 | 0.0805255384615385 | -0.0661110978043912 | 0.235124850299401 | 0.0799180838323353 |
| 2081 | 0.0563261538461538 | -0.214414371257485 | -0.0105582834331337 | 0.119064950099800 |
| 2082 | 0.0400541538461538 | 0.113704590818363 | -0.168932534930140 | -0.0932377644710579 |
| 2083 | 0.0410972307692308 | 0.281175209580838 | 0.0462940119760479 | -0.0402839121756487 |
| 2084 | 0.0108480000000000 | 0.0958367265469062 | 0.105582834331337 | 0.0862530538922156 |
| 2085 | -0.0346301538461538 | 0.115328942115768 | -0.0467000998003992 | 0.000324870259481038 |
| 2086 | -0.0894960000000000 | 0.201744431137725 | -0.148628143712575 | 0.00730958083832335 |
| 2087 | -0.109523076923077 | 0.0776439920159681 | 0.0601009980039920 | 0.0862530538922156 |
| 2088 | -0.0815686153846154 | -0.0880398403193613 | -0.0994915169660679 | -0.0488929740518962 |
| 2089 | -0.0490246153846154 | -0.0360605988023952 | -0.244058782435130 | -0.144242395209581 |
| 2090 | -0.0173150769230769 | -0.108344231536926 | -0.131978542914172 | 0.0774815568862275 |
| 2091 | -0.0277458461538462 | -0.225459960079840 | -0.00487305389221557 | 0.189886666666667 |
| 2092 | -0.0413058461538462 | 0.0584766467065868 | -0.290758882235529 | -0.134333852295409 |
| 2093 | -0.0210701538461538 | 0.226109700598802 | -0.0198983033932136 | -0.172506107784431 |
| 2094 | 0.0137686153846154 | -0.0500300199600798 | 0.606695209580838 | 0.263794650698603 |
| 2095 | 0.0438092307692308 | -0.164059481037924 | -0.512482834331337 | 0.166496007984032 |
| 2096 | 0.00458953846153846 | -0.0138069860279441 | -1.02862045908184 | -0.324545389221557 |
| 2097 | -0.0598726153846154 | -0.121826347305389 | 1.22597914171657 | -0.0898266267465070 |
| 2098 | -0.0894960000000000 | -0.328768702594810 | 1.43998742514970 | 0.305702914171657 |
| 2099 | -0.0619587692307692 | -0.168932534930140 | -1.89643013972056 | -0.0563649900199601 |
| 2100 | 0.0114738461538462 | 0.0268017964071856 | -0.591669960079840 | -0.340464031936128 |
| 2101 | 0.0552830769230769 | -0.0311875449101796 | 2.48606966067864 | 0.0968113373253493 |
| 2102 | 0.0761446153846154 | 0.137095249500998 | -0.100709780439122 | 0.341925948103792 |
| 2103 | 0.0652966153846154 | 0.366128782435130 | -2.31307624750499 | -0.121826347305389 |
| 2104 | 0.0513193846153846 | 0.204993133732535 | 0.507609780439122 | -0.185176047904192 |
| 2105 | 0.0619587692307692 | -0.0259896207584830 | 1.90211536926148 | 0.326332175648703 |
| 2106 | 0.0550744615384615 | -0.0639994411177645 | -0.958367265469062 | 0.180790299401198 |
| 2107 | 0.0258683076923077 | -0.00747201596806387 | -0.874307085828343 | -0.328443832335329 |
| 2108 | -0.0450609230769231 | -0.00454818363273453 | 1.03633612774451 | -0.0912885429141717 |
| 2109 | -0.0911649230769231 | -0.0834916566866268 | -0.0491366267465070 | 0.246089221556886 |
| 2110 | -0.0798996923076923 | -0.0216038722554890 | -0.580299500998004 | -0.103308742514970 |
| 2111 | -0.0406800000000000 | 0.140019081836327 | 0.634715269461078 | -0.220099600798403 |

| | | | | |
|---|---|---|---|---|
| 2112 | 0.0267027692307692 | -0.0391468662674651 | -0.120201996007984 | 0.111268063872255 |
| 2113 | 0.0623760000000000 | -0.174292894211577 | -1.25399920159681 | 0.0339489421157685 |
| 2114 | 0.0794824615384615 | -0.000162435129740519 | 0.556340319361277 | -0.193297804391218 |
| 2115 | 0.0853236923076923 | -0.0501924550898204 | 0.999788223552894 | 0.0425580039920160 |
| 2116 | 0.0947113846153846 | -0.275165109780439 | -1.14029461077844 | 0.187612574850299 |
| 2117 | 0.135391384615385 | -0.131085149700599 | -0.998976047904192 | -0.152526586826347 |
| 2118 | 0.117033230769231 | 0.145054570858283 | 1.27308532934132 | -0.217825508982036 |
| 2119 | 0.0461040000000000 | -0.0575020359281437 | 0.983950798403194 | 0.190536407185629 |
| 2120 | -0.0302492307692308 | -0.223348303393214 | -1.41724650698603 | 0.205155568862275 |
| 2121 | -0.0757273846153846 | -0.341438642714571 | -0.681415369261477 | -0.222048822355289 |
| 2122 | -0.0538227692307692 | -0.0334616367265469 | 1.27674011976048 | -0.0602634331337325 |
| 2123 | -0.0233649230769231 | 0.940012095808383 | 0.397559980039920 | 0.326007305389222 |
| 2124 | 0.00396369230769231 | 0.896317045908184 | -1.00263083832335 | 0.0492178443113772 |
| 2125 | 0.0166892307692308 | 0.0781312974051896 | -0.246901397205589 | -0.172830978043912 |
| 2126 | -0.00229476923076923 | -0.436138323353293 | 0.872276646706587 | 0.0927504590818363 |
| 2127 | -0.00751015384615385 | -0.311388143712575 | 0.0775627744510978 | 0.126049660678643 |
| 2128 | 0.0200270769230769 | 0.223023433133733 | -0.398778243512974 | -0.136445508982036 |
| 2129 | 0.0575778461538462 | 0.143592654690619 | 0.278170159680639 | -0.0570147305389222 |
| 2130 | 0.0531969230769231 | -0.621639241516966 | 0.336240718562874 | 0.185988223552894 |
| 2131 | 0.00542400000000000 | -0.705293333333333 | -0.150658582834331 | 0.0618877844311377 |
| 2132 | -0.0377593846153846 | 0.730308343313373 | 0.118983732534930 | -0.178841077844311 |
| 2133 | -0.0394283076923077 | 1.05923948103792 | 0.308626746506986 | 0.0428828742514970 |
| 2134 | 0.00354646153846154 | -0.593213093812375 | -0.338271157684631 | 0.252586626746507 |
| 2135 | 0.0296233846153846 | -0.763120239520958 | -0.0970549900199601 | -0.0766693812375250 |
| 2136 | 0.0262855384615385 | 0.536848103792415 | 0.422331337325349 | -0.154475808383234 |
| 2137 | 0.0129341538461538 | 0.226921876247505 | 0.144973353293413 | 0.236830419161677 |
| 2138 | 0.00146030769230769 | -0.651689740518962 | -0.445884431137725 | 0.198170858283433 |
| 2139 | 0.0171064615384615 | -0.268667704590818 | -0.105988922155689 | -0.168607664670659 |
| 2140 | 0.0354646153846154 | 0.0893393213572854 | 0.431265269461078 | -0.0318372854291417 |
| 2141 | 0.0348387692307692 | 0.0631872654690619 | -0.0864967065868263 | 0.151064670658683 |
| 2142 | -0.00980492307692308 | 0.443123033932136 | -0.436138323353293 | -0.0774815568862275 |
| 2143 | -0.0686344615384615 | 0.511345788423154 | 0.0751262475049900 | -0.195409461077844 |
| 2144 | -0.0719723076923077 | -0.134496287425150 | 0.346799001996008 | 0.114029461077844 |
| 2145 | -0.0335870769230769 | 0.0193297804391218 | -0.345580738522954 | 0.163897045908184 |
| 2146 | 0.0106393846153846 | 0.502574291417166 | -0.356951197604790 | -0.242840518962076 |
| 2147 | 0.0260769230769231 | -0.0575020359281437 | 0.302941516966068 | -0.108019361277445 |
| 2148 | 0.00813600000000000 | -0.436625628742515 | 0.321215469061876 | 0.275327544910180 |
| 2149 | -0.0123083076923077 | 0.108669101796407 | -0.370758183632735 | 0.0160810778443114 |
| 2150 | -0.0173150769230769 | 0.324545389221557 | -0.216850898203593 | -0.331854970059880 |
| 2151 | 0.00396369230769231 | -0.275165109780439 | 0.553091616766467 | 0.00958367265469062 |
| 2152 | 0.0340043076923077 | -0.232769540918164 | 0.135633333333333 | 0.184526307385230 |
| 2153 | 0.0317095384615385 | 0.319347465069860 | -0.407712175648703 | -0.188099880239521 |
| 2154 | -0.00292061538461538 | 0.0399590419161677 | -0.0678166666666667 | -0.135470898203593 |
| 2155 | -0.0287889230769231 | -0.301804471057884 | 0.300098902195609 | 0.215064111776447 |

| | | | | |
|---|---|---|---|---|
| 2156 | -0.0118910769230769 | -0.0914509780439122 | -0.0629436127744511 | 0.0459691417165669 |
| 2157 | 0.0337956923076923 | 0.0389844311377246 | -0.148628143712575 | -0.187612574850299 |
| 2158 | 0.0625846153846154 | -0.0578269061876247 | -0.0690349301397206 | 0.155937724550898 |
| 2159 | 0.0456867692307692 | -0.0397966067864271 | 0.0596949101796407 | 0.207754530938124 |
| 2160 | 0.0212787692307692 | -0.0302129341317365 | 0.265581437125749 | -0.188424750499002 |
| 2161 | 0.0212787692307692 | 0.0531162874251497 | 0.132790718562874 | -0.181115169660679 |
| 2162 | 0.0352560000000000 | 0.246576526946108 | -0.00203043912175649 | 0.162272694610778 |
| 2163 | 0.0540313846153846 | 0.0423955688622755 | -0.199795209580838 | 0.0968113373253493 |
| 2164 | 0.0469384615384615 | -0.237317724550898 | 0.136851596806387 | -0.155287984031936 |
| 2165 | 0.0189840000000000 | -0.0406087824351297 | 0.149440319361277 | 0.0232282235528942 |
| 2166 | -0.00521538461538462 | 0.318210419161677 | -0.151876846307385 | 0.170069580838323 |
| 2167 | -0.00959630769230769 | 0.148953013972056 | -0.190049101796407 | 0.0553903792415170 |
| 2168 | 0.0152289230769231 | -0.180302994011976 | 0.0791871257485030 | 0.0342738123752495 |
| 2169 | 0.0383852307692308 | -0.0484056686626747 | 0.337865069860279 | 0.0669232734530938 |
| 2170 | 0.0175236923076923 | 0.0352484231536926 | -0.159186427145709 | -0.00763445109780439 |
| 2171 | -0.0233649230769231 | -0.116303552894212 | -0.160404690618762 | 0.0877149700598802 |
| 2172 | -0.0590381538461538 | 0.0212790019960080 | 0.255835329341317 | 0.0904763672654691 |
| 2173 | -0.0634190769230769 | 0.139206906187625 | 0.191267365269461 | -0.157074770459082 |
| 2174 | -0.0191926153846154 | -0.162597564870259 | -0.299692814371258 | -0.0639994411177645 |
| 2175 | -0.00604984615384615 | -0.190211536926148 | -0.0791871257485030 | 0.136120638722555 |
| 2176 | -0.0421403076923077 | 0.106557445109780 | 0.266799700598802 | -0.0428828742514970 |
| 2177 | -0.0988836923076923 | 0.0185176047904192 | -0.172587325349301 | -0.231145189620758 |
| 2178 | -0.135182769230769 | -0.182577085828343 | -0.289946706586826 | 0.0407712175648703 |
| 2179 | -0.112860923076923 | 0.0396341716566866 | -0.0178678642714571 | 0.231145189620758 |
| 2180 | -0.0717636923076923 | 0.123613133732535 | 0.183145608782435 | -0.151389540918164 |
| 2181 | -0.0342129230769231 | -0.183389261477046 | 0.000812175648702595 | -0.293032974051896 |
| 2182 | -0.0264941538461538 | -0.0888520159680639 | -0.0479183632734531 | 0.178028902195609 |
| 2183 | -0.0502763076923077 | 0.217175768463074 | -0.0475122754491018 | 0.258434291417166 |
| 2184 | -0.0602898461538462 | -0.0782937325349302 | 0.0255835329341317 | -0.214901676646707 |
| 2185 | -0.0415144615384615 | -0.237317724550898 | 0.208729141716567 | -0.262007864271457 |
| 2186 | -0.0196098461538462 | 0.299855249500998 | -0.0211165668662675 | 0.0542533333333333 |
| 2187 | -0.0394283076923077 | 0.321621556886228 | -0.376849500998004 | 0.0789434730538922 |
| 2188 | -0.0784393846153846 | -0.389194570858283 | -0.118171556886228 | -0.0747201596806387 |
| 2189 | -0.0955458461538462 | -0.319672335329341 | 0.337865069860279 | -0.0578269061876247 |
| 2190 | -0.0834461538461538 | 0.338189940119761 | -0.0296444111776447 | -0.0172181237524950 |
| 2191 | -0.0598726153846154 | 0.123125828343313 | -0.351265968063872 | -0.0332992015968064 |
| 2192 | -0.0452695384615385 | -0.183064391217565 | -0.0357357285429142 | 0.122800958083832 |
| 2193 | -0.0327526153846154 | -0.0290758882235529 | 0.387001696606786 | 0.165034091816367 |
| 2194 | -0.0379680000000000 | -0.0917758483033932 | 0.120608083832335 | -0.134171417165669 |
| 2195 | -0.0344215384615385 | -0.262657604790419 | -0.221317864271457 | -0.158211816367265 |
| 2196 | -0.0102221538461538 | -0.178516207584830 | 0.0556340319361277 | 0.226759441117764 |
| 2197 | 0.0143944615384615 | 0.210028622754491 | 0.228221357285429 | 0.256972375249501 |
| 2198 | 0.0300406153846154 | 0.493153053892216 | 0.0397966067864271 | -0.170394451097804 |
| 2199 | 0.00584123076923077 | 0.315611457085828 | -0.0889332335329341 | -0.256972375249501 |

| | | | | |
|---|---|---|---|---|
| 2200 | -0.0296233846153846 | 0.0497051497005988 | 0.0519792415169661 | 0.191511017964072 |
| 2201 | -0.0406800000000000 | 0.0391468662674651 | 0.0353296407185629 | 0.285073652694611 |
| 2202 | -0.0271200000000000 | -0.543183073852295 | -0.0349235528942116 | -0.0903139321357285 |
| 2203 | 0.0114738461538462 | -0.314799281437126 | 0.0198983033932136 | -0.138232295409182 |
| 2204 | 0.0216960000000000 | 0.621314371257485 | -0.0373600798403194 | 0.116628423153693 |
| 2205 | -0.00709292307692308 | -0.361743033932136 | 0.189236926147705 | 0.154800678642715 |
| 2206 | -0.0160633846153846 | -0.437275369261477 | 0.481214071856287 | 0.0103958483033932 |
| 2207 | -0.0135600000000000 | 1.10293453093812 | -0.857657485029940 | 0.00958367265469062 |
| 2208 | 0.00229476923076923 | 0.509721437125749 | -1.00994041916168 | 0.0219287425149701 |
| 2209 | 0.0154375384615385 | -0.812338083832335 | 1.41277954091816 | -0.100384910179641 |
| 2210 | 0.00312923076923077 | -0.168607664670659 | 0.760602495009980 | -0.0467813173652695 |
| 2211 | -0.0350473846153846 | 0.531162874251497 | -2.23795000000000 | 0.0665984031936128 |
| 2212 | -0.0736412307692308 | -0.409011656686627 | -0.319185029940120 | -0.112567544910180 |
| 2213 | -0.0521538461538462 | -0.716501357285429 | 2.61033253493014 | -0.192972934131737 |
| 2214 | -0.00292061538461538 | 0.275652415169661 | -0.557558582834331 | 0.0295631936127745 |
| 2215 | 0.0365076923076923 | 0.260058642714571 | -2.03165738522954 | 0.0976235129740519 |
| 2216 | 0.0440178461538462 | -0.224322914171657 | 1.00181866267465 | -0.0199795209580838 |
| 2217 | 0.0342129230769231 | 0.349235528942116 | 1.28120708582834 | -0.0542533333333333 |
| 2218 | 0.0365076923076923 | 0.298555768463074 | -0.924661976047904 | 0.00292383233532934 |
| 2219 | 0.0227390769230769 | -0.536035928143713 | -0.320809381237525 | 0.0539284630738523 |
| 2220 | 0.0227390769230769 | 0.162597564870259 | 0.894611477045908 | 0.0609131736526946 |
| 2221 | 0.0166892307692308 | 0.746389421157685 | -0.778876447105788 | 0.0568522954091816 |
| 2222 | -0.0196098461538462 | -0.279063552894212 | -0.0885271457085828 | -0.0246901397205589 |
| 2223 | -0.0410972307692308 | -0.404463473053892 | 1.27958273453094 | -0.0225784830339321 |
| 2224 | -0.0340043076923077 | 0.518655369261477 | -0.622126546906188 | 0.0755323353293413 |
| 2225 | 0.0156461538461538 | 0.411610618762475 | -1.19755299401198 | 0.0318372854291417 |
| 2226 | 0.0529883076923077 | -0.356545109780439 | 1.07775708582834 | 0.0160810778443114 |
| 2227 | 0.0483987692307692 | -0.174617764471058 | 1.14273113772455 | 0.0449945309381238 |
| 2228 | 0.0277458461538462 | 0.235855808383234 | -1.24872005988024 | -0.00373600798403194 |
| 2229 | 0.0160633846153846 | -0.0487305389221557 | -0.789434730538922 | -0.0155937724550898 |
| 2230 | 0.0431833846153846 | -0.0485681037924152 | 1.05095528942116 | 0.0875525349301397 |
| 2231 | 0.0715550769230769 | 0.0815424351297405 | 0.581517764471058 | 0.127349141716567 |
| 2232 | 0.0786480000000000 | -0.0968113373253493 | -1.05379790419162 | -0.00227409181636727 |
| 2233 | 0.0519452307692308 | -0.0753699001996008 | -0.448727045908184 | -0.103796047904192 |
| 2234 | 0.0112652307692308 | 0.0155937724550898 | 0.908824550898204 | 0.0256647504990020 |
| 2235 | -0.00229476923076923 | -0.0498675848303393 | 0.0889332335329341 | 0.140831257485030 |
| 2236 | 0.00292061538461538 | -0.0700095409181637 | -0.411773053892216 | -0.0708217165668663 |
| 2237 | 0.0342129230769231 | -0.105745269461078 | 0.202231736526946 | -0.106232574850299 |
| 2238 | 0.0604984615384615 | -0.158536686626747 | 0.406493912175649 | 0.114679201596806 |
| 2239 | 0.0694689230769231 | 0.0332992015968064 | -0.219287425149701 | 0.0355732934131737 |
| 2240 | 0.0861581538461538 | 0.264931696606786 | -0.195328243512974 | -0.138557165668663 |
| 2241 | 0.119745230769231 | 0.238779640718563 | 0.382534730538922 | -0.0441823552894212 |
| 2242 | 0.173985230769231 | -0.0123450698602794 | -0.0877149700598802 | 0.0646491816367266 |
| 2243 | 0.204443076923077 | -0.00129948103792415 | -0.359387724550898 | -0.00324870259481038 |

| | | | | |
|---|---|---|---|---|
| 2244 | 0.186084923076923 | 0.144242395209581 | 0.133196806387226 | -0.0579893413173653 |
| 2245 | 0.139980923076923 | 0.0131572455089820 | 0.191267365269461 | -0.00552279441117765 |
| 2246 | 0.0997181538461539 | -0.0294007584830339 | -0.241216167664671 | 0.0160810778443114 |
| 2247 | 0.0907476923076923 | 0.0332992015968064 | 0.0637557884231537 | -0.0508421956087824 |
| 2248 | 0.0982578461538461 | 0.0536035928143713 | 0.353296407185629 | -0.0105582834331337 |
| 2249 | 0.108062769230769 | -0.0194922155688623 | -0.0840601796407186 | 0.0253398802395210 |
| 2250 | 0.0936683076923077 | -0.0787810379241517 | -0.202637824351297 | 0.00487305389221557 |
| 2251 | 0.0596640000000000 | -0.0201419560878244 | 0.0686288423153693 | 0.0732582435129741 |
| 2252 | 0.0642535384615385 | -0.210678363273453 | 0.143755089820359 | 0.0451569660678643 |
| 2253 | 0.0959630769230769 | -0.236018243512974 | -0.244464870259481 | -0.0747201596806387 |
| 2254 | 0.133305230769231 | 0.0795932135728543 | -0.322433732534930 | 0.00665984031936128 |
| 2255 | 0.146239384615385 | 0.0246901397205589 | 0.0885271457085828 | 0.142780479041916 |
| 2256 | 0.118284923076923 | -0.248200878243513 | 0.214414371257485 | -0.0821921756487026 |
| 2257 | 0.0694689230769231 | -0.103146307385230 | -0.153907285429142 | -0.202231736526946 |
| 2258 | 0.0444350769230769 | 0.167470618762475 | -0.227815269461078 | 0.0930753293413174 |
| 2259 | 0.0709292307692308 | -0.0820297405189621 | 0.231470059880240 | 0.0828419161676647 |
| 2260 | 0.0999267692307692 | -0.154475808383234 | 0.310657185628743 | -0.276302155688623 |
| 2261 | 0.104516307692308 | -0.0341113772455090 | -0.0531975049900200 | -0.162272694610778 |
| 2262 | 0.0761446153846154 | 0.0307002395209581 | -0.248525748502994 | 0.207429660678643 |
| 2263 | 0.0392196923076923 | 0.0391468662674651 | 0.131572455089820 | 0.00763445109780439 |
| 2264 | 0.0166892307692308 | -0.0168932534930140 | 0.181115169660679 | -0.283611736526946 |
| 2265 | 0.00563261538461538 | 0.166008702594810 | -0.190455189620759 | 0.0133196806387226 |
| 2266 | 0.00897046153846154 | 0.141156127744511 | -0.0698471057884232 | 0.293032974051896 |
| 2267 | 0.00730153846153846 | 0.128648622754491 | 0.0121826347305389 | -0.0126699401197605 |
| 2268 | -0.0127255384615385 | 0.106232574850299 | 0.0954306387225549 | -0.123125828343313 |
| 2269 | -0.0166892307692308 | -0.0578269061876247 | 0.0726897205588822 | 0.214576806387226 |
| 2270 | 0.0267027692307692 | -0.0927504590818363 | -0.0467000998003992 | 0.108344231536926 |
| 2271 | 0.0673827692307692 | 0.218637684630739 | -0.0239591816367265 | -0.104283353293413 |
| 2272 | 0.0709292307692308 | 0.258921596806387 | 0.107613273453094 | 0.157399640718563 |
| 2273 | 0.0479815384615385 | -0.294170019960080 | 0.133196806387226 | 0.148140838323353 |
| 2274 | 0.00250338461538462 | -0.561700678642715 | -0.228627445109780 | -0.220586906187625 |
| 2275 | -0.0150203076923077 | -0.206130179640719 | -0.261520558882236 | -0.0909636726546906 |
| 2276 | 0.0104307692307692 | 0.233256846307385 | -0.0105582834331337 | 0.249337924151697 |
| 2277 | 0.0321267692307692 | 0.0856033133732535 | 0.0592888223552894 | 0.0170556886227545 |
| 2278 | 0.0310836923076923 | -0.111917804391218 | -0.0913697604790419 | -0.310738403193613 |
| 2279 | -0.00688430769230769 | -0.140506387225549 | -0.108425449101796 | -0.00568522954091816 |
| 2280 | -0.0496504615384615 | 0.430615528942116 | 0.0661923153692615 | 0.256160199600798 |
| 2281 | -0.0546572307692308 | 0.891606427145709 | 0.319591117764471 | -0.174455329341317 |
| 2282 | -0.0273286153846154 | -0.0831667864271457 | 0.203043912175649 | -0.291895928143713 |
| 2283 | 0.0156461538461538 | -0.609456606786427 | -0.270860578842315 | 0.130760279441118 |
| 2284 | 0.0244080000000000 | 0.152364151696607 | 0.0312687624750499 | 0.0539284630738523 |
| 2285 | 0.00354646153846154 | 0.536360798403194 | 0.417052195608782 | -0.236180678642715 |
| 2286 | -0.0114738461538462 | -0.131085149700599 | -0.0698471057884232 | 0.0264769261477046 |
| 2287 | 0.000834461538461538 | -0.257946986027944 | -0.428422654690619 | 0.204343393213573 |

| | | | | |
|---|---|---|---|---|
| 2288 | 0.0323353846153846 | 0.415021756487026 | 0.0312687624750499 | -0.121501477045908 |
| 2289 | 0.0350473846153846 | 0.293195409181637 | 0.304565868263473 | -0.116628423153693 |
| 2290 | 0.00980492307692308 | 0.0357357285429142 | -0.169744710578842 | 0.296281676646707 |
| 2291 | -0.0402627692307692 | 0.0518168063872256 | -0.322027644710579 | 0.185500918163673 |
| 2292 | -0.0786480000000000 | -0.341601077844311 | 0.199795209580838 | -0.253073932135729 |
| 2293 | -0.0604984615384615 | -0.684176766467066 | 0.282231037924152 | -0.0212790019960080 |
| 2294 | -0.00959630769230769 | -0.287022874251497 | -0.133196806387226 | 0.383184471057884 |
| 2295 | 0.0256596923076923 | 0.132547065868263 | -0.102740219560878 | 0.0976235129740519 |
| 2296 | 0.0143944615384615 | -0.0875525349301397 | 0.110455888223553 | -0.192323193612774 |
| 2297 | 0.00104307692307692 | -0.0100709780439122 | -0.0231470059880240 | 0.131410019960080 |
| 2298 | 0.0198184615384615 | 0.414372015968064 | -0.140100299401198 | 0.270779361277445 |
| 2299 | 0.0294147692307692 | 0.275165109780439 | -0.0909636726546906 | -0.100384910179641 |
| 2300 | 0.0214873846153846 | -0.0423955688622755 | 0.0227409181636727 | -0.147491097804391 |
| 2301 | 0.0146030769230769 | 0.0841413972055888 | 0.0986793413173653 | 0.116628423153693 |
| 2302 | -0.00855323076923077 | 0.0898266267465070 | 0.0852784431137725 | 0.0869027944111777 |
| 2303 | -0.0383852307692308 | -0.143267784431138 | 0.00934001996007984 | -0.0817048702594810 |
| 2304 | -0.0335870769230769 | -0.162110259481038 | 0.00812175648702595 | -0.0344362475049900 |
| 2305 | 0.0116824615384615 | 0.245601916167665 | 0.114922854291417 | 0.0639994411177645 |
| 2306 | 0.0241993846153846 | 0.421194291417166 | 0.0601009980039920 | 0.0217663073852295 |
| 2307 | -0.00375507692307692 | 0.0758572055888224 | -0.0426392215568862 | 0.00324870259481038 |
| 2308 | -0.0385938461538462 | 0.0133196806387226 | -0.0978671656686627 | 0.0167308183632735 |
| 2309 | -0.0592467692307692 | 0.0578269061876247 | -0.0381722554890220 | -0.0501924550898204 |
| 2310 | -0.0394283076923077 | -0.0656237924151697 | -0.0134008982035928 | -0.0199795209580838 |
| 2311 | -0.0104307692307692 | -0.347123872255489 | -0.0235530938123753 | 0.0524665469061876 |
| 2312 | 0.0164806153846154 | -0.466676127744511 | -0.0320809381237525 | -0.0440199201596806 |
| 2313 | -0.00166892307692308 | -0.208729141716567 | -0.0873088822355290 | -0.0631872654690619 |
| 2314 | -0.0392196923076923 | -0.0396341716566866 | 0.221317864271457 | 0.0678978842315369 |
| 2315 | -0.0398455384615385 | 0.0449945309381238 | 0.0381722554890220 | 0.0875525349301397 |
| 2316 | -0.0123083076923077 | 0.143755089820359 | -0.000812175648702595 | -0.0596136926147705 |
| 2317 | 0.0200270769230769 | 0.0134821157684631 | 0.0560401197604790 | -0.0914509780439122 |
| 2318 | 0.0281630769230769 | -0.0969737724550898 | -0.0747201596806387 | 0.0756947704590818 |
| 2319 | 0.0158547692307692 | 0.134171417165669 | 0.479589720558882 | 0.0956742914171657 |
| 2320 | -0.0235735384615385 | 0.196708942115768 | -0.0621314371257485 | 0.00438574850299401 |
| 2321 | -0.0327526153846154 | 0.0321621556886228 | -1.05623443113772 | -0.0784561676646707 |
| 2322 | -0.0183581538461538 | -0.0648116167664671 | -0.431671357285429 | -0.100709780439122 |
| 2323 | -0.0152289230769231 | 0.0298880638722555 | 1.63815828343313 | 0.0490554091816367 |
| 2324 | -0.0173150769230769 | 0.158049381237525 | -0.0365479041916168 | 0.0290758882235529 |
| 2325 | -0.0287889230769231 | 0.0146191616766467 | -1.99104860279441 | -0.194272415169661 |
| 2326 | -0.0135600000000000 | -0.118252774451098 | 0.998976047904192 | -0.173155848303393 |
| 2327 | 0.0127255384615385 | -0.142455608782435 | 1.69054361277445 | 0.0482432335329341 |
| 2328 | 0.0569520000000000 | -0.0906388023952096 | -1.03836656686627 | 0.0331367664670659 |
| 2329 | 0.0894960000000000 | -0.0859281836327345 | -1.49683972055888 | -0.108669101796407 |
| 2330 | 0.0621673846153846 | -0.0846287025948104 | 1.44932744510978 | -0.0407712175648703 |
| 2331 | 0.0164806153846154 | 0.0826794810379242 | 0.686288423153693 | 0.0648116167664671 |

| | | | | |
|---|---|---|---|---|
| 2332 | -0.0164806153846154 | 0.0740704191616766 | -1.25968443113772 | 0.0271266666666667 |
| 2333 | 0.00292061538461538 | -0.0562025548902196 | 0.311469361277445 | 0.0607507385229541 |
| 2334 | 0.0471470769230769 | 0.00828419161676647 | 0.490148003992016 | 0.148140838323353 |
| 2335 | 0.0483987692307692 | 0.0971362075848303 | -0.312687624750499 | -0.0550655089820359 |
| 2336 | 0.0381766153846154 | -0.0864154890219561 | 0.0998976047904192 | -0.125399920159681 |
| 2337 | 0.0187753846153846 | -0.208404271457086 | 0.761008582834331 | 0.216201157684631 |
| 2338 | -0.00479815384615385 | 0.236180678642715 | -0.767099900199601 | 0.235855808383234 |
| 2339 | 0.0100135384615385 | 0.383184471057884 | -0.516949800399202 | -0.198820598802395 |
| 2340 | 0.0525710769230769 | -0.114679201596806 | 1.29704451097804 | -0.181440039920160 |
| 2341 | 0.0680086153846154 | -0.0763445109780439 | -0.0885271457085828 | 0.262332734530938 |
| 2342 | 0.0160633846153846 | 0.350535009980040 | -1.39450558882236 | 0.138557165668663 |
| 2343 | -0.0404713846153846 | 0.0480807984031936 | -0.0917758483033932 | -0.231145189620758 |
| 2344 | -0.0621673846153846 | -0.181764910179641 | 1.33765329341317 | -0.0428828742514970 |
| 2345 | -0.0396369230769231 | 0.102821437125749 | -0.378067764471058 | 0.197033812375250 |
| 2346 | -0.00458953846153846 | 0.159023992015968 | -1.39206906187625 | -0.0547406387225549 |
| 2347 | 0.00604984615384615 | 0.281337644710579 | 0.433295708582834 | -0.197683552894212 |
| 2348 | 0.00563261538461538 | -0.397803632734531 | 1.03471177644711 | -0.000324870259481038 |
| 2349 | -0.0135600000000000 | -1.01521956087824 | -0.147003792415170 | -0.00503548902195609 |
| 2350 | -0.00458953846153846 | -0.250150099800399 | -0.657456187624751 | -0.0859281836327345 |
| 2351 | 0.0202356923076923 | 0.316423632734531 | 0.472686227544910 | 0.0901514970059880 |
| 2352 | 0.0177323076923077 | 0.305215608782435 | 0.482026247504990 | 0.0328118962075848 |
| 2353 | -0.0204443076923077 | 0.0956742914171657 | -0.294819760479042 | -0.260221077844311 |
| 2354 | -0.0713464615384615 | -0.162760000000000 | -0.212383932135729 | 0.0186800399201597 |
| 2355 | -0.0834461538461538 | 0.181115169660679 | 0.0174617764471058 | 0.292870538922156 |
| 2356 | -0.0625846153846154 | 0.787647944111776 | -0.0507609780439122 | -0.138882035928144 |
| 2357 | -0.0123083076923077 | -0.164709221556886 | -0.0779688622754491 | -0.220586906187625 |
| 2358 | 0.0233649230769231 | -1.14679201596806 | 0.233500499001996 | 0.254860718562874 |
| 2359 | 0.0277458461538462 | 0.220262035928144 | -0.133196806387226 | 0.210191057884232 |
| 2360 | 0.0254510769230769 | 1.15004071856287 | -0.169338622754491 | -0.197196247504990 |
| 2361 | 0.0325440000000000 | -0.100060039920160 | 0.337458982035928 | -0.121826347305389 |
| 2362 | 0.0700947692307692 | -0.546106906187625 | 0.374006886227545 | 0.238292335329341 |
| 2363 | 0.0863667692307692 | 0.532137485029940 | -0.265175349301397 | 0.177379161676647 |
| 2364 | 0.0467298461538462 | 0.483731816367266 | -0.434107884231537 | -0.110618323353293 |
| 2365 | -0.0118910769230769 | -0.359468942115768 | 0.234312674650699 | 0.0425580039920160 |
| 2366 | -0.0630018461538462 | -0.279063552894212 | 0.161216866267465 | 0.258921596806387 |
| 2367 | -0.0811513846153846 | -0.165196526946108 | -0.304159780439122 | 0.0370352095808383 |
| 2368 | -0.0488160000000000 | -0.521579201596806 | -0.225784830339321 | -0.0581517764471058 |
| 2369 | 0.00542400000000000 | 0.0495427145708583 | 0.379692115768463 | 0.131572455089820 |
| 2370 | 0.0521538461538462 | 0.435813453093812 | 0.147815968063872 | 0.134496287425150 |
| 2371 | 0.0663396923076923 | -0.326007305389222 | -0.218475249500998 | -0.0555528143712575 |
| 2372 | 0.0796910769230769 | -0.0612380439121757 | 0.113704590818363 | -0.138719600798403 |
| 2373 | 0.0988836923076923 | 0.659811497005988 | 0.332992015968064 | -0.0107207185628743 |
| 2374 | 0.112443692307692 | 0.0225784830339321 | 0.0300504990019960 | 0.0427204391217565 |
| 2375 | 0.106393846153846 | -0.524340598802395 | -0.348423353293413 | -0.142780479041916 |

| | | | | |
|---|---|---|---|---|
| 2376 | 0.0548658461538462 | 0.167633053892216 | 0.124668962075848 | -0.100384910179641 |
| 2377 | 0.00458953846153846 | 0.406087824351297 | 0.203856087824351 | 0.147653532934132 |
| 2378 | -0.0327526153846154 | -0.262332734530938 | -0.153501197604790 | -0.00763445109780439 |
| 2379 | -0.0150203076923077 | -0.182252215568862 | -0.195734331337325 | -0.191673453093812 |
| 2380 | 0.0388024615384615 | 0.386108303393214 | 0.136851596806387 | 0.00487305389221557 |
| 2381 | 0.0711378461538462 | 0.266718483033932 | 0.248931836327345 | 0.144079960079840 |
| 2382 | 0.103890461538462 | -0.0508421956087824 | 0.0272078842315369 | -0.0354108582834331 |
| 2383 | 0.0919993846153846 | 0.104608223552894 | 0.0950245508982036 | -0.175592375249501 |
| 2384 | 0.0719723076923077 | 0.119714690618762 | -0.0442635728542914 | 0.0443447904191617 |
| 2385 | 0.0725981538461538 | -0.175917245508982 | 0.111268063872255 | 0.155125548902196 |
| 2386 | 0.0538227692307692 | -0.143917524950100 | 0.212383932135729 | -0.117440598802395 |
| 2387 | 0.0191926153846154 | -0.282312255489022 | -0.101115868263473 | -0.154150938123753 |
| 2388 | -0.0577864615384615 | -0.353621277445110 | -0.376443413173653 | 0.115491377245509 |
| 2389 | -0.108688615384615 | 0.195734331337325 | -0.210353493013972 | 0.0805678243512974 |
| 2390 | -0.0924166153846154 | 0.265581437125749 | 0.179084730538922 | -0.107694491017964 |
| 2391 | -0.0406800000000000 | -0.276302155688623 | -0.0913697604790419 | 0.00698471057884232 |
| 2392 | 0.0329612307692308 | -0.210678363273453 | -0.222942215568862 | 0.166658443113772 |
| 2393 | 0.0715550769230769 | 0.337052894211577 | -0.0296444111776447 | 0.0162435129740519 |
| 2394 | 0.0740584615384615 | 0.202069301397206 | 0.215226546906188 | -0.109156407185629 |
| 2395 | 0.0602898461538462 | -0.130272974051896 | 0.164059481037924 | -0.0103958483033932 |
| 2396 | 0.0717636923076923 | -0.0336240718562874 | -0.174617764471058 | -0.00113704590818363 |
| 2397 | 0.0947113846153846 | -0.0272891017964072 | -0.0442635728542914 | -0.0493802794411178 |
| 2398 | 0.0650880000000000 | -0.129298363273453 | 0.185988223552894 | -0.000162435129740519 |
| 2399 | -0.000834461538461538 | -0.0128323752495010 | 0.0531975049900200 | 0.0233906586826347 |
| 2400 | -0.0863667692307692 | 0.0812175648702595 | -0.235937025948104 | -0.00893393213572854 |
| 2401 | -0.117867692307692 | -0.0342738123752495 | 0.0483244510978044 | 0.0477559281437126 |
| 2402 | -0.0884529230769231 | 0.0289134530938124 | 0.288728443113772 | 0.154150938123753 |
| 2403 | -0.0483987692307692 | 0.182089780439122 | -0.125481137724551 | 0.0415833932135729 |
| 2404 | -0.00187753846153846 | 0.0389844311377246 | -0.149440319361277 | -0.107694491017964 |
| 2405 | 0.0179409230769231 | -0.183876566866267 | 0.228627445109780 | 0.0786186027944112 |
| 2406 | 0.0477729230769231 | 0.0274515369261477 | 0.116547205588822 | 0.211652974051896 |
| 2407 | 0.0803169230769231 | 0.149277884231537 | -0.261926646706587 | -0.0397966067864271 |
| 2408 | 0.0970061538461539 | -0.169744710578842 | -0.146191616766467 | -0.153663632734531 |
| 2409 | 0.0863667692307692 | -0.128811057884232 | -0.00121826347305389 | 0.100709780439122 |
| 2410 | 0.0123083076923077 | 0.165521397205589 | -0.119389820359281 | 0.165521397205589 |
| 2411 | -0.0682172307692308 | 0.0791059081836327 | -0.114516766467066 | -0.0768318163672655 |
| 2412 | -0.128089846153846 | -0.161135648702595 | -0.131978542914172 | -0.123775568862275 |
| 2413 | -0.115364307692308 | 0.0648116167664671 | -0.203043912175649 | 0.0579893413173653 |
| 2414 | -0.0596640000000000 | 0.313824670658683 | 0.0755323353293413 | 0.0258271856287425 |
| 2415 | -0.0191926153846154 | -0.0337865069860280 | 0.286698003992016 | -0.118090339321357 |
| 2416 | 0.0244080000000000 | -0.269642315369262 | -0.0442635728542914 | -0.0399590419161677 |
| 2417 | 0.0296233846153846 | -0.0458067065868264 | -0.170150798403194 | 0.0263144910179641 |
| 2418 | 0.0362990769230769 | 0.0612380439121757 | 0.188424750499002 | -0.0553903792415170 |
| 2419 | 0.0619587692307692 | 0.0969737724550898 | 0.279388423153693 | 0.0110455888223553 |

| | | | | |
|---|---|---|---|---|
| 2420 | 0.0644621538461539 | 0.208566706586826 | -0.143755089820359 | 0.103146307385230 |
| 2421 | 0.0315009230769231 | 0.0813800000000000 | -0.192485628742515 | -0.0742328542914172 |
| 2422 | -0.0498590769230769 | 0.107369620758483 | 0.105176746506986 | -0.163084870259481 |
| 2423 | -0.114321230769231 | 0.0740704191616766 | 0.102334131736527 | 0.0190049101796407 |
| 2424 | -0.117867692307692 | -0.384971257485030 | -0.0787810379241517 | 0.0635121357285429 |
| 2425 | -0.0909563076923077 | -0.424930299401198 | -0.0263957085828343 | -0.0851160079840319 |
| 2426 | -0.0436006153846154 | 0.0756947704590818 | 0.0568522954091816 | -0.0188424750499002 |
| 2427 | -0.00709292307692308 | 0.319997205588822 | 0.0657862275449102 | 0.118577644710579 |
| 2428 | -0.000208615384615385 | 0.00828419161676647 | -0.0877149700598802 | -0.0233906586826347 |
| 2429 | -0.00897046153846154 | -0.165358962075848 | 0.0572583832335329 | -0.0123450698602794 |
| 2430 | -0.0154375384615385 | 0.106395009980040 | 0.00934001996007984 | 0.196708942115768 |
| 2431 | -0.00771876923076923 | 0.139856646706587 | -0.0170556886227545 | 0.0326494610778443 |
| 2432 | -0.0319181538461538 | -0.294170019960080 | 0.572177744510978 | -0.166658443113772 |
| 2433 | -0.0930424615384616 | -0.461478203592814 | -0.216444810379242 | 0.112080239520958 |
| 2434 | -0.144570461538462 | 0.0807302594810379 | -1.12486327345309 | 0.266231177644711 |
| 2435 | -0.155209846153846 | 0.524340598802395 | -0.0966489021956088 | -0.0937250698602794 |
| 2436 | -0.111817846153846 | 0.172830978043912 | 1.92160758483034 | -0.202719041916168 |
| 2437 | -0.0410972307692308 | -0.144242395209581 | -0.352890319361277 | 0.181440039920160 |
| 2438 | 0.00730153846153846 | 0.106232574850299 | -2.00363732534930 | 0.192323193612774 |
| 2439 | 0.0223218461538462 | 0.293845149700599 | 1.38516556886228 | -0.216038722554890 |
| 2440 | 0.0233649230769231 | 0.154800678642715 | 1.68567055888224 | -0.115816247504990 |
| 2441 | 0.0196098461538462 | -0.117278163672655 | -1.13745199600798 | 0.246089221556886 |
| 2442 | 0.0175236923076923 | -0.168120359281437 | -1.67714271457086 | 0.0750450299401198 |
| 2443 | -0.00834461538461538 | 0.118577644710579 | 1.33562285429142 | -0.184039001996008 |
| 2444 | -0.0671741538461538 | 0.438574850299401 | 0.846693113772455 | 0.00909636726546906 |
| 2445 | -0.139772307692308 | 0.254210978043912 | -0.939687225548902 | 0.172181237524950 |
| 2446 | -0.197350153846154 | -0.374575409181637 | 0.105582834331337 | -0.0704968463073852 |
| 2447 | -0.188379692307692 | -0.392118403193613 | 0.0808114770459082 | -0.182901956087824 |
| 2448 | -0.113278153846154 | 0.192160758483034 | -0.0121826347305389 | 0.0680603193612775 |
| 2449 | -0.0279544615384615 | 0.0557152495009980 | 0.494208882235529 | 0.110131017964072 |
| 2450 | 0.0233649230769231 | -0.509559001996008 | 0.597355189620758 | -0.136445508982036 |
| 2451 | 0.0296233846153846 | -0.237967465069860 | -1.18333992015968 | -0.0940499401197605 |
| 2452 | 0.0241993846153846 | 0.257946986027944 | -0.512076746506986 | 0.112567544910180 |
| 2453 | 0.0352560000000000 | 0.0614004790419162 | 1.45582485029940 | 0.0523041117764471 |
| 2454 | 0.0404713846153846 | -0.147815968063872 | 0.00121826347305389 | -0.0932377644710579 |
| 2455 | 0.0264941538461538 | 0.0154313373253493 | -1.38232295409182 | -0.0479183632734531 |
| 2456 | -0.0262855384615385 | -0.113542155688623 | -0.326494610778443 | 0.100222475049900 |
| 2457 | -0.0801083076923077 | -0.168607664670659 | 1.39450558882236 | 0.0235530938123753 |
| 2458 | -0.0842806153846154 | 0.192323193612774 | 0.134008982035928 | -0.131247584830339 |
| 2459 | -0.0425575384615385 | 0.278413812375250 | -1.24384700598802 | -0.0462940119760479 |
| 2460 | 0.0342129230769231 | 0.0563649900199601 | -0.103146307385230 | 0.0688724950099800 |
| 2461 | 0.0849064615384615 | -0.0820297405189621 | 0.838165269461078 | 0.0112080239520958 |
| 2462 | 0.0997181538461539 | 0.107369620758483 | 0.191267365269461 | -0.0586390818363273 |
| 2463 | 0.0915821538461538 | 0.205480439121756 | -0.692379740518962 | -0.0121826347305389 |

| | | | | |
|---|---|---|---|---|
| 2464 | 0.0861581538461538 | -0.0612380439121757 | 0.142536826347305 | 0.0227409181636727 |
| 2465 | 0.110357538461538 | -0.132222195608782 | 0.413803493013972 | -0.0451569660678643 |
| 2466 | 0.109940307692308 | -0.0352484231536926 | -0.170962974051896 | -0.0440199201596806 |
| 2467 | 0.0650880000000000 | -0.0545782035928144 | -0.0641618762475050 | 0.0488929740518962 |
| 2468 | -0.00584123076923077 | 0.0875525349301397 | 0.300504990019960 | 0.0297256287425150 |
| 2469 | -0.0481901538461539 | 0.214901676646707 | 0.138882035928144 | -0.0133196806387226 |
| 2470 | -0.0216960000000000 | -0.00633497005988024 | -0.225784830339321 | 0.0178678642714571 |
| 2471 | 0.0481901538461539 | -0.0797556487025948 | 0.235937025948104 | 0.0349235528942116 |
| 2472 | 0.120579692307692 | 0.0274515369261477 | 0.174617764471058 | 0.0430453093812375 |
| 2473 | 0.144570461538462 | -0.0880398403193613 | -0.135227245508982 | 0.0168932534930140 |
| 2474 | 0.108897230769231 | -0.241541037924152 | 0.0272078842315369 | 0.0157562075848303 |
| 2475 | 0.0778135384615385 | -0.117115728542914 | 0.159592514970060 | 0.0458067065868264 |
| 2476 | 0.0786480000000000 | 0.0945372455089820 | -0.116547205588822 | -0.00795932135728543 |
| 2477 | 0.0886615384615385 | 0.0869027944111777 | -0.300098902195609 | -0.00292383233532934 |
| 2478 | 0.0684258461538462 | 0.113542155688623 | 0.0592888223552894 | 0.0691973652694611 |
| 2479 | 0 | 0.185988223552894 | 0.0523853293413174 | 0.00324870259481038 |
| 2480 | -0.0634190769230769 | 0.0410960878243513 | -0.226597005988024 | -0.0823546107784431 |
| 2481 | -0.0755187692307692 | -0.0638370059880239 | -0.112486327345309 | -0.0155937724550898 |
| 2482 | -0.00834461538461538 | -0.00649740518962076 | 0.293195409181637 | 0.0701719760479042 |
| 2483 | 0.0659224615384615 | -0.0552279441117765 | 0.0471061876247505 | 0.0386595608782435 |
| 2484 | 0.106185230769231 | -0.0526289820359282 | -0.303753692614771 | -0.118090339321357 |
| 2485 | 0.123291692307692 | 0.0971362075848303 | -0.202637824351297 | -0.0750450299401198 |
| 2486 | 0.0999267692307692 | 0.0795932135728543 | 0.275327544910180 | 0.123775568862275 |
| 2487 | 0.0869926153846154 | -0.0129948103792415 | 0.192079540918164 | -0.0302129341317365 |
| 2488 | 0.0531969230769231 | 0.00909636726546906 | -0.499488023952096 | -0.172830978043912 |
| 2489 | 0.0108480000000000 | 0.0391468662674651 | -0.224160479041916 | -0.0341113772455090 |
| 2490 | -0.0327526153846154 | -0.0456442714570858 | 0.365479041916168 | 0.0233906586826347 |
| 2491 | -0.0976320000000000 | -0.155775289421158 | 0.199795209580838 | -0.0277764071856287 |
| 2492 | -0.107019692307692 | -0.166171137724551 | -0.389438223552894 | -0.00828419161676647 |
| 2493 | -0.0788566153846154 | -0.0523041117764471 | -0.0771566866267465 | 0.0690349301397206 |
| 2494 | -0.0141858461538462 | 0.173155848303393 | 0.319591117764471 | 0.0886895808383234 |
| 2495 | 0.0469384615384615 | 0.113867025948104 | 0.0105582834331337 | 0.0461315768463074 |
| 2496 | 0.0456867692307692 | -0.101197085828343 | -0.239997904191617 | 0.0264769261477046 |
| 2497 | 0.0277458461538462 | -0.249500359281437 | -0.142942914171657 | 0.0488929740518962 |
| 2498 | -0.00166892307692308 | -0.0601009980039920 | 0.181115169660679 | 0.0172181237524950 |
| 2499 | 0.0102221538461538 | 0.614004790419162 | 0.117765469061876 | 0.0669232734530938 |
| 2500 | 0.0375507692307692 | 0.460666027944112 | 0.0211165668662675 | 0.103633612774451 |
| 2501 | 0.0114738461538462 | -0.0927504590818363 | -0.0426392215568862 | -0.0406087824351297 |
| 2502 | -0.0446436923076923 | -0.0129948103792415 | -0.0190861277445110 | -0.0292383233532934 |
| 2503 | -0.115572923076923 | -0.0475934930139721 | 0.123856786427146 | 0.191023712574850 |
| 2504 | -0.114947076923077 | -0.0389844311377246 | 0.219693512974052 | 0.135470898203593 |
| 2505 | -0.0559089230769231 | 0.256809940119761 | 0.00487305389221557 | -0.109156407185629 |
| 2506 | 0.00959630769230769 | -0.320322075848303 | -0.219287425149701 | 0.0472686227544910 |
| 2507 | 0.0486073846153846 | -0.843038323353293 | 0.167714271457086 | 0.173805588822355 |

| | | | | |
|---|---|---|---|---|
| 2508 | 0.0340043076923077 | 0.442473293413174 | 0.319591117764471 | -0.100222475049900 |
| 2509 | 0.0385938461538462 | 0.908662115768463 | -0.177866467065868 | -0.171856367265469 |
| 2510 | 0.0427661538461539 | -0.511345788423154 | -0.341519860279441 | 0.0805678243512974 |
| 2511 | 0.0590381538461538 | -0.540259241516966 | 0.290758882235529 | 0.0393093013972056 |
| 2512 | 0.0659224615384615 | 0.546431776447106 | 0.211571756487026 | -0.240241556886228 |
| 2513 | 0.0175236923076923 | 0.115166506986028 | -0.373194710578842 | -0.0818673053892216 |
| 2514 | -0.0137686153846154 | -0.545457165668663 | -0.101521956087824 | 0.147653532934132 |
| 2515 | -0.0187753846153846 | -0.0610756087824351 | 0.275327544910180 | -0.115004071856287 |
| 2516 | 0.0373421538461538 | 0.0167308183632735 | -0.0288322355289421 | -0.238617205588822 |
| 2517 | 0.0930424615384616 | -0.261845429141717 | -0.409336526946108 | 0.185013612774451 |
| 2518 | 0.0982578461538461 | 0.401377205588822 | -0.0215226546906188 | 0.236830419161677 |
| 2519 | 0.0878270769230769 | 0.643080678642715 | 0.182333433133733 | -0.103633612774451 |
| 2520 | 0.0563261538461538 | -0.284586347305389 | -0.123450698602794 | 0.0193297804391218 |
| 2521 | 0.0582036923076923 | -0.169419840319361 | -0.186800399201597 | 0.191023712574850 |
| 2522 | 0.0798996923076923 | 0.539934371257485 | -0.0203043912175649 | -0.0510046307385230 |
| 2523 | 0.0917907692307692 | -0.0334616367265469 | 0.218069161676647 | -0.181602475049900 |
| 2524 | 0.0725981538461538 | -0.554553532934132 | 0.103552395209581 | 0.0857657485029940 |
| 2525 | 0.0143944615384615 | 0.0332992015968064 | -0.0755323353293413 | 0.0323245908183633 |
| 2526 | -0.0296233846153846 | 0.313174930139721 | -0.0625375249500998 | -0.209703752495010 |
| 2527 | -0.0241993846153846 | -0.204830698602794 | 0.209541317365269 | 0.0172181237524950 |
| 2528 | 0.0289975384615385 | -0.180627864271457 | 0.289134530938124 | 0.192972934131737 |
| 2529 | 0.0924166153846154 | 0.230170578842315 | -0.0657862275449102 | -0.0935626347305389 |
| 2530 | 0.102847384615385 | 0.00617253493013972 | -0.129135928143713 | -0.197845988023952 |
| 2531 | 0.0711378461538462 | -0.253073932135729 | 0.0946184630738523 | 0.214251936127745 |
| 2532 | 0.0477729230769231 | -0.0807302594810379 | 0.375225149700599 | 0.170719321357285 |
| 2533 | 0.0417230769230769 | 0.0165683832335329 | 0.0929941117764471 | -0.308464311377246 |
| 2534 | 0.0490246153846154 | -0.0220911776447106 | -0.110455888223553 | -0.215064111776447 |
| 2535 | 0.0300406153846154 | 0.0641618762475050 | 0.160810778443114 | 0.246089221556886 |
| 2536 | -0.00458953846153846 | 0.119714690618762 | 0.170150798403194 | 0.147003792415170 |
| 2537 | -0.0304578461538462 | 0.0976235129740519 | 0.0402026946107784 | -0.199470339321357 |
| 2538 | -0.0181495384615385 | 0.0839789620758483 | -0.134008982035928 | -0.0162435129740519 |
| 2539 | 0.0469384615384615 | -0.0222536127744511 | -0.135227245508982 | 0.234556327345309 |
| 2540 | 0.0976320000000000 | -0.106719880239521 | -0.166902095808383 | 0.0895017564870260 |
| 2541 | 0.100552615384615 | 0.00828419161676647 | -0.0974610778443114 | -0.132871936127745 |
| 2542 | 0.0621673846153846 | 0.113217285429142 | 0.00121826347305389 | 0.0406087824351297 |
| 2543 | 0.0200270769230769 | 0.0961615968063872 | -0.115735029940120 | 0.215226546906188 |
| 2544 | -0.000834461538461538 | 0.0882022754491018 | -0.101521956087824 | 0.0328118962075848 |
| 2545 | -0.00312923076923077 | -0.0310251097804391 | 0.469031437125748 | -0.144567265469062 |
| 2546 | -0.00813600000000000 | -0.0911261077844311 | 0.234312674650699 | 0.0190049101796407 |
| 2547 | -0.0446436923076923 | -0.0138069860279441 | -1.43511437125749 | 0.145541876247505 |
| 2548 | -0.0919993846153846 | -0.0388219960079840 | -0.0755323353293413 | -0.153988502994012 |
| 2549 | -0.113695384615385 | -0.119227385229541 | 2.01297734530938 | -0.237155289421158 |
| 2550 | -0.0713464615384615 | -0.0852784431137725 | -0.791465169660679 | 0.0984356886227545 |
| 2551 | 0.00250338461538462 | -0.0575020359281437 | -2.24485349301397 | 0.213764630738523 |

| | | | | |
|---|---|---|---|---|
| 2552 | 0.0404713846153846 | -0.0628623952095808 | 1.79653253493014 | -0.198495728542914 |
| 2553 | 0.0360904615384615 | 0.117278163672655 | 1.96871377245509 | -0.269642315369262 |
| 2554 | 0.00897046153846154 | 0.154963113772455 | -2.58962205588822 | 0.275327544910180 |
| 2555 | 0.00125169230769231 | 0.0307002395209581 | -1.05461007984032 | 0.263144910179641 |
| 2556 | 0.0323353846153846 | -0.00958367265469062 | 2.56282025948104 | -0.271753972055888 |
| 2557 | 0.0671741538461538 | -0.0739079840319361 | 0.269236227544910 | -0.164384351297405 |
| 2558 | 0.0642535384615385 | 0.0160810778443114 | -1.92891716566866 | 0.163734610778443 |
| 2559 | 0.0225304615384615 | 0.110943193612774 | 0.642430938123752 | -0.114841636726547 |
| 2560 | -0.0302492307692308 | -0.0901514970059880 | 1.14191896207585 | -0.120689301397206 |
| 2561 | -0.0400541538461538 | -0.0891768862275449 | -1.24465918163673 | 0.289784271457086 |
| 2562 | -0.0102221538461538 | 0.216363592814371 | 0.241622255489022 | 0.145379441117764 |
| 2563 | 0.0150203076923077 | 0.165358962075848 | 0.985169061876248 | -0.248363313373253 |
| 2564 | 0.0137686153846154 | -0.254535848303393 | -1.32059760479042 | 0.0201419560878244 |
| 2565 | -0.0152289230769231 | -0.289621836327345 | -0.483650598802395 | 0.398453373253493 |
| 2566 | -0.0216960000000000 | 0.113542155688623 | 1.85622744510978 | -0.0146191616766467 |
| 2567 | -0.0108480000000000 | 0.0776439920159681 | -0.0105582834331337 | -0.378636287425150 |
| 2568 | 0.0118910769230769 | -0.128648622754491 | -2.04424610778443 | 0.169094970059880 |
| 2569 | 0.0285803076923077 | -0.00129948103792415 | 0.662329241516966 | 0.442960598802395 |
| 2570 | -0.00751015384615385 | -0.00714714570858283 | 1.96587115768463 | -0.200282514970060 |
| 2571 | -0.0513193846153846 | -0.216850898203593 | -0.839383532934132 | -0.274515369261477 |
| 2572 | -0.0542400000000000 | -0.386758043912176 | -1.70841147704591 | 0.294657325349301 |
| 2573 | 0.00458953846153846 | 0.127024271457086 | 0.945372455089820 | 0.178353772455090 |
| 2574 | 0.0744756923076923 | 0.841738842315369 | 1.23653742514970 | -0.328281397205589 |
| 2575 | 0.0919993846153846 | 0.636095968063872 | -1.02983872255489 | -0.121501477045908 |
| 2576 | 0.0609156923076923 | 0.140506387225549 | -0.578269061876248 | 0.280850339321357 |
| 2577 | 0.0173150769230769 | -0.107532055888224 | 0.890956686626747 | -0.0852784431137725 |
| 2578 | -0.0120996923076923 | -0.428503872255489 | 0.267205788423154 | -0.318697724550898 |
| 2579 | -0.0104307692307692 | -0.303753692614771 | -0.538878542914172 | 0.167795489021956 |
| 2580 | 0.0219046153846154 | 0.430777964071856 | 0.0783749500998004 | 0.227409181636727 |
| 2581 | 0.00959630769230769 | -0.242028343313373 | 0.373600798403194 | -0.310900838323353 |
| 2582 | -0.0611243076923077 | -0.929291377245509 | -0.267611876247505 | -0.173643153692615 |
| 2583 | -0.123500307692308 | 0.904763672654691 | -0.0255835329341317 | 0.310251097804391 |
| 2584 | -0.126212307692308 | 1.23077097804391 | 0.239591816367265 | 0.0248525748502994 |
| 2585 | -0.0705120000000000 | -0.844987544910180 | -0.286291916167665 | -0.295469500998004 |
| 2586 | -0.0177323076923077 | -0.726897205588822 | -0.114922854291417 | 0.0987605588822355 |
| 2587 | -0.000834461538461538 | 0.681415369261477 | 0.358575548902196 | 0.231470059880240 |
| 2588 | -0.00959630769230769 | -0.0420706986027944 | 0.142942914171657 | -0.181927345309381 |
| 2589 | -0.0221132307692308 | -1.02902654690619 | -0.395529540918164 | -0.0555528143712575 |
| 2590 | -0.0135600000000000 | -0.0987605588822355 | -0.114110678642715 | 0.247713572854291 |
| 2591 | 0.0137686153846154 | 0.376037325349301 | 0.380098203592814 | -0.0474310578842315 |
| 2592 | 0.0129341538461538 | -0.179490818363273 | -0.0625375249500998 | -0.273053453093812 |
| 2593 | -0.0133513846153846 | 0.287672614770459 | -0.461721856287425 | 0.106395009980040 |
| 2594 | -0.0285803076923077 | 0.591913612774451 | -0.0645679640718563 | 0.261195688622755 |
| 2595 | -0.0239907692307692 | -0.329418443113772 | 0.510452395209581 | -0.165034091816367 |

| | | | | |
|---|---|---|---|---|
| 2596 | 0.0131427692307692 | -0.122313652694611 | 0.0381722554890220 | -0.144729700598802 |
| 2597 | 0.0492332307692308 | 0.727709381237525 | -0.525883732534930 | 0.246089221556886 |
| 2598 | 0.0431833846153846 | -0.0779688622754491 | 0.143349001996008 | 0.0984356886227545 |
| 2599 | 0.0118910769230769 | -0.749962994011976 | 0.679384930139721 | -0.191186147704591 |
| 2600 | -0.0254510769230769 | 0.132709500998004 | 0.0560401197604790 | 0.00259896207584830 |
| 2601 | -0.0143944615384615 | 0.555040838323353 | -0.529944610778443 | 0.121339041916168 |
| 2602 | 0.0227390769230769 | -0.211003233532934 | 0.123450698602794 | -0.0177054291417166 |
| 2603 | 0.0323353846153846 | -0.241053732534930 | 0.531162874251497 | 0.0289134530938124 |
| 2604 | 0.0133513846153846 | 0.346636566866268 | -0.101928043912176 | 0.104283353293413 |
| 2605 | -0.0490246153846154 | 0.0875525349301397 | -0.300098902195609 | -0.0238779640718563 |
| 2606 | -0.0855323076923077 | -0.128811057884232 | -0.00649740518962076 | -0.0339489421157685 |
| 2607 | -0.0571606153846154 | 0.0466188822355289 | 0.150252495009980 | 0.141643433133733 |
| 2608 | 0.0106393846153846 | -0.138882035928144 | 0.0519792415169661 | -0.00909636726546906 |
| 2609 | 0.0801083076923077 | -0.187937445109780 | -0.163653393213573 | -0.200607385229541 |
| 2610 | 0.105142153846154 | -0.0120201996007984 | -0.199389121756487 | 0.0321621556886228 |
| 2611 | 0.0938769230769231 | -0.0618877844311377 | -0.00487305389221557 | 0.136445508982036 |
| 2612 | 0.0819858461538462 | 0.0336240718562874 | 0.208323053892216 | -0.152039281437126 |
| 2613 | 0.0982578461538461 | 0.210028622754491 | -0.0588827345309381 | -0.142618043912176 |
| 2614 | 0.0997181538461539 | -0.0622126546906188 | -0.337458982035928 | 0.178841077844311 |
| 2615 | 0.0563261538461538 | -0.207754530938124 | -0.101115868263473 | 0.0844662674650699 |
| 2616 | -0.0104307692307692 | 0.229358403193613 | 0.141318562874252 | -0.179003512974052 |
| 2617 | -0.0703033846153846 | 0.320971816367266 | -0.0129948103792415 | -0.0144567265469062 |
| 2618 | -0.0796910769230769 | -0.114354331337325 | -0.216850898203593 | 0.181764910179641 |
| 2619 | -0.0473556923076923 | -0.240241556886228 | 0.00406087824351297 | -0.0349235528942116 |
| 2620 | 0.0129341538461538 | 0.0544157684630739 | 0.302941516966068 | -0.132059760479042 |
| 2621 | 0.0436006153846154 | 0.0339489421157685 | 0.210759580838323 | 0.0937250698602794 |
| 2622 | 0.0267027692307692 | -0.118252774451098 | -0.222130039920160 | 0.127674011976048 |
| 2623 | 0.0141858461538462 | 0.121663912175649 | -0.0231470059880240 | -0.0422331337325349 |
| 2624 | 0.0185667692307692 | 0.0921007185628743 | 0.403245209580838 | -0.0652989221556886 |
| 2625 | 0.0550744615384615 | -0.156100159680639 | -0.0536035928143713 | 0.0607507385229541 |
| 2626 | 0.0644621538461539 | -0.0404463473053892 | -0.359793812375250 | 0.0402839121756487 |
| 2627 | 0.0146030769230769 | 0.124587744510978 | 0.0576644710578842 | -0.0367103393213573 |
| 2628 | -0.0383852307692308 | -0.184851177644711 | 0.243246606786427 | 0.0310251097804391 |
| 2629 | -0.0627932307692308 | -0.330555489021956 | -0.175836027944112 | 0.000487305389221557 |
| 2630 | -0.0448523076923077 | 0.151064670658683 | -0.192079540918164 | -0.0251774451097804 |
| 2631 | -0.00917907692307692 | 0.201581996007984 | 0.146191616766467 | 0.108506666666667 |
| 2632 | 0.0120996923076923 | -0.293682714570858 | 0.154313373253493 | 0.0459691417165669 |
| 2633 | 0.0141858461538462 | -0.207104790419162 | 0.0625375249500998 | -0.137420119760479 |
| 2634 | -0.0104307692307692 | 0.214576806387226 | -0.0950245508982036 | 0.0100709780439122 |
| 2635 | -0.0287889230769231 | 0.0539284630738523 | -0.138069860279441 | 0.234393892215569 |
| 2636 | -0.0173150769230769 | -0.121826347305389 | 0.0665984031936128 | -0.0274515369261477 |
| 2637 | -0.00625846153846154 | 0.134333852295409 | 0.135633333333333 | -0.268667704590818 |
| 2638 | -0.0262855384615385 | 0.273540758483034 | -0.219693512974052 | 0.0362230339321357 |
| 2639 | -0.0717636923076923 | -0.0178678642714571 | -0.242434431137725 | 0.214089500998004 |

| | | | | |
|---|---|---|---|---|
| 2640 | -0.110148923076923 | -0.0508421956087824 | 0.210759580838323 | -0.103633612774451 |
| 2641 | -0.110357538461538 | 0.274028063872256 | 0.244058782435130 | -0.265094131736527 |
| 2642 | -0.0552830769230769 | 0.121014171656687 | -0.124262874251497 | 0.0290758882235529 |
| 2643 | -0.00125169230769231 | -0.0311875449101796 | -0.202231736526946 | 0.143917524950100 |
| 2644 | 0.0296233846153846 | 0.0706592814371258 | 0.214008283433134 | -0.0995727345309381 |
| 2645 | 0.0329612307692308 | 0.0178678642714571 | 0.227409181636727 | -0.132222195608782 |
| 2646 | 0.0210701538461538 | -0.0974610778443114 | -0.156749900199601 | 0.0544157684630739 |
| 2647 | 0.0463126153846154 | -0.116953293413174 | -0.160404690618762 | 0.0466188822355289 |
| 2648 | 0.0713464615384615 | 0.0204668263473054 | 0.131572455089820 | -0.0168932534930140 |
| 2649 | 0.0738498461538462 | 0.159186427145709 | 0.0296444111776447 | 0.0266393612774451 |
| 2650 | 0.0323353846153846 | -0.0328118962075848 | -0.132790718562874 | -0.0428828742514970 |
| 2651 | -0.0300406153846154 | -0.262982475049900 | -0.0881210578842315 | -0.0841413972055888 |
| 2652 | -0.0433920000000000 | -0.118415209580838 | -0.0605070858283433 | 0.0992478642714571 |
| 2653 | -0.0210701538461538 | -0.127511576846307 | 0.000812175648702595 | 0.150577365269461 |
| 2654 | 0.0327526153846154 | -0.148140838323353 | -0.0552279441117765 | -0.0864154890219561 |
| 2655 | 0.0782307692307692 | 0.0880398403193613 | -0.0312687624750499 | -0.0791059081836327 |
| 2656 | 0.0728067692307692 | 0.193785109780439 | -0.184363872255489 | 0.193297804391218 |
| 2657 | 0.0538227692307692 | 0.362230339321357 | 0.433701796407186 | 0.0810551297405190 |
| 2658 | 0.0527796923076923 | 0.414696886227545 | 0.313093712574850 | -0.139694211576846 |
| 2659 | 0.0786480000000000 | 0.115653812375249 | -1.04770658682635 | 0.109318842315369 |
| 2660 | 0.0915821538461538 | -0.173805588822355 | -0.278982335329341 | 0.197358682634731 |
| 2661 | 0.0596640000000000 | -0.126699401197605 | 1.58780339321357 | -0.225459960079840 |
| 2662 | 0.00333784615384615 | 0.0998976047904192 | -0.0990854291417166 | -0.184851177644711 |
| 2663 | -0.0402627692307692 | -0.0272891017964072 | -1.94840938123753 | 0.217175768463074 |
| 2664 | -0.0379680000000000 | -0.0998976047904192 | 1.12161457085828 | 0.00844662674650699 |
| 2665 | 0.00479815384615385 | 0.0310251097804391 | 1.74861417165669 | -0.330393053892216 |
| 2666 | 0.0573692307692308 | 0.0516543712574850 | -1.22435479041916 | 0.00178678642714571 |
| 2667 | 0.0796910769230769 | -0.0295631936127745 | -0.897860179640719 | 0.345499520958084 |
| 2668 | 0.0744756923076923 | -0.0245277045908184 | 1.22232435129741 | -0.0729333732534930 |
| 2669 | 0.0671741538461538 | -0.292545668662675 | 0.428828742514970 | -0.250150099800399 |
| 2670 | 0.0780221538461538 | -0.633172135728543 | -0.970143812375250 | 0.247876007984032 |
| 2671 | 0.111400615384615 | 0.146029181636727 | 0.282231037924152 | 0.243977564870259 |
| 2672 | 0.102638769230769 | 0.678004231536926 | 0.281418862275449 | -0.206130179640719 |
| 2673 | 0.0531969230769231 | -0.0110455888223553 | -0.735831137724551 | -0.0626999600798403 |
| 2674 | 0.00834461538461538 | -0.226921876247505 | 0.376443413173653 | 0.264606826347305 |
| 2675 | -0.0146030769230769 | 0.344524910179641 | 0.694410179640719 | -0.0219287425149701 |
| 2676 | 0.0210701538461538 | 0.353946147704591 | -0.841820059880239 | -0.244789740518962 |
| 2677 | 0.0531969230769231 | -0.0665984031936128 | -0.446696606786427 | 0.107044750499002 |
| 2678 | 0.0584123076923077 | -0.230657884231537 | 1.09034580838323 | 0.258921596806387 |
| 2679 | 0.0569520000000000 | -0.0620502195608782 | 0.272484930139721 | -0.138069860279441 |
| 2680 | 0.0463126153846154 | 0.0851160079840319 | -0.999382135728543 | -0.179653253493014 |
| 2681 | 0.0751015384615385 | 0.240079121756487 | -0.207916966067864 | 0.230820319361277 |
| 2682 | 0.0984664615384615 | 0.415509061876248 | 0.994915169660679 | 0.165034091816367 |
| 2683 | 0.0978406153846154 | -0.0462940119760479 | 0.229439620758483 | -0.173805588822355 |

| | | | | |
|---|---|---|---|---|
| 2684 | 0.0807341538461538 | -0.231957365269461 | -0.849129640718563 | -0.104770658682635 |
| 2685 | 0.0369249230769231 | 0.0558776846307385 | -0.0227409181636727 | 0.0776439920159681 |
| 2686 | 0.0139772307692308 | 0.0146191616766467 | 0.732988522954092 | -0.00682227544910180 |
| 2687 | 0.0133513846153846 | -0.443935209580838 | 0.0747201596806387 | -0.0817048702594810 |
| 2688 | 0.0444350769230769 | -0.483731816367266 | -0.428828742514970 | -0.0562025548902196 |
| 2689 | 0.0636276923076923 | 0.0233906586826347 | 0.0601009980039920 | -0.0355732934131737 |
| 2690 | 0.0454781538461538 | 0.180627864271457 | 0.305784131736527 | -0.0347611177644711 |
| 2691 | 0.0381766153846154 | 0.128811057884232 | -0.184363872255489 | -0.0188424750499002 |
| 2692 | 0.0285803076923077 | 0.0972986427145709 | -0.201013473053892 | 0.0341113772455090 |
| 2693 | 0.0413058461538462 | 0.0402839121756487 | 0.140100299401198 | -0.0311875449101796 |
| 2694 | 0.0479815384615385 | -0.106395009980040 | -0.158374251497006 | -0.0121826347305389 |
| 2695 | 0.0308750769230769 | 0.00519792415169661 | -0.250962275449102 | 0.125237485029940 |
| 2696 | 0.0106393846153846 | 0.0685476247504990 | 0.264363173652695 | 0.0656237924151697 |
| 2697 | -0.0365076923076923 | -0.0981108183632735 | 0.0998976047904192 | -0.0527914171656687 |
| 2698 | -0.0415144615384615 | -0.0384971257485030 | -0.248525748502994 | -0.0167308183632735 |
| 2699 | -0.0158547692307692 | 0.167633053892216 | 0.0300504990019960 | 0.0821921756487026 |
| 2700 | 0.00542400000000000 | 0.128648622754491 | 0.348017265469062 | 0.0373600798403194 |
| 2701 | 0.0168978461538462 | -0.114354331337325 | 0.0296444111776447 | -0.0880398403193613 |
| 2702 | 0.00208615384615385 | -0.0951869860279441 | -0.377255588822355 | 0.0326494610778443 |
| 2703 | 0.00709292307692308 | -0.130272974051896 | -0.0268017964071856 | 0.192648063872255 |
| 2704 | 0.00667569230769231 | -0.137744990019960 | 0.178678642714571 | -0.0339489421157685 |
| 2705 | 0.0223218461538462 | -0.00129948103792415 | -0.148222055888224 | -0.180465429141717 |
| 2706 | 0.0319181538461538 | 0.0360605988023952 | -0.313093712574850 | 0.112242674650699 |
| 2707 | 0.00375507692307692 | 0.0510046307385230 | 0.142130738522954 | 0.137095249500998 |
| 2708 | -0.0146030769230769 | 0.125887225548902 | 0.244464870259481 | -0.199632774451098 |
| 2709 | -0.0312923076923077 | 0.121014171656687 | -0.222130039920160 | -0.150577365269461 |
| 2710 | -0.00479815384615385 | -0.0224160479041916 | -0.122232435129741 | 0.173968023952096 |
| 2711 | 0.0377593846153846 | -0.0384971257485030 | 0.261520558882236 | 0.0654613572854292 |
| 2712 | 0.0590381538461538 | -0.0865779241516966 | 0.181927345309381 | -0.215713852295409 |
| 2713 | 0.0527796923076923 | -0.101521956087824 | -0.335022455089820 | 0.0284261477045908 |
| 2714 | 0.00855323076923077 | 0.120364431137725 | -0.110049800399202 | 0.269642315369262 |
| 2715 | -0.00813600000000000 | 0.138882035928144 | 0.325276347305389 | -0.0565274251497006 |
| 2716 | -0.00271200000000000 | -0.0664359680638723 | -0.0454818363273453 | -0.223998043912176 |
| 2717 | -0.00479815384615385 | -0.0229033532934132 | -0.285479740518962 | 0.137095249500998 |
| 2718 | -0.0325440000000000 | 0.141805868263473 | -0.0808114770459082 | 0.147815968063872 |
| 2719 | -0.0738498461538462 | 0.0342738123752495 | 0.148628143712575 | -0.214576806387226 |
| 2720 | -0.0926252307692308 | -0.175429940119760 | 0.0511670658682635 | -0.0578269061876247 |
| 2721 | -0.0955458461538462 | -0.0696846706586826 | -0.0869027944111777 | 0.230657884231537 |
| 2722 | -0.0625846153846154 | 0.0524665469061876 | -0.121826347305389 | -0.0828419161676647 |
| 2723 | -0.0123083076923077 | 0.348748223552894 | -0.0702531936127745 | -0.311550578842315 |
| 2724 | 0.00312923076923077 | 0.294494890219561 | 0.137257684630739 | 0.0909636726546906 |
| 2725 | -0.0125169230769231 | -0.762145628742515 | 0.0881210578842315 | 0.215064111776447 |
| 2726 | -0.0271200000000000 | -0.770267385229541 | -0.226190918163673 | -0.156100159680639 |
| 2727 | -0.0362990769230769 | 0.302291776447106 | -0.247307485029940 | -0.163897045908184 |

| | | | | |
|---|---|---|---|---|
| 2728 | -0.0448523076923077 | 0.368727744510978 | 0.143755089820359 | 0.224647784431138 |
| 2729 | -0.0655052307692308 | -0.0224160479041916 | 0.210353493013972 | 0.254210978043912 |
| 2730 | -0.0940855384615385 | 0.0294007584830339 | -0.0856845309381238 | -0.0963240319361277 |
| 2731 | -0.112235076923077 | 0 | -0.134008982035928 | -0.0708217165668663 |
| 2732 | -0.105976615384615 | 0.585091337325349 | 0.183551696606786 | 0.150577365269461 |
| 2733 | -0.0634190769230769 | 0.874063433133733 | 0.355732934131737 | 0.0521416766467066 |
| 2734 | -0.0241993846153846 | -0.657699840319361 | -0.0706592814371258 | -0.0246901397205589 |
| 2735 | -0.0319181538461538 | -0.890794251497006 | -0.219287425149701 | 0.0178678642714571 |
| 2736 | -0.0721809230769231 | 0.812338083832335 | 0.226597005988024 | -0.0480807984031936 |
| 2737 | -0.111192000000000 | 0.835241437125749 | 0.173399500998004 | 0.0259896207584830 |
| 2738 | -0.124334769230769 | -0.560238762475050 | -0.290352794411178 | 0.197196247504990 |
| 2739 | -0.0805255384615385 | -0.268830139720559 | -0.155531636726547 | -0.00487305389221557 |
| 2740 | -0.0216960000000000 | 0.754348742514970 | 0.240810079840319 | -0.249500359281437 |
| 2741 | 0.00312923076923077 | 0.224972654690619 | -0.0263957085828343 | -0.0292383233532934 |
| 2742 | -0.0120996923076923 | -0.223185868263473 | -0.313093712574850 | 0.235368502994012 |
| 2743 | -0.0296233846153846 | -0.103146307385230 | 0.108425449101796 | -0.0138069860279441 |
| 2744 | -0.00897046153846154 | -0.522066506986028 | 0.372788622754491 | -0.254698283433134 |
| 2745 | 0.0129341538461538 | -0.428016566866267 | -0.0178678642714571 | 0.0635121357285429 |
| 2746 | 0.0187753846153846 | 0.277276766467066 | -0.137257684630739 | 0.225135089820359 |
| 2747 | -0.00438092307692308 | 0.0217663073852295 | 0.166902095808383 | -0.00909636726546906 |
| 2748 | -0.0348387692307692 | -0.500949940119761 | 0.216444810379242 | -0.0206292614770459 |
| 2749 | -0.0381766153846154 | 0.204830698602794 | -0.00974610778443114 | 0.135958203592814 |
| 2750 | -0.0281630769230769 | 0.564462075848303 | -0.137663772455090 | 0.0233906586826347 |
| 2751 | -0.00396369230769231 | -0.262495169660679 | -0.0690349301397206 | -0.0763445109780439 |
| 2752 | 0 | -0.361093293413174 | 0.0726897205588822 | 0.0352484231536926 |
| 2753 | -0.0104307692307692 | 0.299367944111776 | 0.159186427145709 | 0.00503548902195609 |
| 2754 | -0.0112652307692308 | 0.119877125748503 | -0.0393905189620759 | -0.0779688622754491 |
| 2755 | -0.0114738461538462 | -0.325520000000000 | -0.166089920159681 | -0.0227409181636727 |
| 2756 | 0.0225304615384615 | 0.0800805189620758 | 0.0166496007984032 | 0.0258271856287425 |
| 2757 | 0.0588295384615385 | 0.373113493013972 | 0.205886526946108 | -0.0475934930139721 |
| 2758 | 0.0552830769230769 | 0.150414930139721 | 0.0471061876247505 | -0.0742328542914172 |
| 2759 | 0.0229476923076923 | 0.0388219960079840 | -0.177054291417166 | -0.0284261477045908 |
| 2760 | -0.0146030769230769 | -0.0339489421157685 | -0.0296444111776447 | -0.0529538522954092 |
| 2761 | 0.00187753846153846 | -0.0955118562874252 | 0.128729840319361 | -0.0729333732534930 |
| 2762 | 0.0509021538461538 | -0.108831536926148 | 0.0665984031936128 | 0.00942123752495010 |
| 2763 | 0.0767704615384615 | -0.299692814371258 | 0.0215226546906188 | 0.0177054291417166 |
| 2764 | 0.0709292307692308 | -0.446046866267465 | -0.202637824351297 | -0.0508421956087824 |
| 2765 | 0.0385938461538462 | -0.0337865069860280 | 0.102740219560878 | 0.0898266267465070 |
| 2766 | 0.0252424615384615 | 0.258596726546906 | 0.136445508982036 | 0.192810499001996 |
| 2767 | 0.0352560000000000 | 0.00227409181636727 | 0.0162435129740519 | 0.0214414371257485 |
| 2768 | 0.0496504615384615 | -0.205967744510978 | -0.117359381237525 | -0.0618877844311377 |
| 2769 | 0.0362990769230769 | 0.0480807984031936 | 0.0601009980039920 | 0.0813800000000000 |
| 2770 | -0.00709292307692308 | 0.240566427145709 | 0.668014471057884 | 0.0680603193612775 |
| 2771 | -0.0402627692307692 | -0.0194922155688623 | -0.578269061876248 | 0.00909636726546906 |

| | | | | |
|---|---|---|---|---|
| 2772 | -0.0350473846153846 | 0.0467813173652695 | -0.776439920159681 | 0.0948621157684631 |
| 2773 | 0.0285803076923077 | 0.0696846706586826 | 0.140100299401198 | -0.0490554091816367 |
| 2774 | 0.112026461538462 | -0.141968303393214 | 1.14801027944112 | -0.157074770459082 |
| 2775 | 0.169395692307692 | 0.0235530938123753 | -0.686288423153693 | 0.0755323353293413 |
| 2776 | 0.172524923076923 | 0.199957644710579 | -1.23491307385230 | 0.0946996806387226 |
| 2777 | 0.151037538461538 | -0.0423955688622755 | 1.31125758483034 | -0.260221077844311 |
| 2778 | 0.135182769230769 | -0.133684111776447 | 0.694410179640719 | -0.234069021956088 |
| 2779 | 0.114947076923077 | 0.179165948103792 | -0.605476946107784 | 0.164546786427146 |
| 2780 | 0.0872012307692308 | 0.179328383233533 | -1.02049870259481 | 0.101034650698603 |
| 2781 | 0.0371335384615385 | -0.0903139321357285 | 1.21542085828343 | -0.215551417165669 |
| 2782 | -0.0269113846153846 | -0.0617253493013972 | 0.460097504990020 | -0.145866746506986 |
| 2783 | -0.0655052307692308 | 0.225135089820359 | -0.735018962075848 | 0.0883647105788423 |
| 2784 | -0.0538227692307692 | -0.0419082634730539 | 0.267205788423154 | 0.0782937325349302 |
| 2785 | 0.000834461538461538 | -0.320322075848303 | -0.188830838323353 | 0.0462940119760479 |
| 2786 | 0.0479815384615385 | 0.114841636726547 | 0.114516766467066 | -0.0336240718562874 |
| 2787 | 0.0486073846153846 | 0.113054850299401 | 0.0714714570858283 | -0.168607664670659 |
| 2788 | 0.0298320000000000 | -0.201581996007984 | 0.343550299401198 | 0.0828419161676647 |
| 2789 | 0.0106393846153846 | 0.0971362075848303 | -0.968925548902196 | 0.271429101796407 |
| 2790 | 0.0296233846153846 | 0.325195129740519 | -0.277357984031936 | -0.0747201596806387 |
| 2791 | 0.0590381538461538 | -0.0344362475049900 | 1.13623373253493 | -0.282961996007984 |
| 2792 | 0.0483987692307692 | -0.0380098203592814 | -0.274921457085828 | 0.112405109780439 |
| 2793 | 0.0120996923076923 | 0.110943193612774 | -1.05623443113772 | 0.273378323353293 |
| 2794 | -0.0344215384615385 | -0.235693373253493 | -0.198170858283433 | -0.164546786427146 |
| 2795 | -0.0342129230769231 | -0.177704031936128 | 1.17927904191617 | -0.193297804391218 |
| 2796 | 0.000208615384615385 | 0.332017405189621 | -0.358575548902196 | 0.246414091816367 |
| 2797 | 0.0469384615384615 | 0.0415833932135729 | -0.934814171656687 | 0.185338483033932 |
| 2798 | 0.0757273846153846 | -0.0930753293413174 | 0.258271856287425 | -0.140993692614770 |
| 2799 | 0.0527796923076923 | 0.453356447105788 | 0.624156986027944 | -0.00584766467065868 |
| 2800 | 0.0206529230769231 | -0.377986546906188 | 0.0637557884231537 | 0.177704031936128 |
| 2801 | 0.00208615384615385 | -1.04640710578842 | -0.516137624750499 | 0.000162435129740519 |
| 2802 | 0.00938769230769231 | -0.0227409181636727 | 0.444260079840319 | -0.0612380439121757 |
| 2803 | 0.00917907692307692 | 0.727222075848304 | 0.199389121756487 | 0.125562355289421 |
| 2804 | -0.0264941538461538 | 0.00828419161676647 | -0.181521257485030 | 0.0544157684630739 |
| 2805 | -0.0713464615384615 | -0.349397964071856 | -0.0491366267465070 | -0.108344231536926 |
| 2806 | -0.0934596923076923 | 0.525152774451098 | 0.0621314371257485 | 0.0864154890219561 |
| 2807 | -0.0592467692307692 | 0.440686506986028 | -0.0479183632734531 | 0.197683552894212 |
| 2808 | -0.00166892307692308 | -0.232282235528942 | -0.197764770459082 | -0.0508421956087824 |
| 2809 | 0.0431833846153846 | -0.450107744510978 | 0.239591816367265 | -0.0458067065868264 |
| 2810 | 0.0642535384615385 | -0.216201157684631 | -0.107613273453094 | 0.220586906187625 |
| 2811 | 0.0613329230769231 | 0.411448183632735 | -0.0609131736526946 | 0.0350859880239521 |
| 2812 | 0.0742670769230769 | 0.562837724550898 | 0.230251796407186 | -0.280038163672655 |
| 2813 | 0.0769790769230769 | -0.0183551696606786 | 0.275733632734531 | 0.0112080239520958 |
| 2814 | 0.0567433846153846 | -0.228708662674651 | -0.220911776447106 | 0.285885828343313 |
| 2815 | 0.00584123076923077 | 0.319672335329341 | -0.200201297405190 | -0.102496566866267 |

| | | | | |
|---|---|---|---|---|
| 2816 | -0.0634190769230769 | 0.482919640718563 | 0.312687624750499 | -0.239916686626747 |
| 2817 | -0.0861581538461538 | -0.114679201596806 | -0.00771566866267465 | 0.220911776447106 |
| 2818 | -0.0709292307692308 | -0.583629421157685 | -0.291571057884232 | 0.197845988023952 |
| 2819 | -0.0106393846153846 | -0.264931696606786 | -0.110861976047904 | -0.269479880239521 |
| 2820 | 0.0492332307692308 | 0.310738403193613 | 0.377255588822355 | -0.133196806387226 |
| 2821 | 0.0617501538461538 | 0.397641197604790 | -0.0751262475049900 | 0.312525189620759 |
| 2822 | 0.0356732307692308 | -0.268992574850299 | -0.256647504990020 | 0.127998882235529 |
| 2823 | -0.00730153846153846 | -0.519792415169661 | 0.0410148702594810 | -0.245439481037924 |
| 2824 | -0.0264941538461538 | 0.297906027944112 | 0.292383233532934 | -0.0475934930139721 |
| 2825 | -0.0377593846153846 | 0.424930299401198 | 0.0601009980039920 | 0.213277325349301 |
| 2826 | -0.0569520000000000 | -0.486818083832335 | -0.270048403193613 | -0.0230657884231537 |
| 2827 | -0.0867840000000000 | -0.619690019960080 | 0.131572455089820 | -0.164871656686627 |
| 2828 | -0.118284923076923 | 0.217338203592814 | 0.122232435129741 | 0.0810551297405190 |
| 2829 | -0.115572923076923 | 0.335915848303393 | 0.113298502994012 | 0.0248525748502994 |
| 2830 | -0.0728067692307692 | -0.217987944111776 | -0.147815968063872 | -0.191511017964072 |
| 2831 | -0.00458953846153846 | -0.0680603193612775 | 0.00934001996007984 | 0.0914509780439122 |
| 2832 | 0.0490246153846154 | 0.174617764471058 | 0.159186427145709 | 0.261195688622755 |
| 2833 | 0.0527796923076923 | -0.0722836327345309 | 0.103958483033932 | -0.166171137724551 |
| 2834 | 0.0481901538461539 | 0.0674105788423154 | 0.0629436127744511 | -0.261358123752495 |
| 2835 | 0.0504849230769231 | 0.306027784431138 | -0.199389121756487 | 0.202881477045908 |
| 2836 | 0.0577864615384615 | 0.0354108582834331 | 0.146597704590818 | 0.176891856287425 |
| 2837 | 0.0454781538461538 | -0.153338762475050 | 0.194109980039920 | -0.295307065868264 |
| 2838 | -0.0173150769230769 | 0.0678978842315369 | -0.00527914171656687 | -0.198333293413174 |
| 2839 | -0.0967975384615385 | 0.0997351696606787 | -0.317154590818363 | 0.230820319361277 |
| 2840 | -0.136434461538462 | 0.0126699401197605 | -0.135633333333333 | 0.103796047904192 |
| 2841 | -0.0915821538461538 | 0.141805868263473 | 0.162841217564870 | -0.229683273453094 |
| 2842 | -0.0321267692307692 | 0.0492178443113772 | 0.00284261477045908 | 0.00730958083832335 |
| 2843 | 0.00730153846153846 | -0.206617485029940 | -0.138069860279441 | 0.241216167664671 |
| 2844 | 0.0271200000000000 | -0.106395009980040 | -0.110455888223553 | -0.0984356886227545 |
| 2845 | 0.0120996923076923 | 0.227409181636727 | 0.327306786427146 | -0.225622395209581 |
| 2846 | 0.0229476923076923 | 0.0638370059880239 | 0.158374251497006 | 0.104608223552894 |
| 2847 | 0.0523624615384615 | -0.236343113772455 | -0.168526447105788 | 0.0843038323353293 |
| 2848 | 0.0853236923076923 | -0.0272891017964072 | -0.0966489021956088 | -0.223023433133733 |
| 2849 | 0.0694689230769231 | 0.0289134530938124 | 0.226597005988024 | -0.126861836327345 |
| 2850 | -0.0156461538461538 | -0.157724510978044 | 0.149034231536926 | 0.140506387225549 |
| 2851 | -0.0721809230769231 | -0.121339041916168 | -0.294413672654691 | 0.0484056686626747 |
| 2852 | -0.0794824615384615 | -0.0683851896207585 | -0.0207104790419162 | -0.172668542914172 |
| 2853 | -0.0275372307692308 | -0.0555528143712575 | 0.204262175648703 | -0.0289134530938124 |
| 2854 | 0.0519452307692308 | 0.167145748502994 | -0.0410148702594810 | 0.187937445109780 |
| 2855 | 0.0894960000000000 | 0.184688742514970 | -0.334616367265469 | 0.0584766467065868 |
| 2856 | 0.0844892307692308 | -0.149277884231537 | 0.0304565868263473 | -0.105745269461078 |
| 2857 | 0.0642535384615385 | -0.132547065868263 | 0.147815968063872 | 0.0131572455089820 |
| 2858 | 0.0546572307692308 | 0.156749900199601 | -0.235124850299401 | 0.176079680638723 |
| 2859 | 0.0502763076923077 | 0.0305378043912176 | -0.175023852295409 | 0.0662735329341317 |

| | | | | |
|---|---|---|---|---|
| 2860 | 0.0275372307692308 | -0.202719041916168 | -0.0913697604790419 | -0.119389820359281 |
| 2861 | -0.0239907692307692 | -0.0183551696606786 | 0.0418270459081836 | -0.117603033932136 |
| 2862 | -0.0840720000000000 | 0.140668822355289 | -0.0873088822355290 | 0.0518168063872256 |
| 2863 | -0.116198769230769 | -0.0302129341317365 | -0.0771566866267465 | 0.0828419161676647 |
| 2864 | -0.0763532307692308 | -0.0893393213572854 | -0.0515731536926148 | -0.0909636726546906 |
| 2865 | -0.00146030769230769 | 0.139531776447106 | 0.0207104790419162 | -0.130272974051896 |
| 2866 | 0.0292061538461538 | 0.0734206786427146 | 0.226190918163673 | 0.0383346906187625 |
| 2867 | 0.0173150769230769 | -0.179490818363273 | 0.0596949101796407 | 0.139694211576846 |
| 2868 | -0.0112652307692308 | -0.0685476247504990 | -0.0966489021956088 | 0.0362230339321357 |
| 2869 | -0.0148116923076923 | -0.0562025548902196 | 0.00934001996007984 | -0.0172181237524950 |
| 2870 | 0.0196098461538462 | -0.0664359680638723 | 0.353702495009980 | 0.0222536127744511 |
| 2871 | 0.0471470769230769 | 0.297093852295409 | -0.00649740518962076 | 0.0222536127744511 |
| 2872 | 0.0448523076923077 | 0.252749061876248 | -0.184769960079840 | 0.0277764071856287 |
| 2873 | -0.00500676923076923 | -0.0191673453093812 | 0.0771566866267465 | 0.0414209580838323 |
| 2874 | -0.0723895384615385 | -0.0511670658682635 | 0.150658582834331 | -0.0482432335329341 |
| 2875 | -0.0876184615384615 | 0.0508421956087824 | -0.00324870259481038 | -0.0458067065868264 |
| 2876 | -0.0433920000000000 | 0.257946986027944 | -0.185988223552894 | 0.141480998003992 |
| 2877 | 0.0219046153846154 | 0.0778064271457086 | 0.0990854291417166 | 0.142455608782435 |
| 2878 | 0.0496504615384615 | 0.0430453093812375 | 0.0572583832335329 | -0.0264769261477046 |
| 2879 | 0.0304578461538462 | 0.100872215568862 | -0.0182739520958084 | -0.0570147305389222 |
| 2880 | 0.00625846153846154 | -0.0674105788423154 | 0.000812175648702595 | 0.151064670658683 |
| 2881 | -0.00688430769230769 | 0.0873900998003992 | 0.146191616766467 | 0.172830978043912 |
| 2882 | 0.00500676923076923 | 0.0287510179640719 | -0.101928043912176 | -0.124100439121756 |
| 2883 | 0.00876184615384615 | -0.545944471057884 | 0.0885271457085828 | -0.168607664670659 |
| 2884 | -0.0354646153846154 | -0.446046866267465 | 0.623750898203593 | 0.0552279441117765 |
| 2885 | -0.0851150769230769 | 0.428828742514970 | -0.606695209580838 | 0.0817048702594810 |
| 2886 | -0.105142153846154 | 0.336728023952096 | -1.30069930139721 | -0.129135928143713 |
| 2887 | -0.0615415384615385 | -0.258596726546906 | 1.00100648702595 | -0.186475528942116 |
| 2888 | 0.00834461538461538 | -0.0172181237524950 | 1.51633193612774 | 0.0204668263473054 |
| 2889 | 0.0600812307692308 | 0.233906586826347 | -1.89521187624751 | 0.111105628742515 |
| 2890 | 0.102221538461538 | 0.0690349301397206 | -1.00019431137725 | -0.0854408782435130 |
| 2891 | 0.102012923076923 | -0.0633497005988024 | 2.35449720558882 | -0.0898266267465070 |
| 2892 | 0.0949200000000000 | -0.204993133732535 | 0.357763373253493 | 0.120201996007984 |
| 2893 | 0.0886615384615385 | -0.178841077844311 | -2.30739101796407 | 0.0232282235528942 |
| 2894 | 0.0504849230769231 | 0.204018522954092 | 0.106395009980040 | -0.137907425149701 |
| 2895 | -0.0260769230769231 | 0.445721996007984 | 1.97886596806387 | -0.00454818363273453 |
| 2896 | -0.126420923076923 | 0.208404271457086 | -0.609131736526946 | 0.100709780439122 |
| 2897 | -0.183790153846154 | -0.0399590419161677 | -0.903139321357286 | -0.0222536127744511 |
| 2898 | -0.164806153846154 | 0.0540908982035928 | 0.949433333333333 | -0.0578269061876247 |
| 2899 | -0.0861581538461538 | 0.104608223552894 | 0.189643013972056 | 0.135795768463074 |
| 2900 | -0.0118910769230769 | -0.127998882235529 | -0.441823552894212 | 0.250312534930140 |
| 2901 | 0.0164806153846154 | -0.262982475049900 | 0.812987824351298 | 0.0349235528942116 |
| 2902 | 0.0112652307692308 | -0.0370352095808383 | 0.106395009980040 | -0.0769942514970060 |
| 2903 | 0 | -0.0151064670658683 | -1.19267994011976 | 0.118577644710579 |

| | | | | |
|---|---|---|---|---|
| 2904 | 0.0256596923076923 | -0.126536966067864 | 0.635121357285429 | 0.0750450299401198 |
| 2905 | 0.0550744615384615 | 0.118740079840319 | 1.14273113772455 | -0.0972986427145709 |
| 2906 | 0.0567433846153846 | 0.220749341317365 | -1.00060039920160 | 0.0123450698602794 |
| 2907 | 0.0281630769230769 | -0.0880398403193613 | -1.05339181636727 | 0.0898266267465070 |
| 2908 | -0.0198184615384615 | -0.255835329341317 | 1.07044750499002 | -0.0618877844311377 |
| 2909 | -0.0317095384615385 | 0.0646491816367266 | 0.968925548902196 | -0.0474310578842315 |
| 2910 | -0.00125169230769231 | 0.221399081836327 | -1.28729840319361 | 0.121501477045908 |
| 2911 | 0.0440178461538462 | -0.0573396007984032 | -0.847505289421158 | 0.0388219960079840 |
| 2912 | 0.0604984615384615 | -0.0882022754491018 | 1.10537105788423 | -0.161622954091816 |
| 2913 | 0.0573692307692308 | 0.172018802395210 | 0.542939421157685 | -0.0685476247504990 |
| 2914 | 0.0486073846153846 | 0.131897325349301 | -0.895829740518962 | 0.153176327345309 |
| 2915 | 0.0383852307692308 | -0.110780758483034 | -0.324058083832335 | -0.0138069860279441 |
| 2916 | 0.0504849230769231 | -0.0784561676646707 | 0.797556487025948 | -0.249825229540918 |
| 2917 | 0.0442264615384615 | -0.0173805588822355 | 0.0982732534930140 | 0.0285885828343313 |
| 2918 | 0.0156461538461538 | -0.113054850299401 | -0.542533333333333 | 0.201094690618762 |
| 2919 | -0.00584123076923077 | 0.0453194011976048 | 0.0759384231536926 | -0.136607944111776 |
| 2920 | -0.00938769230769231 | 0.253073932135729 | 0.284261477045908 | -0.182252215568862 |
| 2921 | 0.0210701538461538 | 0.0222536127744511 | -0.252992714570858 | 0.161460518962076 |
| 2922 | 0.0809427692307692 | -0.116953293413174 | -0.183145608782435 | 0.139369341317365 |
| 2923 | 0.127255384615385 | 0.0680603193612775 | 0.185176047904192 | -0.0875525349301397 |
| 2924 | 0.144570461538462 | -0.0285885828343313 | -0.246901397205589 | 0.0300504990019960 |
| 2925 | 0.136225846153846 | -0.269967185628743 | -0.221723952095808 | 0.168120359281437 |
| 2926 | 0.120371076923077 | -0.230170578842315 | 0.235937025948104 | -0.0159186427145709 |
| 2927 | 0.142275692307692 | -0.0407712175648703 | 0.0661923153692615 | -0.164709221556886 |
| 2928 | 0.155209846153846 | -0.0625375249500998 | -0.403651297405190 | -0.00536035928143713 |
| 2929 | 0.133305230769231 | -0.0674105788423154 | -0.134008982035928 | 0.00454818363273453 |
| 2930 | 0.0767704615384615 | 0.186637964071856 | 0.438574850299401 | -0.153663632734531 |
| 2931 | 0.0110566153846154 | 0.171856367265469 | 0.0198983033932136 | -0.0524665469061876 |
| 2932 | 0.0154375384615385 | -0.0357357285429142 | -0.244870958083832 | 0.0518168063872256 |
| 2933 | 0.0703033846153846 | -0.103471177644711 | 0.300504990019960 | -0.0178678642714571 |
| 2934 | 0.126212307692308 | -0.0523041117764471 | 0.467813173652695 | 0.0498675848303393 |
| 2935 | 0.149160000000000 | 0.0155937724550898 | -0.174617764471058 | 0.144242395209581 |
| 2936 | 0.129550153846154 | -0.0380098203592814 | -0.304159780439122 | -0.0742328542914172 |
| 2937 | 0.101178461538462 | -0.134658722554890 | 0.350859880239521 | -0.106232574850299 |
| 2938 | 0.0807341538461538 | 0.0549030738522954 | 0.312687624750499 | 0.125399920159681 |
| 2939 | 0.0784393846153846 | 0.279225988023952 | -0.356951197604790 | 0.0407712175648703 |
| 2940 | 0.0748929230769231 | 0.00503548902195609 | -0.337865069860279 | -0.193947544910180 |
| 2941 | 0.0429747692307692 | -0.122476087824351 | 0.434920059880240 | -0.0578269061876247 |
| 2942 | 0.0206529230769231 | 0.0558776846307385 | 0.196140419161677 | 0.236667984031936 |
| 2943 | 0.0275372307692308 | 0.00763445109780439 | -0.522635029940120 | 0.0800805189620758 |
| 2944 | 0.0728067692307692 | -0.0870652295409182 | -0.188424750499002 | -0.106232574850299 |
| 2945 | 0.134139692307692 | 0.0730958083832335 | 0.289134530938124 | 0.0740704191616766 |
| 2946 | 0.151872000000000 | -0.0282637125748503 | 0.0406087824351297 | 0.131085149700599 |
| 2947 | 0.118702153846154 | -0.243002954091816 | -0.387407784431138 | -0.0854408782435130 |

| | | | | |
|---|---|---|---|---|
| 2948 | 0.0596640000000000 | -0.224647784431138 | -0.101928043912176 | -0.0919382834331337 |
| 2949 | 0.0440178461538462 | -0.341601077844311 | 0.151470758483034 | 0.0423955688622755 |
| 2950 | 0.0452695384615385 | 0.398453373253493 | 0.0605070858283433 | -0.0630248303393214 |
| 2951 | 0.0254510769230769 | 0.999950658682635 | 0.0406087824351297 | -0.0282637125748503 |
| 2952 | 0.00187753846153846 | 0.521579201596806 | 0.00730958083832335 | 0.0992478642714571 |
| 2953 | -0.0565347692307692 | 0.0263144910179641 | -0.0990854291417166 | -0.0407712175648703 |
| 2954 | -0.0919993846153846 | -0.486493213572854 | -0.0259896207584830 | -0.153663632734531 |
| 2955 | -0.0830289230769231 | -0.290758882235529 | 0.262738822355289 | 0.0729333732534930 |
| 2956 | -0.0419316923076923 | 0.200607385229541 | -0.0231470059880240 | 0.143592654690619 |
| 2957 | 0.0123083076923077 | -0.100384910179641 | -0.272078842315369 | -0.101359520958084 |
| 2958 | 0.0223218461538462 | -0.789434730538922 | 0.0726897205588822 | 0.00958367265469062 |
| 2959 | 0.0158547692307692 | -0.225947265469062 | 0.320403293413174 | 0.175592375249501 |
| 2960 | 0.00897046153846154 | 1.23174558882236 | -0.0649740518962076 | -0.0393093013972056 |
| 2961 | 0.00897046153846154 | 0.493640359281437 | -0.418270459081836 | -0.127186706586826 |
| 2962 | 0.0194012307692308 | -1.02204183632735 | 0.244464870259481 | 0.115653812375249 |
| 2963 | 0.00980492307692308 | -0.153826067864271 | 0.418270459081836 | 0.0396341716566866 |
| 2964 | -0.0225304615384615 | 0.710978562874251 | -0.320809381237525 | -0.252424191616766 |
| 2965 | -0.0394283076923077 | -0.353946147704591 | -0.190049101796407 | 0.0185176047904192 |
| 2966 | -0.0166892307692308 | -0.709354211576846 | 0.499081936127745 | 0.355570499001996 |
| 2967 | 0.0281630769230769 | 0.0633497005988024 | 0.220911776447106 | 0.0243652694610778 |
| 2968 | 0.0648793846153846 | 0.169907145708583 | -0.441823552894212 | -0.235206067864271 |
| 2969 | 0.0669655384615385 | 0.116628423153693 | 0.0117765469061876 | 0.210678363273453 |
| 2970 | 0.0429747692307692 | 0.536685668662675 | 0.418676546906188 | 0.362230339321357 |
| 2971 | 0.0200270769230769 | 0.186800399201597 | -0.0162435129740519 | -0.121339041916168 |
| 2972 | 0.00730153846153846 | -0.298230898203593 | -0.294413672654691 | -0.226759441117764 |
| 2973 | 0.0225304615384615 | 0.408849221556886 | -0.0751262475049900 | 0.157886946107784 |
| 2974 | 0.0398455384615385 | 0.436950499001996 | 0.181521257485030 | 0.153176327345309 |
| 2975 | 0.00354646153846154 | -0.547731257485030 | 0.00121826347305389 | -0.201906866267465 |
| 2976 | -0.0492332307692308 | -0.363042514970060 | -0.0410148702594810 | -0.145217005988024 |
| 2977 | -0.0748929230769231 | 0.501924550898204 | -0.139288123752495 | 0.103796047904192 |
| 2978 | -0.0398455384615385 | 0.136932814371257 | -0.0791871257485030 | 0.0713090219560878 |
| 2979 | 0.0379680000000000 | -0.480158243512974 | 0.285479740518962 | -0.0703344111776447 |
| 2980 | 0.0811513846153846 | 0.0506797604790419 | 0.178272554890220 | -0.0985981237524950 |
| 2981 | 0.0892873846153846 | 0.403326427145709 | -0.200607385229541 | -0.0693598003992016 |
| 2982 | 0.0506935384615385 | -0.162272694610778 | -0.197764770459082 | 0.00584766467065868 |
| 2983 | 0.00625846153846154 | -0.186313093812375 | 0.374412974051896 | 0.0527914171656687 |
| 2984 | -0.000208615384615385 | 0.127024271457086 | 0.261520558882236 | -0.0513295009980040 |
| 2985 | -0.0129341538461538 | -0.0607507385229541 | -0.206698702594810 | -0.113542155688623 |
| 2986 | -0.0448523076923077 | -0.0846287025948104 | -0.0649740518962076 | 0.0339489421157685 |
| 2987 | -0.109940307692308 | 0.0297256287425150 | 0.228221357285429 | 0.177541596806387 |
| 2988 | -0.164806153846154 | 0.0341113772455090 | 0.0958367265469062 | 0.0115328942115768 |
| 2989 | -0.162928615384615 | 0.184363872255489 | -0.208323053892216 | -0.125237485029940 |
| 2990 | -0.120371076923077 | 0.203856087824351 | -0.110861976047904 | 0.0789434730538922 |
| 2991 | -0.0663396923076923 | -0.209054011976048 | -0.0507609780439122 | 0.215226546906188 |

| | | | | |
|---|---|---|---|---|
| 2992 | -0.0582036923076923 | -0.171531497005988 | -0.0954306387225549 | 0.0560401197604790 |
| 2993 | -0.0867840000000000 | 0.303103952095808 | -0.119795908183633 | -0.0719587624750499 |
| 2994 | -0.0988836923076923 | 0.202556606786427 | 0.0495427145708583 | 0.0160810778443114 |
| 2995 | -0.0819858461538462 | -0.235530938123752 | 0.00121826347305389 | 0.0807302594810379 |
| 2996 | -0.0486073846153846 | -0.269967185628743 | 0.195734331337325 | -0.0123450698602794 |
| 2997 | -0.0438092307692308 | 0.0138069860279441 | 0.436950499001996 | -0.102659001996008 |
| 2998 | -0.0890787692307692 | -0.0857657485029940 | -0.902733233532934 | -0.0285885828343313 |
| 2999 | -0.153123692307692 | -0.169744710578842 | -0.851160079840319 | 0.0164059481037924 |
| 3000 | -0.189840000000000 | 0.0529538522954092 | 1.65724441117764 | -0.0946996806387226 |
| 3001 | -0.167726769230769 | -0.0386595608782435 | 0.694004091816367 | -0.0864154890219561 |
| 3002 | -0.117241846153846 | -0.177866467065868 | -2.27977704590818 | 0.0844662674650699 |
| 3003 | -0.0840720000000000 | 0.0644867465069860 | 0.159998602794411 | 0.0472686227544910 |
| 3004 | -0.0784393846153846 | 0.0768318163672655 | 2.76464590818363 | -0.199307904191617 |
| 3005 | -0.0815686153846154 | -0.222211257485030 | -0.784561676646707 | -0.120689301397206 |
| 3006 | -0.0621673846153846 | -0.0190049101796407 | -2.28465009980040 | 0.210515928143713 |
| 3007 | -0.0212787692307692 | 0.263632215568862 | 1.22800958083832 | 0.129298363273453 |
| 3008 | 0.0123083076923077 | 0.0246901397205589 | 1.43795698602794 | -0.195084590818363 |
| 3009 | 0.00229476923076923 | -0.158861556886228 | -1.23044610778443 | -0.0781312974051896 |
| 3010 | -0.0471470769230769 | 0.159673732534930 | -0.525477644710579 | 0.262820039920160 |
| 3011 | -0.0938769230769231 | 0.156912335329341 | 0.830855688622755 | 0.159186427145709 |
| 3012 | -0.100761230769231 | -0.199470339321357 | -0.570147305389222 | -0.161947824351297 |
| 3013 | -0.0619587692307692 | 0.00438574850299401 | -0.184769960079840 | -0.0255023153692615 |
| 3014 | -0.00604984615384615 | 0.249013053892216 | 1.13136067864271 | 0.209054011976048 |
| 3015 | 0.0227390769230769 | -0.0856033133732535 | -0.847505289421158 | -0.0360605988023952 |
| 3016 | 0.0189840000000000 | -0.426392215568862 | -1.27064880239521 | -0.209703752495010 |
| 3017 | 0.0312923076923077 | 0.0237155289421158 | 1.30679061876248 | 0.0371976447105788 |
| 3018 | 0.0559089230769231 | 0.240079121756487 | 1.07572664670659 | 0.112567544910180 |
| 3019 | 0.0859495384615385 | -0.0810551297405190 | -1.52607804391218 | -0.0737455489021956 |
| 3020 | 0.0876184615384615 | -0.0103958483033932 | -0.839383532934132 | -0.0271266666666667 |
| 3021 | 0.0402627692307692 | 0.0110455888223553 | 1.58577295409182 | 0.114029461077844 |
| 3022 | -0.0171064615384615 | -0.154800678642715 | 0.616035229540918 | -0.0435326147704591 |
| 3023 | -0.0458953846153846 | -0.229358403193613 | -1.40871866267465 | -0.185500918163673 |
| 3024 | -0.00500676923076923 | -0.0769942514970060 | -0.479995808383234 | -0.0138069860279441 |
| 3025 | 0.0511107692307692 | 0.288972095808383 | 1.11511716566866 | 0.0890144510978044 |
| 3026 | 0.0826116923076923 | 0.454655928143713 | 0.109643712574850 | -0.0646491816367266 |
| 3027 | 0.0792738461538462 | 0.0971362075848303 | -0.740704191616767 | -0.120526866267465 |
| 3028 | 0.0604984615384615 | 0.00893393213572854 | 0.205074351297405 | 0.0813800000000000 |
| 3029 | 0.0790652307692308 | -0.197683552894212 | 0.492990618762475 | 0.163247305389222 |
| 3030 | 0.0993009230769231 | -0.548868303393214 | -0.188424750499002 | -0.0334616367265469 |
| 3031 | 0.0984664615384615 | 0.210678363273453 | -0.203856087824351 | -0.0934001996007984 |
| 3032 | 0.0579950769230769 | 0.285073652694611 | 0.415021756487026 | 0.0558776846307385 |
| 3033 | -0.0187753846153846 | -0.516706147704591 | 0.0633497005988024 | 0.0974610778443114 |
| 3034 | -0.0644621538461539 | 0.200444950099800 | -0.305378043912176 | 0.0415833932135729 |
| 3035 | -0.0619587692307692 | 1.01294546906188 | 0.117765469061876 | -0.00795932135728543 |

| | | | | |
|---|---|---|---|---|
| 3036 | -0.00917907692307692 | -0.0492178443113772 | 0.149440319361277 | -0.00341113772455090 |
| 3037 | 0.0531969230769231 | -0.686125988023952 | -0.127511576846307 | 0.0646491816367266 |
| 3038 | 0.0719723076923077 | 0.130760279441118 | 0.101115868263473 | -0.00389844311377246 |
| 3039 | 0.0765618461538462 | 0.227246746506986 | 0.231876147704591 | -0.127186706586826 |
| 3040 | 0.0780221538461538 | -0.432239880239521 | -0.214008283433134 | -0.0240403992015968 |
| 3041 | 0.0934596923076923 | -0.206455049900200 | -0.214008283433134 | 0.103796047904192 |
| 3042 | 0.113278153846154 | 0.318860159680639 | 0.211165668662675 | -0.0326494610778443 |
| 3043 | 0.0986750769230769 | 0.0562025548902196 | 0.0982732534930140 | -0.165034091816367 |
| 3044 | 0.0627932307692308 | 0.110618323353293 | -0.377255588822355 | 0.00958367265469062 |
| 3045 | 0.0137686153846154 | 0.472036487025948 | -0.195734331337325 | 0.162272694610778 |
| 3046 | -0.00584123076923077 | -0.204343393213573 | 0.339083333333333 | -0.0402839121756487 |
| 3047 | 0.0221132307692308 | -0.382209860279441 | 0.248525748502994 | -0.173643153692615 |
| 3048 | 0.0703033846153846 | 0.588989780439122 | -0.387813872255489 | 0.0935626347305389 |
| 3049 | 0.0988836923076923 | 0.368890179640719 | -0.354108582834331 | 0.235530938123752 |
| 3050 | 0.0844892307692308 | -0.453356447105788 | 0.406900000000000 | -0.0828419161676647 |
| 3051 | 0.0755187692307692 | -0.0298880638722555 | 0.415833932135729 | -0.215388982035928 |
| 3052 | 0.0869926153846154 | 0.555365708582834 | -0.308626746506986 | 0.166333572854291 |
| 3053 | 0.0995095384615385 | 0.0713090219560878 | -0.398372155688623 | 0.298718203592814 |
| 3054 | 0.0892873846153846 | -0.336240718562874 | 0.388219960079840 | -0.0113704590818363 |
| 3055 | 0.0427661538461539 | 0.0242028343313373 | 0.391062574850299 | -0.126374530938124 |
| 3056 | -0.0264941538461538 | 0.0690349301397206 | -0.227409181636727 | 0.0873900998003992 |
| 3057 | -0.0657138461538462 | -0.118577644710579 | -0.287916267465070 | 0.152689021956088 |
| 3058 | -0.0385938461538462 | 0.0565274251497006 | 0.0617253493013972 | -0.104770658682635 |
| 3059 | 0.0235735384615385 | 0.0222536127744511 | 0.226597005988024 | -0.157724510978044 |
| 3060 | 0.0890787692307692 | -0.264931696606786 | -0.0475122754491018 | 0.104608223552894 |
| 3061 | 0.111192000000000 | -0.0943748103792415 | -0.198983033932136 | 0.0782937325349302 |
| 3062 | 0.110148923076923 | 0.116628423153693 | -0.120608083832335 | -0.128323752495010 |
| 3063 | 0.129967384615385 | -0.0963240319361277 | 0.233094411177645 | 0.00519792415169661 |
| 3064 | 0.148116923076923 | -0.228383792415170 | 0.193297804391218 | 0.100872215568862 |
| 3065 | 0.153540923076923 | -0.0618877844311377 | -0.220911776447106 | -0.0285885828343313 |
| 3066 | 0.120371076923077 | 0.0469437524950100 | -0.216850898203593 | -0.0142942914171657 |
| 3067 | 0.0550744615384615 | -0.0263144910179641 | 0.143755089820359 | 0.0938875049900200 |
| 3068 | 0.00396369230769231 | 0.274677804391218 | 0.205886526946108 | 0.0998976047904192 |
| 3069 | -0.00959630769230769 | 0.221236646706587 | -0.138069860279441 | -0.0839789620758483 |
| 3070 | 0.0156461538461538 | -0.212627584830339 | -0.0584766467065868 | -0.160485908183633 |
| 3071 | 0.0448523076923077 | 0.0152689021956088 | 0.158780339321357 | 0.116303552894212 |
| 3072 | 0.0650880000000000 | 0.156425029940120 | 0.247713572854291 | 0.158374251497006 |
| 3073 | 0.0600812307692308 | -0.0773191217564870 | -0.0531975049900200 | -0.0997351696606787 |
| 3074 | 0.0354646153846154 | -0.0823546107784431 | -0.288728443113772 | -0.143592654690619 |
| 3075 | 0.0342129230769231 | 0.202556606786427 | 0.108425449101796 | -0.0167308183632735 |
| 3076 | 0.0440178461538462 | 0.105257964071856 | 0.225378742514970 | 0.0857657485029940 |
| 3077 | 0.0408886153846154 | -0.125399920159681 | -0.183145608782435 | -0.0108831536926148 |
| 3078 | 0.00625846153846154 | 0.00909636726546906 | -0.245683133732535 | -0.141643433133733 |
| 3079 | -0.0360904615384615 | 0.0217663073852295 | 0.250150099800399 | -0.0414209580838323 |

| | | | | |
|---|---|---|---|---|
| 3080 | -0.0431833846153846 | -0.254698283433134 | 0.232282235528942 | 0.0976235129740519 |
| 3081 | -0.0158547692307692 | -0.141480998003992 | -0.184769960079840 | 0.00649740518962076 |
| 3082 | 0.0310836923076923 | 0.156425029940120 | -0.169744710578842 | -0.0921007185628743 |
| 3083 | 0.0438092307692308 | -0.154150938123753 | 0.180709081836327 | 0.0427204391217565 |
| 3084 | 0.0125169230769231 | -0.204018522954092 | 0.206698702594810 | 0.103796047904192 |
| 3085 | -0.0281630769230769 | 0.137582554890220 | -0.131572455089820 | -0.00601009980039920 |
| 3086 | -0.0563261538461538 | 0.0981108183632735 | -0.223348303393214 | 0.00227409181636727 |
| 3087 | -0.0596640000000000 | -0.127836447105788 | 0.0211165668662675 | 0.0549030738522954 |
| 3088 | -0.0692603076923077 | -0.0435326147704591 | 0.168120359281437 | -0.0869027944111777 |
| 3089 | -0.101595692307692 | 0.00243652694610778 | -0.0406087824351297 | -0.103471177644711 |
| 3090 | -0.147699692307692 | 0.0246901397205589 | -0.159592514970060 | 0.0526289820359282 |
| 3091 | -0.175028307692308 | 0.0825170459081836 | 0.0121826347305389 | 0.0102334131736527 |
| 3092 | -0.149994461538462 | -0.0389844311377246 | 0.232282235528942 | -0.186637964071856 |
| 3093 | -0.0907476923076923 | 0.0227409181636727 | 0.0913697604790419 | -0.137420119760479 |
| 3094 | -0.0400541538461538 | 0.187937445109780 | -0.117765469061876 | 0.132222195608782 |
| 3095 | -0.0337956923076923 | 0.213764630738523 | -0.00446696606786427 | -0.00779688622754491 |
| 3096 | -0.0544486153846154 | -0.0477559281437126 | 0.157562075848303 | -0.243490259481038 |
| 3097 | -0.0492332307692308 | -0.133846546906188 | 0.0763445109780439 | 0.0410960878243513 |
| 3098 | -0.0267027692307692 | 0.198333293413174 | -0.135633333333333 | 0.220099600798403 |
| 3099 | -0.00333784615384615 | 0.395529540918164 | -0.113704590818363 | -0.0997351696606787 |
| 3100 | 0.00584123076923077 | -0.254535848303393 | 0.0235530938123753 | -0.112080239520958 |
| 3101 | -0.0198184615384615 | -0.844175369261477 | -0.0162435129740519 | 0.216688463073852 |
| 3102 | -0.0634190769230769 | -0.261358123752495 | -0.100709780439122 | 0.0990854291417166 |
| 3103 | -0.114112615384615 | 0.314474411177645 | -0.123856786427146 | -0.0745577245508982 |
| 3104 | -0.111817846153846 | 0.172668542914172 | -0.0109643712574850 | 0.127674011976048 |
| 3105 | -0.0765618461538462 | 0.0412585229540918 | 0.0113704590818363 | 0.183876566866267 |
| 3106 | -0.0680086153846154 | -0.0769942514970060 | -0.125887225548902 | -0.0781312974051896 |
| 3107 | -0.0540313846153846 | 0.161135648702595 | 0.00487305389221557 | -0.105420399201597 |
| 3108 | -0.0325440000000000 | 0.983057405189621 | -0.146597704590818 | 0.158049381237525 |
| 3109 | 0.0214873846153846 | 0.252261756487026 | 0.218475249500998 | 0.0937250698602794 |
| 3110 | 0.0838633846153846 | -0.907037764471058 | 0.0247713572854291 | -0.103958483033932 |
| 3111 | 0.103473230769231 | -0.0737455489021956 | -0.460909680638723 | 0.0149440319361277 |
| 3112 | 0.0811513846153846 | 0.842226147704591 | -0.000406087824351297 | 0.0690349301397206 |
| 3113 | 0.0210701538461538 | 0.0290758882235529 | 0.585578642714571 | -0.104120918163673 |
| 3114 | -0.0181495384615385 | -0.667933253493014 | 0.0763445109780439 | -0.0389844311377246 |
| 3115 | 0.000625846153846154 | 0.254048542914172 | -0.927910678642715 | 0.120364431137725 |
| 3116 | 0.0421403076923077 | 0.632522395209581 | 0.627811776447106 | -0.0581517764471058 |
| 3117 | 0.0778135384615385 | -0.0177054291417166 | 0.754917265469062 | -0.123775568862275 |
| 3118 | 0.0805255384615385 | -0.223510738522954 | -0.501112375249501 | 0.0985981237524950 |
| 3119 | 0.0402627692307692 | -0.173318283433134 | -0.448320958083832 | 0.154963113772455 |
| 3120 | 0.00208615384615385 | -0.524340598802395 | 0.448320958083832 | -0.0232282235528942 |
| 3121 | -0.00458953846153846 | -0.441011377245509 | 0.499081936127745 | -0.0937250698602794 |
| 3122 | 0.00292061538461538 | 0.124750179640719 | -0.642837025948104 | 0.0839789620758483 |
| 3123 | 0.0106393846153846 | -0.0755323353293413 | 0.0856845309381238 | 0.228871097804391 |

| | | | | |
|---|---|---|---|---|
| 3124 | -0.00166892307692308 | -0.173968023952096 | 0.0113704590818363 | 0.0201419560878244 |
| 3125 | -0.0114738461538462 | 0.381560119760479 | -0.257053592814371 | -0.238617205588822 |
| 3126 | 0.0239907692307692 | 0.315286586826347 | 0.211165668662675 | -0.0188424750499002 |
| 3127 | 0.0592467692307692 | -0.162597564870259 | 0.104770658682635 | 0.253073932135729 |
| 3128 | 0.0859495384615385 | 0.0549030738522954 | -0.301317165668663 | 0.0466188822355289 |
| 3129 | 0.0771876923076923 | 0.247226267465070 | -0.269642315369262 | -0.288972095808383 |
| 3130 | 0.0260769230769231 | -0.0994102994011976 | 0.737861576846307 | -0.0778064271457086 |
| 3131 | -0.0139772307692308 | -0.217663073852295 | 0.0909636726546906 | 0.312200319361277 |
| 3132 | -0.0335870769230769 | 0.181440039920160 | -0.628217864271457 | 0.0847911377245509 |
| 3133 | -0.00688430769230769 | 0.345012215568862 | -0.0377661676646707 | -0.210191057884232 |
| 3134 | 0.00813600000000000 | 0.103958483033932 | 0.654207485029940 | 0.0711465868263473 |
| 3135 | -0.00584123076923077 | 0.0298880638722555 | 0.209541317365269 | 0.339976726546906 |
| 3136 | -0.0152289230769231 | 0.0183551696606786 | -0.737861576846307 | 0.00665984031936128 |
| 3137 | -0.0162720000000000 | 0.0579893413173653 | -0.00812175648702595 | -0.285398522954092 |
| 3138 | 0.0171064615384615 | -0.159511297405190 | 0.490554091816367 | 0.00698471057884232 |
| 3139 | 0.0481901538461539 | -0.506310299401198 | 0.0592888223552894 | 0.187775009980040 |
| 3140 | 0.0456867692307692 | -0.318860159680639 | -0.404463473053892 | -0.0857657485029940 |
| 3141 | 0.00208615384615385 | 0.118090339321357 | -0.0288322355289421 | -0.174780199600798 |
| 3142 | -0.0486073846153846 | 0.0992478642714571 | 0.369946007984032 | 0.0302129341317365 |
| 3143 | -0.0665483076923077 | -0.0555528143712575 | -0.0649740518962076 | 0.0536035928143713 |
| 3144 | -0.0500676923076923 | 0.100547345309381 | -0.00446696606786427 | -0.0846287025948104 |
| 3145 | 0.00187753846153846 | 0.0717963273453094 | 0.0856845309381238 | -0.0529538522954092 |
| 3146 | 0.0289975384615385 | 0.0527914171656687 | 0.00649740518962076 | 0.0680603193612775 |
| 3147 | 0.0281630769230769 | 0.183226826347305 | -0.00609131736526946 | 0.00925880239520958 |
| 3148 | 0.0139772307692308 | 0.139694211576846 | 0.173805588822355 | -0.0185176047904192 |
| 3149 | 0.00396369230769231 | -0.0862530538922156 | 0.00487305389221557 | 0.00357357285429142 |
| 3150 | 0.0388024615384615 | -0.0717963273453094 | -0.222942215568862 | -0.0841413972055888 |
| 3151 | 0.0448523076923077 | 0.167633053892216 | 0.120201996007984 | -0.0532787225548902 |
| 3152 | 0.0133513846153846 | 0.0324870259481038 | 0.224566566866267 | 0.0307002395209581 |
| 3153 | -0.0241993846153846 | -0.0766693812375250 | -0.0994915169660679 | -0.0391468662674651 |
| 3154 | -0.0521538461538462 | -0.0490554091816367 | -0.208729141716567 | -0.0448320958083832 |
| 3155 | -0.0417230769230769 | -0.0841413972055888 | 0.129135928143713 | 0.115328942115768 |
| 3156 | -0.0194012307692308 | -0.0750450299401198 | 0.0219287425149701 | -0.0216038722554890 |
| 3157 | 0.0133513846153846 | -0.0609131736526946 | -0.170962974051896 | -0.191835888223553 |
| 3158 | 0.0277458461538462 | -0.0178678642714571 | -0.181521257485030 | 0.0297256287425150 |
| 3159 | 0.0150203076923077 | -0.106882315369261 | 0.0763445109780439 | 0.0963240319361277 |
| 3160 | 0.0196098461538462 | -0.117115728542914 | 0.0369539920159681 | -0.112892415169661 |
| 3161 | 0.0398455384615385 | 0.0459691417165669 | -0.222536127744511 | -0.124912614770459 |
| 3162 | 0.0588295384615385 | 0.0389844311377246 | -0.0487305389221557 | 0.130760279441118 |
| 3163 | 0.0469384615384615 | -0.128648622754491 | 0.149440319361277 | 0.0815424351297405 |
| 3164 | -0.00751015384615385 | -0.126699401197605 | 0.147409880239521 | -0.154150938123753 |
| 3165 | -0.0550744615384615 | 0.114354331337325 | -0.237561377245509 | 0.0243652694610778 |
| 3166 | -0.0734326153846154 | 0.264606826347305 | 0.0848723552894212 | 0.301317165668663 |
| 3167 | -0.0463126153846154 | 0.0873900998003992 | 0.267205788423154 | 0.0951869860279441 |

| | | | | |
|---|---|---|---|---|
| 3168 | 0.00312923076923077 | -0.165358962075848 | -0.0369539920159681 | -0.143917524950100 |
| 3169 | 0.0179409230769231 | 0.0173805588822355 | -0.144161177644711 | 0.120851736526946 |
| 3170 | 0.0106393846153846 | 0.223348303393214 | -0.0243652694610778 | 0.170556886227545 |
| 3171 | -0.00354646153846154 | 0.0206292614770459 | 0.124262874251497 | -0.123450698602794 |
| 3172 | 0.00917907692307692 | 0.0336240718562874 | -0.116547205588822 | -0.0998976047904192 |
| 3173 | 0.0390110769230769 | 0.0373600798403194 | -0.0978671656686627 | 0.0532787225548902 |
| 3174 | 0.0469384615384615 | 0.322596167664671 | -0.120201996007984 | -0.0656237924151697 |
| 3175 | 0.0179409230769231 | 0.292383233532934 | -0.0503548902195609 | -0.126374530938124 |
| 3176 | -0.0383852307692308 | -0.901352534930140 | 0.0747201596806387 | 0.00503548902195609 |
| 3177 | -0.0721809230769231 | -0.907200199600799 | 0.0860906187624751 | 0.000649740518962076 |
| 3178 | -0.0680086153846154 | 0.205967744510978 | -0.0812175648702595 | -0.0691973652694611 |
| 3179 | -0.0194012307692308 | 0.592076047904192 | -0.0913697604790419 | 0.0477559281437126 |
| 3180 | 0.0350473846153846 | 0.166820878243513 | 0.239185728542914 | 0.123288263473054 |
| 3181 | 0.0586209230769231 | -0.121501477045908 | 0.122638522954092 | -0.0865779241516966 |
| 3182 | 0.0736412307692308 | 0.180465429141717 | -0.0434513972055888 | -0.0303753692614770 |
| 3183 | 0.0853236923076923 | 0.536523233532934 | -0.0694410179640719 | 0.213439760479042 |
| 3184 | 0.105350769230769 | 0.335753413173653 | 0.170150798403194 | -0.0563649900199601 |
| 3185 | 0.117033230769231 | -0.763445109780439 | 0.248119660678643 | -0.277601636726547 |
| 3186 | 0.0926252307692308 | -0.665984031936128 | -0.0893393213572854 | 0.151714411177645 |
| 3187 | 0.0408886153846154 | 0.882510059880240 | -0.0791871257485030 | 0.336890459081836 |
| 3188 | -0.00751015384615385 | 0.824520718562874 | 0.203043912175649 | -0.157724510978044 |
| 3189 | -0.0164806153846154 | -0.458391936127745 | 0.0942123752495010 | -0.238454770459082 |
| 3190 | 0.0181495384615385 | -0.141968303393214 | -0.315124151696607 | 0.275327544910180 |
| 3191 | 0.0665483076923077 | 0.738267664670659 | 0.00243652694610778 | 0.300180119760479 |
| 3192 | 0.0884529230769231 | 0.202069301397206 | 0.175023852295409 | -0.213114890219561 |
| 3193 | 0.0872012307692308 | -0.450270179640719 | -0.257865768463074 | -0.247388702594810 |
| 3194 | 0.0955458461538462 | -0.368727744510978 | -0.152282934131737 | 0.187287704590818 |
| 3195 | 0.110983384615385 | -0.370352095808383 | 0.134415069860279 | 0.209703752495010 |
| 3196 | 0.120371076923077 | -0.222211257485030 | 0.123044610778443 | -0.102009261477046 |
| 3197 | 0.112235076923077 | 0.322596167664671 | -0.124262874251497 | -0.136445508982036 |
| 3198 | 0.0613329230769231 | -0.0851160079840319 | -0.0239591816367265 | 0.102334131736527 |
| 3199 | -0.00292061538461538 | -0.550005349301397 | 0.0909636726546906 | 0.174455329341317 |
| 3200 | -0.0377593846153846 | 0.308464311377246 | -0.0568522954091816 | 0.00795932135728543 |
| 3201 | -0.0129341538461538 | 0.453518882235529 | 0.0117765469061876 | -0.103146307385230 |
| 3202 | 0.0579950769230769 | -0.476259800399202 | -0.00609131736526946 | 0.0937250698602794 |
| 3203 | 0.111192000000000 | -0.386758043912176 | -0.0495427145708583 | 0.234556327345309 |
| 3204 | 0.139772307692308 | 0.489904351297405 | -0.0410148702594810 | -0.0334616367265469 |
| 3205 | 0.137686153846154 | 0.255835329341317 | 0.145379441117764 | -0.0895017564870260 |
| 3206 | 0.127464000000000 | -0.288484790419162 | 0.0694410179640719 | 0.0906388023952096 |
| 3207 | 0.126838153846154 | 0.181764910179641 | -0.113298502994012 | 0.0167308183632735 |
| 3208 | 0.0990923076923077 | 0.324870259481038 | -0.00162435129740519 | -0.140343952095808 |
| 3209 | 0.0467298461538462 | 0.00698471057884232 | 0.135633333333333 | -0.0641618762475050 |
| 3210 | -0.0148116923076923 | 0.0730958083832335 | 0.135633333333333 | 0.0492178443113772 |
| 3211 | -0.0456867692307692 | 0.179328383233533 | -0.152689021956088 | -0.107694491017964 |

| | | | | |
|---|---|---|---|---|
| 3212 | -0.0287889230769231 | -0.0665984031936128 | 0.0511670658682635 | -0.173643153692615 |
| 3213 | 0.0214873846153846 | -0.190211536926148 | 0.183551696606786 | 0.0404463473053892 |
| 3214 | 0.0788566153846154 | -0.0797556487025948 | 0.0105582834331337 | 0.101846826347305 |
| 3215 | 0.0999267692307692 | -0.235530938123752 | -0.0913697604790419 | -0.0578269061876247 |
| 3216 | 0.109731692307692 | -0.0268017964071856 | 0.00243652694610778 | -0.0188424750499002 |
| 3217 | 0.116198769230769 | 0.305865349301397 | 0.181521257485030 | 0.125399920159681 |
| 3218 | 0.120996923076923 | -0.0344362475049900 | -0.179896906187625 | 0.0488929740518962 |
| 3219 | 0.121414153846154 | -0.317073373253493 | 0.0633497005988024 | -0.0510046307385230 |
| 3220 | 0.0786480000000000 | 0.0492178443113772 | -0.0507609780439122 | 0.0279388423153693 |
| 3221 | 0.00938769230769231 | 0.249175489021956 | 0.256241417165669 | -0.00276139720558882 |
| 3222 | -0.0667569230769231 | -0.0732582435129741 | 0.497457584830339 | -0.0799180838323353 |
| 3223 | -0.102638769230769 | -0.0979483832335329 | -0.765069461077844 | 0.127836447105788 |
| 3224 | -0.0840720000000000 | -0.0180302994011976 | -1.31085149700599 | 0.0678978842315369 |
| 3225 | -0.0410972307692308 | -0.192648063872255 | 0.574614271457086 | -0.236018243512974 |
| 3226 | -0.00521538461538462 | -0.0594512574850299 | 1.79084730538922 | -0.118252774451098 |
| 3227 | 0.00187753846153846 | 0.105745269461078 | -1.76566986027944 | 0.155287984031936 |
| 3228 | 0.00730153846153846 | -0.0779688622754491 | -1.46151007984032 | 0.0659486626746507 |
| 3229 | 0.0146030769230769 | -0.0138069860279441 | 2.21439690618762 | -0.227246746506986 |
| 3230 | 0.0225304615384615 | 0.219124990019960 | 1.09481277445110 | -0.0743952894211577 |
| 3231 | 0.0114738461538462 | 0.107369620758483 | -2.01460169660679 | 0.268830139720559 |
| 3232 | -0.0279544615384615 | -0.171044191616766 | -0.792683433133733 | 0.0511670658682635 |
| 3233 | -0.0880356923076923 | -0.127511576846307 | 2.10191057884232 | -0.260870818363273 |
| 3234 | -0.127046769230769 | 0.0817048702594810 | -0.0442635728542914 | 0.0118577644710579 |
| 3235 | -0.0955458461538462 | -0.0513295009980040 | -0.815424351297405 | 0.228871097804391 |
| 3236 | -0.0337956923076923 | -0.116141117764471 | 0.379692115768463 | -0.0516543712574850 |
| 3237 | 0.0164806153846154 | 0.0930753293413174 | -0.0690349301397206 | -0.116953293413174 |
| 3238 | 0.0273286153846154 | 0.142618043912176 | -0.266393612774451 | 0.135146027944112 |
| 3239 | 0.0223218461538462 | -0.00292383233532934 | 0.803241716566866 | 0.164709221556886 |
| 3240 | 0.0304578461538462 | -0.00276139720558882 | 0.213602195608782 | -0.0337865069860280 |
| 3241 | 0.0310836923076923 | 0.197845988023952 | -1.71409670658683 | -0.0484056686626747 |
| 3242 | 0.0346301538461538 | 0.0620502195608782 | 0.320809381237525 | 0.0300504990019960 |
| 3243 | -0.00667569230769231 | -0.205318003992016 | 1.59592514970060 | -0.0237155289421158 |
| 3244 | -0.0886615384615385 | -0.0740704191616766 | -0.520198502994012 | -0.0272891017964072 |
| 3245 | -0.147073846153846 | 0.0453194011976048 | -1.51714411177645 | -0.0329743313373254 |
| 3246 | -0.166892307692308 | 0.0115328942115768 | 0.418270459081836 | -0.128323752495010 |
| 3247 | -0.121622769230769 | 0.191673453093812 | 1.44932744510978 | -0.0779688622754491 |
| 3248 | -0.0613329230769231 | 0.245439481037924 | -0.634715269461078 | 0.123288263473054 |
| 3249 | -0.0252424615384615 | 0.0643243113772455 | -1.10090409181637 | 0.0430453093812375 |
| 3250 | -0.0146030769230769 | -0.0781312974051896 | 0.438980938123753 | -0.202069301397206 |
| 3251 | -0.0198184615384615 | -0.167145748502994 | 0.834916566866267 | -0.103633612774451 |
| 3252 | 0.00584123076923077 | -0.0646491816367266 | -0.273703193612774 | 0.199145469061876 |
| 3253 | 0.0383852307692308 | -0.0133196806387226 | -0.339083333333333 | 0.106232574850299 |
| 3254 | 0.0444350769230769 | 0.166983313373254 | 0.503142814371258 | -0.128323752495010 |
| 3255 | 0.000625846153846154 | 0.187937445109780 | 0.0174617764471058 | 0.0540908982035928 |

| | | | | |
|---|---|---|---|---|
| 3256 | -0.0667569230769231 | -0.0961615968063872 | -0.214008283433134 | 0.133846546906188 |
| 3257 | -0.0917907692307692 | 0.0159186427145709 | 0.128323752495010 | -0.0180302994011976 |
| 3258 | -0.0703033846153846 | 0.0243652694610778 | 0.151064670658683 | 0.0196546506986028 |
| 3259 | -0.0191926153846154 | -0.381722554890220 | -0.296444111776447 | 0.0804053892215569 |
| 3260 | 0.0277458461538462 | -0.516381277445110 | -0.134415069860279 | -0.0357357285429142 |
| 3261 | 0.0410972307692308 | 0.0116953293413174 | 0.296444111776447 | -0.0388219960079840 |
| 3262 | 0.0229476923076923 | 0.425580039920160 | -0.150658582834331 | 0.172343672654691 |
| 3263 | -0.00146030769230769 | 0.139206906187625 | -0.130354191616766 | 0.0968113373253493 |
| 3264 | -0.00667569230769231 | -0.132547065868263 | 0.217663073852295 | -0.223998043912176 |
| 3265 | -0.0118910769230769 | 0.109806147704591 | 0.107613273453094 | -0.0358981636726547 |
| 3266 | -0.0417230769230769 | 0.251449580838323 | -0.297662375249501 | 0.345012215568862 |
| 3267 | -0.0815686153846154 | 0.00324870259481038 | -0.0856845309381238 | -0.00292383233532934 |
| 3268 | -0.0995095384615385 | -0.102983872255489 | 0.255835329341317 | -0.343550299401198 |
| 3269 | -0.0657138461538462 | -0.137095249500998 | 0.0243652694610778 | 0.0886895808383234 |
| 3270 | -0.00584123076923077 | 0.0107207185628743 | -0.244464870259481 | 0.305865349301397 |
| 3271 | 0.0552830769230769 | 0.462940119760479 | 0.0994915169660679 | -0.181764910179641 |
| 3272 | 0.0924166153846154 | 0.464239600798403 | 0.463752295409182 | -0.279713293413174 |
| 3273 | 0.0840720000000000 | -0.139369341317365 | 0.0129948103792415 | 0.285885828343313 |
| 3274 | 0.0778135384615385 | -0.358169461077844 | -0.312281536926148 | 0.287022874251497 |
| 3275 | 0.0901218461538462 | 0.127349141716567 | 0.0402026946107784 | -0.288809660678643 |
| 3276 | 0.102638769230769 | 0.131247584830339 | 0.559182934131737 | -0.195734331337325 |
| 3277 | 0.0861581538461538 | -0.408361916167665 | -0.00771566866267465 | 0.359144071856287 |
| 3278 | 0.0264941538461538 | -0.314149540918164 | -0.342332035928144 | 0.149277884231537 |
| 3279 | -0.0410972307692308 | 0.226759441117764 | 0.191673453093812 | -0.380585508982036 |
| 3280 | -0.0657138461538462 | 0.152526586826347 | 0.358981636726547 | -0.114354331337325 |
| 3281 | -0.0369249230769231 | -0.132709500998004 | -0.0385783433133733 | 0.317560678642715 |
| 3282 | 0.0171064615384615 | 0.0839789620758483 | -0.293195409181637 | -0.0199795209580838 |
| 3283 | 0.0644621538461539 | -0.0607507385229541 | 0.132384630738523 | -0.298718203592814 |
| 3284 | 0.0705120000000000 | -0.286535568862276 | 0.0856845309381238 | 0.127511576846307 |
| 3285 | 0.0586209230769231 | 0.134658722554890 | -0.0832480039920160 | 0.216201157684631 |
| 3286 | 0.0573692307692308 | 0.297256287425150 | -0.187612574850299 | -0.193785109780439 |
| 3287 | 0.0636276923076923 | -0.0394717365269461 | -0.104770658682635 | -0.164709221556886 |
| 3288 | 0.0705120000000000 | -0.0149440319361277 | 0.0406087824351297 | 0.181927345309381 |
| 3289 | 0.0392196923076923 | 0.304403433133733 | -0.0296444111776447 | 0.0792683433133733 |
| 3290 | -0.00834461538461538 | 0.165358962075848 | -0.0864967065868263 | -0.235043632734531 |
| 3291 | -0.0371335384615385 | -0.126212095808383 | -0.382534730538922 | -0.0540908982035928 |
| 3292 | -0.0356732307692308 | -0.0782937325349302 | -0.124262874251497 | 0.210028622754491 |
| 3293 | 0.00834461538461538 | -0.0138069860279441 | 0.173805588822355 | -0.0388219960079840 |
| 3294 | 0.0479815384615385 | -0.209054011976048 | 0.0365479041916168 | -0.172018802395210 |
| 3295 | 0.0469384615384615 | -0.111592934131737 | -0.112486327345309 | 0.117278163672655 |
| 3296 | 0.0189840000000000 | 0.289296966067864 | 0.0454818363273453 | 0.133846546906188 |
| 3297 | -0.00104307692307692 | 0.0776439920159681 | 0.388626047904192 | -0.0638370059880239 |
| 3298 | 0.00897046153846154 | -0.208241836327345 | 0.209541317365269 | -0.0895017564870260 |
| 3299 | 0.0262855384615385 | 0.0849535728542914 | -0.192485628742515 | -0.000649740518962076 |

| | | | | |
|---|---|---|---|---|
| 3300 | 0.0133513846153846 | 0.0500300199600798 | -0.0877149700598802 | 0.0141318562874252 |
| 3301 | -0.0398455384615385 | -0.330880359281437 | 0.339083333333333 | -0.0938875049900200 |
| 3302 | -0.0907476923076923 | -0.159511297405190 | 0.0917758483033932 | -0.0427204391217565 |
| 3303 | -0.0855323076923077 | 0.198820598802395 | -0.292789321357285 | 0.0734206786427146 |
| 3304 | -0.0337956923076923 | 0.0526289820359282 | 0.0929941117764471 | 0.0201419560878244 |
| 3305 | 0.0406800000000000 | 0.0282637125748503 | 0.305784131736527 | 0 |
| 3306 | 0.0997181538461539 | 0.325032694610778 | -0.110455888223553 | 0.135795768463074 |
| 3307 | 0.104516307692308 | 0.137095249500998 | -0.332179840319361 | 0.140993692614770 |
| 3308 | 0.0930424615384616 | -0.146354051896208 | 0.0605070858283433 | -0.0383346906187625 |
| 3309 | 0.0882443076923077 | -0.0386595608782435 | 0.126293313373253 | 0.0107207185628743 |
| 3310 | 0.0963803076923077 | -0.0349235528942116 | -0.280606686626747 | 0.157562075848303 |
| 3311 | 0.0909563076923077 | -0.182089780439122 | -0.253804890219561 | -0.00308626746506986 |
| 3312 | 0.0438092307692308 | -0.0719587624750499 | 0.00974610778443114 | -0.187287704590818 |
| 3313 | -0.0118910769230769 | 0.0922631536926148 | 0.112892415169661 | -0.0251774451097804 |
| 3314 | -0.0438092307692308 | -0.00162435129740519 | -0.0812175648702595 | 0.159511297405190 |
| 3315 | -0.0216960000000000 | -0.0165683832335329 | -0.144973353293413 | 0.0177054291417166 |
| 3316 | 0.0310836923076923 | 0.0402839121756487 | -0.00730958083832335 | -0.139694211576846 |
| 3317 | 0.0736412307692308 | -0.0282637125748503 | 0.185582135728543 | -0.000812175648702595 |
| 3318 | 0.0721809230769231 | -0.106232574850299 | 0.178678642714571 | 0.149277884231537 |
| 3319 | 0.0429747692307692 | -0.0906388023952096 | -0.112080239520958 | -0.0201419560878244 |
| 3320 | 0.0573692307692308 | -0.0990854291417166 | -0.117765469061876 | -0.0987605588822355 |
| 3321 | 0.0886615384615385 | 0.0459691417165669 | 0.0852784431137725 | 0.0964864670658683 |
| 3322 | 0.111817846153846 | 0.178516207584830 | 0.200201297405190 | 0.0305378043912176 |
| 3323 | 0.106811076923077 | -0.0307002395209581 | -0.161216866267465 | -0.146354051896208 |
| 3324 | 0.0529883076923077 | -0.234718762475050 | -0.277764071856287 | -0.0774815568862275 |
| 3325 | 0.00292061538461538 | -0.278088942115769 | 0.0138069860279441 | 0.0378473852295409 |
| 3326 | -0.0100135384615385 | 0.399915289421158 | 0.0966489021956088 | 0.0531162874251497 |
| 3327 | 0.0325440000000000 | 0.869027944111777 | -0.0706592814371258 | -0.0675730139720559 |
| 3328 | 0.0678000000000000 | 0.297418722554890 | -0.191267365269461 | -0.0126699401197605 |
| 3329 | 0.0692603076923077 | -0.167470618762475 | 0.0190861277445110 | 0.266880918163673 |
| 3330 | 0.0498590769230769 | -0.268505269461078 | 0.0592888223552894 | 0.192485628742515 |
| 3331 | 0.0116824615384615 | -0.0708217165668663 | 0.0893393213572854 | -0.0878774051896208 |
| 3332 | 0.0183581538461538 | 0.178516207584830 | 0.0442635728542914 | 0.0240403992015968 |
| 3333 | 0.0267027692307692 | -0.206130179640719 | 0.0938062874251497 | 0.198333293413174 |
| 3334 | 0.0248252307692308 | -0.953169341317365 | -0.0446696606786427 | -0.0480807984031936 |
| 3335 | -0.00959630769230769 | -0.169257405189621 | 0.0994915169660679 | -0.293520279441118 |
| 3336 | -0.0811513846153846 | 1.14614227544910 | 0.823546107784431 | -0.0701719760479042 |
| 3337 | -0.0924166153846154 | 0.141805868263473 | -0.403651297405190 | 0.230008143712575 |
| 3338 | -0.0619587692307692 | -0.898428702594810 | -1.32953153692615 | -0.0477559281437126 |
| 3339 | -0.0104307692307692 | 0.178191337325349 | 1.05664051896208 | -0.324707824351297 |
| 3340 | 0.0285803076923077 | 0.624563073852296 | 1.71003582834331 | 0.113867025948104 |
| 3341 | 0.0143944615384615 | -0.311388143712575 | -1.80059341317365 | 0.340301596806387 |
| 3342 | -0.0166892307692308 | -0.435326147704591 | -1.24344091816367 | -0.196384071856287 |
| 3343 | -0.0525710769230769 | 0.0168932534930140 | 2.71266666666667 | -0.351184750499002 |

| | | | | |
|---|---|---|---|---|
| 3344 | -0.0519452307692308 | -0.0279388423153693 | 0.224972654690619 | 0.203856087824351 |
| 3345 | -0.0325440000000000 | 0.155287984031936 | -2.69885968063872 | 0.183876566866267 |
| 3346 | -0.0477729230769231 | 0.603608942115769 | 0.525071556886228 | -0.369539920159681 |
| 3347 | -0.0675913846153846 | 0.131897325349301 | 2.14536197604790 | -0.201094690618762 |
| 3348 | -0.0813600000000000 | -0.239429381237525 | -1.04892485029940 | 0.324058083832335 |
| 3349 | -0.0579950769230769 | 0.357844590818363 | -1.37988642714571 | 0.0781312974051896 |
| 3350 | -0.0114738461538462 | 0.257297245508982 | 1.48831187624751 | -0.275002674650699 |
| 3351 | 0.0108480000000000 | -0.471549181636727 | 0.155125548902196 | 0.152039281437126 |
| 3352 | -0.00667569230769231 | -0.225297524950100 | -1.09521886227545 | 0.341113772455090 |
| 3353 | -0.0675913846153846 | 0.310251097804391 | 0.835322654690619 | -0.216201157684631 |
| 3354 | -0.100552615384615 | -0.0321621556886228 | 0.278982335329341 | -0.239754251497006 |
| 3355 | -0.0792738461538462 | -0.277926506986028 | -1.25196876247505 | 0.344687345309381 |
| 3356 | -0.0367163076923077 | 0.100222475049900 | 0.507609780439122 | 0.109318842315369 |
| 3357 | -0.00813600000000000 | 0.177054291417166 | 1.40506387225549 | -0.402676686626747 |
| 3358 | -0.0216960000000000 | -0.140506387225549 | -1.19389820359281 | -0.0402839121756487 |
| 3359 | -0.0531969230769231 | -0.141643433133733 | -1.13014241516966 | 0.304728303393214 |
| 3360 | -0.0634190769230769 | -0.0480807984031936 | 1.66699051896208 | -0.0935626347305389 |
| 3361 | -0.0352560000000000 | -0.0700095409181637 | 0.978265568862275 | -0.311063273453094 |
| 3362 | 0.00542400000000000 | 0.0474310578842315 | -1.71856367265469 | 0.135795768463074 |
| 3363 | 0.0100135384615385 | 0.0648116167664671 | -0.642837025948104 | 0.292058363273453 |
| 3364 | -0.0200270769230769 | 0.0282637125748503 | 1.69907145708583 | -0.0877149700598802 |
| 3365 | -0.0584123076923077 | 0.193785109780439 | 0.340301596806387 | -0.132709500998004 |
| 3366 | -0.0836547692307692 | 0.187937445109780 | -1.30557235528942 | 0.191023712574850 |
| 3367 | -0.0897046153846154 | -0.162110259481038 | -0.0215226546906188 | 0.0872276646706587 |
| 3368 | -0.0817772307692308 | -0.168932534930140 | 0.965676846307385 | -0.121339041916168 |
| 3369 | -0.0784393846153846 | 0.171693932135729 | -0.0909636726546906 | 0.0886895808383234 |
| 3370 | -0.0915821538461538 | 0.166008702594810 | -0.605070858283433 | 0.0971362075848303 |
| 3371 | -0.0740584615384615 | -0.0264769261477046 | 0.218475249500998 | -0.122638522954092 |
| 3372 | -0.0175236923076923 | -0.0268017964071856 | 0.247307485029940 | 0.0815424351297405 |
| 3373 | 0.0436006153846154 | -0.0817048702594810 | -0.357357285429142 | 0.276302155688623 |
| 3374 | 0.0728067692307692 | -0.136445508982036 | 0.101115868263473 | -0.00324870259481038 |
| 3375 | 0.0504849230769231 | 0.0469437524950100 | 0.129948103792415 | -0.0859281836327345 |
| 3376 | 0.0189840000000000 | -0.0204668263473054 | -0.446290518962076 | 0.117603033932136 |
| 3377 | 0.00417230769230769 | -0.300667425149701 | -0.0832480039920160 | 0.0352484231536926 |
| 3378 | 0.0106393846153846 | -0.0906388023952096 | 0.453194011976048 | -0.225947265469062 |
| 3379 | 0.0306664615384615 | 0.100872215568862 | 0.0219287425149701 | -0.0882022754491018 |
| 3380 | 0.0321267692307692 | -0.0233906586826347 | -0.546594211576846 | 0.123125828343313 |
| 3381 | 0.00312923076923077 | 0.0477559281437126 | 0.0869027944111777 | -0.135470898203593 |
| 3382 | -0.0294147692307692 | 0.137420119760479 | 0.442635728542914 | -0.155450419161677 |
| 3383 | -0.0133513846153846 | 0.119552255489022 | -0.0799993013972056 | 0.224810219560878 |
| 3384 | 0.0327526153846154 | -0.0175429940119760 | -0.296038023952096 | 0.0930753293413174 |
| 3385 | 0.0682172307692308 | 0.00503548902195609 | 0.246901397205589 | -0.310738403193613 |
| 3386 | 0.0855323076923077 | 0.0748825948103793 | 0.348423353293413 | -0.0323245908183633 |
| 3387 | 0.0559089230769231 | -0.0539284630738523 | -0.397153892215569 | 0.337215329341317 |

| | | | | |
|---|---|---|---|---|
| 3388 | 0.0277458461538462 | -0.0380098203592814 | -0.237967465069860 | 0.00763445109780439 |
| 3389 | 0.0350473846153846 | 0.108181796407186 | 0.323651996007984 | -0.276951896207585 |
| 3390 | 0.0538227692307692 | 0.161947824351297 | 0.140506387225549 | 0.0652989221556886 |
| 3391 | 0.0492332307692308 | -0.0453194011976048 | -0.405681736526946 | 0.274190499001996 |
| 3392 | -0.0137686153846154 | -0.167633053892216 | -0.195328243512974 | -0.0484056686626747 |
| 3393 | -0.0659224615384615 | -0.0563649900199601 | 0.440605289421158 | -0.0682227544910180 |
| 3394 | -0.0711378461538462 | 0.0578269061876247 | 0.111268063872255 | 0.167308183632735 |
| 3395 | -0.0452695384615385 | -0.0371976447105788 | -0.401620858283433 | 0.0207916966067864 |
| 3396 | -0.00146030769230769 | -0.120689301397206 | -0.102740219560878 | -0.111592934131737 |
| 3397 | 0.0183581538461538 | -0.146516487025948 | 0.268424051896208 | 0.0838165269461078 |
| 3398 | -0.000834461538461538 | -0.189399361277445 | 0.0775627744510978 | 0.0604258682634731 |
| 3399 | -0.0431833846153846 | -0.146516487025948 | -0.122232435129741 | -0.171531497005988 |
| 3400 | -0.0515280000000000 | -0.257297245508982 | -0.0998976047904192 | -0.0349235528942116 |
| 3401 | -0.0179409230769231 | 0.116303552894212 | -0.00893393213572854 | 0.132059760479042 |
| 3402 | -0.000834461538461538 | 0.721861716566866 | 0.188018662674651 | -0.0620502195608782 |
| 3403 | -0.0158547692307692 | 0.577132015968064 | 0.139694211576846 | -0.184201437125749 |
| 3404 | -0.0500676923076923 | 0.0410960878243513 | -0.200201297405190 | 0.0820297405189621 |
| 3405 | -0.0778135384615385 | -0.0482432335329341 | -0.210759580838323 | 0.165683832335329 |
| 3406 | -0.0611243076923077 | -0.317723113772455 | 0.168120359281437 | -0.121826347305389 |
| 3407 | -0.0250338461538462 | -0.444747385229541 | 0.135633333333333 | -0.0563649900199601 |
| 3408 | 0.00417230769230769 | 0.487955129740519 | -0.181927345309381 | 0.218637684630739 |
| 3409 | -0.000834461538461538 | -0.0984356886227545 | -0.120608083832335 | 0.0308626746506986 |
| 3410 | -0.0390110769230769 | -0.808439640718563 | 0.0885271457085828 | -0.216688463073852 |
| 3411 | -0.0475643076923077 | 0.801454930139721 | 0.138475948103792 | -0.00665984031936128 |
| 3412 | -0.0304578461538462 | 0.901352534930140 | -0.143755089820359 | 0.121988782435130 |
| 3413 | 0.0162720000000000 | -0.792358562874252 | -0.218475249500998 | -0.107369620758483 |
| 3414 | 0.0458953846153846 | -0.516381277445110 | 0.208729141716567 | -0.133359241516966 |
| 3415 | 0.0233649230769231 | 0.670207345309381 | 0.227815269461078 | 0.0805678243512974 |
| 3416 | -0.0118910769230769 | -0.138069860279441 | -0.179084730538922 | 0.0635121357285429 |
| 3417 | -0.0279544615384615 | -0.851809820359281 | -0.0316748502994012 | -0.0423955688622755 |
| 3418 | 0.0102221538461538 | 0.215713852295409 | 0.427204391217565 | 0.0191673453093812 |
| 3419 | 0.0360904615384615 | 0.373600798403194 | 0.0852784431137725 | 0.0562025548902196 |
| 3420 | 0.0444350769230769 | -0.371164271457086 | -0.325276347305389 | -0.0620502195608782 |
| 3421 | 0.0554916923076923 | 0.249175489021956 | 0.00406087824351297 | -0.0620502195608782 |
| 3422 | 0.0513193846153846 | 0.566573732534930 | 0.237967465069860 | 0.0971362075848303 |
| 3423 | 0.0492332307692308 | -0.438412415169661 | -0.0446696606786427 | 0.0261520558882236 |
| 3424 | 0.0630018461538462 | -0.142780479041916 | -0.243246606786427 | -0.129785668662675 |
| 3425 | 0.0882443076923077 | 0.788622554890220 | -0.0393905189620759 | 0.0324870259481038 |
| 3426 | 0.0592467692307692 | -0.0220911776447106 | 0.0357357285429142 | 0.237317724550898 |
| 3427 | -0.0152289230769231 | -0.670207345309381 | 0.142536826347305 | 0.00649740518962076 |
| 3428 | -0.0425575384615385 | 0.276139720558882 | 0.0828419161676647 | -0.179328383233533 |
| 3429 | 0.00709292307692308 | 0.505335688622755 | -0.254210978043912 | 0.0575020359281437 |
| 3430 | 0.0742670769230769 | -0.302616646706587 | -0.0247713572854291 | 0.208404271457086 |
| 3431 | 0.114321230769231 | -0.223510738522954 | 0.391874750499002 | 0.0271266666666667 |

| | | | | |
|---|---|---|---|---|
| 3432 | 0.114529846153846 | 0.247713572854291 | 0.112892415169661 | -0.196059201596806 |
| 3433 | 0.0755187692307692 | -0.0919382834331337 | -0.371570359281437 | -0.0575020359281437 |
| 3434 | 0.0406800000000000 | -0.160323473053892 | -0.0296444111776447 | 0.186475528942116 |
| 3435 | 0.0394283076923077 | 0.144892135728543 | 0.358981636726547 | 0.0373600798403194 |
| 3436 | 0.0448523076923077 | -0.162597564870259 | 0.0393905189620759 | -0.221561516966068 |
| 3437 | 0.00834461538461538 | -0.250962275449102 | -0.229439620758483 | -0.0987605588822355 |
| 3438 | -0.0598726153846154 | 0.0557152495009980 | -0.0467000998003992 | 0.155612854291417 |
| 3439 | -0.101595692307692 | 0.0579893413173653 | 0.187612574850299 | 0.0649740518962076 |
| 3440 | -0.0913735384615385 | 0.0154313373253493 | 0.0450757485029940 | -0.129298363273453 |
| 3441 | -0.0408886153846154 | 0.0985981237524950 | -0.0897454091816367 | -0.0477559281437126 |
| 3442 | 0.0227390769230769 | -0.0646491816367266 | -0.0540096806387226 | 0.135308463073852 |
| 3443 | 0.0529883076923077 | -0.0661110978043912 | 0.0121826347305389 | 0.104770658682635 |
| 3444 | 0.0450609230769231 | 0.285073652694611 | 0.0113704590818363 | -0.0765069461077844 |
| 3445 | 0.0275372307692308 | 0.251287145708583 | -0.0735018962075848 | -0.0880398403193613 |
| 3446 | 0.0294147692307692 | -0.173643153692615 | 0.00406087824351297 | 0.0394717365269461 |
| 3447 | 0.0502763076923077 | -0.183389261477046 | -0.108019361277445 | 0.0268017964071856 |
| 3448 | 0.0448523076923077 | 0.263957085828343 | 0.377255588822355 | -0.0589639520958084 |
| 3449 | 0.0206529230769231 | 0.0669232734530938 | 0.178272554890220 | -0.00601009980039920 |
| 3450 | -0.0131427692307692 | -0.209054011976048 | -1.17806077844311 | 0.0691973652694611 |
| 3451 | -0.0406800000000000 | 0.256809940119761 | 0.117765469061876 | -0.0485681037924152 |
| 3452 | -0.0281630769230769 | 0.160485908183633 | 1.69054361277445 | -0.0700095409181637 |
| 3453 | 0.00354646153846154 | -0.276464590818363 | -0.647710079840319 | 0.101684391217565 |
| 3454 | 0.0333784615384615 | -0.00958367265469062 | -1.92648063872255 | 0.0378473852295409 |
| 3455 | 0.0319181538461538 | 0.184363872255489 | 1.54516417165669 | -0.118902514970060 |
| 3456 | 0.0198184615384615 | -0.269317445109780 | 1.55287984031936 | -0.0159186427145709 |
| 3457 | 0.0448523076923077 | -0.367753133732535 | -1.98942425149701 | 0.130922714570858 |
| 3458 | 0.0884529230769231 | 0.251287145708583 | -0.651364870259481 | 0.0922631536926148 |
| 3459 | 0.127255384615385 | 0.174942634730539 | 1.58293033932136 | -0.0230657884231537 |
| 3460 | 0.118702153846154 | -0.363042514970060 | 0.172993413173653 | -0.0230657884231537 |
| 3461 | 0.0577864615384615 | -0.144404830339321 | -1.10049800399202 | 0.0516543712574850 |
| 3462 | 0.000834461538461538 | 0.215064111776447 | 0.521416766467066 | 0.0318372854291417 |
| 3463 | -0.00542400000000000 | -0.0807302594810379 | 0.323245908183633 | 0.00373600798403194 |
| 3464 | 0.0431833846153846 | -0.232282235528942 | -0.955930738522954 | -0.0157562075848303 |
| 3465 | 0.0945027692307692 | 0.160648343313373 | 0.749232035928144 | -0.0328118962075848 |
| 3466 | 0.117659076923077 | 0.158699121756487 | 0.529944610778443 | 0.0345986826347305 |
| 3467 | 0.121622769230769 | -0.0383346906187625 | -1.19146167664671 | 0.0427204391217565 |
| 3468 | 0.109314461538462 | 0.134171417165669 | -0.278576247504990 | -0.0243652694610778 |
| 3469 | 0.109940307692308 | 0.201094690618762 | 1.24790788423154 | 0.000974610778443114 |
| 3470 | 0.124126153846154 | 0.0357357285429142 | 0.0166496007984032 | 0.0622126546906188 |
| 3471 | 0.103264615384615 | 0.0737455489021956 | -1.24953223552894 | 0.0134821157684631 |
| 3472 | 0.0342129230769231 | 0.117440598802395 | 0.0726897205588822 | -0.0721211976047904 |
| 3473 | -0.0521538461538462 | -0.157886946107784 | 1.02943263473054 | -0.0243652694610778 |
| 3474 | -0.0840720000000000 | -0.127024271457086 | -0.0410148702594810 | 0.0422331337325349 |
| 3475 | -0.0496504615384615 | 0.101034650698603 | -1.04648832335329 | -0.0539284630738523 |

| | | | | |
|---|---|---|---|---|
| 3476 | 0.0129341538461538 | 0.0313499800399202 | 0.154719461077844 | -0.0690349301397206 |
| 3477 | 0.0690516923076923 | -0.0618877844311377 | 0.728521556886228 | 0.0651364870259481 |
| 3478 | 0.0846978461538462 | -0.348585788423154 | -0.182739520958084 | -0.00487305389221557 |
| 3479 | 0.0878270769230769 | -0.201094690618762 | -0.402839121756487 | -0.102496566866267 |
| 3480 | 0.0972147692307692 | 0.131085149700599 | 0.230251796407186 | 0.0505173253493014 |
| 3481 | 0.110983384615385 | -0.00292383233532934 | 0.370758183632735 | 0.108831536926148 |
| 3482 | 0.109940307692308 | -0.121501477045908 | -0.287510179640719 | -0.0752074650698603 |
| 3483 | 0.0778135384615385 | -0.0250150099800399 | -0.0211165668662675 | -0.0995727345309381 |
| 3484 | 0.0329612307692308 | 0.582329940119761 | 0.341113772455090 | 0.0948621157684631 |
| 3485 | -0.00479815384615385 | 0.657374970059880 | -0.116953293413174 | 0.0151064670658683 |
| 3486 | -0.000208615384615385 | -0.138069860279441 | -0.256241417165669 | -0.129135928143713 |
| 3487 | 0.0433920000000000 | -0.255672894211577 | 0.248119660678643 | 0.0466188822355289 |
| 3488 | 0.0734326153846154 | 0.224647784431138 | 0.0381722554890220 | 0.134658722554890 |
| 3489 | 0.0696775384615385 | 0.142293173652695 | -0.296038023952096 | -0.0701719760479042 |
| 3490 | 0.0315009230769231 | -0.163734610778443 | 0.0771566866267465 | -0.0794307784431138 |
| 3491 | -0.00521538461538462 | -0.0615629141716567 | 0.160404690618762 | 0.170556886227545 |
| 3492 | -0.0118910769230769 | 0.189886666666667 | -0.0962428143712575 | 0.0722836327345309 |
| 3493 | -0.0127255384615385 | 0.153176327345309 | -0.173399500998004 | -0.153013892215569 |
| 3494 | -0.0219046153846154 | -0.0115328942115768 | 0.217663073852295 | 0.0175429940119760 |
| 3495 | -0.0438092307692308 | -0.132059760479042 | 0.180709081836327 | 0.179328383233533 |
| 3496 | -0.0609156923076923 | -0.296444111776447 | -0.106801097804391 | -0.0417458283433134 |
| 3497 | -0.0627932307692308 | -0.517193453093812 | -0.0982732534930140 | -0.114354331337325 |
| 3498 | -0.0410972307692308 | -0.433052055888224 | 0.255023153692615 | 0.0836540918163673 |
| 3499 | -0.00208615384615385 | 0.204343393213573 | 0.222536127744511 | 0.0321621556886228 |
| 3500 | 0.0160633846153846 | 0.266068742514970 | -0.255429241516966 | -0.0510046307385230 |
| 3501 | 0.0202356923076923 | 0.0183551696606786 | -0.0767505988023952 | 0.0134821157684631 |
| 3502 | 0.0342129230769231 | 0.262820039920160 | 0.270454491017964 | -0.0193297804391218 |
| 3503 | 0.0504849230769231 | 0.294170019960080 | 0.136039421157685 | -0.0206292614770459 |
| 3504 | 0.0579950769230769 | 0.191023712574850 | -0.384159081836327 | 0.0826794810379242 |
| 3505 | 0.0377593846153846 | 0.0758572055888224 | -0.0893393213572854 | 0.110780758483034 |
| 3506 | -0.0104307692307692 | -0.0773191217564870 | 0.313905888223553 | 0.0107207185628743 |
| 3507 | -0.0615415384615385 | -0.0901514970059880 | -0.0592888223552894 | -0.0636745708582834 |
| 3508 | -0.0796910769230769 | 0.0438574850299401 | -0.250962275449102 | 0.0229033532934132 |
| 3509 | -0.0360904615384615 | 0.344849780439122 | -0.0320809381237525 | 0.0576644710578842 |
| 3510 | 0.0204443076923077 | 0.256485069860279 | 0.112080239520958 | -0.0558776846307385 |
| 3511 | 0.0469384615384615 | -0.0703344111776447 | -0.0259896207584830 | -0.0678978842315369 |
| 3512 | 0.0561175384615385 | -0.0818673053892216 | -0.0755323353293413 | 0.0162435129740519 |
| 3513 | 0.0567433846153846 | -0.0266393612774451 | -0.0994915169660679 | -0.00893393213572854 |
| 3514 | 0.0559089230769231 | -0.156425029940120 | 0.134821157684631 | 0.0285885828343313 |
| 3515 | 0.0715550769230769 | -0.438249980039920 | 0.180709081836327 | 0.124262874251497 |
| 3516 | 0.0945027692307692 | -0.399427984031936 | 0.0609131736526946 | -0.0362230339321357 |
| 3517 | 0.0838633846153846 | -0.0895017564870260 | -0.101928043912176 | -0.0791059081836327 |
| 3518 | 0.0344215384615385 | 0.0516543712574850 | -0.000406087824351297 | 0.159348862275449 |
| 3519 | 0.00125169230769231 | 0.170394451097804 | 0.281012774451098 | 0.108019361277445 |

| | | | | |
|---|---|---|---|---|
| 3520 | 0.0189840000000000 | 0.110943193612774 | -0.0186800399201597 | -0.185338483033932 |
| 3521 | 0.0648793846153846 | -0.148303273453094 | -0.159186427145709 | -0.126536966067864 |
| 3522 | 0.101804307692308 | -0.0121826347305389 | -0.162029041916168 | 0.0964864670658683 |
| 3523 | 0.0974233846153846 | 0.182252215568862 | 0.143349001996008 | 0.0329743313373254 |
| 3524 | 0.0659224615384615 | 0.00178678642714571 | 0.114110678642715 | -0.111430499001996 |
| 3525 | 0.0302492307692308 | -0.0651364870259481 | -0.332585928143713 | -0.0588015169660679 |
| 3526 | 0.0285803076923077 | 0.104283353293413 | -0.223348303393214 | 0.0251774451097804 |
| 3527 | 0.0446436923076923 | 0.213439760479042 | 0.265175349301397 | -0.0328118962075848 |
| 3528 | 0.0258683076923077 | 0.0706592814371258 | 0.147409880239521 | 0.0435326147704591 |
| 3529 | -0.00250338461538462 | -0.0393093013972056 | -0.307002395209581 | 0.0724460678642715 |
| 3530 | -0.0275372307692308 | -0.108019361277445 | 0.0942123752495010 | -0.107694491017964 |
| 3531 | -0.0310836923076923 | -0.214901676646707 | 0.248525748502994 | -0.0454818363273453 |
| 3532 | -0.0104307692307692 | -0.101359520958084 | -0.0146191616766467 | 0.181115169660679 |
| 3533 | 0.00187753846153846 | -0.0397966067864271 | -0.144973353293413 | 0.0201419560878244 |
| 3534 | -0.00604984615384615 | -0.0977859481037924 | 0.0410148702594810 | -0.195084590818363 |
| 3535 | -0.0402627692307692 | -0.0389844311377246 | 0.0889332335329341 | 0.0474310578842315 |
| 3536 | -0.0678000000000000 | 0.126049660678643 | -0.140100299401198 | 0.166820878243513 |
| 3537 | -0.0536141538461538 | 0.0488929740518962 | -0.0832480039920160 | -0.0508421956087824 |
| 3538 | -0.0120996923076923 | -0.104283353293413 | -0.0268017964071856 | -0.0703344111776447 |
| 3539 | 0.0173150769230769 | -0.0519792415169661 | -0.00203043912175649 | 0.126049660678643 |
| 3540 | -0.00250338461538462 | -0.0553903792415170 | -0.0219287425149701 | 0.0389844311377246 |
| 3541 | -0.0531969230769231 | 0.103633612774451 | -0.0450757485029940 | -0.109318842315369 |
| 3542 | -0.0930424615384616 | 0.231957365269461 | -0.0893393213572854 | 0.0701719760479042 |
| 3543 | -0.0844892307692308 | 0.0688724950099800 | 0.0739079840319361 | 0.134008982035928 |
| 3544 | -0.0342129230769231 | -0.0133196806387226 | 0.112080239520958 | -0.0903139321357285 |
| 3545 | -0.00292061538461538 | 0.144242395209581 | -0.120201996007984 | -0.141968303393214 |
| 3546 | -0.0181495384615385 | 0.175917245508982 | -0.132790718562874 | 0.0691973652694611 |
| 3547 | -0.0784393846153846 | -0.0560401197604790 | 0.100709780439122 | 0.0558776846307385 |
| 3548 | -0.107228307692308 | -0.0870652295409182 | 0.176648203592814 | -0.108506666666667 |
| 3549 | -0.0840720000000000 | 0.0363854690618763 | -0.0357357285429142 | -0.0555528143712575 |
| 3550 | -0.0509021538461538 | 0.392768143712575 | -0.119389820359281 | -0.0319997205588822 |
| 3551 | -0.0135600000000000 | 0.200444950099800 | 0.152282934131737 | -0.0571771656686627 |
| 3552 | -0.0208615384615385 | -0.779688622754491 | 0.139288123752495 | 0.0925880239520958 |
| 3553 | -0.0477729230769231 | -0.662410459081836 | -0.0661923153692615 | 0.0313499800399202 |
| 3554 | -0.0392196923076923 | 0.270454491017964 | -0.0406087824351297 | -0.216038722554890 |
| 3555 | -0.00417230769230769 | 0.400240159680639 | -0.0722836327345309 | -0.0410960878243513 |
| 3556 | 0.0410972307692308 | 0.126049660678643 | 0.0304565868263473 | 0.239429381237525 |
| 3557 | 0.0398455384615385 | 0.0196546506986028 | -0.105176746506986 | 0.0328118962075848 |
| 3558 | -0.00208615384615385 | -0.168932534930140 | 0.0929941117764471 | -0.225784830339321 |
| 3559 | -0.0304578461538462 | 0.443772774451098 | -0.206292614770459 | 0.0547406387225549 |
| 3560 | -0.0365076923076923 | 0.709516646706587 | 0.183957784431138 | 0.254373413173653 |
| 3561 | -0.00938769230769231 | -0.733719481037924 | 0.597761277445110 | -0.0282637125748503 |
| 3562 | 0.00688430769230769 | -0.865291936127745 | -0.598573453093812 | 0.00373600798403194 |
| 3563 | 0.00438092307692308 | 0.802591976047904 | -1.23613133732535 | 0.249662794411178 |

| | | | | |
|---|---|---|---|---|
| 3564 | 0.00479815384615385 | 0.776115049900200 | 0.521822854291417 | 0.0354108582834331 |
| 3565 | 0.0185667692307692 | -0.617578363273453 | 2.02434780439122 | -0.122476087824351 |
| 3566 | 0.0638363076923077 | -0.101197085828343 | -1.54069720558882 | 0.207592095808383 |
| 3567 | 0.115781538461538 | 0.855220958083832 | -1.41684041916168 | 0.194272415169661 |
| 3568 | 0.143736000000000 | 0.103633612774451 | 2.22211257485030 | -0.223673173652695 |
| 3569 | 0.100135384615385 | -0.345174650698603 | 1.20851736526946 | -0.0792683433133733 |
| 3570 | 0.0273286153846154 | -0.0719587624750499 | -2.08282445109780 | 0.339326986027944 |
| 3571 | -0.00375507692307692 | -0.437275369261477 | -0.872276646706587 | 0.121501477045908 |
| 3572 | 0.00938769230769231 | -0.510696047904192 | 2.44708522954092 | -0.255672894211577 |
| 3573 | 0.0575778461538462 | 0.270292055888224 | -0.277357984031936 | -0.0776439920159681 |
| 3574 | 0.0809427692307692 | 0.0727709381237525 | -1.24668962075848 | 0.184039001996008 |
| 3575 | 0.0682172307692308 | -0.386920479041916 | 0.524259381237525 | 0.0977859481037924 |
| 3576 | 0.0421403076923077 | 0.279225988023952 | 0.316342415169661 | -0.171044191616766 |
| 3577 | 0.0289975384615385 | 0.370514530938124 | -0.460097504990020 | -0.222211257485030 |
| 3578 | 0.0410972307692308 | -0.335266107784431 | 0.358169461077844 | -0.0220911776447106 |
| 3579 | 0.0388024615384615 | -0.245114610778443 | 0.505985429141717 | 0.0974610778443114 |
| 3580 | 0.0325440000000000 | 0.330880359281437 | -1.46435269461078 | -0.0440199201596806 |
| 3581 | 0.0106393846153846 | 0.00243652694610778 | 0.356139021956088 | -0.199470339321357 |
| 3582 | -0.0118910769230769 | -0.465863952095808 | 1.62881826347305 | 0.0180302994011976 |
| 3583 | 0.0248252307692308 | 0.145379441117764 | -0.554715968063872 | 0.247388702594810 |
| 3584 | 0.0821944615384615 | 0.474310578842315 | -1.53663632734531 | 0.00714714570858283 |
| 3585 | 0.125377846153846 | 0.0113704590818363 | 0.812581736526946 | -0.249662794411178 |
| 3586 | 0.134556923076923 | 0.0698471057884232 | 1.75551766467066 | 0.0206292614770459 |
| 3587 | 0.110357538461538 | 0.219774730538922 | -1.17399990019960 | 0.210678363273453 |
| 3588 | 0.0967975384615385 | -0.0461315768463074 | -1.17887295409182 | -0.0925880239520958 |
| 3589 | 0.102638769230769 | -0.0558776846307385 | 0.987605588822355 | -0.217175768463074 |
| 3590 | 0.127672615384615 | -0.192810499001996 | 0.958773353293413 | 0.0449945309381238 |
| 3591 | 0.123083076923077 | -0.413234970059880 | -0.678978842315369 | 0.100709780439122 |
| 3592 | 0.0688430769230769 | 0.0683851896207585 | -0.493802794411178 | -0.0641618762475050 |
| 3593 | 0.0104307692307692 | 0.379610898203593 | 0.823952195608782 | 0.0594512574850299 |
| 3594 | -0.0148116923076923 | -0.0700095409181637 | 0.0905575848303393 | 0.187937445109780 |
| 3595 | 0.0146030769230769 | -0.277276766467066 | -0.308626746506986 | -0.0349235528942116 |
| 3596 | 0.0473556923076923 | 0.126212095808383 | -0.0170556886227545 | -0.159998602794411 |
| 3597 | 0.0579950769230769 | 0.294332455089820 | 0.0406087824351297 | 0.0862530538922156 |
| 3598 | 0.0373421538461538 | -0.0688724950099800 | -0.287916267465070 | 0.113542155688623 |
| 3599 | 0.00688430769230769 | -0.141643433133733 | -0.0726897205588822 | -0.175754810379242 |
| 3600 | 0.00834461538461538 | 0.0891768862275449 | 0.229845708582834 | -0.0407712175648703 |
| 3601 | 0.0229476923076923 | -0.0532787225548902 | -0.532381137724551 | 0.225622395209581 |
| 3602 | 0.0461040000000000 | -0.172018802395210 | -0.0726897205588822 | -0.0388219960079840 |
| 3603 | 0.0206529230769231 | 0.0555528143712575 | 0.450351397205589 | -0.252911497005988 |
| 3604 | -0.0429747692307692 | 0.0355732934131737 | 0.0832480039920160 | 0.0573396007984032 |
| 3605 | -0.0728067692307692 | -0.0626999600798403 | -0.449539221556886 | 0.169582275449102 |
| 3606 | -0.0525710769230769 | 0.0462940119760479 | -0.160404690618762 | -0.0979483832335329 |
| 3607 | 0.000417230769230769 | 0.0635121357285429 | 0.404057385229541 | -0.0761820758483034 |

| | | | | |
|---|---|---|---|---|
| 3608 | 0.0446436923076923 | -0.0630248303393214 | -0.0284261477045908 | 0.135958203592814 |
| 3609 | 0.0680086153846154 | -0.0311875449101796 | -0.391874750499002 | 0.0175429940119760 |
| 3610 | 0.0604984615384615 | 0.121826347305389 | -0.0816236526946108 | -0.179815688622755 |
| 3611 | 0.0536141538461538 | -0.0531162874251497 | 0.495021057884232 | 0.0360605988023952 |
| 3612 | 0.0559089230769231 | -0.211652974051896 | -0.0536035928143713 | 0.153501197604790 |
| 3613 | 0.0413058461538462 | 0.00779688622754491 | -0.465782734530938 | -0.0909636726546906 |
| 3614 | -0.0152289230769231 | 0.146516487025948 | 0.0694410179640719 | -0.113379720558882 |
| 3615 | -0.109105846153846 | -0.0657862275449102 | 0.557558582834331 | 0.0729333732534930 |
| 3616 | -0.162720000000000 | -0.0729333732534930 | -0.0198983033932136 | 0.103471177644711 |
| 3617 | -0.147282461538462 | 0.283449301397206 | -0.477965369261477 | -0.00877149700598802 |
| 3618 | -0.0832375384615385 | 0.248688183632735 | 0.233094411177645 | 0.0235530938123753 |
| 3619 | -0.0131427692307692 | -0.127186706586826 | 0.304159780439122 | 0.118252774451098 |
| 3620 | 0.00751015384615385 | -0.147815968063872 | -0.108831536926148 | -0.0185176047904192 |
| 3621 | 0.00375507692307692 | -0.0126699401197605 | -0.322027644710579 | -0.108506666666667 |
| 3622 | -0.000625846153846154 | -0.0233906586826347 | -0.0121826347305389 | 0.0318372854291417 |
| 3623 | -0.000625846153846154 | 0.142780479041916 | 0.0215226546906188 | 0.00373600798403194 |
| 3624 | 0.0189840000000000 | 0.0472686227544910 | -0.0101521956087824 | -0.144079960079840 |
| 3625 | -0.00897046153846154 | -0.00763445109780439 | -0.0828419161676647 | -0.0417458283433134 |
| 3626 | -0.0711378461538462 | 0.353458842315369 | -0.278982335329341 | 0.101197085828343 |
| 3627 | -0.130801846153846 | -0.138719600798403 | 0.0982732534930140 | 0.0500300199600798 |
| 3628 | -0.168352615384615 | -0.742490978043912 | 0.235124850299401 | -0.0446696606786427 |
| 3629 | -0.126420923076923 | -0.254373413173653 | -0.0434513972055888 | 0.0186800399201597 |
| 3630 | -0.0703033846153846 | 0.375225149700599 | -0.428422654690619 | 0.111430499001996 |
| 3631 | -0.0321267692307692 | 0.215226546906188 | -0.00609131736526946 | 0.0477559281437126 |
| 3632 | -0.0331698461538462 | -0.182739520958084 | 0.262332734530938 | 0.0216038722554890 |
| 3633 | -0.0723895384615385 | -0.00990854291417166 | -0.00974610778443114 | 0.0633497005988024 |
| 3634 | -0.101595692307692 | 0.264281956087824 | -0.162029041916168 | 0.0711465868263473 |
| 3635 | -0.106811076923077 | 0.128811057884232 | -0.0438574850299401 | 0.0449945309381238 |
| 3636 | -0.0853236923076923 | -0.324870259481038 | 0.378067764471058 | 0.0134821157684631 |
| 3637 | -0.107436923076923 | -0.474635449101796 | 0.124668962075848 | 0.0623750898203593 |
| 3638 | -0.149577230769231 | 0.279875728542914 | -0.249744011976048 | 0.0631872654690619 |
| 3639 | -0.182329846153846 | 0.743628023952096 | -0.0231470059880240 | -0.00958367265469062 |
| 3640 | -0.179409230769231 | 0.0560401197604790 | 0.419894810379242 | -0.0255023153692615 |
| 3641 | -0.0980492307692308 | -0.243002954091816 | -0.00893393213572854 | 0.0186800399201597 |
| 3642 | -0.0248252307692308 | 0.274515369261477 | -0.257459680638723 | 0.0562025548902196 |
| 3643 | 0.0204443076923077 | 0.421031856287425 | 0.272891017964072 | 0.0230657884231537 |
| 3644 | 0.0143944615384615 | -0.00747201596806387 | 0.242028343313373 | -0.0474310578842315 |
| 3645 | -0.0114738461538462 | -0.266718483033932 | -0.142942914171657 | -0.0144567265469062 |
| 3646 | 0.00333784615384615 | -0.213927065868264 | -0.248119660678643 | 0.0566898602794411 |
| 3647 | 0 | 0.0449945309381238 | 0.176242115768463 | -0.0508421956087824 |
| 3648 | -0.0294147692307692 | 0.391631097804391 | 0.0869027944111777 | -0.0953494211576846 |
| 3649 | -0.104516307692308 | 0.0224160479041916 | -0.109643712574850 | 0.0212790019960080 |
| 3650 | -0.179200615384615 | -0.610431217564870 | -0.0613192614770459 | 0.0472686227544910 |
| 3651 | -0.179826461538462 | -0.106070139720559 | -0.0495427145708583 | -0.0428828742514970 |

| | | | | |
|---|---|---|---|---|
| 3652 | -0.131219076923077 | 0.529863393213573 | 0.162029041916168 | -0.0722836327345309 |
| 3653 | -0.0329612307692308 | -0.143755089820359 | 0.0726897205588822 | 0.0635121357285429 |
| 3654 | 0.0402627692307692 | -0.562025548902196 | -0.129948103792415 | 0.112729980039920 |
| 3655 | 0.0659224615384615 | 0.107856926147705 | -0.134821157684631 | -0.0259896207584830 |
| 3656 | 0.0872012307692308 | 0.386108303393214 | 0.311875449101796 | -0.120364431137725 |
| 3657 | 0.0934596923076923 | -0.0994102994011976 | 0.244058782435130 | -0.0204668263473054 |
| 3658 | 0.115572923076923 | -0.122313652694611 | -0.0962428143712575 | 0.0516543712574850 |
| 3659 | 0.108480000000000 | 0.125399920159681 | -0.0365479041916168 | -0.0446696606786427 |
| 3660 | 0.0567433846153846 | -0.166008702594810 | 0.116141117764471 | -0.0523041117764471 |
| 3661 | -0.0156461538461538 | -0.0383346906187625 | 0.201013473053892 | 0.0146191616766467 |
| 3662 | -0.0598726153846154 | 0.353133972055888 | -0.134415069860279 | -0.0592888223552894 |
| 3663 | -0.0308750769230769 | 0.110293453093812 | -0.144973353293413 | -0.0895017564870260 |
| 3664 | 0.0194012307692308 | -0.185988223552894 | 0.0479183632734531 | 0.0261520558882236 |
| 3665 | 0.0690516923076923 | 0.102334131736527 | 0.0345174650698603 | 0.0129948103792415 |
| 3666 | 0.0853236923076923 | 0.192160758483034 | -0.0751262475049900 | -0.0456442714570858 |
| 3667 | 0.0703033846153846 | -0.119714690618762 | -0.147409880239521 | 0.0255023153692615 |
| 3668 | 0.0488160000000000 | -0.00406087824351297 | 0.108425449101796 | 0.123775568862275 |
| 3669 | 0.0306664615384615 | 0.0578269061876247 | 0.0231470059880240 | 0.0786186027944112 |
| 3670 | 0.0379680000000000 | -0.194434850299401 | -0.0272078842315369 | -0.0443447904191617 |
| 3671 | 0.0146030769230769 | -0.168120359281437 | 0.0925880239520958 | 0.0251774451097804 |
| 3672 | -0.0373421538461538 | 0.122313652694611 | -0.141318562874252 | 0.103471177644711 |
| 3673 | -0.0824030769230769 | 0.0680603193612775 | 0.406087824351297 | -0.0672481437125748 |
| 3674 | -0.0744756923076923 | -0.182901956087824 | 0.404869560878244 | -0.144567265469062 |
| 3675 | -0.00500676923076923 | -0.0940499401197605 | -0.886895808383234 | 0.0402839121756487 |
| 3676 | 0.0523624615384615 | 0.00357357285429142 | -1.33643502994012 | 0.0851160079840319 |
| 3677 | 0.0784393846153846 | -0.169419840319361 | 1.37866816367265 | -0.105907704590818 |
| 3678 | 0.0552830769230769 | -0.166983313373254 | 1.62800608782435 | -0.177216726546906 |
| 3679 | 0.0317095384615385 | 0.0864154890219561 | -2.33500499001996 | 0.0503548902195609 |
| 3680 | 0.0104307692307692 | 0.0513295009980040 | -0.788622554890220 | 0.154150938123753 |
| 3681 | -0.00208615384615385 | 0.0867403592814371 | 2.76342764471058 | -0.119064950099800 |
| 3682 | 0.0106393846153846 | 0.284261477045908 | 0.567710778443114 | -0.195571896207585 |
| 3683 | -0.0173150769230769 | 0.0597761277445110 | -2.78982335329341 | 0.129948103792415 |
| 3684 | -0.0630018461538462 | -0.126212095808383 | 0.0499488023952096 | 0.125237485029940 |
| 3685 | -0.0993009230769231 | 0.0779688622754491 | 2.23104650698603 | -0.228221357285429 |
| 3686 | -0.0780221538461538 | 0.0274515369261477 | -0.797962574850299 | -0.0828419161676647 |
| 3687 | -0.0100135384615385 | -0.162922435129741 | -0.910042814371258 | 0.262495169660679 |
| 3688 | 0.0335870769230769 | 0.0698471057884232 | 0.455630538922156 | -0.0185176047904192 |
| 3689 | 0.0584123076923077 | 0.154150938123753 | -0.151064670658683 | -0.295307065868264 |
| 3690 | 0.0506935384615385 | -0.0753699001996008 | -0.387813872255489 | 0.188424750499002 |
| 3691 | 0.0517366153846154 | -0.0339489421157685 | 0.928316766467066 | 0.366453652694611 |
| 3692 | 0.0594553846153846 | 0.111268063872255 | -0.213602195608782 | -0.206455049900200 |
| 3693 | 0.0348387692307692 | 0.0126699401197605 | -1.62069650698603 | -0.244302435129741 |
| 3694 | 0.00187753846153846 | -0.145866746506986 | 0.824764371257485 | 0.321459121756487 |
| 3695 | -0.0634190769230769 | -0.0921007185628743 | 1.43552045908184 | 0.218312814371257 |

| | | | | |
|---|---|---|---|---|
| 3696 | -0.0967975384615385 | -0.0482432335329341 | -0.968925548902196 | -0.375550019960080 |
| 3697 | -0.0663396923076923 | -0.121826347305389 | -1.52932674650699 | -0.132547065868263 |
| 3698 | -0.00604984615384615 | 0.119227385229541 | 1.10821367265469 | 0.338677245508982 |
| 3699 | 0.0579950769230769 | 0.197196247504990 | 1.45257614770459 | -0.0352484231536926 |
| 3700 | 0.0675913846153846 | -0.0919382834331337 | -1.20445648702595 | -0.257784550898204 |
| 3701 | 0.0575778461538462 | -0.112892415169661 | -1.01846826347305 | 0.188262315369261 |
| 3702 | 0.0423489230769231 | 0.125562355289421 | 1.11795978043912 | 0.202394171656687 |
| 3703 | 0.0450609230769231 | 0.486493213572854 | 0.991260379241517 | -0.153988502994012 |
| 3704 | 0.0588295384615385 | 0.348748223552894 | -0.719181536926148 | 0.0765069461077844 |
| 3705 | 0.0325440000000000 | 0.0927504590818363 | -0.278982335329341 | 0.320646946107784 |
| 3706 | 0 | 0.118090339321357 | 0.860500099800399 | -0.112567544910180 |
| 3707 | -0.0592467692307692 | -0.146516487025948 | 0.128729840319361 | -0.316586067864271 |
| 3708 | -0.0846978461538462 | -0.0407712175648703 | -0.465782734530938 | 0.246251656686627 |
| 3709 | -0.0377593846153846 | 0.127511576846307 | 0.0605070858283433 | 0.279388423153693 |
| 3710 | 0.0204443076923077 | -0.536523233532934 | 0.268017964071856 | -0.372301317365269 |
| 3711 | 0.0867840000000000 | -0.780663233532934 | -0.376443413173653 | -0.157886946107784 |
| 3712 | 0.112860923076923 | 0.309276487025948 | -0.133196806387226 | 0.476909540918164 |
| 3713 | 0.116407384615385 | 0.648441037924152 | 0.242028343313373 | 0.161460518962076 |
| 3714 | 0.110357538461538 | -0.324058083832335 | -0.268830139720559 | -0.379286027944112 |
| 3715 | 0.106185230769231 | -0.274840239520958 | -0.211165668662675 | -0.0285885828343313 |
| 3716 | 0.110148923076923 | 0.433052055888224 | 0.324464171656687 | 0.333479321357285 |
| 3717 | 0.0569520000000000 | 0.0831667864271457 | 0.148628143712575 | -0.0916134131736527 |
| 3718 | -0.00813600000000000 | -0.339651856287425 | -0.507203692614771 | -0.299530379241517 |
| 3719 | -0.0550744615384615 | -0.109806147704591 | -0.0885271457085828 | 0.0948621157684631 |
| 3720 | -0.0427661538461539 | -0.202556606786427 | 0.427204391217565 | 0.194434850299401 |
| 3721 | 0.0267027692307692 | -0.163572175648703 | -0.0515731536926148 | -0.108506666666667 |
| 3722 | 0.0763532307692308 | 0.450757485029940 | -0.428016566866267 | -0.0989229940119761 |
| 3723 | 0.103473230769231 | 0.468950219560878 | 0.245683133732535 | 0.144892135728543 |
| 3724 | 0.0926252307692308 | -0.130922714570858 | 0.596136926147705 | 0.0633497005988024 |
| 3725 | 0.0903304615384615 | -0.0167308183632735 | -0.212790019960080 | -0.132059760479042 |
| 3726 | 0.123708923076923 | 0.422656207584830 | -0.388626047904192 | 0.0480807984031936 |
| 3727 | 0.152915076923077 | 0.0258271856287425 | 0.298880638722555 | 0.191998323353293 |
| 3728 | 0.144361846153846 | -0.351996926147705 | 0.525477644710579 | -0.0199795209580838 |
| 3729 | 0.0771876923076923 | -0.00601009980039920 | -0.389844311377246 | -0.0445072255489022 |
| 3730 | 0.0168978461538462 | 0.188912055888224 | -0.494208882235529 | 0.0985981237524950 |
| 3731 | -0.00187753846153846 | -0.127349141716567 | 0.387001696606786 | -0.0378473852295409 |
| 3732 | 0.0281630769230769 | -0.185013612774451 | 0.380910379241517 | -0.146029181636727 |
| 3733 | 0.0809427692307692 | 0.107044750499002 | -0.296850199600798 | 0.0471061876247505 |
| 3734 | 0.0909563076923077 | 0.0597761277445110 | -0.356951197604790 | 0.111268063872255 |
| 3735 | 0.0815686153846154 | -0.194759720558882 | 0.379692115768463 | -0.0350859880239521 |
| 3736 | 0.0496504615384615 | -0.105582834331337 | 0.192079540918164 | -0.0318372854291417 |
| 3737 | 0.0398455384615385 | 0.0940499401197605 | -0.214820459081836 | 0.134496287425150 |
| 3738 | 0.0673827692307692 | 0.0802429540918164 | -0.0836540918163673 | 0.0573396007984032 |
| 3739 | 0.0819858461538462 | 0.0462940119760479 | 0.000406087824351297 | -0.156912335329341 |

| | | | | |
|---|---|---|---|---|
| 3740 | 0.0769790769230769 | 0.0659486626746507 | 0.0657862275449102 | -0.0552279441117765 |
| 3741 | 0.0185667692307692 | 0.103146307385230 | 0.161622954091816 | 0.0792683433133733 |
| 3742 | -0.00792738461538461 | -0.00649740518962076 | 0.0621314371257485 | -0.0890144510978044 |
| 3743 | 0.0183581538461538 | -0.139206906187625 | -0.356951197604790 | -0.177866467065868 |
| 3744 | 0.0538227692307692 | -0.0589639520958084 | 0.00406087824351297 | 0.0326494610778443 |
| 3745 | 0.0884529230769231 | 0.0112080239520958 | 0.369539920159681 | 0.113054850299401 |
| 3746 | 0.0876184615384615 | -0.0178678642714571 | -0.0462940119760479 | -0.0201419560878244 |
| 3747 | 0.0826116923076923 | 0.156425029940120 | -0.336646806387226 | -0.0367103393213573 |
| 3748 | 0.0713464615384615 | 0.279550858283433 | 0.00609131736526946 | 0.0997351696606787 |
| 3749 | 0.0694689230769231 | -0.0409336526946108 | 0.325682435129741 | 0.0139694211576846 |
| 3750 | 0.0880356923076923 | -0.185013612774451 | -0.137257684630739 | -0.170232015968064 |
| 3751 | 0.0598726153846154 | 0.00844662674650699 | -0.427610479041916 | -0.0588015169660679 |
| 3752 | -0.0156461538461538 | 0.0289134530938124 | 0.00203043912175649 | 0.0433701796407186 |
| 3753 | -0.0759360000000000 | -0.184526307385230 | 0.263957085828343 | -0.0831667864271457 |
| 3754 | -0.0690516923076923 | -0.156425029940120 | -0.175429940119760 | -0.128323752495010 |
| 3755 | -0.00959630769230769 | 0.0233906586826347 | -0.245277045908184 | 0.0979483832335329 |
| 3756 | 0.0342129230769231 | -0.0594512574850299 | 0.294413672654691 | 0.125887225548902 |
| 3757 | 0.0329612307692308 | 0.0370352095808383 | 0.321215469061876 | -0.139044471057884 |
| 3758 | -0.00250338461538462 | 0.207429660678643 | -0.301723253493014 | -0.0914509780439122 |
| 3759 | -0.0258683076923077 | 0.00129948103792415 | -0.204262175648703 | 0.128323752495010 |
| 3760 | -0.0175236923076923 | -0.229683273453094 | 0.456442714570858 | -0.0129948103792415 |
| 3761 | 0 | -0.105257964071856 | 0.177460379241517 | -0.139694211576846 |
| 3762 | 0.0173150769230769 | 0.0562025548902196 | -0.383752994011976 | 0.0602634331337325 |
| 3763 | 0.00584123076923077 | -0.0256647504990020 | -0.192485628742515 | 0.0802429540918164 |
| 3764 | -0.0337956923076923 | -0.134008982035928 | 0.380098203592814 | -0.148790578842315 |
| 3765 | -0.0575778461538462 | -0.00276139720558882 | 0.192079540918164 | -0.0516543712574850 |
| 3766 | -0.0354646153846154 | 0.253886107784431 | -0.165277744510978 | 0.306515089820359 |
| 3767 | 0.00521538461538462 | 0.148140838323353 | -0.158374251497006 | 0.121988782435130 |
| 3768 | 0.0179409230769231 | -0.137420119760479 | 0.113298502994012 | -0.218962554890220 |
| 3769 | 0.00771876923076923 | -0.0527914171656687 | 0.414209580838323 | 0.127836447105788 |
| 3770 | -0.00834461538461538 | 0.188749620758483 | 0.0523853293413174 | 0.361093293413174 |
| 3771 | -0.00751015384615385 | 0.0402839121756487 | -0.307002395209581 | -0.0813800000000000 |
| 3772 | 0.00917907692307692 | -0.0682227544910180 | -0.255429241516966 | -0.168445229540918 |
| 3773 | 0.00688430769230769 | -0.0490554091816367 | 0.237561377245509 | 0.159186427145709 |
| 3774 | -0.0216960000000000 | -0.174455329341317 | 0.142536826347305 | 0.00584766467065868 |
| 3775 | -0.0934596923076923 | -0.406250259481038 | -0.378879940119761 | -0.220749341317365 |
| 3776 | -0.161051076923077 | -0.481132854291417 | -0.291977145708583 | -0.00795932135728543 |
| 3777 | -0.151037538461538 | 0.541396287425150 | 0.0816236526946108 | 0.150902235528942 |
| 3778 | -0.0828203076923077 | 1.06752367265469 | 0.264363173652695 | -0.0950245508982036 |
| 3779 | -0.0216960000000000 | 0.362717644710579 | -0.126699401197605 | -0.0508421956087824 |
| 3780 | -0.00938769230769231 | 0.0511670658682635 | -0.169338622754491 | 0.294657325349301 |
| 3781 | -0.0181495384615385 | -0.280038163672655 | 0.0373600798403194 | 0.0869027944111777 |
| 3782 | -0.0214873846153846 | -0.524827904191617 | 0.141724650698603 | -0.220749341317365 |
| 3783 | 0.0123083076923077 | 0.0376849500998004 | 0.0519792415169661 | 0.0196546506986028 |

| | | | | |
|---|---|---|---|---|
| 3784 | 0.0569520000000000 | 0.166983313373254 | -0.131978542914172 | 0.157074770459082 |
| 3785 | 0.0615415384615385 | -0.961128662674651 | 0.0357357285429142 | -0.184526307385230 |
| 3786 | 0.0346301538461538 | -0.383509341317365 | 0.417458283433134 | -0.149927624750499 |
| 3787 | -0.0229476923076923 | 1.58081868263473 | 0.333398103792415 | 0.119877125748503 |
| 3788 | -0.0652966153846154 | 0.447021477045908 | -1.16506596806387 | -0.0415833932135729 |
| 3789 | -0.0642535384615385 | -1.11657908183633 | -0.485274950099800 | -0.0745577245508982 |
| 3790 | -0.0456867692307692 | 0.149277884231537 | 1.99998253493014 | 0.224485349301397 |
| 3791 | -0.0350473846153846 | 0.818510618762475 | 0.172181237524950 | 0.0821921756487026 |
| 3792 | -0.0586209230769231 | -0.533599401197605 | -2.46414091816367 | -0.274677804391218 |
| 3793 | -0.0819858461538462 | -0.815261916167665 | 0.804053892215569 | 0.0703344111776447 |
| 3794 | -0.0817772307692308 | 0.287672614770459 | 2.86860439121757 | 0.300342554890220 |
| 3795 | -0.0538227692307692 | 0.219449860279441 | -1.64303133732535 | -0.192810499001996 |
| 3796 | -0.0277458461538462 | -0.0654613572854292 | -2.27124920159681 | -0.285885828343313 |
| 3797 | -0.0550744615384615 | 0.576482275449102 | 1.96871377245509 | 0.268992574850299 |
| 3798 | -0.0872012307692308 | 0.263794650698603 | 1.38029251497006 | 0.247388702594810 |
| 3799 | -0.108688615384615 | -0.447508782435130 | -1.68201576846307 | -0.264119520958084 |
| 3800 | -0.0824030769230769 | 0.331530099800399 | -0.300098902195609 | -0.0691973652694611 |
| 3801 | -0.0196098461538462 | 0.456442714570858 | 1.24912614770459 | 0.291895928143713 |
| 3802 | 0.0112652307692308 | -0.689049820359281 | -0.691567564870260 | 0.0516543712574850 |
| 3803 | 0.0254510769230769 | -0.444584950099800 | -0.127511576846307 | -0.0539284630738523 |
| 3804 | 0.0127255384615385 | 0.568685389221557 | 1.23897395209581 | 0.195247025948104 |
| 3805 | 0.00917907692307692 | 0.226272135728543 | -1.03552395209581 | 0.0188424750499002 |
| 3806 | 0.0331698461538462 | -0.521579201596806 | -1.12323892215569 | -0.188099880239521 |
| 3807 | 0.0527796923076923 | 0.0204668263473054 | 1.73074630738523 | 0.113217285429142 |
| 3808 | 0.0454781538461538 | 0.428503872255489 | 0.801617365269461 | 0.0963240319361277 |
| 3809 | -0.0175236923076923 | -0.100222475049900 | -2.14414371257485 | -0.355245628742515 |
| 3810 | -0.0680086153846154 | -0.230982754491018 | -0.495021057884232 | -0.203368782435130 |
| 3811 | -0.0611243076923077 | -0.00665984031936128 | 2.08810359281437 | 0.342575688622755 |
| 3812 | -0.0183581538461538 | -0.0232282235528942 | 0.0621314371257485 | 0.0558776846307385 |
| 3813 | 0.0325440000000000 | -0.0943748103792415 | -1.85541526946108 | -0.428503872255489 |
| 3814 | 0.0586209230769231 | 0.0191673453093812 | 0.110455888223553 | 0.0259896207584830 |
| 3815 | 0.0742670769230769 | 0.0890144510978044 | 1.44810918163673 | 0.454006187624751 |
| 3816 | 0.0602898461538462 | 0.221236646706587 | -0.345580738522954 | -0.0878774051896208 |
| 3817 | 0.0494418461538462 | 0.154800678642715 | -0.893393213572854 | -0.426879520958084 |
| 3818 | 0.0715550769230769 | -0.183226826347305 | 0.492584530938124 | 0.102009261477046 |
| 3819 | 0.0807341538461538 | -0.0474310578842315 | 0.480401896207585 | 0.293032974051896 |
| 3820 | 0.0623760000000000 | 0.275977285429142 | -0.390250399201597 | -0.152039281437126 |
| 3821 | 0.00667569230769231 | 0.0930753293413174 | -0.181521257485030 | -0.182577085828343 |
| 3822 | -0.0196098461538462 | -0.227896487025948 | 0.479995808383234 | 0.246901397205589 |
| 3823 | 0.00667569230769231 | -0.161947824351297 | 0.0637557884231537 | 0.183876566866267 |
| 3824 | 0.0590381538461538 | -0.0922631536926148 | -0.251368363273453 | -0.128323752495010 |
| 3825 | 0.116616000000000 | -0.147653532934132 | 0.174617764471058 | 0.0332992015968064 |
| 3826 | 0.130593230769231 | -0.0373600798403194 | -0.0105582834331337 | 0.189561796407186 |
| 3827 | 0.126838153846154 | 0.0886895808383234 | -0.231063972055888 | -0.0693598003992016 |

| | | | | |
|---|---|---|---|---|
| 3828 | 0.139980923076923 | -0.117115728542914 | 0.131978542914172 | -0.187287704590818 |
| 3829 | 0.167100923076923 | -0.156587465069860 | 0.207916966067864 | 0.0355732934131737 |
| 3830 | 0.179200615384615 | 0.169419840319361 | -0.255023153692615 | 0.0877149700598802 |
| 3831 | 0.150828923076923 | 0.0168932534930140 | -0.261114471057884 | -0.0511670658682635 |
| 3832 | 0.0899132307692308 | -0.313174930139721 | 0.356951197604790 | -0.0406087824351297 |
| 3833 | 0.0175236923076923 | -0.0428828742514970 | 0.178272554890220 | 0.0115328942115768 |
| 3834 | -0.00146030769230769 | 0.270941796407186 | -0.454006187624751 | 0.0123450698602794 |
| 3835 | 0.0312923076923077 | -0.0769942514970060 | -0.197358682634731 | 0.102983872255489 |
| 3836 | 0.0692603076923077 | -0.203368782435130 | 0.525883732534930 | 0.0209541317365269 |
| 3837 | 0.0878270769230769 | 0.231794930139721 | 0.221723952095808 | -0.192160758483034 |
| 3838 | 0.0673827692307692 | 0.129460798403194 | -0.684664071856287 | -0.0126699401197605 |
| 3839 | 0.0648793846153846 | -0.174455329341317 | -0.263144910179641 | 0.260221077844311 |
| 3840 | 0.0861581538461538 | 0.0687100598802395 | 0.528726347305389 | 0.0422331337325349 |
| 3841 | 0.108897230769231 | 0.270616926147705 | 0.209541317365269 | -0.284261477045908 |
| 3842 | 0.108897230769231 | -0.00909636726546906 | -0.483650598802395 | 0.0141318562874252 |
| 3843 | 0.0736412307692308 | -0.255185588822355 | -0.204262175648703 | 0.329093572854291 |
| 3844 | 0.0375507692307692 | -0.00779688622754491 | 0.512076746506986 | -0.114029461077844 |
| 3845 | -0.00876184615384615 | 0.221236646706587 | 0.201419560878244 | -0.335266107784431 |
| 3846 | -0.00959630769230769 | -0.00162435129740519 | -0.228627445109780 | 0.100384910179641 |
| 3847 | 0.0275372307692308 | -0.104283353293413 | -0.148222055888224 | 0.144079960079840 |
| 3848 | 0.0402627692307692 | -0.0328118962075848 | 0.112080239520958 | -0.180627864271457 |
| 3849 | 0.0400541538461538 | -0.124912614770459 | 0.220911776447106 | -0.101359520958084 |
| 3850 | 0.0321267692307692 | -0.132547065868263 | -0.0649740518962076 | 0.0469437524950100 |
| 3851 | 0.0390110769230769 | -0.137582554890220 | -0.346392914171657 | -0.0714714570858283 |
| 3852 | 0.0454781538461538 | 0.189074491017964 | -0.142536826347305 | -0.0683851896207585 |
| 3853 | 0.0296233846153846 | 0.463589860279441 | 0.376443413173653 | 0.135795768463074 |
| 3854 | -0.00396369230769231 | 0.0402839121756487 | 0.0885271457085828 | 0.0901514970059880 |
| 3855 | -0.0481901538461539 | -0.0134821157684631 | -0.387813872255489 | -0.178028902195609 |
| 3856 | -0.0732240000000000 | 0.0639994411177645 | -0.0767505988023952 | -0.0414209580838323 |
| 3857 | -0.0531969230769231 | -0.348260918163673 | 0.347611177644711 | 0.214414371257485 |
| 3858 | -0.00229476923076923 | -0.186637964071856 | 0.104770658682635 | -0.0116953293413174 |
| 3859 | 0.0477729230769231 | 0.396666586826347 | -0.270860578842315 | -0.207104790419162 |
| 3860 | 0.0725981538461538 | 0.0165683832335329 | -0.0804053892215569 | 0.0467813173652695 |
| 3861 | 0.0642535384615385 | -0.139369341317365 | 0.151470758483034 | 0.132709500998004 |
| 3862 | 0.0513193846153846 | 0.760033972055888 | 0.217256986027944 | -0.113704590818363 |
| 3863 | 0.0527796923076923 | 0.348748223552894 | -0.165277744510978 | -0.0638370059880239 |
| 3864 | 0.0546572307692308 | -0.653801397205589 | -0.321215469061876 | 0.147491097804391 |
| 3865 | 0.0536141538461538 | -0.152201716566866 | 0.245277045908184 | 0.0524665469061876 |
| 3866 | 0.0196098461538462 | 0.388869700598802 | 0.386189520958084 | -0.0550655089820359 |
| 3867 | -0.0264941538461538 | -0.198008423153693 | -0.145379441117764 | 0.0779688622754491 |
| 3868 | -0.0241993846153846 | -0.430615528942116 | -0.227815269461078 | 0.0903139321357285 |
| 3869 | 0.0143944615384615 | 0.198820598802395 | 0.330961576846307 | -0.0419082634730539 |
| 3870 | 0.0623760000000000 | 0.142942914171657 | 0.266393612774451 | -0.0115328942115768 |
| 3871 | 0.0782307692307692 | -0.131085149700599 | -0.326088522954092 | 0.0691973652694611 |

| | | | | |
|---|---|---|---|---|
| 3872 | 0.0682172307692308 | 0.191998323353293 | -0.332585928143713 | 0.0371976447105788 |
| 3873 | 0.0513193846153846 | -0.139856646706587 | 0.218881337325349 | -0.00617253493013972 |
| 3874 | 0.0356732307692308 | -0.524340598802395 | 0.155531636726547 | 0.0586390818363273 |
| 3875 | 0.0542400000000000 | 0.393742754491018 | -0.296444111776447 | 0.0665984031936128 |
| 3876 | 0.0607070769230769 | 0.707404990019960 | -0.173399500998004 | 0.00341113772455090 |
| 3877 | 0.0177323076923077 | -0.270779361277445 | 0.151064670658683 | 0.0990854291417166 |
| 3878 | -0.0333784615384615 | -0.227734051896208 | 0.168932534930140 | 0.141480998003992 |
| 3879 | -0.0492332307692308 | 0.583791856287425 | -0.107613273453094 | -0.0495427145708583 |
| 3880 | -0.0239907692307692 | 0.326657045908184 | -0.122232435129741 | -0.0873900998003992 |
| 3881 | 0.0150203076923077 | -0.321134251497006 | 0.151064670658683 | 0.136607944111776 |
| 3882 | 0.0375507692307692 | -0.140506387225549 | 0.259490119760479 | 0.103146307385230 |
| 3883 | 0.0335870769230769 | 0.118577644710579 | -0.0633497005988024 | -0.189399361277445 |
| 3884 | 0.0233649230769231 | -0.0968113373253493 | -0.208729141716567 | -0.155287984031936 |
| 3885 | 0.0383852307692308 | 0.121663912175649 | 0.0341113772455090 | 0.116465988023952 |
| 3886 | 0.0817772307692308 | 0.185825788423154 | 0.182739520958084 | 0.0662735329341317 |
| 3887 | 0.0986750769230769 | -0.313499800399202 | 0.0142130738522954 | -0.196708942115768 |
| 3888 | 0.0483987692307692 | -0.146354051896208 | -0.178272554890220 | -0.127674011976048 |
| 3889 | -0.0279544615384615 | 0.180140558882236 | 0.0138069860279441 | 0.104608223552894 |
| 3890 | -0.0886615384615385 | -0.0917758483033932 | 0.211571756487026 | 0.0531162874251497 |
| 3891 | -0.101595692307692 | -0.348910658682635 | 0.0280200598802395 | -0.0768318163672655 |
| 3892 | -0.0494418461538462 | -0.0716338922155689 | -0.104770658682635 | -0.0560401197604790 |
| 3893 | 0.00604984615384615 | 0.117927904191617 | -0.0235530938123753 | 0.0120201996007984 |
| 3894 | 0.0131427692307692 | -0.0825170459081836 | 0.0495427145708583 | 0.116465988023952 |
| 3895 | -0.0114738461538462 | 0.0474310578842315 | 0.0328931137724551 | 0.155612854291417 |
| 3896 | -0.0446436923076923 | 0.324870259481038 | -0.0369539920159681 | -0.00925880239520958 |
| 3897 | -0.0565347692307692 | -0.0667608383233533 | 0.00771566866267465 | -0.0532787225548902 |
| 3898 | -0.0515280000000000 | -0.311875449101796 | -0.0877149700598802 | 0.0890144510978044 |
| 3899 | -0.0561175384615385 | 0.141480998003992 | 0.408930439121757 | 0.0781312974051896 |
| 3900 | -0.0755187692307692 | 0.0911261077844311 | -0.0235530938123753 | 0.00633497005988024 |
| 3901 | -0.101178461538462 | -0.143105349301397 | -0.523041117764471 | 0.0594512574850299 |
| 3902 | -0.102847384615385 | 0.0688724950099800 | 0.143755089820359 | 0.0362230339321357 |
| 3903 | -0.0552830769230769 | 0.233419281437126 | 0.681415369261477 | -0.0778064271457086 |
| 3904 | 0.0100135384615385 | 0.0323245908183633 | -0.187206487025948 | -0.0339489421157685 |
| 3905 | 0.0419316923076923 | -0.117440598802395 | -0.879586227544910 | 0.0529538522954092 |
| 3906 | 0.0248252307692308 | -0.0121826347305389 | 0.836134830339321 | -0.0472686227544910 |
| 3907 | -0.0164806153846154 | -0.135958203592814 | 0.468625349301397 | -0.201257125748503 |
| 3908 | -0.0289975384615385 | -0.198333293413174 | -0.659486626746507 | -0.0955118562874252 |
| 3909 | -0.0173150769230769 | 0.0690349301397206 | -0.390656487025948 | 0.0774815568862275 |
| 3910 | -0.0164806153846154 | 0.141156127744511 | 0.463752295409182 | 0.0198170858283433 |
| 3911 | -0.0302492307692308 | -0.101034650698603 | 0.258271856287425 | -0.0885271457085828 |
| 3912 | -0.0575778461538462 | 0.0118577644710579 | -0.562025548902196 | -0.0914509780439122 |
| 3913 | -0.0832375384615385 | 0.178028902195609 | 0.225378742514970 | -0.00617253493013972 |
| 3914 | -0.0642535384615385 | -0.0890144510978044 | -0.114922854291417 | 0.0990854291417166 |
| 3915 | -0.0106393846153846 | -0.118252774451098 | -0.163247305389222 | 0.0605883033932136 |

| | | | | |
|---|---|---|---|---|
| 3916 | 0.0312923076923077 | -0.00129948103792415 | 0.207104790419162 | -0.117115728542914 |
| 3917 | 0.0304578461538462 | -0.0139694211576846 | -0.0203043912175649 | -0.0316748502994012 |
| 3918 | 0.00980492307692308 | 0.0238779640718563 | -0.408118263473054 | 0.201257125748503 |
| 3919 | 0.0166892307692308 | 0.0263144910179641 | -0.199389121756487 | 0.0485681037924152 |
| 3920 | 0.0467298461538462 | -0.0259896207584830 | 0.611974351297405 | -0.204993133732535 |
| 3921 | 0.0830289230769231 | 0.0203043912175649 | -0.116141117764471 | -0.0274515369261477 |
| 3922 | 0.0857409230769231 | 0.159348862275449 | -0.546594211576846 | 0.160323473053892 |
| 3923 | 0.0565347692307692 | -0.0255023153692615 | -0.0134008982035928 | -0.0419082634730539 |
| 3924 | 0.0179409230769231 | -0.245439481037924 | 0.624969161676647 | -0.176566986027944 |
| 3925 | -0.0202356923076923 | 0.00406087824351297 | 0.126699401197605 | -0.0255023153692615 |
| 3926 | -0.0127255384615385 | 0.463589860279441 | -0.612380439121756 | 0.0409336526946108 |
| 3927 | 0.0264941538461538 | 0.113217285429142 | 0.0860906187624751 | -0.151551976047904 |
| 3928 | 0.0342129230769231 | -0.794957524950100 | 0.438980938123753 | -0.0758572055888224 |
| 3929 | 0.00229476923076923 | -0.448970698602794 | 0.122232435129741 | 0.179328383233533 |
| 3930 | -0.0292061538461538 | 0.349073093812375 | -0.355326846307385 | -0.0160810778443114 |
| 3931 | -0.0394283076923077 | 0.202881477045908 | 0.0272078842315369 | -0.140993692614770 |
| 3932 | -0.0406800000000000 | 0.0636745708582834 | 0.260302295409182 | 0.226109700598802 |
| 3933 | -0.0438092307692308 | 0.115328942115768 | -0.0921819361277445 | 0.281987385229541 |
| 3934 | -0.0575778461538462 | -0.124750179640719 | -0.0523853293413174 | -0.146516487025948 |
| 3935 | -0.0773963076923077 | 0.520929461077844 | 0.0324870259481038 | -0.115491377245509 |
| 3936 | -0.0876184615384615 | 0.833941956087824 | -0.0215226546906188 | 0.304890738522954 |
| 3937 | -0.0698861538461539 | -0.616116447105788 | -0.188424750499002 | 0.212627584830339 |
| 3938 | -0.0444350769230769 | -0.743303153692615 | 0.145785528942116 | -0.175267504990020 |
| 3939 | -0.0442264615384615 | 0.785373852295409 | -0.0422331337325349 | -0.0311875449101796 |
| 3940 | -0.0594553846153846 | 0.656400359281437 | -0.310657185628743 | 0.256160199600798 |
| 3941 | -0.0705120000000000 | -0.707729860279441 | -0.0308626746506986 | -0.0360605988023952 |
| 3942 | -0.0609156923076923 | -0.225947265469062 | 0.195328243512974 | -0.267530658682635 |
| 3943 | -0.0346301538461538 | 0.817373572854292 | -0.0312687624750499 | 0.0547406387225549 |
| 3944 | 0.000417230769230769 | 0.162597564870259 | -0.217256986027944 | 0.188587185628743 |
| 3945 | 0.00980492307692308 | -0.365641477045908 | 0.230251796407186 | -0.0930753293413174 |
| 3946 | -0.0141858461538462 | -0.0446696606786427 | 0.181115169660679 | -0.102009261477046 |
| 3947 | -0.0350473846153846 | -0.288159920159681 | -0.0101521956087824 | 0.0911261077844311 |
| 3948 | -0.0609156923076923 | -0.633009700598802 | -0.0308626746506986 | 0.0235530938123753 |
| 3949 | -0.0552830769230769 | -0.0134821157684631 | 0.239591816367265 | -0.0701719760479042 |
| 3950 | -0.0304578461538462 | 0.145054570858283 | 0.0747201596806387 | -0.0523041117764471 |
| 3951 | -0.0365076923076923 | -0.260058642714571 | -0.215632634730539 | -0.00146191616766467 |
| 3952 | -0.0636276923076923 | 0.157237205588822 | -0.0150252495009980 | 0.0367103393213573 |
| 3953 | -0.0982578461538461 | 0.417620718562874 | 0.110455888223553 | 0.0246901397205589 |
| 3954 | -0.0773963076923077 | -0.00990854291417166 | 0.0454818363273453 | -0.0251774451097804 |
| 3955 | -0.0281630769230769 | -0.106882315369261 | -0.277357984031936 | -0.0207916966067864 |
| 3956 | 0.00959630769230769 | 0.265906307385230 | 0.0605070858283433 | 0.0998976047904192 |
| 3957 | 0.0246166153846154 | 0.131897325349301 | 0.276139720558882 | 0.134983592814371 |
| 3958 | 0.0221132307692308 | -0.249662794411178 | 0.0686288423153693 | -0.0246901397205589 |
| 3959 | 0.0577864615384615 | -0.00389844311377246 | -0.162841217564870 | -0.167633053892216 |

| | | | | |
|---|---|---|---|---|
| 3960 | 0.0901218461538462 | 0.327794091816367 | 0.0203043912175649 | -0.0194922155688623 |
| 3961 | 0.124126153846154 | 0.203368782435130 | 0.307408483033932 | 0.0115328942115768 |
| 3962 | 0.142901538461538 | -0.0389844311377246 | -0.00243652694610778 | -0.124587744510978 |
| 3963 | 0.113904000000000 | 0.0242028343313373 | -0.173399500998004 | 0.0401214770459082 |
| 3964 | 0.0740584615384615 | 0.0938875049900200 | -0.0775627744510978 | 0.132059760479042 |
| 3965 | 0.0494418461538462 | -0.0557152495009980 | 0.144161177644711 | -0.0253398802395210 |
| 3966 | 0.0903304615384615 | -0.341113772455090 | 0.0426392215568862 | 0.0180302994011976 |
| 3967 | 0.131636307692308 | -0.452381836327345 | -0.105176746506986 | 0.222536127744511 |
| 3968 | 0.122040000000000 | -0.0682227544910180 | -0.0767505988023952 | 0.161947824351297 |
| 3969 | 0.0930424615384616 | 0.174942634730539 | 0.0357357285429142 | -0.0571771656686627 |
| 3970 | 0.0575778461538462 | -0.0409336526946108 | 0.0438574850299401 | -0.0555528143712575 |
| 3971 | 0.0527796923076923 | -0.130435409181637 | 0.0580705588822355 | 0.156100159680639 |
| 3972 | 0.0619587692307692 | 0.0833292215568862 | -0.0633497005988024 | 0.0527914171656687 |
| 3973 | 0.0786480000000000 | 0.120364431137725 | -0.252992714570858 | -0.253886107784431 |
| 3974 | 0.0680086153846154 | 0.0542533333333333 | 0.103958483033932 | -0.130435409181637 |
| 3975 | 0.0256596923076923 | 0.156912335329341 | 0.136445508982036 | 0.0985981237524950 |
| 3976 | 0.0154375384615385 | 0.00779688622754491 | -0.256647504990020 | 0.0854408782435130 |
| 3977 | 0.0271200000000000 | -0.147328662674651 | -0.257459680638723 | -0.0843038323353293 |
| 3978 | 0.0725981538461538 | 0.109806147704591 | 0.366697305389222 | -0.161460518962076 |
| 3979 | 0.109940307692308 | 0.146841357285429 | 0.263957085828343 | 0.0318372854291417 |
| 3980 | 0.114738461538462 | -0.0752074650698603 | -0.242028343313373 | 0.144404830339321 |
| 3981 | 0.111609230769231 | -0.0436950499001996 | 0.121014171656687 | -0.00536035928143713 |
| 3982 | 0.0957544615384615 | 0.0307002395209581 | 0.386595608782435 | -0.146516487025948 |
| 3983 | 0.102012923076923 | -0.00536035928143713 | 0.0203043912175649 | -0.0575020359281437 |
| 3984 | 0.104307692307692 | -0.0485681037924152 | -0.351265968063872 | -0.0115328942115768 |
| 3985 | 0.0765618461538462 | -0.0334616367265469 | 0.0767505988023952 | -0.0854408782435130 |
| 3986 | 0.0164806153846154 | -0.00373600798403194 | 0.190455189620759 | -0.0352484231536926 |
| 3987 | -0.0486073846153846 | -0.151551976047904 | -0.263550998003992 | 0.00129948103792415 |
| 3988 | -0.0567433846153846 | -0.124425309381238 | -0.209541317365269 | -0.0384971257485030 |
| 3989 | -0.0294147692307692 | 0.0972986427145709 | 0.0812175648702595 | 0.0614004790419162 |
| 3990 | 0.0152289230769231 | 0.00714714570858283 | 0.0978671656686627 | 0.184526307385230 |
| 3991 | 0.0538227692307692 | -0.121988782435130 | -0.127511576846307 | -0.0196546506986028 |
| 3992 | 0.0569520000000000 | 0.0540908982035928 | 0.00893393213572854 | -0.123775568862275 |
| 3993 | 0.0473556923076923 | 0.309438922155689 | 0.0840601796407186 | 0.100547345309381 |
| 3994 | 0.0348387692307692 | 0.175917245508982 | 0.00771566866267465 | 0.108506666666667 |
| 3995 | 0.0475643076923077 | -0.0852784431137725 | 0.0450757485029940 | -0.129460798403194 |
| 3996 | 0.0438092307692308 | -0.113054850299401 | 0.0121826347305389 | -0.156425029940120 |
| 3997 | -0.0127255384615385 | 0.0677354491017964 | -0.0527914171656687 | 0.0865779241516966 |
| 3998 | -0.0736412307692308 | 0.0784561676646707 | -0.164871656686627 | 0.0940499401197605 |
| 3999 | -0.119745230769231 | 0.00665984031936128 | 0.129948103792415 | -0.0250150099800399 |
| 4000 | -0.104933538461538 | 0.0492178443113772 | 0.100303692614770 | 0.114029461077844 |
| 4001 | -0.0404713846153846 | 0.223023433133733 | -0.184769960079840 | 0.0748825948103793 |
| 4002 | 0.0302492307692308 | 0.403976167664671 | -0.124262874251497 | -0.133846546906188 |
| 4003 | 0.0596640000000000 | -0.522391377245509 | 0.172181237524950 | 0.0461315768463074 |

| | | | | |
|---|---|---|---|---|
| 4004 | 0.0461040000000000 | -0.912073253493014 | 0.0860906187624751 | 0.238292335329341 |
| 4005 | 0.0362990769230769 | -0.0891768862275449 | -0.244870958083832 | -0.0266393612774451 |
| 4006 | 0.00917907692307692 | 0.553741357285429 | -0.0714714570858283 | -0.191186147704591 |
| 4007 | -0.00438092307692308 | 0.309601357285429 | 0.0791871257485030 | 0.115004071856287 |
| 4008 | -0.0373421538461538 | -0.319347465069860 | -0.00893393213572854 | 0.226109700598802 |
| 4009 | -0.105350769230769 | 0.0566898602794411 | -0.146191616766467 | -0.165196526946108 |
| 4010 | -0.135600000000000 | 0.463589860279441 | 0.0873088822355290 | -0.244140000000000 |
| 4011 | -0.132470769230769 | 0.199957644710579 | -0.161622954091816 | 0.144892135728543 |
| 4012 | -0.0773963076923077 | -0.723323632734531 | 0.457254890219561 | 0.103796047904192 |
| 4013 | -0.0156461538461538 | -0.716014051896208 | 0.392686926147705 | -0.147653532934132 |
| 4014 | 0.0131427692307692 | 0.643567984031936 | -0.931971556886228 | 0.0258271856287425 |
| 4015 | 0.0279544615384615 | 0.849210858283433 | -0.823952195608782 | 0.0776439920159681 |
| 4016 | 0.0177323076923077 | -0.226597005988024 | 1.38678992015968 | -0.110780758483034 |
| 4017 | 0.0221132307692308 | -0.180790299401198 | 1.19146167664671 | 0.0118577644710579 |
| 4018 | 0.0325440000000000 | 0.621964111776447 | -2.18272205588822 | 0.196059201596806 |
| 4019 | 0.0189840000000000 | 0.341925948103792 | -0.178678642714571 | -0.0717963273453094 |
| 4020 | -0.0371335384615385 | -0.125887225548902 | 2.12302714570858 | -0.212140279441118 |
| 4021 | -0.112026461538462 | -0.398453373253493 | 0.0816236526946108 | 0.155287984031936 |
| 4022 | -0.123917538461538 | -0.445072255489022 | -2.05602265469062 | 0.238617205588822 |
| 4023 | -0.0857409230769231 | -0.0519792415169661 | 0.205480439121756 | -0.127511576846307 |
| 4024 | -0.0208615384615385 | 0.409986267465070 | 1.71815758483034 | -0.161460518962076 |
| 4025 | 0.0273286153846154 | -0.147978403193613 | -0.795119960079840 | 0.186150658682635 |
| 4026 | 0.0333784615384615 | -0.674430658682635 | -0.240810079840319 | 0.207267225548902 |
| 4027 | 0.0377593846153846 | 0.311388143712575 | 0.243246606786427 | -0.0510046307385230 |
| 4028 | 0.0221132307692308 | 0.518817804391218 | -0.121014171656687 | -0.124750179640719 |
| 4029 | 0.00917907692307692 | -0.471061876247505 | 0.0544157684630739 | 0.0225784830339321 |
| 4030 | -0.00354646153846154 | -0.514269620758483 | 0.804053892215569 | 0.139206906187625 |
| 4031 | -0.0536141538461538 | 0.363204950099800 | -0.434107884231537 | 0.0742328542914172 |
| 4032 | -0.121622769230769 | 0.270454491017964 | -1.39328732534930 | -0.106557445109780 |
| 4033 | -0.168144000000000 | -0.257297245508982 | 1.16425379241517 | -0.114191896207585 |
| 4034 | -0.143110153846154 | 0.0740704191616766 | 1.00100648702595 | 0.175592375249501 |
| 4035 | -0.0794824615384615 | 0.199145469061876 | -1.06679271457086 | 0.182739520958084 |
| 4036 | -0.00292061538461538 | -0.0864154890219561 | -1.11917804391218 | -0.156587465069860 |
| 4037 | 0.0521538461538462 | 0.137744990019960 | 1.21217215568862 | -0.196059201596806 |
| 4038 | 0.0582036923076923 | 0.339164550898204 | 0.908012375249501 | 0.100222475049900 |
| 4039 | 0.0442264615384615 | 0 | -1.22110608782435 | 0.128973493013972 |
| 4040 | 0.0158547692307692 | -0.0682227544910180 | -0.411773053892216 | -0.140506387225549 |
| 4041 | -0.0127255384615385 | 0.0909636726546906 | 0.740704191616767 | -0.151876846307385 |
| 4042 | -0.0582036923076923 | -0.0677354491017964 | 0.504361077844311 | 0.0974610778443114 |
| 4043 | -0.109940307692308 | -0.0506797604790419 | -0.556340319361277 | 0.134983592814371 |
| 4044 | -0.143527384615385 | 0.202881477045908 | 0.0848723552894212 | -0.0898266267465070 |
| 4045 | -0.133931076923077 | -0.0706592814371258 | 0.440605289421158 | -0.0591263872255489 |
| 4046 | -0.0636276923076923 | -0.362067904191617 | -0.198170858283433 | 0.0903139321357285 |
| 4047 | 0.00897046153846154 | -0.0817048702594810 | 0.0154313373253493 | -0.00925880239520958 |

| | | | | |
|---|---|---|---|---|
| 4048 | 0.0557003076923077 | 0.174780199600798 | 0.0913697604790419 | -0.0635121357285429 |
| 4049 | 0.0686344615384615 | -0.0151064670658683 | 0.0207104790419162 | 0.00162435129740519 |
| 4050 | 0.0425575384615385 | -0.193947544910180 | -0.328118962075848 | -0.00341113772455090 |
| 4051 | 0.0273286153846154 | 0.00601009980039920 | 0.153501197604790 | -0.00617253493013972 |
| 4052 | 0.0292061538461538 | -0.0417458283433134 | -0.0231470059880240 | 0.0469437524950100 |
| 4053 | 0.0302492307692308 | -0.115491377245509 | -0.325276347305389 | -0.0563649900199601 |
| 4054 | 0.0148116923076923 | -0.0462940119760479 | 0.143755089820359 | -0.165846267465070 |
| 4055 | -0.0358818461538462 | -0.112729980039920 | 0.162029041916168 | -0.0185176047904192 |
| 4056 | -0.0617501538461538 | 0.120039560878244 | -0.120201996007984 | 0.0841413972055888 |
| 4057 | -0.0346301538461538 | 0.246251656686627 | -0.382940818363274 | -0.131897325349301 |
| 4058 | 0.0181495384615385 | 0.0500300199600798 | 0.196952594810379 | -0.205642874251497 |
| 4059 | 0.0709292307692308 | -0.0695222355289421 | 0.183551696606786 | 0.134983592814371 |
| 4060 | 0.0917907692307692 | -0.0154313373253493 | -0.167308183632735 | 0.115166506986028 |
| 4061 | 0.0861581538461538 | 0.0586390818363273 | -0.207916966067864 | -0.288159920159681 |
| 4062 | 0.0788566153846154 | -0.0271266666666667 | 0.229033532934132 | -0.131085149700599 |
| 4063 | 0.0771876923076923 | -0.0469437524950100 | 0.272484930139721 | 0.317073373253493 |
| 4064 | 0.0648793846153846 | 0.0204668263473054 | -0.319185029940120 | 0.0873900998003992 |
| 4065 | 0.0337956923076923 | 0.0711465868263473 | -0.242028343313373 | -0.269642315369262 |
| 4066 | -0.0173150769230769 | 0.0940499401197605 | 0.136445508982036 | 0.134821157684631 |
| 4067 | -0.0694689230769231 | 0.0479183632734531 | 0.363854690618762 | 0.411773053892216 |
| 4068 | -0.0778135384615385 | 0.0217663073852295 | -0.265175349301397 | -0.0612380439121757 |
| 4069 | -0.0306664615384615 | 0.0781312974051896 | -0.324870259481038 | -0.193785109780439 |
| 4070 | 0.0327526153846154 | -0.0417458283433134 | 0.190049101796407 | 0.297743592814371 |
| 4071 | 0.0732240000000000 | -0.207916966067864 | 0.268017964071856 | 0.244952175648703 |
| 4072 | 0.0807341538461538 | -0.103471177644711 | -0.0836540918163673 | -0.253561237524950 |
| 4073 | 0.0644621538461539 | 0.0734206786427146 | -0.250962275449102 | -0.117440598802395 |
| 4074 | 0.0659224615384615 | 0.182414650698603 | 0.113704590818363 | 0.268342834331337 |
| 4075 | 0.0721809230769231 | 0.117440598802395 | 0.0791871257485030 | 0.0601009980039920 |
| 4076 | 0.0515280000000000 | 0.110943193612774 | 0.0820297405189621 | -0.256322634730539 |
| 4077 | -0.00521538461538462 | 0.147166227544910 | -0.116547205588822 | -0.0155937724550898 |
| 4078 | -0.0713464615384615 | -0.0990854291417166 | -0.0954306387225549 | 0.201094690618762 |
| 4079 | -0.0957544615384615 | -0.119064950099800 | 0.220505688622755 | -0.0547406387225549 |
| 4080 | -0.0640449230769231 | -0.0102334131736527 | 0.226597005988024 | -0.144242395209581 |
| 4081 | 0.0160633846153846 | 0.0367103393213573 | -0.0913697604790419 | 0.0818673053892216 |
| 4082 | 0.0830289230769231 | 0.262820039920160 | -0.255429241516966 | 0.0864154890219561 |
| 4083 | 0.108897230769231 | 0.0558776846307385 | 0.101521956087824 | -0.105907704590818 |
| 4084 | 0.101595692307692 | -0.199145469061876 | 0.120608083832335 | -0.0601009980039920 |
| 4085 | 0.0842806153846154 | 0.0651364870259481 | -0.0925880239520958 | 0.126212095808383 |
| 4086 | 0.0872012307692308 | -0.0605883033932136 | -0.192485628742515 | 0.0630248303393214 |
| 4087 | 0.0809427692307692 | -0.620177325349301 | 0.0613192614770459 | -0.111917804391218 |
| 4088 | 0.0448523076923077 | -0.392118403193613 | 0.134821157684631 | -0.0368727744510978 |
| 4089 | -0.0123083076923077 | 0.320971816367266 | -0.0978671656686627 | 0.125887225548902 |
| 4090 | -0.0538227692307692 | 0.329905748502994 | -0.165277744510978 | 0.0268017964071856 |
| 4091 | -0.0375507692307692 | -0.00438574850299401 | 0.00609131736526946 | -0.105907704590818 |

| | | | | |
|---|---|---|---|---|
| 4092 | 0.0267027692307692 | -0.0118577644710579 | 0.214008283433134 | 0.0102334131736527 |
| 4093 | 0.0903304615384615 | 0.210191057884232 | -0.0718775449101796 | 0.100384910179641 |
| 4094 | 0.117450461538462 | 0.174780199600798 | -0.142130738522954 | -0.0149440319361277 |
| 4095 | 0.110148923076923 | -0.119714690618762 | 0.155531636726547 | -0.0362230339321357 |
| 4096 | 0.104933538461538 | -0.167795489021956 | 0.211571756487026 | 0.0324870259481038 |
| 4097 | 0.116198769230769 | -0.0458067065868264 | -0.128729840319361 | -0.0383346906187625 |
| 4098 | 0.118702153846154 | 0.199307904191617 | -0.110455888223553 | -0.0984356886227545 |
| 4099 | 0.0915821538461538 | 0.445559560878244 | 0.190049101796407 | -0.0321621556886228 |
| 4100 | 0.0271200000000000 | 0.219937165668663 | 0.0860906187624751 | 0.0776439920159681 |
| 4101 | -0.0348387692307692 | -0.136445508982036 | -0.0158374251497006 | 0.0498675848303393 |
| 4102 | -0.0344215384615385 | -0.0930753293413174 | -0.0523853293413174 | -0.101846826347305 |
| 4103 | 0.0388024615384615 | 0.0849535728542914 | 0.122232435129741 | -0.0791059081836327 |
| 4104 | 0.117867692307692 | -0.108181796407186 | 0.149034231536926 | 0.102496566866267 |
| 4105 | 0.152080615384615 | -0.278738682634731 | 0.0994915169660679 | 0.140343952095808 |
| 4106 | 0.139355076923077 | -0.0560401197604790 | -0.00609131736526946 | -0.0332992015968064 |
| 4107 | 0.103890461538462 | 0.107694491017964 | 0.0609131736526946 | -0.109968582834331 |
| 4108 | 0.0999267692307692 | -0.00259896207584830 | 0.279794510978044 | 0.0911261077844311 |
| 4109 | 0.108062769230769 | 0.00844662674650699 | 0.109643712574850 | 0.186962834331337 |
| 4110 | 0.114738461538462 | 0.121176606786427 | -0.0198983033932136 | -0.0441823552894212 |
| 4111 | 0.0890787692307692 | -0.203206347305389 | -0.143755089820359 | -0.133196806387226 |
| 4112 | 0.0164806153846154 | -0.262332734530938 | 0.121014171656687 | 0.0740704191616766 |
| 4113 | -0.0438092307692308 | 0.159023992015968 | 0.0438574850299401 | 0.0396341716566866 |
| 4114 | -0.0634190769230769 | 0.219287425149701 | -0.152282934131737 | -0.149765189620759 |
| 4115 | -0.0133513846153846 | 0.0505173253493014 | -0.0795932135728543 | -0.0217663073852295 |
| 4116 | 0.0542400000000000 | 0.177541596806387 | -0.0621314371257485 | 0.102009261477046 |
| 4117 | 0.0982578461538461 | 0.244952175648703 | 0.0259896207584830 | -0.0352484231536926 |
| 4118 | 0.103681846153846 | 0.00373600798403194 | -0.192891716566866 | -0.00162435129740519 |
| 4119 | 0.0861581538461538 | -0.0555528143712575 | -0.101115868263473 | 0.206942355289421 |
| 4120 | 0.0949200000000000 | -0.0652989221556886 | -0.160810778443114 | 0.0719587624750499 |
| 4121 | 0.0838633846153846 | -0.220099600798403 | -0.0820297405189621 | -0.195409461077844 |
| 4122 | 0.0365076923076923 | -0.251449580838323 | -0.0844662674650699 | -0.0830043512974052 |
| 4123 | -0.0519452307692308 | 0.118740079840319 | -0.0475122754491018 | 0.128161317365269 |
| 4124 | -0.123291692307692 | 0.205642874251497 | -0.0194922155688623 | -0.0784561676646707 |
| 4125 | -0.115572923076923 | -0.167795489021956 | -0.209947405189621 | -0.243327824351297 |
| 4126 | -0.0488160000000000 | -0.0722836327345309 | 0.566492514970060 | 0.131085149700599 |
| 4127 | 0.0506935384615385 | 0.0618877844311377 | 0.150252495009980 | 0.163247305389222 |
| 4128 | 0.0844892307692308 | -0.201906866267465 | -1.26861836327345 | -0.271753972055888 |
| 4129 | 0.0686344615384615 | -0.287835049900200 | 0.0430453093812375 | -0.0396341716566866 |
| 4130 | 0.0569520000000000 | -0.0605883033932136 | 2.17825508982036 | 0.418432894211577 |
| 4131 | 0.0446436923076923 | 0.0331367664670659 | -0.515731536926148 | 0.0240403992015968 |
| 4132 | 0.0584123076923077 | 0.0440199201596806 | -2.08282445109780 | -0.353133972055888 |
| 4133 | 0.0446436923076923 | 0.264119520958084 | 1.88546576846307 | 0.121988782435130 |
| 4134 | 0.00542400000000000 | 0.347611177644711 | 1.74374111776447 | 0.401702075848303 |
| 4135 | -0.0590381538461538 | 0.157074770459082 | -2.18028552894212 | -0.0930753293413174 |

| | | | | |
|---|---|---|---|---|
| 4136 | -0.124752000000000 | -0.0181927345309381 | -1.06151357285429 | -0.316748502994012 |
| 4137 | -0.119745230769231 | -0.116953293413174 | 2.00932255489022 | 0.175592375249501 |
| 4138 | -0.0794824615384615 | -0.0867403592814371 | 0.120608083832335 | 0.266718483033932 |
| 4139 | -0.0381766153846154 | -0.0102334131736527 | -1.73196457085828 | -0.196546506986028 |
| 4140 | -0.0410972307692308 | -0.0659486626746507 | 0.505579341317365 | -0.0948621157684631 |
| 4141 | -0.0584123076923077 | -0.0165683832335329 | 0.767912075848303 | 0.279063552894212 |
| 4142 | -0.0542400000000000 | 0.192160758483034 | -0.911667165668663 | 0.0529538522954092 |
| 4143 | -0.0544486153846154 | 0.101359520958084 | 0.175429940119760 | -0.124912614770459 |
| 4144 | -0.0344215384615385 | -0.144242395209581 | 0.655831836327345 | 0.172343672654691 |
| 4145 | -0.0354646153846154 | -0.0735831137724551 | -1.12729980039920 | 0.0576644710578842 |
| 4146 | -0.0688430769230769 | -0.0258271856287425 | -0.295631936127745 | -0.310738403193613 |
| 4147 | -0.0922080000000000 | -0.199470339321357 | 1.43998742514970 | -0.102496566866267 |
| 4148 | -0.0928338461538462 | -0.182901956087824 | -0.257053592814371 | 0.154475808383234 |
| 4149 | -0.0373421538461538 | 0.0994102994011976 | -1.58252425149701 | -0.116953293413174 |
| 4150 | 0.00813600000000000 | -0.0334616367265469 | 0.587202994011976 | -0.289621836327345 |
| 4151 | 0.00646707692307692 | -0.229195968063872 | 1.63734610778443 | 0.0605883033932136 |
| 4152 | -0.0179409230769231 | -0.114841636726547 | -0.725678942115768 | 0.221399081836327 |
| 4153 | -0.0456867692307692 | -0.243490259481038 | -1.45217005988024 | -0.113867025948104 |
| 4154 | -0.0219046153846154 | 0.404788343313373 | 0.826794810379242 | -0.159511297405190 |
| 4155 | 0.0100135384615385 | 1.20380674650699 | 1.17115728542914 | 0.133846546906188 |
| 4156 | 0.0158547692307692 | 0.609619041916168 | -0.747607684630739 | 0.0229033532934132 |
| 4157 | -0.0204443076923077 | -0.346961437125749 | -0.775221656686627 | -0.186637964071856 |
| 4158 | -0.0786480000000000 | -0.458067065868263 | 0.706998902195609 | 0.0151064670658683 |
| 4159 | -0.0932510769230769 | 0.0763445109780439 | 0.506797604790419 | 0.117765469061876 |
| 4160 | -0.0719723076923077 | 0.142618043912176 | -0.522635029940120 | -0.0558776846307385 |
| 4161 | -0.0123083076923077 | -0.432402315369262 | -0.177054291417166 | -0.0245277045908184 |
| 4162 | 0.0471470769230769 | -0.932864950099800 | 0.479589720558882 | 0.146516487025948 |
| 4163 | 0.0465212307692308 | -0.0896641916167665 | 0.0255835329341317 | 0.141480998003992 |
| 4164 | 0.0227390769230769 | 1.12372622754491 | -0.181927345309381 | 0.00990854291417166 |
| 4165 | 0.0173150769230769 | 0.200932255489022 | 0.343144211576846 | -0.0138069860279441 |
| 4166 | 0.0308750769230769 | -0.902652015968064 | 0.0523853293413174 | 0.109156407185629 |
| 4167 | 0.0554916923076923 | -0.00698471057884232 | -0.244058782435130 | 0.0540908982035928 |
| 4168 | 0.0483987692307692 | 0.650227824351297 | 0.256241417165669 | -0.0605883033932136 |
| 4169 | 0.0108480000000000 | -0.157724510978044 | 0.367509481037924 | 0.0269642315369261 |
| 4170 | -0.0131427692307692 | -0.590289261477046 | -0.230251796407186 | 0.0311875449101796 |
| 4171 | 0.00396369230769231 | -0.135308463073852 | -0.409336526946108 | -0.0823546107784431 |
| 4172 | 0.0640449230769231 | 0.141156127744511 | 0.259490119760479 | -0.0129948103792415 |
| 4173 | 0.121831384615385 | 0.253561237524950 | 0.211165668662675 | 0.0532787225548902 |
| 4174 | 0.127046769230769 | 0.452381836327345 | -0.360199900199601 | -0.0495427145708583 |
| 4175 | 0.0846978461538462 | 0.180465429141717 | -0.116141117764471 | -0.0685476247504990 |
| 4176 | 0.0607070769230769 | -0.0950245508982036 | 0.371164271457086 | 0.0646491816367266 |
| 4177 | 0.0463126153846154 | 0.338189940119761 | 0.0288322355289421 | 0.117278163672655 |
| 4178 | 0.0369249230769231 | 0.321621556886228 | -0.467407085828343 | -0.0834916566866268 |
| 4179 | 0.0264941538461538 | -0.394879800399202 | 0.113704590818363 | -0.0934001996007984 |

| | | | | |
|---|---|---|---|---|
| 4180 | -0.00667569230769231 | -0.296281676646707 | 0.442635728542914 | 0.0805678243512974 |
| 4181 | -0.0187753846153846 | 0.270292055888224 | -0.269642315369262 | -0.0220911776447106 |
| 4182 | -0.00229476923076923 | 0.00129948103792415 | -0.447914870259481 | -0.133846546906188 |
| 4183 | 0.0486073846153846 | -0.371001836327345 | 0.391874750499002 | 0.0524665469061876 |
| 4184 | 0.101595692307692 | -0.0220911776447106 | 0.483650598802395 | 0.111917804391218 |
| 4185 | 0.110983384615385 | 0.180140558882236 | -0.418270459081836 | -0.118740079840319 |
| 4186 | 0.0755187692307692 | -0.0906388023952096 | -0.207104790419162 | -0.0524665469061876 |
| 4187 | 0.0379680000000000 | -0.205318003992016 | 0.343550299401198 | 0.182739520958084 |
| 4188 | 0.0235735384615385 | -0.0368727744510978 | 0.112486327345309 | 0.0375225149700599 |
| 4189 | 0.0346301538461538 | 0.132059760479042 | -0.156749900199601 | -0.225135089820359 |
| 4190 | 0.0567433846153846 | -0.0523041117764471 | -0.215226546906188 | -0.0295631936127745 |
| 4191 | 0.0404713846153846 | -0.0669232734530938 | -0.0393905189620759 | 0.155287984031936 |
| 4192 | 0.00125169230769231 | 0.203531217564870 | 0.0994915169660679 | -0.0589639520958084 |
| 4193 | -0.0394283076923077 | 0.230657884231537 | 0.194109980039920 | -0.101846826347305 |
| 4194 | -0.0325440000000000 | 0.0490554091816367 | -0.0219287425149701 | 0.128973493013972 |
| 4195 | 0.00834461538461538 | -0.143917524950100 | -0.177460379241517 | 0.113217285429142 |
| 4196 | 0.0312923076923077 | -0.0963240319361277 | 0.0759384231536926 | -0.0747201596806387 |
| 4197 | 0.0350473846153846 | 0.177541596806387 | 0.240403992015968 | 0.0904763672654691 |
| 4198 | 0.0175236923076923 | 0.264281956087824 | 0.00893393213572854 | 0.179978123752495 |
| 4199 | 0.0235735384615385 | -0.128323752495010 | -0.186394311377246 | -0.0644867465069860 |
| 4200 | 0.0283716923076923 | -0.196708942115768 | 0.0231470059880240 | -0.0730958083832335 |
| 4201 | 0.0219046153846154 | 0.0354108582834331 | 0.207510878243513 | 0.169094970059880 |
| 4202 | 0.0146030769230769 | -0.00779688622754491 | 0.0726897205588822 | 0.0755323353293413 |
| 4203 | -0.0360904615384615 | -0.0432077445109780 | -0.211977844311377 | -0.0974610778443114 |
| 4204 | -0.0776049230769231 | -0.00113704590818363 | -0.00893393213572854 | 0.0570147305389222 |
| 4205 | -0.0686344615384615 | -0.0474310578842315 | 0.237967465069860 | 0.0779688622754491 |
| 4206 | -0.0154375384615385 | -0.0951869860279441 | -0.0901514970059880 | -0.0229033532934132 |
| 4207 | 0.0383852307692308 | 0.0519792415169661 | -0.259490119760479 | 0.0274515369261477 |
| 4208 | 0.0327526153846154 | 0.0378473852295409 | 0.127105489021956 | 0.0830043512974052 |
| 4209 | 0.00229476923076923 | -0.0903139321357285 | 0.0471061876247505 | -0.0885271457085828 |
| 4210 | -0.0239907692307692 | -0.0154313373253493 | -0.257459680638723 | -0.152201716566866 |
| 4211 | -0.0216960000000000 | 0.168932534930140 | -0.156343812375250 | 0.144404830339321 |
| 4212 | 0.00604984615384615 | -0.0485681037924152 | 0.00324870259481038 | 0.106882315369261 |
| 4213 | 0.0204443076923077 | -0.221886387225549 | 0.0759384231536926 | -0.272891017964072 |
| 4214 | 0.0108480000000000 | 0.101034650698603 | -0.128323752495010 | -0.166333572854291 |
| 4215 | -0.0325440000000000 | 0.0614004790419162 | -0.187612574850299 | 0.190373972055888 |
| 4216 | -0.0452695384615385 | -0.0987605588822355 | -0.0706592814371258 | 0.117603033932136 |
| 4217 | -0.0164806153846154 | 0.132384630738523 | 0.257459680638723 | -0.135633333333333 |
| 4218 | 0.0100135384615385 | 0.223673173652695 | 0.190861277445110 | -0.120526866267465 |
| 4219 | 0.0415144615384615 | -0.101684391217565 | -0.273703193612774 | 0.133521676646707 |
| 4220 | 0.0183581538461538 | -0.210353493013972 | -0.0836540918163673 | 0.213602195608782 |
| 4221 | -0.0248252307692308 | 0.0319997205588822 | 0.337865069860279 | 0.0177054291417166 |
| 4222 | -0.0333784615384615 | 0.0622126546906188 | 0.225784830339321 | -0.145866746506986 |
| 4223 | -0.0319181538461538 | -0.0404463473053892 | -0.359793812375250 | -0.135958203592814 |

| | | | | |
|---|---|---|---|---|
| 4224 | -0.00813600000000000 | -0.0269642315369261 | -0.272078842315369 | 0.0134821157684631 |
| 4225 | -0.0164806153846154 | -0.179653253493014 | 0.218881337325349 | 0.0968113373253493 |
| 4226 | -0.0296233846153846 | -0.217013333333333 | 0.161216866267465 | -0.0760196407185629 |
| 4227 | -0.0285803076923077 | -0.0769942514970060 | -0.170962974051896 | -0.209541317365269 |
| 4228 | -0.00959630769230769 | -0.228546227544910 | -0.132384630738523 | 0.0397966067864271 |
| 4229 | 0.0408886153846154 | 0.115004071856287 | 0.268830139720559 | 0.237967465069860 |
| 4230 | 0.0506935384615385 | 0.766044071856287 | 0.171369061876248 | 0.0472686227544910 |
| 4231 | 0.0410972307692308 | 0.313499800399202 | -0.00852784431137725 | -0.156262594810379 |
| 4232 | -0.0123083076923077 | -0.294170019960080 | 0.00609131736526946 | 0.00211165668662675 |
| 4233 | -0.0561175384615385 | -0.0584766470658688 | 0.0324870259481038 | 0.192160758483034 |
| 4234 | -0.0233649230769231 | -0.346961437125749 | -0.00609131736526946 | 0.0328118962075848 |
| 4235 | 0.0106393846153846 | -0.234718762475050 | -0.0552279441117765 | -0.148140838323353 |
| 4236 | 0.0289975384615385 | 0.440686506986028 | -0.0743140718562874 | -0.0420706986027944 |
| 4237 | -0.0102221538461538 | -0.323570778443114 | 0.140100299401198 | 0.115491377245509 |
| 4238 | -0.0640449230769231 | -0.190049101796407 | 0.563649900199601 | 0.00324870259481038 |
| 4239 | -0.0696775384615385 | 1.06330035928144 | -0.731364171656687 | -0.0503548902195609 |
| 4240 | -0.0523624615384615 | 0.351996926147705 | -1.06719880239521 | 0.0529538522954092 |
| 4241 | -0.000625846153846154 | -0.725597724550898 | 1.43552045908184 | -0.0445072255489022 |
| 4242 | 0.0337956923076923 | -0.0268017964071856 | 1.06638662674651 | -0.107369620758483 |
| 4243 | 0.0333784615384615 | 0.535223752495010 | -2.14089500998004 | 0.0620502195608782 |
| 4244 | 0.0143944615384615 | -0.343875169660679 | -0.601009980039920 | 0.0717963273453094 |
| 4245 | 0.0175236923076923 | -0.513132574850299 | 2.74109281437126 | -0.0784561676646707 |
| 4246 | 0.0623760000000000 | 0.315449021956088 | -0.225378742514970 | -0.0404463473053892 |
| 4247 | 0.0659224615384615 | 0.170719321357285 | -2.32607105788423 | 0.0227409181636727 |
| 4248 | 0.0294147692307692 | -0.188912055888224 | 0.757759880239521 | -0.0284261477045908 |
| 4249 | -0.0310836923076923 | 0.244627305389222 | 1.46475878243513 | -0.0685476247504990 |
| 4250 | -0.0667569230769231 | 0.0246901397205589 | -0.862936626746507 | -0.0544157684630739 |
| 4251 | -0.0529883076923077 | -0.511508223552894 | -0.539284630738523 | 0.0107207185628743 |
| 4252 | -0.0317095384615385 | 0.233906586826347 | 0.987605588822355 | 0.00730958083832335 |
| 4253 | 0.00479815384615385 | 0.483082075848303 | -0.570959481037924 | -0.0168932534930140 |
| 4254 | 0.00938769230769231 | -0.329093572854291 | -0.267611876247505 | 0.0344362475049900 |
| 4255 | -0.00312923076923077 | -0.239429381237525 | 1.33968373253493 | 0.0402839121756487 |
| 4256 | -0.000834461538461538 | 0.452219401197605 | -0.516949800399202 | 0.0516543712574850 |
| 4257 | 0.00625846153846154 | 0.291895928143713 | -1.45013962075848 | 0.0453194011976048 |
| 4258 | 0.0325440000000000 | -0.317073373253493 | 0.874713173652695 | -0.0605883033932136 |
| 4259 | 0.0235735384615385 | -0.0214414371257485 | 1.26090269461078 | -0.0651364870259481 |
| 4260 | -0.0114738461538462 | 0.179328383233533 | -1.24709570858283 | 0.0471061876247505 |
| 4261 | -0.0486073846153846 | -0.142618043912176 | -0.915321956087824 | 0.00487305389221557 |
| 4262 | -0.0492332307692308 | 0.000162435129740519 | 1.12811197604790 | -0.0549030738522954 |
| 4263 | 0.0120996923076923 | 0.0735831137724551 | 0.778064271457086 | 0.0602634331337325 |
| 4264 | 0.0680086153846154 | -0.122638522954092 | -0.905981936127745 | 0.0792683433133733 |
| 4265 | 0.102847384615385 | -0.0844662674650699 | -0.503142814371258 | -0.0646491816367266 |
| 4266 | 0.0846978461538462 | 0.0417458283433134 | 0.925474151696607 | -0.0763445109780439 |
| 4267 | 0.0594553846153846 | -0.0211165668662675 | 0.152689021956088 | 0.124262874251497 |

| | | | | |
|---|---|---|---|---|
| 4268 | 0.0567433846153846 | 0.0729333732534930 | -0.447508782435130 | 0.106395009980040 |
| 4269 | 0.0673827692307692 | -0.0294007584830339 | 0.0637557884231537 | -0.100709780439122 |
| 4270 | 0.0907476923076923 | -0.196871377245509 | 0.365479041916168 | 0.0272891017964072 |
| 4271 | 0.0571606153846154 | 0.117603033932136 | -0.0966489021956088 | 0.228871097804391 |
| 4272 | 0.00250338461538462 | 0.257459680638723 | -0.151470758483034 | 0.0659486626746507 |
| 4273 | -0.0369249230769231 | 0.0164059481037924 | 0.410554790419162 | -0.130272974051896 |
| 4274 | -0.0323353846153846 | -0.206617485029940 | -0.0316748502994012 | -0.0362230339321357 |
| 4275 | 0.00959630769230769 | 0.00779688622754491 | -0.303753692614771 | 0.141156127744511 |
| 4276 | 0.0373421538461538 | 0.125237485029940 | 0.110455888223553 | 0.0675730139720559 |
| 4277 | 0.0615415384615385 | -0.0856033133732535 | 0.162029041916168 | -0.128161317365269 |
| 4278 | 0.0607070769230769 | 0.0326494610778443 | -0.301723253493014 | -0.0513295009980040 |
| 4279 | 0.0709292307692308 | 0.222698562874252 | -0.109643712574850 | 0.0458067065868264 |
| 4280 | 0.111817846153846 | 0.0555528143712575 | 0.209541317365269 | 0.00974610778443114 |
| 4281 | 0.135391384615385 | -0.0752074650698603 | -0.126293313373253 | -0.0112080239520958 |
| 4282 | 0.121831384615385 | 0.0714714570858283 | -0.216850898203593 | -0.0420706986027944 |
| 4283 | 0.0515280000000000 | -0.0433701796407186 | 0.00893393213572854 | 0.0601009980039920 |
| 4284 | -0.0112652307692308 | -0.275814850299401 | 0.198983033932136 | 0.112080239520958 |
| 4285 | -0.0327526153846154 | -0.0724460678642715 | -0.121420259481038 | -0.0778064271457086 |
| 4286 | -0.0371335384615385 | 0.0761820758483034 | -0.215632634730539 | -0.103633612774451 |
| 4287 | -0.0198184615384615 | -0.0924255888223553 | 0.148628143712575 | 0.107694491017964 |
| 4288 | -0.0241993846153846 | -0.139369341317365 | 0.218475249500998 | 0.0399590419161677 |
| 4289 | -0.0396369230769231 | 0.0875525349301397 | -0.181115169660679 | -0.187287704590818 |
| 4290 | -0.0475643076923077 | 0.0997351696606787 | -0.248525748502994 | -0.0284261477045908 |
| 4291 | -0.0317095384615385 | -0.212140279441118 | 0.148222055888224 | 0.204668263473054 |
| 4292 | -0.00312923076923077 | -0.0755323353293413 | 0.169338622754491 | -0.0266393612774451 |
| 4293 | -0.0239907692307692 | 0.190861277445110 | -0.0771566866267465 | -0.193135369261477 |
| 4294 | -0.0619587692307692 | 0.0358981636726547 | -0.231876147704591 | 0.133521676646707 |
| 4295 | -0.108062769230769 | -0.133521676646707 | 0.148222055888224 | 0.208891576846307 |
| 4296 | -0.117867692307692 | 0.0908012375249501 | 0.220911776447106 | -0.168282794411178 |
| 4297 | -0.0613329230769231 | 0.160161037924152 | -0.116547205588822 | -0.231632495009980 |
| 4298 | -0.0131427692307692 | 0.00503548902195609 | -0.0950245508982036 | 0.115328942115768 |
| 4299 | 0.0158547692307692 | 0.0859281836327345 | 0.00934001996007984 | 0.129785668662675 |
| 4300 | 0.00563261538461538 | -0.00860906187624751 | -0.0162435129740519 | -0.151227105788423 |
| 4301 | -0.0183581538461538 | -0.145866746506986 | -0.00365479041916168 | -0.0113704590818363 |
| 4302 | -0.00959630769230769 | 0.0713090219560878 | -0.136445508982036 | 0.164546786427146 |
| 4303 | -0.00604984615384615 | 0.369702355289421 | -0.159186427145709 | -0.00438574850299401 |
| 4304 | 0.00709292307692308 | 0.103471177644711 | 0.157968163672655 | 0.0354108582834331 |
| 4305 | 0.00500676923076923 | -0.408849221556886 | 0.180709081836327 | 0.130110538922156 |
| 4306 | -0.0196098461538462 | -0.441173812375250 | -0.0690349301397206 | -0.0467813173652695 |
| 4307 | -0.0415144615384615 | 0.0430453093812375 | -0.204668263473054 | -0.0677354491017964 |
| 4308 | -0.0544486153846154 | 0.239591816367265 | 0.186394311377246 | 0.0880398403193613 |
| 4309 | -0.0219046153846154 | -0.00487305389221557 | 0.194109980039920 | 0.0425580039920160 |
| 4310 | -0.00792738461538461 | -0.130272974051896 | -0.0714714570858283 | -0.196059201596806 |
| 4311 | -0.0150203076923077 | -0.0144567265469062 | -0.103146307385230 | -0.134171417165669 |

| | | | | |
|---|---|---|---|---|
| 4312 | -0.0212787692307692 | 0.734531656686627 | -0.0365479041916168 | 0.230657884231537 |
| 4313 | -0.0191926153846154 | 0.692623393213573 | 0.171775149700599 | -0.000649740518962076 |
| 4314 | 0.0191926153846154 | -0.467000998003992 | 0.0385783433133733 | -0.316423632734531 |
| 4315 | 0.0429747692307692 | -0.428503872255489 | -0.179084730538922 | 0.103308742514970 |
| 4316 | 0.0433920000000000 | 0.390981357285429 | -0.138882035928144 | 0.213764630738523 |
| 4317 | 0.00208615384615385 | 0.239429381237525 | 0.273297105788423 | -0.198170858283433 |
| 4318 | -0.0406800000000000 | -0.334291497005988 | 0.112892415169661 | -0.0992478642714571 |
| 4319 | -0.0304578461538462 | -0.0389844311377246 | -0.250556187624750 | 0.258271856287425 |
| 4320 | 0.0123083076923077 | 0.338839680638723 | -0.0162435129740519 | 0.0758572055888224 |
| 4321 | 0.0757273846153846 | 0.132709500998004 | 0.231470059880240 | -0.229520838323353 |
| 4322 | 0.0999267692307692 | 0.0667608383233533 | -0.0402026946107784 | -0.00617253493013972 |
| 4323 | 0.0751015384615385 | -0.148790578842315 | -0.382128642714571 | 0.289621836327345 |
| 4324 | 0.0431833846153846 | -0.478371457085828 | 0.0377661676646707 | -0.0765069461077844 |
| 4325 | 0.0388024615384615 | -0.570634610778443 | 0.208729141716567 | -0.327631656686627 |
| 4326 | 0.0602898461538462 | -0.227084311377246 | -0.0730958083832335 | 0.134658722554890 |
| 4327 | 0.0604984615384615 | 0.144242395209581 | -0.0942123752495010 | 0.262982475049900 |
| 4328 | 0.0360904615384615 | 0.0116953293413174 | 0.166089920159681 | -0.111268063872255 |
| 4329 | -0.00250338461538462 | 0.129948103792415 | 0.187612574850299 | -0.0558776846307385 |
| 4330 | -0.0191926153846154 | 0.366128782435130 | 0.0560401197604790 | 0.235693373253493 |
| 4331 | -0.000625846153846154 | 0.201744431137725 | 0.0621314371257485 | 0.0812175648702595 |
| 4332 | 0.0379680000000000 | 0.0501924550898204 | 0.0324870259481038 | -0.0886895808383234 |
| 4333 | 0.0663396923076923 | 0.0992478642714571 | 0.216444810379242 | 0.0102334131736527 |
| 4334 | 0.0385938461538462 | 0.0579893413173653 | 0.161216866267465 | 0.0568522954091816 |
| 4335 | -0.00292061538461538 | -0.142942914171657 | -0.152282934131737 | 0.00568522954091816 |
| 4336 | -0.0296233846153846 | -0.118577644710579 | -0.165683832335329 | -0.0243652694610778 |
| 4337 | -0.0137686153846154 | 0.316261197604790 | 0.141318562874252 | -0.00568522954091816 |
| 4338 | 0.0198184615384615 | 0.337540199600798 | 0.0389844311377246 | 0.00552279441117765 |
| 4339 | 0.00479815384615385 | -0.0584766467065868 | -0.292789321357285 | 0.0180302994011976 |
| 4340 | -0.00876184615384615 | -0.0589639520958084 | -0.128323752495010 | 0.0290758882235529 |
| 4341 | -0.0227390769230769 | 0.0722836327345309 | 0.0848723552894212 | -0.0180302994011976 |
| 4342 | -0.0118910769230769 | -0.0753699001996008 | -0.00365479041916168 | -0.0206292614770459 |
| 4343 | 0.0256596923076923 | -0.470249700598802 | -0.108019361277445 | 0.0620502195608782 |
| 4344 | 0.0271200000000000 | -0.355245628742515 | 0.129542015968064 | -0.0126699401197605 |
| 4345 | 0.00292061538461538 | -0.00243652694610778 | -0.0568522954091816 | -0.0523041117764471 |
| 4346 | -0.0661310769230769 | -0.0388219960079840 | 0.0190861277445110 | 0.0523041117764471 |
| 4347 | -0.131010461538462 | 0.0495427145708583 | 0.129135928143713 | 0.0121826347305389 |
| 4348 | -0.131010461538462 | 0.0825170459081836 | -0.0353296407185629 | -0.0930753293413174 |
| 4349 | -0.0890787692307692 | -0.148465708582834 | -0.0962428143712575 | -0.0435326147704591 |
| 4350 | -0.0492332307692308 | -0.135308463073852 | -0.0852784431137725 | 0.0414209580838323 |
| 4351 | -0.0373421538461538 | 0.141480998003992 | 0.694410179640719 | -0.0503548902195609 |
| 4352 | -0.0440178461538462 | 0.163409740518962 | 0.0576644710578842 | 0.0274515369261477 |
| 4353 | -0.0579950769230769 | 0.0547406387225549 | -1.10861976047904 | 0.141643433133733 |
| 4354 | -0.0333784615384615 | 0.0607507385229541 | -0.440605289421158 | -0.100384910179641 |
| 4355 | 0.0129341538461538 | 0.190698842315369 | 1.71166017964072 | -0.194272415169661 |

| | | | | |
|---|---|---|---|---|
| 4356 | 0.0273286153846154 | 0.147815968063872 | 0.147003792415170 | 0.0417458283433134 |
| 4357 | 0.00521538461538462 | -0.0521416766467066 | -2.22536127744511 | 0.0734206786427146 |
| 4358 | -0.0335870769230769 | -0.0696846706586826 | 0.823140019960080 | -0.208404271457086 |
| 4359 | -0.0506935384615385 | -0.187775009980040 | 1.87734401197605 | -0.224972654690619 |
| 4360 | -0.0377593846153846 | -0.187612574850299 | -1.00466127744511 | 0.0464564471057884 |
| 4361 | -0.0160633846153846 | -0.0472686227544910 | -1.79206556886228 | 0.0721211976047904 |
| 4362 | -0.00521538461538462 | 0.00389844311377246 | 1.37014031936128 | -0.0644867465069860 |
| 4363 | -0.0194012307692308 | 0.0594512574850299 | 1.03674221556886 | -0.0396341716566866 |
| 4364 | -0.0308750769230769 | -0.0612380439121757 | -1.40059690618762 | 0.0118577644710579 |
| 4365 | 0.00312923076923077 | -0.105745269461078 | 0.0816236526946108 | 0.0565274251497006 |
| 4366 | 0.0506935384615385 | 0.135795768463074 | 0.680197105788423 | 0.152526586826347 |
| 4367 | 0.0771876923076923 | 0.0497051497005988 | -0.324870259481038 | -0.0206292614770459 |
| 4368 | 0.0554916923076923 | -0.332017405189621 | -0.0917758483033932 | -0.173155848303393 |
| 4369 | 0.0160633846153846 | -0.0367103393213573 | 0.824764371257485 | 0.192972934131737 |
| 4370 | 0.00312923076923077 | 0.435813453093812 | -0.719993712574850 | 0.427854131736527 |
| 4371 | 0.0120996923076923 | 0.148303273453094 | -0.759384231536926 | -0.0110455888223553 |
| 4372 | 0.0408886153846154 | -0.125887225548902 | 1.31410019960080 | -0.270941796407186 |
| 4373 | 0.0440178461538462 | 0.149927624750499 | 0.169744710578842 | 0.179328383233533 |
| 4374 | 0.0252424615384615 | 0.195571896207585 | -1.52039281437126 | 0.342900558882236 |
| 4375 | 0.00688430769230769 | 0.0175429940119760 | -0.231470059880240 | -0.171044191616766 |
| 4376 | 0.0108480000000000 | 0.0752074650698603 | 1.64668612774451 | -0.266718483033932 |
| 4377 | 0.0454781538461538 | 0.160810778443114 | -0.110861976047904 | 0.148628143712575 |
| 4378 | 0.0782307692307692 | 0.389844311377246 | -1.56587465069860 | 0.0854408782435130 |
| 4379 | 0.0767704615384615 | -0.140831257485030 | 0.305784131736527 | -0.201906866267465 |
| 4380 | 0.0183581538461538 | -0.863667584830339 | 1.24750179640719 | -0.118252774451098 |
| 4381 | -0.0329612307692308 | -0.304078562874252 | -0.159186427145709 | 0.0185176047904192 |
| 4382 | -0.0354646153846154 | 0.235693373253493 | -0.837353093812375 | 0.00779688622754491 |
| 4383 | 0.00354646153846154 | 0.263144910179641 | 0.412179141716567 | 0.110293453093812 |
| 4384 | 0.0632104615384615 | 0.150902235528942 | 0.525477644710579 | 0.121826347305389 |
| 4385 | 0.0792738461538462 | -0.137744990019960 | -0.188018662674651 | -0.136445508982036 |
| 4386 | 0.0634190769230769 | 0.0391468662674651 | -0.105176746506986 | -0.0461315768463074 |
| 4387 | 0.0550744615384615 | 0.606045469061876 | 0.0836540918163673 | 0.285073652694611 |
| 4388 | 0.0594553846153846 | -0.0355732934131737 | -0.158780339321357 | 0.0628623952095808 |
| 4389 | 0.0769790769230769 | -1.10488375249501 | -0.0454818363273453 | -0.255023153692615 |
| 4390 | 0.0673827692307692 | -0.100222475049900 | 0.254617065868263 | 0.0295631936127745 |
| 4391 | 0.0208615384615385 | 1.10797001996008 | -0.345986826347305 | 0.223185868263473 |
| 4392 | -0.0289975384615385 | 0.120364431137725 | -0.384971257485030 | -0.0776439920159681 |
| 4393 | -0.0486073846153846 | -0.592725788423154 | 0.278170159680639 | -0.206779920159681 |
| 4394 | -0.0250338461538462 | 0.441661117764471 | 0.423549600798403 | 0.130272974051896 |
| 4395 | 0.0168978461538462 | 0.696359401197605 | -0.312687624750499 | 0.287347744510978 |
| 4396 | 0.0569520000000000 | -0.0834916566866268 | -0.402839121756487 | -0.0245277045908184 |
| 4397 | 0.0488160000000000 | -0.230982754491018 | 0.335428542914172 | -0.0860906187624751 |
| 4398 | 0.0302492307692308 | -0.307164830339321 | 0.296038023952096 | 0.129785668662675 |
| 4399 | 0.0446436923076923 | -0.567061037924152 | -0.179084730538922 | 0.0562025548902196 |

| | | | | |
|---|---|---|---|---|
| 4400 | 0.0740584615384615 | 0.0112080239520958 | -0.148628143712575 | -0.129785668662675 |
| 4401 | 0.100761230769231 | 0.347286307385230 | 0.421113073852295 | -0.0771566866267465 |
| 4402 | 0.0682172307692308 | -0.433376926147705 | 0.148222055888224 | 0.0795932135728543 |
| 4403 | 0.0146030769230769 | -0.146354051896208 | -0.231470059880240 | 0.0639994411177645 |
| 4404 | -0.0358818461538462 | 0.742815848303393 | 0.00690349301397206 | -0.0503548902195609 |
| 4405 | -0.0554916923076923 | 0.100384910179641 | 0.327306786427146 | 0.0297256287425150 |
| 4406 | -0.0127255384615385 | -0.590776566866267 | 0.0722836327345309 | 0.198658163672655 |
| 4407 | 0.0406800000000000 | -0.00747201596806387 | -0.367103393213573 | 0.0417458283433134 |
| 4408 | 0.0786480000000000 | 0.464239600798403 | 0.0791871257485030 | -0.149440319361277 |
| 4409 | 0.0636276923076923 | -0.154963113772455 | 0.241622255489022 | 0.0735831137724551 |
| 4410 | 0.0504849230769231 | -0.333641756487026 | -0.0503548902195609 | 0.122313652694611 |
| 4411 | 0.0713464615384615 | 0.266718483033932 | -0.198170858283433 | -0.229520838323353 |
| 4412 | 0.0815686153846154 | 0.208566706586826 | 0.0653801397205589 | -0.283936606786427 |
| 4413 | 0.0755187692307692 | 0.0168932534930140 | 0.140100299401198 | 0.0630248303393214 |
| 4414 | 0.0219046153846154 | 0.219124990019960 | 0.0194922155688623 | 0.102983872255489 |
| 4415 | -0.0471470769230769 | 0.0773191217564870 | 0.0446696606786427 | -0.215876287425150 |
| 4416 | -0.0671741538461538 | -0.162597564870259 | -0.136851596806387 | -0.129948103792415 |
| 4417 | -0.0344215384615385 | 0.0380098203592814 | -0.0105582834331337 | 0.252424191616766 |
| 4418 | 0.0375507692307692 | -0.177704031936128 | 0.214820459081836 | 0.103146307385230 |
| 4419 | 0.0953372307692308 | -0.401377205588822 | 0.170556886227545 | -0.238454770459082 |
| 4420 | 0.100344000000000 | 0.177541596806387 | -0.249744011976048 | 0.0307002395209581 |
| 4421 | 0.0830289230769231 | 0.279388423153693 | -0.243246606786427 | 0.197845988023952 |
| 4422 | 0.0669655384615385 | -0.238617205588822 | 0.227409181636727 | -0.136607944111776 |
| 4423 | 0.0815686153846154 | -0.228871097804391 | 0.116141117764471 | -0.175429940119760 |
| 4424 | 0.0867840000000000 | 0.192648063872255 | -0.298474550898204 | 0.138069860279441 |
| 4425 | 0.0423489230769231 | 0.164221916167665 | -0.202637824351297 | 0.130272974051896 |
| 4426 | -0.0208615384615385 | -0.133846546906188 | 0.205074351297405 | -0.190373972055888 |
| 4427 | -0.0690516923076923 | -0.0698471057884232 | 0.0747201596806387 | -0.100384910179641 |
| 4428 | -0.0525710769230769 | -0.00893393213572854 | -0.294413672654691 | 0.221886387225549 |
| 4429 | 0.0108480000000000 | -0.151389540918164 | -0.150658582834331 | 0.0953494211576846 |
| 4430 | 0.0784393846153846 | -0.00276139720558882 | 0.271672754491018 | -0.124750179640719 |
| 4431 | 0.107436923076923 | 0.0462940119760479 | 0.000812175648702595 | 0.0417458283433134 |
| 4432 | 0.0945027692307692 | -0.146678922155689 | -0.218069161676647 | 0.123775568862275 |
| 4433 | 0.0755187692307692 | 0.00113704590818363 | 0.158374251497006 | -0.0630248303393214 |
| 4434 | 0.0607070769230769 | 0.184201437125749 | 0.317560678642715 | -0.0605883033932136 |
| 4435 | 0.0408886153846154 | -0.0118577644710579 | -0.0665984031936128 | 0.0560401197604790 |
| 4436 | -0.000208615384615385 | -0.210028622754491 | -0.242840518962076 | -0.0782937325349302 |
| 4437 | -0.0569520000000000 | 0.0550655089820359 | 0.176648203592814 | -0.151876846307385 |
| 4438 | -0.110566153846154 | 0.202556606786427 | 0.0934001996007984 | 0.127674011976048 |
| 4439 | -0.125586461538462 | -0.0739079840319361 | -0.142942914171657 | 0.172018802395210 |
| 4440 | -0.0986750769230769 | -0.0934001996007984 | -0.168120359281437 | -0.117927904191617 |
| 4441 | -0.0427661538461539 | 0.137907425149701 | -0.0828419161676647 | -0.0704968463073852 |
| 4442 | 0.00771876923076923 | 0.114841636726547 | -0.00934001996007984 | 0.200769820359281 |
| 4443 | 0.0289975384615385 | -0.0307002395209581 | 0.0702531936127745 | 0.0932377644710579 |

| | | | | |
|---|---|---|---|---|
| 4444 | 0.0333784615384615 | 0.0917758483033932 | 0.0328931137724551 | -0.157724510978044 |
| 4445 | 0.0358818461538462 | 0.284586347305389 | -0.315530239520958 | -0.0165683832335329 |
| 4446 | 0.0381766153846154 | 0.0935626347305389 | 0.0158374251497006 | 0.151551976047904 |
| 4447 | 0.0135600000000000 | -0.208729141716567 | 0.333398103792415 | -0.0622126546906188 |
| 4448 | -0.0494418461538462 | -0.102983872255489 | -0.0430453093812375 | -0.152851457085828 |
| 4449 | -0.120788307692308 | -0.0604258682634731 | -0.413803493013972 | 0.0350859880239521 |
| 4450 | -0.160425230769231 | -0.0740704191616766 | -0.0186800399201597 | -0.00877149700598802 |
| 4451 | -0.145822153846154 | 0.0854408782435130 | 0.382534730538922 | -0.0644867465069860 |
| 4452 | -0.0680086153846154 | 0.0797556487025948 | -0.0726897205588822 | 0.0802429540918164 |
| 4453 | 0.00730153846153846 | 0.129298363273453 | -0.187206487025948 | 0.0185176047904192 |
| 4454 | 0.0287889230769231 | -0.0519792415169661 | 0.109237624750499 | -0.112567544910180 |
| 4455 | 0.0200270769230769 | -0.320159640718563 | 0.230251796407186 | -0.00357357285429142 |
| 4456 | 0.00250338461538462 | -0.212302714570858 | 0.0194922155688623 | 0.160810778443114 |
| 4457 | 0.00625846153846154 | 0.0488929740518962 | -0.0101521956087824 | 0.0487305389221557 |
| 4458 | 0.00146030769230769 | 0.232444670658683 | 0.0276139720558882 | -0.122638522954092 |
| 4459 | -0.0408886153846154 | 0.0607507385229541 | 0.0101521956087824 | 0.0194922155688623 |
| 4460 | -0.0999267692307692 | -0.139044471057884 | -0.0954306387225549 | 0.0672481437125748 |
| 4461 | -0.167309538461538 | 0.0196546506986028 | -0.0483244510978044 | -0.114191896207585 |
| 4462 | -0.168561230769231 | 0.178028902195609 | -0.0357357285429142 | -0.0399590419161677 |
| 4463 | -0.0938769230769231 | -0.233419281437126 | -0.131978542914172 | 0.104933093812375 |
| 4464 | -0.0200270769230769 | -0.419082634730539 | 0.547000299401198 | -0.0498675848303393 |
| 4465 | 0.0398455384615385 | 0.243977564870259 | -0.0101521956087824 | -0.0510046307385230 |
| 4466 | 0.0694689230769231 | 0.545294730538922 | -1.35511506986028 | 0.215713852295409 |
| 4467 | 0.0717636923076923 | 0.108181796407186 | -0.405681736526946 | 0.136932814371257 |
| 4468 | 0.0598726153846154 | -0.0378473852295409 | 2.21926996007984 | -0.101197085828343 |
| 4469 | 0.0308750769230769 | 0.217175768463074 | 0.0783749500998004 | 0.0485681037924152 |
| 4470 | 0.00292061538461538 | 0.216688463073852 | -2.47591746506986 | 0.216688463073852 |
| 4471 | -0.0515280000000000 | 0.0932377644710579 | 1.12770588822355 | 0.0177054291417166 |
| 4472 | -0.103056000000000 | -0.0393093013972056 | 2.44058782435130 | -0.0519792415169661 |
| 4473 | -0.0940855384615385 | -0.196546506986028 | -1.05826487025948 | 0.130435409181637 |
| 4474 | -0.0283716923076923 | -0.0531162874251497 | -2.08972794411178 | 0.0406087824351297 |
| 4475 | 0.0402627692307692 | 0.420706986027944 | 1.41359171656687 | -0.129948103792415 |
| 4476 | 0.0694689230769231 | 0.291246187624751 | 1.30110538922156 | 0.0121826347305389 |
| 4477 | 0.0842806153846154 | -0.515406666666667 | -0.954306387225549 | 0.145866746506986 |
| 4478 | 0.0809427692307692 | -0.346474131736527 | 0.00934001996007984 | -0.0508421956087824 |
| 4479 | 0.0836547692307692 | 0.394554930139721 | 0.321621556886228 | -0.181115169660679 |
| 4480 | 0.0947113846153846 | 0.0360605988023952 | -0.278170159680639 | 0.0292383233532934 |
| 4481 | 0.0844892307692308 | -0.508097085828343 | 0.437356586826347 | 0.110131017964072 |
| 4482 | 0.0415144615384615 | -0.0341113772455090 | 0.875119261477046 | -0.0817048702594810 |
| 4483 | -0.0323353846153846 | 0.344362475049900 | -1.34536896207585 | -0.100547345309381 |
| 4484 | -0.0863667692307692 | -0.00617253493013972 | -0.970143812375250 | 0.0266393612774451 |
| 4485 | -0.0776049230769231 | -0.125724790419162 | 1.62760000000000 | 0.0206292614770459 |
| 4486 | -0.00375507692307692 | 0.00714714570858283 | 0.464158383233533 | -0.0268017964071856 |
| 4487 | 0.0773963076923077 | -0.200120079840319 | -1.67186357285429 | -0.0319997205588822 |

| | | | | |
|---|---|---|---|---|
| 4488 | 0.124960615384615 | -0.0878774051896208 | -0.652177045908184 | -0.0255023153692615 |
| 4489 | 0.133931076923077 | 0.319185029940120 | 1.64059481037924 | -0.0246901397205589 |
| 4490 | 0.125586461538462 | 0.155775289421158 | 0.434920059880240 | 0.0102334131736527 |
| 4491 | 0.112235076923077 | -0.0596136926147705 | -1.51633193612774 | 0.0279388423153693 |
| 4492 | 0.0761446153846154 | 0.138069860279441 | -0.347205089820359 | -0.0493802794411178 |
| 4493 | 0.0129341538461538 | 0.204505828343313 | 1.08181796407186 | -0.0602634331337325 |
| 4494 | -0.0696775384615385 | -0.117603033932136 | 0.269642315369262 | 0.0688724950099800 |
| 4495 | -0.125586461538462 | -0.161947824351297 | -0.854002694610779 | 0.0760196407185629 |
| 4496 | -0.116198769230769 | 0.0360605988023952 | 0.110861976047904 | -0.0575020359281437 |
| 4497 | -0.0362990769230769 | -0.198333293413174 | 0.565680339321357 | -0.0536035928143713 |
| 4498 | 0.0696775384615385 | -0.348910658682635 | -0.180709081836327 | 0.0643243113772455 |
| 4499 | 0.124752000000000 | 0.0412585229540918 | -0.213602195608782 | 0.0472686227544910 |
| 4500 | 0.113069538461538 | 0.204668263473054 | 0.305378043912176 | -0.0979483832335329 |
| 4501 | 0.0763532307692308 | -0.121501477045908 | 0.107613273453094 | -0.0652989221556886 |
| 4502 | 0.0721809230769231 | -0.0968113373253493 | -0.340707684630739 | 0.0566898602794411 |
| 4503 | 0.0853236923076923 | 0.112729980039920 | 0.224566566866267 | 0.0199795209580838 |
| 4504 | 0.0617501538461538 | -0.0235530938123753 | 0.157968163672655 | -0.0107207185628743 |
| 4505 | 0.00292061538461538 | -0.143592654690619 | -0.258677944111776 | 0.0112080239520958 |
| 4506 | -0.0598726153846154 | 0.0162435129740519 | 0.0852784431137725 | -0.0136445508982036 |
| 4507 | -0.0880356923076923 | 0.159186427145709 | 0.326494610778443 | -0.0107207185628743 |
| 4508 | -0.0506935384615385 | 0.0766693812375250 | -0.198576946107784 | 0.0193297804391218 |
| 4509 | 0.0317095384615385 | 0.143592654690619 | -0.361824251497006 | -0.0108831536926148 |
| 4510 | 0.0949200000000000 | 0.204993133732535 | 0.278170159680639 | -0.0345986826347305 |
| 4511 | 0.112443692307692 | -0.0706592814371258 | 0.268017964071856 | -0.0123450698602794 |
| 4512 | 0.105559384615385 | -0.141156127744511 | -0.290758882235529 | -0.0183551696606786 |
| 4513 | 0.122040000000000 | 0.0761820758483034 | -0.230251796407186 | -0.0287510179640719 |
| 4514 | 0.151037538461538 | -0.0756947704590818 | 0.402433033932136 | 0.0539284630738523 |
| 4515 | 0.175654153846154 | -0.173643153692615 | 0.183551696606786 | 0.0927504590818363 |
| 4516 | 0.171481846153846 | 0.0792683433133733 | -0.431265269461078 | -0.0448320958083832 |
| 4517 | 0.101804307692308 | 0.105095528942116 | -0.370758183632735 | -0.0516543712574850 |
| 4518 | 0.0214873846153846 | -0.00357357285429142 | 0.297662375249501 | 0.146841357285429 |
| 4519 | -0.0267027692307692 | -0.0488929740518962 | 0.235937025948104 | 0.0784561676646707 |
| 4520 | -0.00229476923076923 | -0.00730958083832335 | -0.557964670658683 | -0.172181237524950 |
| 4521 | 0.0467298461538462 | -0.0701719760479042 | -0.312687624750499 | -0.0883647105788423 |
| 4522 | 0.0736412307692308 | -0.154150938123753 | 0.376849500998004 | 0.121501477045908 |
| 4523 | 0.103473230769231 | -0.118740079840319 | 0.182333433133733 | 0.0566898602794411 |
| 4524 | 0.126212307692308 | -0.127998882235529 | -0.443041816367266 | -0.102659001996008 |
| 4525 | 0.154166769230769 | 0.0253398802395210 | -0.185176047904192 | -0.0422331337325349 |
| 4526 | 0.165640615384615 | 0.158861556886228 | 0.224566566866267 | 0.108506666666667 |
| 4527 | 0.141232615384615 | 0.0302129341317365 | 0.0609131736526946 | 0.0869027944111777 |
| 4528 | 0.0621673846153846 | -0.297093852295409 | -0.186800399201597 | -0.0630248303393214 |
| 4529 | -0.0385938461538462 | -0.159348862275449 | -0.0783749500998004 | -0.100222475049900 |
| 4530 | -0.0832375384615385 | 0.681415369261477 | 0.199795209580838 | 0.0224160479041916 |
| 4531 | -0.0486073846153846 | 0.676867185628743 | 0.235124850299401 | 0.107532055888224 |

| | | | | |
|---|---|---|---|---|
| 4532 | 0.0296233846153846 | 0.00909636726546906 | 0.169744710578842 | 0.114841636726547 |
| 4533 | 0.0849064615384615 | -0.0360605988023952 | -0.0381722554890220 | -0.0207916966067864 |
| 4534 | 0.0988836923076923 | -0.0446696606786427 | -0.0328931137724551 | -0.0948621157684631 |
| 4535 | 0.0844892307692308 | -0.0730958083832335 | 0.176242115768463 | 0.170881756487026 |
| 4536 | 0.0592467692307692 | 0.184526307385230 | 0.202637824351297 | 0.244952175648703 |
| 4537 | 0.0425575384615385 | -0.266231177644711 | -0.0735018962075848 | -0.0643243113772455 |
| 4538 | 0.0379680000000000 | -0.831667864271457 | -0.0730958083832335 | -0.0696846706586826 |
| 4539 | -0.0154375384615385 | 0.255348023952096 | 0.188830838323353 | 0.186150658682635 |
| 4540 | -0.107436923076923 | 1.00969676646707 | 0.174617764471058 | 0.0737455489021956 |
| 4541 | -0.169395692307692 | -0.259246467065868 | -0.152689021956088 | -0.169907145708583 |
| 4542 | -0.180243692307692 | -0.667608383233533 | -0.267611876247505 | -0.0555528143712575 |
| 4543 | -0.122874461538462 | 0.460341157684631 | 0.196140419161677 | 0.119552255489022 |
| 4544 | -0.0536141538461538 | 0.337702634730539 | 0.108831536926148 | -0.0695222355289421 |
| 4545 | -0.0216960000000000 | -0.562025548902196 | -0.322433732534930 | -0.178353772455090 |
| 4546 | -0.0360904615384615 | -0.295956806387226 | -0.110861976047904 | 0.116303552894212 |
| 4547 | -0.0611243076923077 | 0.0945372455089820 | 0.262738822355289 | 0.0888520159680639 |
| 4548 | -0.0529883076923077 | -0.152526586826347 | -0.0332992015968064 | -0.241703473053892 |
| 4549 | -0.0312923076923077 | 0.217175768463074 | -0.450351397205589 | -0.0513295009980040 |
| 4550 | -0.0146030769230769 | 0.657537405189621 | -0.0243652694610778 | 0.218312814371257 |
| 4551 | -0.0325440000000000 | -0.0446696606786427 | 0.234718762475050 | -0.0235530938123753 |
| 4552 | -0.0709292307692308 | -0.197845988023952 | -0.105176746506986 | -0.158699121756487 |
| 4553 | -0.0728067692307692 | 0.455630538922156 | -0.207916966067864 | -0.00129948103792415 |
| 4554 | -0.0356732307692308 | 0.141156127744511 | 0.0954306387225549 | 0.00828419161676647 |
| 4555 | 0.00354646153846154 | -0.426067345309381 | 0.287916267465070 | -0.0417458283433134 |
| 4556 | 0.0131427692307692 | -0.0500300199600798 | 0.0881210578842315 | 0.162272694610778 |
| 4557 | -0.00312923076923077 | 0.327631656686627 | -0.0548218562874252 | 0.191673453093812 |
| 4558 | -0.00813600000000000 | -0.0797556487025948 | -0.0288322355289421 | -0.130110538922156 |
| 4559 | 0.00688430769230769 | -0.230982754491018 | 0.218881337325349 | -0.0441823552894212 |
| 4560 | 0.0256596923076923 | 0.124750179640719 | 0.311063273453094 | 0.215713852295409 |
| 4561 | 0.0494418461538462 | 0.0133196806387226 | -0.0259896207584830 | -0.00259896207584830 |
| 4562 | 0.0352560000000000 | -0.290596447105788 | -0.249744011976048 | -0.304078562874252 |
| 4563 | -0.0267027692307692 | -0.179815688622755 | 0.00609131736526946 | -0.112892415169661 |
| 4564 | -0.0725981538461538 | -0.00487305389221557 | 0.361824251497006 | 0.163409740518962 |
| 4565 | -0.0584123076923077 | 0.0100709780439122 | 0.0511670658682635 | -0.0469437524950100 |
| 4566 | 0.00250338461538462 | 0.110455888223553 | -0.245277045908184 | -0.244140000000000 |
| 4567 | 0.0696775384615385 | 0.0825170459081836 | 0.0211165668662675 | 0.0487305389221557 |
| 4568 | 0.123500307692308 | 0.0243652694610778 | 0.188424750499002 | 0.258434291417166 |
| 4569 | 0.134556923076923 | 0.179490818363273 | 0.0227409181636727 | 0.0199795209580838 |
| 4570 | 0.121205538461538 | 0.00714714570858283 | -0.190049101796407 | -0.0391468662674651 |
| 4571 | 0.120371076923077 | -0.229520838323353 | -0.125481137724551 | 0.0885271457085828 |
| 4572 | 0.110983384615385 | 0.0300504990019960 | -0.0791871257485030 | 0.0833292215568862 |
| 4573 | 0.0723895384615385 | 0.193947544910180 | -0.0812175648702595 | -0.0181927345309381 |
| 4574 | 0.00250338461538462 | 0.0219287425149701 | 0.0560401197604790 | -0.0908012375249501 |
| 4575 | -0.0496504615384615 | 0.0779688622754491 | -0.00527914171656687 | 0.00406087824351297 |

| | | | | |
|---|---|---|---|---|
| 4576 | -0.0502763076923077 | 0.0648116167664671 | -0.161216866267465 | 0.0443447904191617 |
| 4577 | -0.0123083076923077 | -0.178678642714571 | 0.473092315369261 | -0.0497051497005988 |
| 4578 | 0.0461040000000000 | -0.0992478642714571 | 0.451163572854291 | -0.104770658682635 |
| 4579 | 0.0636276923076923 | 0.117603033932136 | -1.33359241516966 | 0.0717963273453094 |
| 4580 | 0.0417230769230769 | -0.0930753293413174 | -0.220099600798403 | 0.136932814371257 |
| 4581 | 0.00855323076923077 | -0.274677804391218 | 2.17987944111776 | -0.0882022754491018 |
| 4582 | -0.00897046153846154 | 0.0519792415169661 | -0.461721856287425 | -0.0808926946107784 |
| 4583 | 0.0110566153846154 | 0.0917758483033932 | -2.39469990019960 | 0.155612854291417 |
| 4584 | 0.00604984615384615 | -0.0903139321357285 | 1.59633123752495 | 0.0300504990019960 |
| 4585 | -0.0306664615384615 | 0.148140838323353 | 2.20952385229541 | -0.286860439121756 |
| 4586 | -0.0851150769230769 | 0.234069021956088 | -2.47713572854291 | -0.0661110978043912 |
| 4587 | -0.111817846153846 | -0.192485628742515 | -1.41359171656687 | 0.204505828343313 |
| 4588 | -0.0780221538461538 | -0.219287425149701 | 2.37642594810379 | -0.0633497005988024 |
| 4589 | -0.0415144615384615 | 0.156749900199601 | 0.389438223552894 | -0.203531217564870 |
| 4590 | -0.0227390769230769 | -0.00795932135728543 | -2.01013473053892 | 0.0984356886227545 |
| 4591 | -0.0400541538461538 | -0.198333293413174 | 0.389844311377246 | 0.111430499001996 |
| 4592 | -0.0769790769230769 | 0.0942123752495010 | 1.22313652694611 | -0.0932377644710579 |
| 4593 | -0.0938769230769231 | 0.172830978043912 | -1.20161387225549 | 0.100872215568862 |
| 4594 | -0.0915821538461538 | -0.0402839121756487 | 0.136039421157685 | 0.176079680638723 |
| 4595 | -0.0698861538461539 | -0.0360605988023952 | 1.05136137724551 | -0.105257964071856 |
| 4596 | -0.0684258461538462 | -0.128648622754491 | -1.27064880239521 | -0.128323752495010 |
| 4597 | -0.0897046153846154 | -0.160810778443114 | -0.523041117764471 | 0.108993972055888 |
| 4598 | -0.107854153846154 | -0.0149440319361277 | 1.80140558882236 | 0.0474310578842315 |
| 4599 | -0.100344000000000 | 0.0282637125748503 | 0.255429241516966 | -0.163247305389222 |
| 4600 | -0.0440178461538462 | -0.0206292614770459 | -2.00120079840319 | 0.0498675848303393 |
| 4601 | -0.0160633846153846 | -0.248038443113772 | 0.484056686626747 | 0.271266666666667 |
| 4602 | -0.0252424615384615 | -0.201906866267465 | 2.04018522954092 | -0.0539284630738523 |
| 4603 | -0.0392196923076923 | -0.239429381237525 | -0.624156986027944 | -0.175267504990020 |
| 4604 | -0.0604984615384615 | 0.0399590419161677 | -1.75267504990020 | 0.193135369261477 |
| 4605 | -0.0429747692307692 | 0.867566027944112 | 0.720805888223553 | 0.184851177644711 |
| 4606 | -0.00813600000000000 | 0.800155449101797 | 1.38110469061876 | -0.157074770459082 |
| 4607 | 0.000834461538461538 | 0.209703752495010 | -0.976641217564870 | -0.0963240319361277 |
| 4608 | -0.0383852307692308 | 0.0272891017964072 | -0.708217165668663 | 0.234393892215569 |
| 4609 | -0.103681846153846 | -0.313987105788423 | 0.778470359281437 | 0.103471177644711 |
| 4610 | -0.119745230769231 | -0.550817524950100 | 0.333398103792415 | -0.212627584830339 |
| 4611 | -0.0878270769230769 | 0.248850618762475 | -0.634715269461078 | -0.00129948103792415 |
| 4612 | -0.0344215384615385 | -0.0734206786427146 | -0.0381722554890220 | 0.208566706586826 |
| 4613 | 0.00292061538461538 | -1.06395009980040 | 0.476747105788423 | -0.0877149700598802 |
| 4614 | 0.00104307692307692 | 0.570797045908184 | -0.323245908183633 | -0.155450419161677 |
| 4615 | -0.00688430769230769 | 1.35633333333333 | -0.161216866267465 | 0.114029461077844 |
| 4616 | 0 | -0.660136367265469 | 0.310251097804391 | 0.0562025548902196 |
| 4617 | 0.0369249230769231 | -0.752724391217565 | -0.0950245508982036 | -0.119877125748503 |
| 4618 | 0.0544486153846154 | 0.774490698602794 | -0.244870958083832 | 0.0386595608782435 |
| 4619 | 0.0152289230769231 | 0.0867403592814371 | 0.261114471057884 | 0.162110259481038 |

| | | | | |
|---|---|---|---|---|
| 4620 | -0.0563261538461538 | -1.07174698602794 | 0.181927345309381 | -0.163897045908184 |
| 4621 | -0.106811076923077 | -0.0706592814371258 | -0.268017964071856 | -0.207916966067864 |
| 4622 | -0.100552615384615 | 0.590776566866267 | -0.172993413173653 | 0.149440319361277 |
| 4623 | -0.0621673846153846 | -0.300829860279441 | 0.270860578842315 | 0.0544157684630739 |
| 4624 | -0.0189840000000000 | 0.106882315369261 | 0.0540096806387226 | -0.230333013972056 |
| 4625 | -0.0175236923076923 | 0.801130059880240 | -0.341113772455090 | 0.00276139720558882 |
| 4626 | -0.0463126153846154 | -0.266718483033932 | -0.0219287425149701 | 0.260708383233533 |
| 4627 | -0.0557003076923077 | -0.314799281437126 | 0.438574850299401 | -0.0417458283433134 |
| 4628 | -0.0300406153846154 | 0.753861437125749 | 0.124262874251497 | -0.170556886227545 |
| 4629 | 0.0112652307692308 | 0.0352484231536926 | -0.541721157684631 | 0.136445508982036 |
| 4630 | 0.0123083076923077 | -0.852297125748503 | 0.0389844311377246 | 0.166008702594810 |
| 4631 | -0.0219046153846154 | 0.115653812375249 | 0.600603892215569 | -0.0795932135728543 |
| 4632 | -0.0638363076923077 | 0.649578083832335 | 0.0154313373253493 | -0.0168932534930140 |
| 4633 | -0.0728067692307692 | -0.268667704590818 | -0.526695908183633 | 0.126536966067864 |
| 4634 | -0.0408886153846154 | -0.356707544910180 | 0.159592514970060 | 0.0316748502994012 |
| 4635 | -0.0189840000000000 | 0.350047704590818 | 0.607913473053892 | 0.0651364870259481 |
| 4636 | -0.00876184615384615 | 0.0357357285429142 | -0.177460379241517 | 0.0768318163672655 |
| 4637 | -0.0444350769230769 | -0.254373413173653 | -0.281824950099800 | -0.134821157684631 |
| 4638 | -0.0803169230769231 | 0.100547345309381 | 0.00121826347305389 | -0.0895017564870260 |
| 4639 | -0.0579950769230769 | -0.0406087824351297 | 0.0970549900199601 | 0.211003233532934 |
| 4640 | -0.00688430769230769 | -0.184039001996008 | 0.0300504990019960 | 0.0644867465069860 |
| 4641 | 0.0490246153846154 | 0.0773191217564870 | -0.160404690618762 | -0.313662235528942 |
| 4642 | 0.0486073846153846 | 0.113217285429142 | -0.171775149700599 | -0.0466188822355289 |
| 4643 | 0.0108480000000000 | 0.0847911377245509 | 0.0519792415169661 | 0.355570499001996 |
| 4644 | -0.0227390769230769 | 0.222536127744511 | 0.325682435129741 | -0.0575020359281437 |
| 4645 | -0.0112652307692308 | 0.0110455888223553 | -0.0134008982035928 | -0.400727465069860 |
| 4646 | 0.0498590769230769 | -0.216526027944112 | -0.272484930139721 | 0.0648116167664671 |
| 4647 | 0.0947113846153846 | 0.187125269461078 | -0.0227409181636727 | 0.235693373253493 |
| 4648 | 0.109523076923077 | 0.309926227544910 | 0.197358682634731 | -0.217175768463074 |
| 4649 | 0.0894960000000000 | -0.181277604790419 | 0.0288322355289421 | -0.226597005988024 |
| 4650 | 0.0776049230769231 | -0.238129900199601 | -0.174617764471058 | 0.196221636726547 |
| 4651 | 0.100552615384615 | 0.108831536926148 | -0.0702531936127745 | 0.136607944111776 |
| 4652 | 0.132888000000000 | -0.0243652694610778 | 0.146191616766467 | -0.145379441117764 |
| 4653 | 0.131636307692308 | -0.199632774451098 | 0.217256986027944 | -0.00438574850299401 |
| 4654 | 0.0830289230769231 | 0.140506387225549 | -0.249337924151697 | 0.187937445109780 |
| 4655 | 0.0360904615384615 | 0.0750450299401198 | -0.240810079840319 | -0.00779688622754491 |
| 4656 | 0.0204443076923077 | -0.227084311377246 | 0.382940818363274 | -0.119877125748503 |
| 4657 | 0.0515280000000000 | 0.00211165668662675 | 0.0653801397205589 | 0.0581517764471058 |
| 4658 | 0.106602461538462 | 0.127674011976048 | -0.418676546906188 | 0.0753699001996008 |
| 4659 | 0.139772307692308 | -0.243652694610778 | 0.0337052894211577 | -0.0427204391217565 |
| 4660 | 0.142275692307692 | -0.329743313373253 | 0.372382534930140 | 0.00162435129740519 |
| 4661 | 0.136434461538462 | 0.194272415169661 | -0.105176746506986 | 0.0274515369261477 |
| 4662 | 0.149368615384615 | 0.130272974051896 | -0.310251097804391 | -0.112242674650699 |
| 4663 | 0.188588307692308 | -0.365479041916168 | 0.148628143712575 | -0.0251774451097804 |

| | | | | |
|---|---|---|---|---|
| 4664 | 0.204860307692308 | -0.0812175648702595 | 0.192079540918164 | 0.0680603193612775 |
| 4665 | 0.162928615384615 | 0.260058642714571 | -0.0357357285429142 | -0.114191896207585 |
| 4666 | 0.0965889230769231 | -0.0618877844311377 | -0.105582834331337 | -0.0805678243512974 |
| 4667 | 0.0400541538461538 | -0.115816247504990 | -0.0190861277445110 | 0.184039001996008 |
| 4668 | 0.0308750769230769 | 0.208404271457086 | 0.131978542914172 | 0.120364431137725 |
| 4669 | 0.0661310769230769 | 0.100872215568862 | 0.149846407185629 | -0.163247305389222 |
| 4670 | 0.0922080000000000 | -0.0469437524950100 | -0.0860906187624751 | -0.0505173253493014 |
| 4671 | 0.0959630769230769 | 0.0311875449101796 | -0.185582135728543 | 0.205155568862275 |
| 4672 | 0.0840720000000000 | 0.130597844311377 | 0.168932534930140 | 0.0342738123752495 |
| 4673 | 0.0780221538461538 | 0.0929128942115768 | 0.169338622754491 | -0.237480159680639 |
| 4674 | 0.101387076923077 | 0.0350859880239521 | -0.169338622754491 | -0.0261520558882236 |
| 4675 | 0.104933538461538 | 0.0878774051896208 | -0.260708383233533 | 0.153826067864271 |
| 4676 | 0.0753101538461539 | -0.0589639520958084 | 0.0873088822355290 | -0.0722836327345309 |
| 4677 | 0.0317095384615385 | -0.0256647504990020 | 0.222536127744511 | -0.0669232734530938 |
| 4678 | -0.0248252307692308 | -0.0149440319361277 | -0.135227245508982 | 0.102983872255489 |
| 4679 | -0.0483987692307692 | -0.0825170459081836 | -0.147815968063872 | -0.0216038722554890 |
| 4680 | -0.0287889230769231 | 0.227571616766467 | 0.199795209580838 | -0.155125548902196 |
| 4681 | 0.000208615384615385 | 0.145866746506986 | 0.176242115768463 | 0.0295631936127745 |
| 4682 | 0.00166892307692308 | -0.280525469061876 | -0.0905575848303393 | 0.0838165269461078 |
| 4683 | -0.0164806153846154 | -0.191998323353293 | -0.0990854291417166 | -0.113054850299401 |
| 4684 | -0.0104307692307692 | -0.0295631936127745 | 0.0613192614770459 | -0.0586390818363273 |
| 4685 | 0.00959630769230769 | -0.194434850299401 | -0.00893393213572854 | 0.142780479041916 |
| 4686 | 0.0436006153846154 | -0.0308626746506986 | -0.189643013972056 | 0.0142942914171657 |
| 4687 | 0.0496504615384615 | 0.231794930139721 | -0.0369539920159681 | -0.151714411177645 |
| 4688 | -0.00104307692307692 | 0.215064111776447 | -0.161216866267465 | 0.0706592814371258 |
| 4689 | -0.0492332307692308 | 0.341601077844311 | 0.276951896207585 | 0.0909636726546906 |
| 4690 | -0.0738498461538462 | 0.302941516966068 | 0.303347604790419 | -0.131897325349301 |
| 4691 | -0.0557003076923077 | -0.0768318163672655 | -0.959991616766467 | 0.0609131736526946 |
| 4692 | -0.0237821538461538 | -0.191023712574850 | -0.421519161676647 | 0.334778802395210 |
| 4693 | -0.0108480000000000 | 0.130435409181637 | 1.60120429141717 | -0.00113704590818363 |
| 4694 | -0.0104307692307692 | 0.149765189620759 | 0.246901397205589 | -0.256160199600798 |
| 4695 | -0.0200270769230769 | -0.156425029940120 | -2.05196177644711 | 0.220911776447106 |
| 4696 | -0.0104307692307692 | -0.00292383233532934 | 0.761820758483034 | 0.336403153692615 |
| 4697 | 0.0154375384615385 | 0.0791059081836327 | 1.94800329341317 | -0.209054011976048 |
| 4698 | 0.0312923076923077 | -0.0776439920159681 | -1.12892415169661 | -0.240728862275449 |
| 4699 | 0.00667569230769231 | -0.0258271856287425 | -1.27755229540918 | 0.288322355289421 |
| 4700 | -0.0544486153846154 | -0.258271856287425 | 1.09968582834331 | 0.164546786427146 |
| 4701 | -0.0867840000000000 | -0.646329381237525 | 0.730145908183633 | -0.289296966067864 |
| 4702 | -0.0767704615384615 | -0.0846287025948104 | -1.02943263473054 | -0.00162435129740519 |
| 4703 | -0.0454781538461538 | 0.657862275449102 | 0.0929941117764471 | 0.286210698602794 |
| 4704 | -0.00917907692307692 | 0.110780758483034 | 0.464564471057884 | -0.0641618762475050 |
| 4705 | -0.00709292307692308 | -0.247713572854291 | -0.660704890219561 | -0.213439760479042 |
| 4706 | -0.0150203076923077 | 0.303591257485030 | 0.174211676646707 | 0.141480998003992 |
| 4707 | -0.0146030769230769 | 0.474635449101796 | 0.736237225548902 | 0.171856367265469 |

| | | | | |
|---|---|---|---|---|
| 4708 | 0.00292061538461538 | -0.0211165668662675 | -0.780906886227545 | -0.136445508982036 |
| 4709 | 0.0256596923076923 | -0.177866467065868 | -0.684664071856287 | -0.0690349301397206 |
| 4710 | 0.00584123076923077 | 0.0698471057884232 | 1.10171626746507 | 0.199470339321357 |
| 4711 | -0.0283716923076923 | -0.108019361277445 | 0.389032135728543 | 0.0394717365269461 |
| 4712 | -0.0675913846153846 | 0.115491377245509 | -0.957149001996008 | -0.170881756487026 |
| 4713 | -0.0761446153846154 | 0.509396566866267 | -0.274109281437126 | 0.0297256287425150 |
| 4714 | -0.0509021538461538 | 0.00568522954091816 | 1.06354401197605 | 0.0916134131736527 |
| 4715 | -0.0383852307692308 | -0.355408063872256 | 0.376849500998004 | -0.0386595608782435 |
| 4716 | -0.0356732307692308 | 0.102171696606786 | -0.880398403193613 | -0.0250150099800399 |
| 4717 | -0.0494418461538462 | 0.163084870259481 | -0.0974610778443114 | 0.0219287425149701 |
| 4718 | -0.0511107692307692 | -0.388057524950100 | 0.719181536926148 | 0.00211165668662675 |
| 4719 | -0.0127255384615385 | -0.404301037924152 | 0.142130738522954 | -0.000162435129740519 |
| 4720 | 0.0394283076923077 | -0.0141318562874252 | -0.567304690618762 | 0.0103958483033932 |
| 4721 | 0.0611243076923077 | 0.0365479041916168 | -0.0288322355289421 | -0.0388219960079840 |
| 4722 | 0.0408886153846154 | -0.00698471057884232 | 0.304565868263473 | -0.0375225149700599 |
| 4723 | -0.00542400000000000 | 0.242028343313373 | -0.181521257485030 | 0.00828419161676647 |
| 4724 | -0.0383852307692308 | 0.0503548902195609 | -0.196546506986028 | -0.0110455888223553 |
| 4725 | -0.0173150769230769 | -0.334778802395210 | 0.134008982035928 | -0.0904763672654691 |
| 4726 | 0.0260769230769231 | 0.0157562075848303 | -0.0840601796407186 | -0.0206292614770459 |
| 4727 | 0.0523624615384615 | 0.167470618762475 | -0.257865768463074 | 0.0990854291417166 |
| 4728 | 0.0461040000000000 | -0.154475808383234 | 0.270860578842315 | 0.0216038722554890 |
| 4729 | 0.0106393846153846 | -0.116465988023952 | 0.142942914171657 | -0.0388219960079840 |
| 4730 | 0.00688430769230769 | 0.227409181636727 | -0.195328243512974 | -0.0623750898203593 |
| 4731 | 0.0429747692307692 | 0.154150938123753 | 0.0397966067864271 | -0.0321621556886228 |
| 4732 | 0.0711378461538462 | -0.0997351696606787 | 0.386189520958084 | 0.0729333732534930 |
| 4733 | 0.0805255384615385 | -0.0144567265469062 | 0.144567265469062 | 0.0116953293413174 |
| 4734 | 0.0657138461538462 | -0.0984356886227545 | -0.257459680638723 | -0.0815424351297405 |
| 4735 | 0.0536141538461538 | -0.175429940119760 | 0.0702531936127745 | 0.0562025548902196 |
| 4736 | 0.0636276923076923 | -0.0222536127744511 | 0.205074351297405 | 0.131897325349301 |
| 4737 | 0.0824030769230769 | -0.00730958083832335 | -0.0243652694610778 | -0.103471177644711 |
| 4738 | 0.0869926153846154 | -0.0233906586826347 | -0.219287425149701 | -0.107856926147705 |
| 4739 | 0.0690516923076923 | 0.121339041916168 | 0.124262874251497 | 0.0958367265469062 |
| 4740 | 0.0486073846153846 | 0.161947824351297 | 0.139288123752495 | -0.0157562075848303 |
| 4741 | 0.0348387692307692 | 0.0199795209580838 | -0.259896207584830 | -0.138232295409182 |
| 4742 | 0.0486073846153846 | -0.0472686227544910 | -0.145785528942116 | 0.0451569660678643 |
| 4743 | 0.0890787692307692 | 0.0399590419161677 | 0.0844662674650699 | 0.119714690618762 |
| 4744 | 0.112860923076923 | 0.00795932135728543 | 0.0698471057884232 | -0.116953293413174 |
| 4745 | 0.0824030769230769 | 0.00129948103792415 | -0.371976447105788 | -0.0657862275449102 |
| 4746 | 0.0327526153846154 | 0.125399920159681 | -0.156343812375250 | 0.198333293413174 |
| 4747 | 0.0137686153846154 | -0.0914509780439122 | 0.259896207584830 | 0.0445072255489022 |
| 4748 | 0.0352560000000000 | -0.138882035928144 | 0.0568522954091816 | -0.239104510978044 |
| 4749 | 0.0634190769230769 | 0.151389540918164 | -0.215226546906188 | -0.000487305389221557 |
| 4750 | 0.0609156923076923 | 0.0576644710578842 | -0.0775627744510978 | 0.189074491017964 |
| 4751 | 0.0398455384615385 | -0.275652415169661 | 0.201825648702595 | -0.129948103792415 |

| | | | | |
|---|---|---|---|---|
| 4752 | 0.0118910769230769 | -0.0703344111776447 | 0.0869027944111777 | -0.108993972055888 |
| 4753 | 0.00354646153846154 | 0.235693373253493 | -0.0897454091816367 | 0.191998323353293 |
| 4754 | 0.0337956923076923 | 0.334129061876248 | -0.173399500998004 | 0.0196546506986028 |
| 4755 | 0.0723895384615385 | 0.125399920159681 | 0.0284261477045908 | -0.288159920159681 |
| 4756 | 0.0840720000000000 | -0.680278323353293 | 0.179490818363273 | 0.00665984031936128 |
| 4757 | 0.0577864615384615 | -0.518492934131737 | 0.0678166666666667 | 0.247713572854291 |
| 4758 | 0.0206529230769231 | 0.290271576846307 | -0.183551696606786 | -0.116141117764471 |
| 4759 | 0.0158547692307692 | 0.280525469061876 | -0.166496007984032 | -0.223673173652695 |
| 4760 | 0.0287889230769231 | 0.119714690618762 | 0.156749900199601 | 0.143917524950100 |
| 4761 | 0.0327526153846154 | 0.0373600798403194 | 0.135633333333333 | 0.234881197604790 |
| 4762 | 0.0177323076923077 | -0.183714131736527 | -0.0990854291417166 | -0.114191896207585 |
| 4763 | -0.00917907692307692 | 0.501112375249501 | -0.218881337325349 | -0.151064670658683 |
| 4764 | -0.00354646153846154 | 0.863180279441118 | 0.0491366267465070 | 0.117927904191617 |
| 4765 | 0.0287889230769231 | -0.714389700598803 | 0.326900698602794 | 0.0680603193612775 |
| 4766 | 0.0630018461538462 | -0.981270618762475 | -0.0454818363273453 | -0.00844662674650699 |
| 4767 | 0.0778135384615385 | 0.743952894211577 | -0.319997205588822 | 0.147166227544910 |
| 4768 | 0.0523624615384615 | 0.788460119760479 | 0.128729840319361 | 0.0454818363273453 |
| 4769 | 0.0283716923076923 | -0.520929461077844 | 0.272891017964072 | -0.0800805189620758 |
| 4770 | 0.0327526153846154 | -0.263144910179641 | -0.153095109780439 | 0.210678363273453 |
| 4771 | 0.0504849230769231 | 0.651202435129741 | -0.140506387225549 | 0.228383792415170 |
| 4772 | 0.0684258461538462 | 0.258921596806387 | 0.245683133732535 | -0.186800399201597 |
| 4773 | 0.0575778461538462 | -0.212302714570858 | 0.0645679640718563 | -0.245601916167665 |
| 4774 | 0.0187753846153846 | -0.0646491816367266 | -0.210759580838323 | 0.124912614770459 |
| 4775 | 0.00187753846153846 | -0.412422794411178 | 0.0389844311377246 | 0.116953293413174 |
| 4776 | 0.0191926153846154 | -0.454818363273453 | 0.217663073852295 | -0.254373413173653 |
| 4777 | 0.0565347692307692 | 0.352971536926148 | -0.00284261477045908 | -0.119064950099800 |
| 4778 | 0.0857409230769231 | 0.228708662674651 | -0.126293313373253 | 0.252586626746507 |
| 4779 | 0.0851150769230769 | -0.394392495009980 | 0.0596949101796407 | 0.122476087824351 |
| 4780 | 0.0644621538461539 | 0.0670857085828343 | 0.174617764471058 | -0.0946996806387226 |
| 4781 | 0.0458953846153846 | 0.518655369261477 | 0.118577644710579 | 0.104770658682635 |
| 4782 | 0.0461040000000000 | -0.00909636726546906 | -0.0621314371257485 | 0.185176047904192 |
| 4783 | 0.0567433846153846 | -0.324220518962076 | -0.0320809381237525 | -0.0138069860279441 |
| 4784 | 0.0567433846153846 | 0.0500300199600798 | 0.181521257485030 | -0.0472686227544910 |
| 4785 | 0.0160633846153846 | 0.147328662674651 | 0.180709081836327 | 0.0482432335329341 |
| 4786 | -0.0398455384615385 | -0.160485908183633 | -0.0747201596806387 | 0.0332992015968064 |
| 4787 | -0.0707206153846154 | -0.0945372455089820 | -0.220505688622755 | -0.0419082634730539 |
| 4788 | -0.0492332307692308 | 0.271266666666667 | -0.0198983033932136 | 0.000324870259481038 |
| 4789 | -0.000208615384615385 | 0.179165948103792 | 0.0942123752495010 | 0.0381722554890220 |
| 4790 | 0.0112652307692308 | -0.0667608383233533 | -0.0393905189620759 | 0.0536035928143713 |
| 4791 | 0.00751015384615385 | 0.0391468662674651 | -0.177460379241517 | 0.0484056686626747 |
| 4792 | 0.00417230769230769 | 0.105907704590818 | -0.0828419161676647 | -0.0425580039920160 |
| 4793 | 0.0202356923076923 | -0.103471177644711 | 0.0881210578842315 | -0.0639994411177645 |
| 4794 | 0.0550744615384615 | -0.358331896207585 | 0.0255835329341317 | 0.0976235129740519 |
| 4795 | 0.0613329230769231 | -0.305215608782435 | 0.0625375249500998 | 0.0436950499001996 |

| | | | | |
|---|---|---|---|---|
| 4796 | 0.0104307692307692 | 0.0349235528942116 | -0.138475948103792 | -0.280200598802395 |
| 4797 | -0.0761446153846154 | 0.129460798403194 | 0.0211165668662675 | -0.172668542914172 |
| 4798 | -0.131427692307692 | -0.0890144510978044 | 0.0832480039920160 | 0.176079680638723 |
| 4799 | -0.132679384615385 | -0.176566986027944 | 0.0747201596806387 | 0.0513295009980040 |
| 4800 | -0.0780221538461538 | -0.0451569660678643 | -0.125075049900200 | -0.198820598802395 |
| 4801 | -0.0212787692307692 | 0.103796047904192 | -0.0125887225548902 | 0.0633497005988024 |
| 4802 | -0.00292061538461538 | 0.158699121756487 | 0.684664071856287 | 0.195084590818363 |
| 4803 | -0.00813600000000000 | 0.0906388023952096 | -0.478371457085828 | -0.0289134530938124 |
| 4804 | -0.0160633846153846 | -0.0596136926147705 | -0.936844610778443 | 0.0768318163672655 |
| 4805 | 0.00771876923076923 | -0.0272891017964072 | 0.153095109780439 | 0.168120359281437 |
| 4806 | 0.0388024615384615 | 0.199957644710579 | 1.49968233532934 | -0.176729421157685 |
| 4807 | 0.0423489230769231 | 0.109968582834331 | -0.728927644710579 | -0.294170019960080 |
| 4808 | 0.00771876923076923 | -0.0963240319361277 | -1.46191616766467 | 0.171856367265469 |
| 4809 | -0.0421403076923077 | -0.00990854291417166 | 1.54475808383234 | 0.145541876247505 |
| 4810 | -0.0531969230769231 | 0.0537660279441118 | 0.982326447105788 | -0.363529820359281 |
| 4811 | -0.0219046153846154 | 0.0601009980039920 | -1.06598053892216 | -0.119064950099800 |
| 4812 | 0.0244080000000000 | -0.0402839121756487 | -1.31653672654691 | 0.396504151696607 |
| 4813 | 0.0523624615384615 | -0.114354331337325 | 1.46191616766467 | 0.0994102994011976 |
| 4814 | 0.0402627692307692 | 0.0250150099800399 | 0.472686227544910 | -0.339326986027944 |
| 4815 | 0.0150203076923077 | -0.0401214770459082 | -0.959585528942116 | -0.0123450698602794 |
| 4816 | 0.00250338461538462 | -0.162597564870259 | 0.282637125748503 | 0.215064111776447 |
| 4817 | 0.00897046153846154 | 0.00795932135728543 | 0.0552279441117765 | -0.0776439920159681 |
| 4818 | 0.00229476923076923 | 0.0534411576846307 | 0.0885271457085828 | -0.193785109780439 |
| 4819 | -0.0331698461538462 | -0.116790858283433 | 0.219693512974052 | -0.0612380439121757 |
| 4820 | -0.0723895384615385 | 0.0102334131736527 | 0.566898602794411 | 0.0217663073852295 |
| 4821 | -0.0980492307692308 | 0.299043073852295 | -1.13298502994012 | 0.0575020359281437 |
| 4822 | -0.0698861538461539 | 0.137582554890220 | -0.308626746506986 | 0.0825170459081836 |
| 4823 | -0.0162720000000000 | -0.233906586826347 | 1.35024201596806 | -0.0628623952095808 |
| 4824 | 0.0375507692307692 | -0.0711465868263473 | -0.305784131736527 | -0.0792683433133733 |
| 4825 | 0.0748929230769231 | 0.227409181636727 | -1.32019151696607 | 0.130272974051896 |
| 4826 | 0.0713464615384615 | -0.0761820758483034 | -0.177460379241517 | 0.0925880239520958 |
| 4827 | 0.0769790769230769 | -0.0323245908183633 | 1.39369341317365 | -0.153338762475050 |
| 4828 | 0.0978406153846154 | 0.295469500998004 | -0.429234830339321 | -0.0357357285429142 |
| 4829 | 0.114112615384615 | 0.106719880239521 | -1.11186846307385 | 0.174942634730539 |
| 4830 | 0.0853236923076923 | 0.262982475049900 | 0.348829441117764 | -0.0776439920159681 |
| 4831 | 0.00897046153846154 | -0.122963393213573 | 0.748825948103792 | -0.158861556886228 |
| 4832 | -0.0675913846153846 | -0.895829740518962 | -0.0418270459081836 | 0.128486187624751 |
| 4833 | -0.102430153846154 | -0.435488582834331 | -0.539690718562874 | 0.140019081836327 |
| 4834 | -0.0728067692307692 | 0.561538243512974 | 0.557152495009980 | -0.0423955688622755 |
| 4835 | -0.0191926153846154 | 0.423468383233533 | 0.114922854291417 | 0.104933093812375 |
| 4836 | 0.0160633846153846 | -0.482757205588822 | -0.196952594810379 | 0.279063552894212 |
| 4837 | 0.0158547692307692 | -0.0131572455089820 | 0.0430453093812375 | 0.0329743313373254 |
| 4838 | -0.000208615384615385 | 0.630735608782435 | 0.0402026946107784 | -0.105745269461078 |
| 4839 | 0.00250338461538462 | -0.00438574850299401 | -0.0511670658682635 | 0.154963113772455 |

| | | | | |
|---|---|---|---|---|
| 4840 | 0.0164806153846154 | -0.691161477045908 | -0.0617253493013972 | 0.121501477045908 |
| 4841 | 0.00521538461538462 | -0.421844031936128 | 0.379286027944112 | -0.241216167664671 |
| 4842 | -0.0267027692307692 | 0.498188542914172 | -0.203856087824351 | -0.111268063872255 |
| 4843 | -0.0703033846153846 | 0.673618483033932 | -0.0674105788423154 | 0.190373972055888 |
| 4844 | -0.0919993846153846 | 0.0536035928143713 | 0.278170159680639 | -0.0540908982035928 |
| 4845 | -0.0803169230769231 | -0.0740704191616766 | 0.186394311377246 | -0.275165109780439 |
| 4846 | -0.0461040000000000 | 0.286048263473054 | -0.358575548902196 | 0.118577644710579 |
| 4847 | -0.0120996923076923 | 0.433376926147705 | -0.331773752495010 | 0.296444111776447 |
| 4848 | -0.0252424615384615 | 0.102821437125749 | 0.325682435129741 | -0.140668822355289 |
| 4849 | -0.0419316923076923 | -0.476909540918164 | 0.00121826347305389 | -0.202719041916168 |
| 4850 | -0.0496504615384615 | -0.416321237524950 | -0.357357285429142 | 0.165521397205589 |
| 4851 | -0.0431833846153846 | 0.216688463073852 | -0.110455888223553 | 0.108344231536926 |
| 4852 | -0.0458953846153846 | 0.397316327345309 | 0.594106487025948 | -0.236505548902196 |
| 4853 | -0.0826116923076923 | -0.255672894211577 | 0.0787810379241517 | -0.0969737724550898 |
| 4854 | -0.129967384615385 | -0.538797325349302 | -0.350859880239521 | 0.287510179640719 |
| 4855 | -0.176280000000000 | 0.198495728542914 | 0.0471061876247505 | 0.143755089820359 |
| 4856 | -0.153332307692308 | 0.361580598802395 | 0.424767864271457 | -0.226597005988024 |
| 4857 | -0.0844892307692308 | -0.378148982035928 | 0.0848723552894212 | -0.0100709780439122 |
| 4858 | 0.000417230769230769 | -0.514107185628743 | -0.395123453093812 | 0.249662794411178 |
| 4859 | 0.0573692307692308 | 0.0706592814371258 | 0.0125887225548902 | -0.0782937325349302 |
| 4860 | 0.0486073846153846 | 0.277601636726547 | 0.0714714570858283 | -0.125887225548902 |
| 4861 | 0.0521538461538462 | -0.0529538522954092 | 0.129135928143713 | 0.148140838323353 |
| 4862 | 0.0567433846153846 | -0.103796047904192 | -0.140912475049900 | -0.0204668263473054 |
| 4863 | 0.0458953846153846 | 0.0331367664670659 | -0.131166367265469 | -0.228871097804391 |
| 4864 | 0.0185667692307692 | -0.00747201596806387 | 0.100709780439122 | 0.0443447904191617 |
| 4865 | -0.0463126153846154 | 0.122638522954092 | 0.227003093812375 | 0.232444670658683 |
| 4866 | -0.103264615384615 | 0.172343672654691 | 0.0694410179640719 | -0.102821437125749 |
| 4867 | -0.118910769230769 | 0.115166506986028 | -0.319591117764471 | -0.235043632734531 |
| 4868 | -0.0832375384615385 | 0.112080239520958 | 0.0844662674650699 | 0.164221916167665 |
| 4869 | -0.0143944615384615 | 0.0357357285429142 | 0.183551696606786 | 0.206942355289421 |
| 4870 | 0.0415144615384615 | -0.0485681037924152 | -0.0438574850299401 | -0.192160758483034 |
| 4871 | 0.0659224615384615 | 0.00422331337325349 | -0.352078143712575 | -0.123613133732535 |
| 4872 | 0.0709292307692308 | 0.136445508982036 | -0.0747201596806387 | 0.225297524950100 |
| 4873 | 0.0942941538461538 | -0.0331367664670659 | 0.250556187624750 | 0.0514919361277445 |
| 4874 | 0.113069538461538 | -0.279550858283433 | 0.103552395209581 | -0.235693373253493 |
| 4875 | 0.104933538461538 | -0.183714131736527 | -0.0637557884231537 | 0.0289134530938124 |
| 4876 | 0.0521538461538462 | 0.122638522954092 | -0.0381722554890220 | 0.197683552894212 |
| 4877 | -0.0317095384615385 | 0.127024271457086 | 0.380910379241517 | -0.117440598802395 |
| 4878 | -0.0690516923076923 | -0.0942123752495010 | 0.205074351297405 | -0.201257125748503 |
| 4879 | -0.0611243076923077 | -0.0623750898203593 | -0.195328243512974 | 0.0724460678642715 |
| 4880 | -0.00813600000000000 | -0.0222536127744511 | -0.290352794411178 | 0.156587465069860 |
| 4881 | 0.0517366153846154 | -0.0415833932135729 | 0.190049101796407 | -0.0713090219560878 |
| 4882 | 0.0711378461538462 | -0.0159186427145709 | 0.197358682634731 | -0.155775289421158 |
| 4883 | 0.0748929230769231 | -0.0849535728542914 | -0.340301596806387 | 0.0963240319361277 |

| | | | | |
|---|---|---|---|---|
| 4884 | 0.0734326153846154 | 0.00747201596806387 | -0.114922854291417 | 0.129785668662675 |
| 4885 | 0.0815686153846154 | 0.277439201596806 | 0.271266666666667 | -0.147491097804391 |
| 4886 | 0.0867840000000000 | 0.242515648702595 | 0.155531636726547 | -0.108993972055888 |
| 4887 | 0.0471470769230769 | -0.0326494610778443 | -0.335834630738523 | 0.0856033133732535 |
| 4888 | -0.0294147692307692 | -0.0601009980039920 | -0.153907285429142 | -0.0383346906187625 |
| 4889 | -0.105976615384615 | 0.0885271457085828 | 0.118171556886228 | -0.149277884231537 |
| 4890 | -0.132470769230769 | 0.00633497005988024 | -0.0576644710578842 | 0.0362230339321357 |
| 4891 | -0.0888701538461539 | -0.133196806387226 | -0.194516067864271 | 0.187775009980040 |
| 4892 | -0.0231563076923077 | 0.00584766467065868 | -0.177054291417166 | -0.0129948103792415 |
| 4893 | 0.0277458461538462 | 0.119389820359281 | 0.118171556886228 | -0.216363592814371 |
| 4894 | 0.0496504615384615 | -0.0227409181636727 | -0.0397966067864271 | 0.0765069461077844 |
| 4895 | 0.0573692307692308 | -0.0560401197604790 | -0.123450698602794 | 0.292383233532934 |
| 4896 | 0.0650880000000000 | 0.109318842315369 | -0.000812175648702595 | -0.0410960878243513 |
| 4897 | 0.0748929230769231 | 0.0310251097804391 | 0.0990854291417166 | -0.179328383233533 |
| 4898 | 0.0673827692307692 | -0.204993133732535 | 0.145379441117764 | 0.107207185628743 |
| 4899 | 0.00813600000000000 | -0.202881477045908 | -0.0182739520958084 | 0.170232015968064 |
| 4900 | -0.0732240000000000 | -0.192972934131737 | -0.0397966067864271 | -0.0700095409181637 |
| 4901 | -0.113695384615385 | -0.107044750499002 | -0.0462940119760479 | -0.0880398403193613 |
| 4902 | -0.0757273846153846 | 0.212465149700599 | 0.117359381237525 | 0.119227385229541 |
| 4903 | -0.00604984615384615 | 0.231307624750499 | -0.0300504990019960 | 0.0752074650698603 |
| 4904 | 0.0544486153846154 | 0.0319997205588822 | -0.0917758483033932 | -0.0885271457085828 |
| 4905 | 0.0723895384615385 | 0.0756947704590818 | 0.0316748502994012 | 0.0258271856287425 |
| 4906 | 0.0490246153846154 | 0.0563649900199601 | 0.0730958083832335 | 0.0833292215568862 |
| 4907 | 0.0444350769230769 | 0.137257684630739 | 0.145379441117764 | -0.0601009980039920 |
| 4908 | 0.0446436923076923 | 0.213439760479042 | 0.0150252495009980 | -0.00860906187624751 |
| 4909 | 0.0486073846153846 | 0.0932377644710579 | 0.0548218562874252 | 0.0982732534930140 |
| 4910 | 0.0246166153846154 | 0.180140558882236 | 0.0852784431137725 | -0.00860906187624751 |
| 4911 | -0.0509021538461538 | -0.0539284630738523 | 0.0353296407185629 | -0.151389540918164 |
| 4912 | -0.0924166153846154 | -0.134658722554890 | -0.0730958083832335 | -0.0417458283433134 |
| 4913 | -0.0686344615384615 | 0.118577644710579 | 0.0113704590818363 | 0.120851736526946 |
| 4914 | -0.00229476923076923 | -0.288159920159681 | -0.131978542914172 | 0.00113704590818363 |
| 4915 | 0.0846978461538462 | -0.753861437125749 | 0.189236926147705 | -0.146354051896208 |
| 4916 | 0.127046769230769 | -0.176079680638723 | 0.512888922155689 | -0.0539284630738523 |
| 4917 | 0.114321230769231 | 0.575020359281437 | -0.718369361277445 | 0.126861836327345 |
| 4918 | 0.0959630769230769 | 0.142455608782435 | -0.998569960079840 | 0.170719321357285 |
| 4919 | 0.0880356923076923 | -0.269642315369262 | 1.37582554890220 | -0.0159186427145709 |
| 4920 | 0.0959630769230769 | 0.136607944111776 | 1.22841566866267 | -0.106719880239521 |
| 4921 | 0.0851150769230769 | 0.327469221556886 | -2.12911846307385 | 0.157724510978044 |
| 4922 | 0.0267027692307692 | 0.0224160479041916 | -0.526695908183633 | 0.188587185628743 |
| 4923 | -0.0296233846153846 | -0.277276766467066 | 2.38982684630739 | -0.0895017564870260 |
| 4924 | -0.0521538461538462 | -0.305702914171657 | -0.210759580838323 | -0.107532055888224 |
| 4925 | -0.0204443076923077 | -0.0407712175648703 | -2.37236506986028 | 0.0469437524950100 |
| 4926 | 0.0442264615384615 | 0.400727465069860 | 0.535223752495010 | 0.00909636726546906 |
| 4927 | 0.0786480000000000 | 0.378473852295409 | 1.79044121756487 | -0.141805868263473 |

| | | | | |
|---|---|---|---|---|
| 4928 | 0.0909563076923077 | 0.0173805588822355 | -0.933595908183633 | -0.0211165668662675 |
| 4929 | 0.0894960000000000 | 0.0495427145708583 | -0.623750898203593 | 0.143592654690619 |
| 4930 | 0.0857409230769231 | 0.210353493013972 | 0.986793413173653 | -0.0474310578842315 |
| 4931 | 0.104933538461538 | 0.00682227544910180 | -0.0686288423153693 | -0.122638522954092 |
| 4932 | 0.120788307692308 | -0.284099041916168 | -0.325276347305389 | 0.207916966067864 |
| 4933 | 0.0809427692307692 | -0.188099880239521 | 0.927504590818363 | 0.164546786427146 |
| 4934 | 0.00271200000000000 | 0.0282637125748503 | -0.206292614770459 | -0.196384071856287 |
| 4935 | -0.0607070769230769 | -0.0654613572854292 | -1.17237554890220 | -0.0633497005988024 |
| 4936 | -0.0607070769230769 | -0.176242115768463 | 0.939687225548902 | 0.177216726546906 |
| 4937 | 0.00354646153846154 | 0.110293453093812 | 1.02699610778443 | -0.00909636726546906 |
| 4938 | 0.0548658461538462 | 0.271753972055888 | -1.22110608782435 | -0.174455329341317 |
| 4939 | 0.0755187692307692 | -0.207754530938124 | -0.851160079840319 | 0.0324870259481038 |
| 4940 | 0.0607070769230769 | -0.325195129740519 | 1.47003792415170 | 0.0948621157684631 |
| 4941 | 0.0398455384615385 | 0.224485349301397 | 0.900702794411178 | -0.0813800000000000 |
| 4942 | 0.0469384615384615 | 0.232931976047904 | -1.48993622754491 | -0.0389844311377246 |
| 4943 | 0.0458953846153846 | -0.0635121357285429 | -0.559182934131737 | 0.150739800399202 |
| 4944 | 0.0296233846153846 | 0.139694211576846 | 1.35064810379242 | 0.0451569660678643 |
| 4945 | -0.0250338461538462 | 0.210028622754491 | 0.248931836327345 | -0.159348862275449 |
| 4946 | -0.0886615384615385 | -0.00860906187624751 | -1.16628423153693 | 0.0279388423153693 |
| 4947 | -0.0880356923076923 | 0.0445072255489022 | -0.204262175648703 | 0.133359241516966 |
| 4948 | -0.0417230769230769 | -0.0326494610778443 | 0.768318163672655 | -0.137744990019960 |
| 4949 | 0.0168978461538462 | -0.290758882235529 | -0.196140419161677 | -0.130272974051896 |
| 4950 | 0.0546572307692308 | -0.0735831137724551 | -0.529944610778443 | 0.162272694610778 |
| 4951 | 0.0538227692307692 | 0.236343113772455 | 0.249744011976048 | 0.00714714570858283 |
| 4952 | 0.0398455384615385 | 0.110780758483034 | 0.196546506986028 | -0.177704031936128 |
| 4953 | 0.0262855384615385 | -0.0665984031936128 | -0.256647504990020 | 0.125562355289421 |
| 4954 | 0.0231563076923077 | -0.00958367265469062 | 0.0113704590818363 | 0.238454770459082 |
| 4955 | 0.0156461538461538 | -0.0212790019960080 | 0.144161177644711 | -0.0458067065868264 |
| 4956 | -0.0325440000000000 | -0.124425309381238 | -0.385783433133733 | -0.103146307385230 |
| 4957 | -0.0926252307692308 | -0.135470898203593 | -0.0580705588822355 | 0.105582834331337 |
| 4958 | -0.112235076923077 | -0.167308183632735 | 0.364260778443114 | 0.0268017964071856 |
| 4959 | -0.0899132307692308 | -0.136120638722555 | -0.0601009980039920 | -0.210840798403194 |
| 4960 | -0.0362990769230769 | 0.0165683832335329 | -0.398778243512974 | -0.125399920159681 |
| 4961 | 0.0106393846153846 | 0.172018802395210 | 0.0714714570858283 | 0.0295631936127745 |
| 4962 | 0.0225304615384615 | 0.221723952095808 | 0.511670658682635 | -0.0360605988023952 |
| 4963 | 0.0191926153846154 | 0.172343672654691 | -0.105176746506986 | 0.0362230339321357 |
| 4964 | 0.0258683076923077 | -0.0269642315369261 | -0.294413672654691 | 0.123775568862275 |
| 4965 | 0.0481901538461539 | -0.257459680638723 | 0.320403293413174 | -0.0652989221556886 |
| 4966 | 0.0688430769230769 | -0.0791059081836327 | 0.323245908183633 | -0.0981108183632735 |
| 4967 | 0.0554916923076923 | 0.115166506986028 | -0.348829441117764 | 0.200607385229541 |
| 4968 | -0.000625846153846154 | -0.118090339321357 | -0.317966766467066 | 0.166008702594810 |
| 4969 | -0.0559089230769231 | -0.106070139720559 | 0.347611177644711 | -0.193297804391218 |
| 4970 | -0.0698861538461539 | 0.194434850299401 | 0.159592514970060 | -0.0677354491017964 |
| 4971 | -0.0212787692307692 | 0.162435129740519 | -0.443447904191617 | 0.205480439121756 |

| | | | | |
|---|---|---|---|---|
| 4972 | 0.0325440000000000 | -0.000324870259481038 | -0.165277744510978 | 0.0206292614770459 |
| 4973 | 0.0573692307692308 | -0.0458067065868264 | 0.466188822355289 | -0.192160758483034 |
| 4974 | 0.0634190769230769 | -0.114354331337325 | 0.0950245508982036 | 0.0617253493013972 |
| 4975 | 0.0410972307692308 | -0.144242395209581 | -0.422737425149701 | 0.214576806387226 |
| 4976 | 0.0327526153846154 | 0.0562025548902196 | -0.0361418163672655 | -0.0295631936127745 |
| 4977 | 0.0306664615384615 | 0.0462940119760479 | 0.270860578842315 | -0.0266393612774451 |
| 4978 | 0.00479815384615385 | -0.269642315369262 | 0.00324870259481038 | 0.101359520958084 |
| 4979 | -0.0529883076923077 | -0.165034091816367 | -0.251774451097804 | -0.0703344111776447 |
| 4980 | -0.117450461538462 | -0.227896487025948 | -0.0601009980039920 | -0.202719041916168 |
| 4981 | -0.136851692307692 | -0.0648116167664671 | 0.123044610778443 | 0.0344362475049900 |
| 4982 | -0.0905390769230769 | 0.970387465069860 | 0.148222055888224 | 0.0217663073852295 |
| 4983 | -0.0235735384615385 | 0.958854570858284 | 0.142536826347305 | -0.197358682634731 |
| 4984 | 0.000417230769230769 | 0.132547065868263 | -0.0276139720558882 | -0.0313499800399202 |
| 4985 | -0.0198184615384615 | -0.244952175648703 | -0.0917758483033932 | 0.130922714570858 |
| 4986 | -0.0569520000000000 | -0.274190499001996 | 0.0925880239520958 | -0.00438574850299401 |
| 4987 | -0.0696775384615385 | -0.0875525349301397 | 0.152282934131737 | -0.0610756087824351 |
| 4988 | -0.0452695384615385 | -0.0107207185628743 | -0.149034231536926 | 0.136932814371257 |
| 4989 | -0.0269113846153846 | -0.397153892215569 | -0.197358682634731 | 0.0540908982035928 |
| 4990 | -0.0486073846153846 | -0.745577245508982 | 0.0353296407185629 | -0.0717963273453094 |
| 4991 | -0.0997181538461539 | 0.478209021956088 | 0.179084730538922 | 0.0648116167664671 |
| 4992 | -0.139355076923077 | 1.18528914171657 | -0.0751262475049900 | 0.0259896207584830 |
| 4993 | -0.118702153846154 | -0.432402315369262 | -0.305784131736527 | -0.142618043912176 |
| 4994 | -0.0713464615384615 | -0.894692694610778 | 0.294819760479042 | -0.0513295009980040 |
| 4995 | -0.0248252307692308 | 0.436138323353293 | 0.331367664670659 | 0.181115169660679 |
| 4996 | -0.000625846153846154 | 0.345337085828343 | -0.265581437125749 | 0.0211165668662675 |
| 4997 | -0.00709292307692308 | -0.676867185628743 | -0.0921819361277445 | -0.0981108183632735 |
| 4998 | -0.00208615384615385 | -0.508909261477046 | 0.445072255489022 | 0.118415209580838 |
| 4999 | 0.00855323076923077 | 0.136445508982036 | 0.0483244510978044 | 0.141643433133733 |
| 5000 | 0.0225304615384615 | 0.199307904191617 | -0.493396706586826 | -0.0961615968063872 |
| 5001 | 0.00688430769230769 | 0.264281956087824 | 0.0560401197604790 | -0.120689301397206 |
| 5002 | -0.0346301538461538 | 0.437437804391218 | 0.324058083832335 | 0.114029461077844 |
| 5003 | -0.0579950769230769 | -0.0641618762475050 | -0.0507609780439122 | 0.0575020359281437 |
| 5004 | -0.0542400000000000 | -0.0557152495009980 | -0.255835329341317 | -0.129460798403194 |
| 5005 | -0.00312923076923077 | 0.574045748502994 | 0.146597704590818 | -0.0266393612774451 |
| 5006 | 0.0515280000000000 | 0.0795932135728543 | 0.278982335329341 | 0.152689021956088 |
| 5007 | 0.0794824615384615 | -0.656400359281437 | 0.0227409181636727 | 0.0570147305389222 |
| 5008 | 0.0824030769230769 | -0.0529538522954092 | -0.0438574850299401 | -0.0661110978043912 |
| 5009 | 0.0648793846153846 | 0.445234690618763 | -0.157155988023952 | 0.000974610778443114 |
| 5010 | 0.0794824615384615 | -0.227409181636727 | -0.0166496007984032 | 0.0246901397205589 |
| 5011 | 0.107019692307692 | -0.323245908183633 | 0.161216866267465 | 0.0232282235528942 |
| 5012 | 0.113069538461538 | 0.235206067864271 | 0.0203043912175649 | 0.00552279441117765 |
| 5013 | 0.0759360000000000 | 0.147815968063872 | -0.356139021956088 | -0.0458067065868264 |
| 5014 | 0.0173150769230769 | -0.191348582834331 | -0.150252495009980 | -0.0394717365269461 |
| 5015 | 0.0108480000000000 | -0.0807302594810379 | 0.377661676646707 | 0.0860906187624751 |

| | | | | |
|---|---|---|---|---|
| 5016 | 0.0527796923076923 | 0.0820297405189621 | 0.241622255489022 | 0.114029461077844 |
| 5017 | 0.114529846153846 | -0.0157562075848303 | -0.259490119760479 | -0.0259896207584830 |
| 5018 | 0.155418461538462 | -0.0714714570858283 | -0.0791871257485030 | -0.101034650698603 |
| 5019 | 0.153749538461538 | 0.0729333732534930 | 0.322839820359281 | 0.00893393213572854 |
| 5020 | 0.128715692307692 | 0.195734331337325 | 0.103146307385230 | 0.179978123752495 |
| 5021 | 0.103890461538462 | 0.207104790419162 | -0.283449301397206 | 0.0578269061876247 |
| 5022 | 0.128507076923077 | 0.0651364870259481 | -0.144973353293413 | -0.151064670658683 |
| 5023 | 0.159590769230769 | -0.181440039920160 | 0.0844662674650699 | -0.0497051497005988 |
| 5024 | 0.135182769230769 | -0.0406087824351297 | -0.138069860279441 | 0.147978403193613 |
| 5025 | 0.0784393846153846 | 0.261033253493014 | -0.0462940119760479 | 0.0638370059880239 |
| 5026 | 0.0162720000000000 | 0.0417458283433134 | 0.0507609780439122 | -0.168282794411178 |
| 5027 | 0.0100135384615385 | -0.362392774451098 | -0.0284261477045908 | -0.127836447105788 |
| 5028 | 0.0509021538461538 | -0.186637964071856 | 0.339895508982036 | 0.116141117764471 |
| 5029 | 0.0878270769230769 | 0.0149440319361277 | 0.404463473053892 | 0.0477559281437126 |
| 5030 | 0.0863667692307692 | -0.122963393213573 | -1.12202065868263 | -0.143917524950100 |
| 5031 | 0.0481901538461539 | 0.00828419161676647 | -0.537254191616767 | 0.0934001996007984 |
| 5032 | 0.0256596923076923 | 0.0735831137724551 | 2.00241906187625 | 0.218312814371257 |
| 5033 | 0.0285803076923077 | -0.179653253493014 | -0.0324870259481038 | -0.0851160079840319 |
| 5034 | 0.0492332307692308 | -0.0575020359281437 | -2.47916616766467 | -0.119389820359281 |
| 5035 | 0.0400541538461538 | 0.223510738522954 | 0.860906187624751 | 0.169744710578842 |
| 5036 | 0.00792738461538461 | -0.0529538522954092 | 2.56606896207585 | 0.0596136926147705 |
| 5037 | -0.0342129230769231 | -0.241053732534930 | -1.63694001996008 | -0.245764351297405 |
| 5038 | -0.0575778461538462 | 0.169257405189621 | -1.90495798403194 | -0.00211165668662675 |
| 5039 | 0.0102221538461538 | 0.274352934131737 | 1.86881616766467 | 0.346799001996008 |
| 5040 | 0.0863667692307692 | -0.212627584830339 | 1.00506736526946 | 0.0477559281437126 |
| 5041 | 0.108062769230769 | -0.0805678243512974 | -1.37298293413174 | -0.337702634730539 |
| 5042 | 0.0886615384615385 | 0.281500079840319 | -0.0523853293413174 | -0.0172181237524950 |
| 5043 | 0.0488160000000000 | -0.140831257485030 | 0.761008582834331 | 0.264769261477046 |
| 5044 | 0.0406800000000000 | -0.134171417165669 | -0.853596606786427 | -0.100872215568862 |
| 5045 | 0.0536141538461538 | 0.362555209580838 | 0.236343113772455 | -0.260708383233533 |
| 5046 | 0.0817772307692308 | 0.110131017964072 | 1.01197085828343 | 0.0604258682634731 |
| 5047 | 0.0890787692307692 | -0.433539361277445 | -1.26861836327345 | 0.0948621157684631 |
| 5048 | 0.0123083076923077 | -0.131734890219561 | -0.851160079840319 | -0.141480998003992 |
| 5049 | -0.0700947692307692 | 0.281337644710579 | 1.63003652694611 | -0.0240403992015968 |
| 5050 | -0.0865753846153846 | -0.0890144510978044 | 0.647303992015968 | 0.144404830339321 |
| 5051 | -0.0492332307692308 | -0.0966489021956088 | -1.77135508982036 | -0.0495427145708583 |
| 5052 | -0.0114738461538462 | 0.0743952894211577 | -0.318372854291417 | -0.150577365269461 |
| 5053 | -0.0198184615384615 | -0.165521397205589 | 1.67226966067864 | 0.0406087824351297 |
| 5054 | -0.0241993846153846 | -0.144079960079840 | 0.116141117764471 | 0.0636745708582834 |
| 5055 | -0.0104307692307692 | 0.0168932534930140 | -1.48100229540918 | -0.0864154890219561 |
| 5056 | 0.0231563076923077 | 0.0534411576846307 | -0.0617253493013972 | -0.0255023153692615 |
| 5057 | 0.0723895384615385 | 0.368565309381238 | 1.14394940119760 | 0.103633612774451 |
| 5058 | 0.0719723076923077 | 0.512970139720559 | -0.179896906187625 | 0.0605883033932136 |
| 5059 | 0.0191926153846154 | 0.0836540918163673 | -0.611974351297405 | -0.00129948103792415 |

| | | | | |
|---|---|---|---|---|
| 5060 | -0.0477729230769231 | -0.207429660678643 | 0.389438223552894 | 0.0165683832335329 |
| 5061 | -0.0736412307692308 | -0.514594491017964 | 0.359387724550898 | -0.0256647504990020 |
| 5062 | -0.0433920000000000 | -0.164384351297405 | -0.321215469061876 | -0.0458067065868264 |
| 5063 | 0.00959630769230769 | 0.340788902195609 | -0.0718775449101796 | 0.0263144910179641 |
| 5064 | 0.0623760000000000 | -0.295144630738523 | 0.443447904191617 | 0.0279388423153693 |
| 5065 | 0.0596640000000000 | -0.265094131736527 | -0.129948103792415 | -0.0425580039920160 |
| 5066 | 0.0377593846153846 | 0.707892295409182 | -0.323651996007984 | -0.0375225149700599 |
| 5067 | 0.0502763076923077 | 0.476259800399202 | 0.194516067864271 | 0.0394717365269461 |
| 5068 | 0.0711378461538462 | -0.428991177644711 | 0.118983732534930 | 0.0232282235528942 |
| 5069 | 0.0886615384615385 | -0.217338203592814 | -0.170150798403194 | -0.00617253493013972 |
| 5070 | 0.0559089230769231 | 0.205805309381238 | 0.0592888223552894 | -0.0178678642714571 |
| 5071 | -0.000417230769230769 | -0.124750179640719 | 0.274109281437126 | -0.0599385628742515 |
| 5072 | -0.0379680000000000 | -0.285885828343313 | -0.123450698602794 | 0.0230657884231537 |
| 5073 | -0.0392196923076923 | 0.105745269461078 | -0.157155988023952 | 0.129135928143713 |
| 5074 | 0.0125169230769231 | 0.121663912175649 | 0.231063972055888 | 0.0180302994011976 |
| 5075 | 0.0557003076923077 | 0.0107207185628743 | 0.128729840319361 | -0.144242395209581 |
| 5076 | 0.0659224615384615 | 0.382372295409182 | -0.272891017964072 | -0.0430453093812375 |
| 5077 | 0.0513193846153846 | 0.0451569660678643 | -0.167308183632735 | 0.142618043912176 |
| 5078 | 0.0429747692307692 | -0.525802514970060 | 0.391062574850299 | 0.0196546506986028 |
| 5079 | 0.0519452307692308 | 0.236830419161677 | 0.202637824351297 | -0.203856087824351 |
| 5080 | 0.0588295384615385 | 0.602959201596806 | -0.436138323353293 | -0.0334616367265469 |
| 5081 | 0.0509021538461538 | -0.212627584830339 | -0.198576946107784 | 0.237317724550898 |
| 5082 | 0.0123083076923077 | -0.240566427145709 | 0.471061876247505 | 0.0461315768463074 |
| 5083 | -0.0131427692307692 | 0.404301037924152 | 0.308626746506986 | -0.205155568862275 |
| 5084 | -0.0146030769230769 | 0.349560399201597 | -0.449133133732535 | -0.00860906187624751 |
| 5085 | 0.00938769230769231 | -0.249987664670659 | -0.248119660678643 | 0.147815968063872 |
| 5086 | 0.0344215384615385 | -0.207267225548902 | 0.389438223552894 | -0.0196546506986028 |
| 5087 | 0.0139772307692308 | 0.154313373253493 | 0.141724650698603 | -0.0906388023952096 |
| 5088 | -0.0191926153846154 | -0.00601009980039920 | -0.322433732534930 | 0.0401214770459082 |
| 5089 | -0.0415144615384615 | -0.0352484231536926 | -0.242028343313373 | 0.106232574850299 |
| 5090 | -0.0471470769230769 | 0.0626999600798403 | 0.204262175648703 | -0.00552279441117765 |
| 5091 | -0.0296233846153846 | -0.144567265469062 | 0.149034231536926 | 0.00178678642714571 |
| 5092 | -0.000208615384615385 | -0.116465988023952 | -0.0779688622754491 | 0.133846546906188 |
| 5093 | -0.00187753846153846 | 0.0784561676646707 | -0.0702531936127745 | 0.0149440319361277 |
| 5094 | -0.0442264615384615 | -0.0696846706586826 | 0.0576644710578842 | -0.126699401197605 |
| 5095 | -0.0805255384615385 | -0.131410019960080 | 0.244464870259481 | 0.0211165668662675 |
| 5096 | -0.0638363076923077 | -0.0477559281437126 | 0.0747201596806387 | 0.125399920159681 |
| 5097 | -0.0227390769230769 | -0.0873900998003992 | -0.167714271457086 | -0.00893393213572854 |
| 5098 | 0.000834461538461538 | -0.0545782035928144 | -0.153095109780439 | -0.0531162874251497 |
| 5099 | -0.00250338461538462 | 0.113217285429142 | 0.123044610778443 | 0.108181796407186 |
| 5100 | -0.0118910769230769 | 0.214414371257485 | 0.0775627744510978 | 0.160161037924152 |
| 5101 | 0.0137686153846154 | -0.0961615968063872 | -0.163653393213573 | -0.00259896207584830 |
| 5102 | 0.0358818461538462 | -0.111430499001996 | -0.0885271457085828 | -0.0969737724550898 |
| 5103 | 0.0544486153846154 | 0.0821921756487026 | 0.107613273453094 | 0.0407712175648703 |

| | | | | |
|---|---|---|---|---|
| 5104 | 0.0369249230769231 | -0.0521416766467066 | 0.209947405189621 | 0.132709500998004 |
| 5105 | -0.0373421538461538 | 0.00129948103792415 | -0.120201996007984 | 0.0141318562874252 |
| 5106 | -0.0899132307692308 | 0.182901956087824 | -0.297256287425150 | -0.0979483832335329 |
| 5107 | -0.109940307692308 | 0.190049101796407 | 0.127105489021956 | -0.0422331337325349 |
| 5108 | -0.0807341538461538 | -0.104445788423154 | 0.166496007984032 | 0.0908012375249501 |
| 5109 | -0.0304578461538462 | 0.00779688622754491 | -0.276545808383234 | 0.0622126546906188 |
| 5110 | 0.00375507692307692 | 0.139694211576846 | -0.300504990019960 | -0.118577644710579 |
| 5111 | 0.0227390769230769 | -0.235855808383234 | 0.183145608782435 | -0.152201716566866 |
| 5112 | 0.0304578461538462 | -0.271753972055888 | 0.157968163672655 | 0.0578269061876247 |
| 5113 | 0.0663396923076923 | 0.0592888223552894 | -0.220911776447106 | 0.0880398403193613 |
| 5114 | 0.0924166153846154 | 0.0141318562874252 | -0.114110678642715 | -0.0925880239520958 |
| 5115 | 0.0742670769230769 | -0.166820878243513 | 0.203856087824351 | -0.0485681037924152 |
| 5116 | 0.0156461538461538 | 0.0243652694610778 | 0.201825648702595 | 0.0948621157684631 |
| 5117 | -0.0540313846153846 | 0.0467813173652695 | -0.127105489021956 | 0.0805678243512974 |
| 5118 | -0.0757273846153846 | -0.0250150099800399 | -0.0483244510978044 | 0.0131572455089820 |
| 5119 | -0.0642535384615385 | -0.0472686227544910 | 0.110455888223553 | -0.00665984031936128 |
| 5120 | -0.0354646153846154 | -0.0193297804391218 | 0.0467000998003992 | -0.0477559281437126 |
| 5121 | -0.0106393846153846 | 0.0472686227544910 | -0.110455888223553 | -0.0467813173652695 |
| 5122 | -0.0289975384615385 | 0.0350859880239521 | -0.111268063872255 | 0.0235530938123753 |
| 5123 | -0.0467298461538462 | -0.0355732934131737 | 0.0637557884231537 | 0.0207916966067864 |
| 5124 | -0.0342129230769231 | -0.0261520558882236 | 0.0998976047904192 | -0.0742328542914172 |
| 5125 | -0.00208615384615385 | 0.123288263473054 | -0.0154313373253493 | -0.0748825948103793 |
| 5126 | 0.0319181538461538 | 0.141156127744511 | -0.109643712574850 | 0.0726085029940120 |
| 5127 | 0.0202356923076923 | 0.0497051497005988 | 0.0824358283433134 | 0.0190049101796407 |
| 5128 | -0.0225304615384615 | -0.0956742914171657 | 0.151876846307385 | -0.150902235528942 |
| 5129 | -0.0613329230769231 | 0.115166506986028 | -0.0430453093812375 | -0.0490554091816367 |
| 5130 | -0.0728067692307692 | 0.474310578842315 | -0.0783749500998004 | 0.0974610778443114 |
| 5131 | -0.0567433846153846 | 0.0384971257485030 | 0.0564462075848303 | -0.0175429940119760 |
| 5132 | -0.0465212307692308 | -0.654613572854291 | 0.0166496007984032 | -0.0563649900199601 |
| 5133 | -0.0602898461538462 | -0.502411856287425 | -0.102334131736527 | 0.115328942115768 |
| 5134 | -0.0876184615384615 | 0.178028902195609 | -0.0678166666666667 | 0.0120201996007984 |
| 5135 | -0.0801083076923077 | 0.252586626746507 | 0.00812175648702595 | -0.0752074650698603 |
| 5136 | -0.0465212307692308 | -0.0555528143712575 | 0.0146191616766467 | 0.105745269461078 |
| 5137 | -0.00980492307692308 | -0.133359241516966 | 0.0661923153692615 | 0.0661110978043912 |
| 5138 | 0.00229476923076923 | 0.0172181237524950 | -0.0385783433133733 | -0.0768318163672655 |
| 5139 | -0.0310836923076923 | 0.797718922155689 | 0.0775627744510978 | -0.0243652694610778 |
| 5140 | -0.0611243076923077 | 0.613030179640719 | -0.128729840319361 | 0.0646491816367266 |
| 5141 | -0.0634190769230769 | -0.659649061876248 | 0.219287425149701 | 0.00308626746506986 |
| 5142 | -0.0194012307692308 | -0.413397405189621 | 0.239185728542914 | -0.0203043912175649 |
| 5143 | 0.0267027692307692 | 0.666958642714571 | -0.557964670658683 | 0.105745269461078 |
| 5144 | 0.0302492307692308 | 0.366128782435130 | -0.180709081836327 | 0.0233906586826347 |
| 5145 | 0.0100135384615385 | -0.581030459081836 | 0.585984730538922 | -0.214739241516966 |
| 5146 | -0.0252424615384615 | -0.0974610778443114 | 0.438574850299401 | -0.0133196806387226 |
| 5147 | -0.0200270769230769 | 0.660623672654691 | -0.989636027944112 | 0.280200598802395 |

| | | | | |
|---|---|---|---|---|
| 5148 | 0.00917907692307692 | 0.272566147704591 | 0.179490818363273 | -0.0592888223552894 |
| 5149 | 0.00792738461538461 | -0.146354051896208 | 0.937250698602794 | -0.279063552894212 |
| 5150 | -0.0210701538461538 | -0.168282794411178 | -0.383752994011976 | 0.0946996806387226 |
| 5151 | -0.0723895384615385 | -0.382859600798403 | -0.639588323353293 | 0.228871097804391 |
| 5152 | -0.104099076923077 | -0.488767305389222 | 0.125075049900200 | -0.0352484231536926 |
| 5153 | -0.0951286153846154 | -0.0836540918163673 | 0.694816267465070 | -0.155287984031936 |
| 5154 | -0.0569520000000000 | -0.0295631936127745 | -0.594918662674651 | -0.0667608383233533 |
| 5155 | -0.00980492307692308 | -0.175429940119760 | -0.129948103792415 | 0.103146307385230 |
| 5156 | 0.00688430769230769 | 0.202556606786427 | 0.241622255489022 | 0.175429940119760 |
| 5157 | 0.00667569230769231 | 0.386920479041916 | -0.164059481037924 | -0.0506797604790419 |
| 5158 | 0.0141858461538462 | 0.0154313373253493 | 0.160810778443114 | -0.193785109780439 |
| 5159 | 0.0406800000000000 | -0.0610756087824351 | 0.190455189620759 | 0.0844662674650699 |
| 5160 | 0.0694689230769231 | 0.126212095808383 | -0.00649740518962076 | 0.306190219560878 |
| 5161 | 0.0527796923076923 | 0.0441823552894212 | -0.357763373253493 | -0.108506666666667 |
| 5162 | 0.0210701538461538 | -0.205967744510978 | 0.566086427145709 | -0.396666586826347 |
| 5163 | -0.0158547692307692 | -0.137257684630739 | 0.336646806387226 | 0.156587465069860 |
| 5164 | -0.0235735384615385 | 0.307652135728543 | -0.537660279441118 | 0.417945588822355 |
| 5165 | 0.0185667692307692 | 0.271104231536926 | -0.251368363273453 | -0.160161037924152 |
| 5166 | 0.0448523076923077 | 0.0300504990019960 | 0.476747105788423 | -0.291733493013972 |
| 5167 | 0.0415144615384615 | 0.0271266666666667 | 0.405681736526946 | 0.307164830339321 |
| 5168 | 0.00959630769230769 | 0.0500300199600798 | -0.698877145708583 | 0.286698003992016 |
| 5169 | 0.00187753846153846 | 0.0388219960079840 | -0.216850898203593 | -0.302291776447106 |
| 5170 | 0.0350473846153846 | -0.329743313373253 | 0.488929740518962 | -0.219449860279441 |
| 5171 | 0.0682172307692308 | -0.468300479041916 | 0.204262175648703 | 0.251774451097804 |
| 5172 | 0.0863667692307692 | -0.0245277045908184 | -0.355732934131737 | 0.114191896207585 |
| 5173 | 0.0826116923076923 | 0.186800399201597 | -0.233094411177645 | -0.188424750499002 |
| 5174 | 0.0671741538461538 | -0.0594512574850299 | 0.356545109780439 | -0.0319997205588822 |
| 5175 | 0.0630018461538462 | -0.174292894211577 | -0.0670044910179641 | 0.109968582834331 |
| 5176 | 0.0978406153846154 | -0.00519792415169661 | -0.177460379241517 | -0.0295631936127745 |
| 5177 | 0.148951384615385 | 0.0472686227544910 | -0.00121826347305389 | -0.121988782435130 |
| 5178 | 0.151454769230769 | 0.0323245908183633 | 0.117765469061876 | -0.0142942914171657 |
| 5179 | 0.106185230769231 | 0.0930753293413174 | 0.0357357285429142 | -0.0349235528942116 |
| 5180 | 0.0650880000000000 | 0.0724460678642715 | 0.142536826347305 | -0.0956742914171657 |
| 5181 | 0.0421403076923077 | -0.102334131736527 | 0.274921457085828 | 0.0362230339321357 |
| 5182 | 0.0538227692307692 | 0.127024271457086 | -0.162435129740519 | 0.00844662674650699 |
| 5183 | 0.0786480000000000 | 0.356382674650699 | 0.0442635728542914 | -0.104933093812375 |
| 5184 | 0.0811513846153846 | -0.0407712175648703 | 0.257459680638723 | 0.0211165668662675 |
| 5185 | 0.0700947692307692 | -0.165196526946108 | 0.0556340319361277 | 0.162922435129741 |
| 5186 | 0.0502763076923077 | 0.109806147704591 | -0.352890319361277 | 0.0126699401197605 |
| 5187 | 0.0446436923076923 | 0.0644867465069860 | -0.125887225548902 | -0.0691973652694611 |
| 5188 | 0.0655052307692308 | -0.216201157684631 | 0.121826347305389 | 0.0755323353293413 |
| 5189 | 0.0882443076923077 | -0.0147815968063872 | -0.119389820359281 | 0.0438574850299401 |
| 5190 | 0.0890787692307692 | 0.155612854291417 | -0.201013473053892 | -0.104608223552894 |
| 5191 | 0.0542400000000000 | -0.108993972055888 | -0.0154313373253493 | -0.0401214770459082 |

| | | | | |
|---|---|---|---|---|
| 5192 | 0.0210701538461538 | -0.0882022754491018 | 0.177866467065868 | 0.140343952095808 |
| 5193 | 0.0377593846153846 | 0.120851736526946 | -0.224566566866267 | 0.0185176047904192 |
| 5194 | 0.0630018461538462 | 0.0133196806387226 | -0.0921819361277445 | -0.102334131736527 |
| 5195 | 0.0696775384615385 | -0.273053453093812 | 0.143755089820359 | 0.0501924550898204 |
| 5196 | 0.0354646153846154 | -0.0862530538922156 | 0.0759384231536926 | 0.139856646706587 |
| 5197 | -0.0104307692307692 | 0.326819481037924 | -0.306190219560878 | 0.0105582834331337 |
| 5198 | -0.0106393846153846 | 0.0573396007984032 | -0.131978542914172 | -0.0303753692614770 |
| 5199 | 0.00479815384615385 | -0.258921596806387 | 0.318778942115768 | 0.199145469061876 |
| 5200 | 0.0365076923076923 | 0.0527914171656687 | -0.0994915169660679 | 0.140993692614770 |
| 5201 | 0.0340043076923077 | 0.140993692614770 | -0.229439620758483 | -0.140506387225549 |
| 5202 | -0.0146030769230769 | -0.0852784431137725 | -0.00527914171656687 | -0.0932377644710579 |
| 5203 | -0.0657138461538462 | 0.0748825948103793 | 0.240810079840319 | 0.106557445109780 |
| 5204 | -0.0901218461538462 | 0.137095249500998 | -0.0540096806387226 | -0.0144567265469062 |
| 5205 | -0.0438092307692308 | 0.262982475049900 | -0.0860906187624751 | -0.177379161676647 |
| 5206 | 0.0100135384615385 | 0.421519161676647 | -0.0280200598802395 | 0.0246901397205589 |
| 5207 | 0.0398455384615385 | -0.596136926147705 | -0.131572455089820 | 0.195409461077844 |
| 5208 | 0.0383852307692308 | -0.899240878243513 | 0.0674105788423154 | -0.0258271856287425 |
| 5209 | 0.0237821538461538 | 0.0290758882235529 | 0.0162435129740519 | -0.198658163672655 |
| 5210 | 0.0481901538461539 | 0.505660558882236 | -0.185176047904192 | 0.0717963273453094 |
| 5211 | 0.0757273846153846 | 0.279388423153693 | -0.224160479041916 | 0.176891856287425 |
| 5212 | 0.0886615384615385 | -0.188424750499002 | 0.257865768463074 | -0.0972986427145709 |
| 5213 | 0.0550744615384615 | 0.0178678642714571 | 0.229033532934132 | -0.215713852295409 |
| 5214 | -0.00417230769230769 | 0.473660838323353 | -0.0324870259481038 | 0.0505173253493014 |
| 5215 | -0.0198184615384615 | 0.370027225548902 | 0.0353296407185629 | 0.226921876247505 |
| 5216 | -0.00354646153846154 | -0.694247744510978 | 0.269236227544910 | -0.0836540918163673 |
| 5217 | 0.0281630769230769 | -0.846124590818363 | 0.292789321357285 | -0.211165668662675 |
| 5218 | 0.0442264615384615 | 0.649253213572854 | -0.194516067864271 | 0.218312814371257 |
| 5219 | 0.0377593846153846 | 0.921332055888224 | -0.116141117764471 | 0.368402874251497 |
| 5220 | 0.0233649230769231 | -0.341925948103792 | 0.183145608782435 | -0.0555528143712575 |
| 5221 | 0.0108480000000000 | -0.291408622754491 | 0.0113704590818363 | -0.205642874251497 |
| 5222 | 0.0146030769230769 | 0.742490978043912 | -0.316342415169661 | 0.0497051497005988 |
| 5223 | 0.0262855384615385 | 0.392118403193613 | 0.0186800399201597 | 0.154638243512974 |
| 5224 | 0.0108480000000000 | -0.305053173652695 | 0.253398802395210 | -0.0240403992015968 |
| 5225 | -0.0383852307692308 | -0.327631656686627 | -0.177866467065868 | -0.206617485029940 |
| 5226 | -0.0769790769230769 | -0.382209860279441 | -0.0832480039920160 | -0.127511576846307 |
| 5227 | -0.0734326153846154 | -0.269155009980040 | 0.204262175648703 | 0.0562025548902196 |
| 5228 | -0.0225304615384615 | 0.345337085828343 | 0.215632634730539 | 0.101846826347305 |
| 5229 | 0.0208615384615385 | -0.0271266666666667 | -0.100709780439122 | -0.0740704191616766 |
| 5230 | 0.0319181538461538 | -0.592563353293413 | -0.0686288423153693 | -0.139044471057884 |
| 5231 | 0.0210701538461538 | 0.327631656686627 | 0.0982732534930140 | 0.126212095808383 |
| 5232 | 0 | 0.554878403193613 | -0.0629436127744511 | 0.247388702594810 |
| 5233 | -0.00917907692307692 | -0.372301317365269 | 0.0438574850299401 | -0.0256647504990020 |
| 5234 | -0.00855323076923077 | -0.478371457085828 | 0.00243652694610778 | -0.0826794810379242 |
| 5235 | -0.0156461538461538 | 0.260221077844311 | -0.0824358283433134 | 0.190373972055888 |

| | | | | |
|---|---|---|---|---|
| 5236 | -0.0621673846153846 | 0.235855808383234 | -0.0568522954091816 | 0.0451569660678643 |
| 5237 | -0.121205538461538 | -0.306677524950100 | 0.304565868263473 | -0.180627864271457 |
| 5238 | -0.145196307692308 | -0.0168932534930140 | 0.173399500998004 | 0.0164059481037924 |
| 5239 | -0.119536615384615 | 0.263144910179641 | -0.257053592814371 | 0.149277884231537 |
| 5240 | -0.0734326153846154 | 0.0402839121756487 | -0.0113704590818363 | -0.0539284630738523 |
| 5241 | -0.0340043076923077 | 0.154800678642715 | 0.155937724550898 | -0.0899890618762475 |
| 5242 | -0.0139772307692308 | 0.331042794411178 | 0.122232435129741 | 0.194434850299401 |
| 5243 | -0.0106393846153846 | -0.00487305389221557 | -0.313905888223553 | 0.132709500998004 |
| 5244 | -0.00146030769230769 | -0.126699401197605 | -0.0986793413173653 | -0.185013612774451 |
| 5245 | 0.0102221538461538 | 0.0329743313373254 | 0.224972654690619 | -0.131897325349301 |
| 5246 | 0.00229476923076923 | -0.298230898203593 | 0.0982732534930140 | 0.0505173253493014 |
| 5247 | -0.0392196923076923 | -0.215713852295409 | -0.0231470059880240 | -0.159998602794411 |
| 5248 | -0.0922080000000000 | 0.363854690618762 | -0.0527914171656687 | -0.257946986027944 |
| 5249 | -0.129550153846154 | 0.0165683832335329 | 0.329743313373253 | 0.00552279441117765 |
| 5250 | -0.110566153846154 | -0.486168343313373 | -0.0495427145708583 | 0.0196546506986028 |
| 5251 | -0.0630018461538462 | 0.0422331337325349 | -0.0134008982035928 | -0.150902235528942 |
| 5252 | -0.0198184615384615 | 0.410473572854291 | -0.156749900199601 | -0.00617253493013972 |
| 5253 | 0.00354646153846154 | -0.0623750898203593 | 0.303347604790419 | 0.117927904191617 |
| 5254 | -0.00813600000000000 | -0.201419560878244 | 0.413397405189621 | -0.0883647105788423 |
| 5255 | -0.0108480000000000 | 0.120364431137725 | -1.00141257485030 | 0.00276139720558882 |
| 5256 | -0.00959630769230769 | -0.0388219960079840 | -1.11633542914172 | 0.213277325349301 |
| 5257 | -0.00917907692307692 | -0.191998323353293 | 0.749232035928144 | 0.0126699401197605 |
| 5258 | -0.0340043076923077 | 0.124912614770459 | 1.64221916167665 | -0.168932534930140 |
| 5259 | -0.0907476923076923 | 0.00779688622754491 | -1.77379161676647 | 0.0745577245508982 |
| 5260 | -0.142692923076923 | -0.179815688622755 | -1.09643712574850 | 0.243002954091816 |
| 5261 | -0.139772307692308 | 0.180627864271457 | 2.17094550898204 | -0.0797556487025948 |
| 5262 | -0.0680086153846154 | 0.223348303393214 | 0.791465169660679 | -0.184526307385230 |
| 5263 | 0.00584123076923077 | -0.253236367265469 | -1.95165808383234 | 0.176729421157685 |
| 5264 | 0.0667569230769231 | -0.268180399201597 | -0.839789620758483 | 0.153501197604790 |
| 5265 | 0.0882443076923077 | 0.192648063872255 | 1.80506037924152 | -0.271266666666667 |
| 5266 | 0.0798996923076923 | 0.0508421956087824 | -0.185988223552894 | -0.108181796407186 |
| 5267 | 0.0869926153846154 | -0.261682994011976 | -0.625781337325349 | 0.306839960079840 |
| 5268 | 0.0794824615384615 | 0.0808926946107784 | 0.203856087824351 | 0.0649740518962076 |
| 5269 | 0.0479815384615385 | 0.237642594810379 | -0.226597005988024 | -0.255835329341317 |
| 5270 | -0.0112652307692308 | -0.0791059081836327 | 0.0994915169660679 | -0.0232282235528942 |
| 5271 | -0.0703033846153846 | -0.101359520958084 | 0.954712475049900 | 0.208079401197605 |
| 5272 | -0.0819858461538462 | 0.208891576846307 | 0.00284261477045908 | -0.00682227544910180 |
| 5273 | -0.0477729230769231 | 0.186150658682635 | -1.71490888223553 | -0.144242395209581 |
| 5274 | 0.0177323076923077 | -0.182089780439122 | 0.591263872255489 | 0.0614004790419162 |
| 5275 | 0.0780221538461538 | -0.177054291417166 | 1.45257614770459 | 0.107044750499002 |
| 5276 | 0.104099076923077 | 0.0531162874251497 | -0.808926946107784 | -0.0802429540918164 |
| 5277 | 0.103681846153846 | 0.0597761277445110 | -1.48993622754491 | 0.0102334131736527 |
| 5278 | 0.0970061538461539 | 0.154150938123753 | 0.615223053892216 | 0.138882035928144 |
| 5279 | 0.109940307692308 | 0.246251656686627 | 1.48059620758483 | -0.0352484231536926 |

| | | | | |
|---|---|---|---|---|
| 5280 | 0.120162461538462 | 0.131247584830339 | -0.653395309381238 | -0.0542533333333333 |
| 5281 | 0.0849064615384615 | 0.0347611177644711 | -0.815830439121757 | 0.104608223552894 |
| 5282 | 0.0123083076923077 | -0.0963240319361277 | 0.585578642714571 | 0.0266393612774451 |
| 5283 | -0.0429747692307692 | -0.121339041916168 | 0.833698303393214 | -0.148790578842315 |
| 5284 | -0.0260769230769231 | -0.0540908982035928 | -0.219693512974052 | -0.000162435129740519 |
| 5285 | 0.0360904615384615 | 0.109643712574850 | -0.329743313373253 | 0.157562075848303 |
| 5286 | 0.104307692307692 | 0.221886387225549 | 0.325682435129741 | -0.0183551696606786 |
| 5287 | 0.146656615384615 | -0.0578269061876247 | -0.147003792415170 | -0.117440598802395 |
| 5288 | 0.147073846153846 | -0.156912335329341 | -0.208323053892216 | 0.00877149700598802 |
| 5289 | 0.127046769230769 | 0.0191673453093812 | 0.00365479041916168 | 0.0691973652694611 |
| 5290 | 0.102012923076923 | -0.127349141716567 | 0.101115868263473 | 0.000162435129740519 |
| 5291 | 0.0965889230769231 | -0.440686506986028 | -0.252992714570858 | -0.0284261477045908 |
| 5292 | 0.0807341538461538 | -0.138557165668663 | -0.0962428143712575 | 0.0440199201596806 |
| 5293 | 0.0223218461538462 | 0.449458003992016 | 0.346392914171657 | 0.105257964071856 |
| 5294 | -0.0471470769230769 | 0.306677524950100 | -0.147409880239521 | 0.0802429540918164 |
| 5295 | -0.0763532307692308 | -0.105745269461078 | -0.0564462075848303 | 0.0371976447105788 |
| 5296 | -0.0333784615384615 | 0.0188424750499002 | 0.196546506986028 | -0.0261520558882236 |
| 5297 | 0.0323353846153846 | 0.214414371257485 | 0.0300504990019960 | -0.0138069860279441 |
| 5298 | 0.0761446153846154 | 0.0921007185628743 | -0.339083333333333 | 0.133359241516966 |
| 5299 | 0.107019692307692 | -0.0706592814371258 | -0.0714714570858283 | 0.0726085029940120 |
| 5300 | 0.114947076923077 | -0.131572455089820 | 0.260302295409182 | -0.128648622754491 |
| 5301 | 0.113695384615385 | -0.0352484231536926 | -0.0353296407185629 | 0.00292383233532934 |
| 5302 | 0.119119384615385 | 0.271429101796407 | -0.145379441117764 | 0.226921876247505 |
| 5303 | 0.105142153846154 | 0.496564191616767 | 0.173805588822355 | 0.0579893413173653 |
| 5304 | 0.0669655384615385 | -0.00129948103792415 | 0.512888922155689 | -0.157886946107784 |
| 5305 | 0.000625846153846154 | -0.499163153692615 | 0.0215226546906188 | 0.0144567265469062 |
| 5306 | -0.0500676923076923 | -0.0105582834331337 | -0.341113772455090 | 0.164546786427146 |
| 5307 | -0.0344215384615385 | 0.269967185628743 | 0.0101521956087824 | -0.0799180838323353 |
| 5308 | 0.0377593846153846 | -0.315611457085828 | 0.423955688622755 | -0.217663073852295 |
| 5309 | 0.102847384615385 | -0.443610339321357 | -0.0909636726546906 | 0.0747201596806387 |
| 5310 | 0.111400615384615 | 0.155612854291417 | -0.537254191616767 | 0.104283353293413 |
| 5311 | 0.0861581538461538 | 0.213439760479042 | 0.0247713572854291 | -0.225622395209581 |
| 5312 | 0.0648793846153846 | -0.134983592814371 | 0.386189520958084 | -0.144567265469062 |
| 5313 | 0.0869926153846154 | -0.0295631936127745 | 0.112080239520958 | 0.193947544910180 |
| 5314 | 0.109523076923077 | -0.0419082634730539 | -0.209947405189621 | 0.0646491816367266 |
| 5315 | 0.101387076923077 | -0.227409181636727 | 0.170556886227545 | -0.231794930139721 |
| 5316 | 0.0728067692307692 | 0.0316748502994012 | 0.304159780439122 | 0.0118577644710579 |
| 5317 | 0.0292061538461538 | 0.269642315369262 | 0.0726897205588822 | 0.234069021956088 |
| 5318 | 0.0141858461538462 | 0.116790858283433 | -0.210759580838323 | -0.0990854291417166 |
| 5319 | 0.0377593846153846 | -0.0266393612774451 | -0.162841217564870 | -0.257784550898204 |
| 5320 | 0.0865753846153846 | 0.152689021956088 | 0.0856845309381238 | 0.0940499401197605 |
| 5321 | 0.123083076923077 | 0.262820039920160 | -0.0495427145708583 | 0.197196247504990 |
| 5322 | 0.121831384615385 | -0.0675730139720559 | -0.203856087824351 | -0.137744990019960 |
| 5323 | 0.102847384615385 | -0.200932255489022 | -0.332585928143713 | -0.175267504990020 |

| | | | | |
|---|---|---|---|---|
| 5324 | 0.0882443076923077 | 0.0155937724550898 | 0.00934001996007984 | 0.134171417165669 |
| 5325 | 0.0780221538461538 | -0.0714714570858283 | 0.109643712574850 | 0.134821157684631 |
| 5326 | 0.0467298461538462 | -0.134171417165669 | -0.00893393213572854 | -0.0946996806387226 |
| 5327 | 0.00208615384615385 | 0.211652974051896 | -0.0747201596806387 | -0.0245277045908184 |
| 5328 | -0.0425575384615385 | 0.129135928143713 | -0.00446696606786427 | 0.0908012375249501 |
| 5329 | -0.0638363076923077 | -0.202556606786427 | 0.292789321357285 | 0.0311875449101796 |
| 5330 | -0.0342129230769231 | -0.0753699001996008 | 0.0848723552894212 | -0.0563649900199601 |
| 5331 | 0.0287889230769231 | -0.0402839121756487 | -0.274515369261477 | -0.106395009980040 |
| 5332 | 0.0830289230769231 | -0.221074211576846 | -0.139288123752495 | 0.00373600798403194 |
| 5333 | 0.0824030769230769 | -0.246738962075848 | 0.361824251497006 | 0.0449945309381238 |
| 5334 | 0.0554916923076923 | 0.0302129341317365 | 0.0990854291417166 | -0.0664359680638723 |
| 5335 | 0.0425575384615385 | 0.190049101796407 | -0.286698003992016 | -0.0388219960079840 |
| 5336 | 0.0471470769230769 | 0.0828419161676647 | 0.121826347305389 | 0.0784561676646707 |
| 5337 | 0.0594553846153846 | 0.222373692614770 | 0.395935628742515 | 0.0704968463073852 |
| 5338 | 0.0352560000000000 | 0.198820598802395 | 0.0280200598802395 | -0.0204668263473054 |
| 5339 | -0.0267027692307692 | -0.0787810379241517 | -0.364666866267465 | -0.0371976447105788 |
| 5340 | -0.0909563076923077 | -0.0513295009980040 | -0.00934001996007984 | 0.0154313373253493 |
| 5341 | -0.106602461538462 | 0.0100709780439122 | 0.185176047904192 | 0.0591263872255489 |
| 5342 | -0.0536141538461538 | -0.107044750499002 | -0.194109980039920 | 0.0167308183632735 |
| 5343 | 0.00438092307692308 | -0.0612380439121757 | -0.347205089820359 | -0.0664359680638723 |
| 5344 | 0.0348387692307692 | 0.0701719760479042 | 0.00487305389221557 | -0.0357357285429142 |
| 5345 | 0.0377593846153846 | 0.0935626347305389 | 0.237155289421158 | 0.0683851896207585 |
| 5346 | 0.0150203076923077 | 0.0846287025948104 | -0.0743140718562874 | 0.0990854291417166 |
| 5347 | 0.0118910769230769 | -0.0381722554890220 | -0.134008982035928 | -0.0279388423153693 |
| 5348 | 0.0206529230769231 | -0.126861836327345 | 0.0605070858283433 | -0.0834916566866268 |
| 5349 | 0 | -0.0479183632734531 | 0.242434431137725 | 0.0701719760479042 |
| 5350 | -0.0500676923076923 | -0.0579893413173653 | 0.103958483033932 | 0.113704590818363 |
| 5351 | -0.109105846153846 | -0.222373692614770 | -0.153095109780439 | -0.0539284630738523 |
| 5352 | -0.127046769230769 | -0.0277764071856287 | -0.164871656686627 | -0.0963240319361277 |
| 5353 | -0.0890787692307692 | 0.237805029940120 | -0.0369539920159681 | 0.0589639520958084 |
| 5354 | -0.0264941538461538 | 0.000649740518962076 | 0.118171556886228 | 0.0224160479041916 |
| 5355 | 0.0112652307692308 | -0.326494610778443 | -0.108019361277445 | -0.0830043512974052 |
| 5356 | 0.00855323076923077 | -0.184851177644711 | -0.125075049900200 | -0.00259896207584830 |
| 5357 | -0.0120996923076923 | 0.612055568862275 | 0.0276139720558882 | 0.00665984031936128 |
| 5358 | -0.0248252307692308 | 0.794957524950100 | 0.136851596806387 | -0.0107207185628743 |
| 5359 | -0.0108480000000000 | 0.229033532934132 | 0.0190861277445110 | 0.0514919361277445 |
| 5360 | -0.00688430769230769 | -0.0518168063872256 | -0.172993413173653 | 0.0787810379241517 |
| 5361 | -0.0325440000000000 | -0.293032974051896 | -0.0462940119760479 | 0.0667608383233533 |
| 5362 | -0.0888701538461539 | -0.217338203592814 | -0.0239591816367265 | -0.0168932534930140 |
| 5363 | -0.134765538461538 | 0.234881197604790 | 0.0430453093812375 | -0.0477559281437126 |
| 5364 | -0.120162461538462 | -0.171206626746507 | -0.00568522954091816 | 0.0859281836327345 |
| 5365 | -0.0728067692307692 | -1.05176746506986 | 0.130760279441118 | 0.101846826347305 |
| 5366 | -0.0262855384615385 | -0.0168932534930140 | -0.0117765469061876 | -0.0472686227544910 |
| 5367 | -0.0191926153846154 | 1.28697353293413 | 0.166902095808383 | -0.0880398403193613 |

| | | | | |
|---|---|---|---|---|
| 5368 | -0.0300406153846154 | -0.0181927345309381 | 0.922631536926148 | 0.0282637125748503 |
| 5369 | -0.0279544615384615 | -0.921656926147705 | -0.353296407185629 | 0.211977844311377 |
| 5370 | -0.0204443076923077 | 0.379610898203593 | -1.21542085828343 | 0.106882315369261 |
| 5371 | 0.0166892307692308 | 0.525152774451098 | 1.08181796407186 | -0.182252215568862 |
| 5372 | 0.0467298461538462 | -0.561538243512974 | 1.47044401197605 | -0.0232282235528942 |
| 5373 | 0.0348387692307692 | -0.356382674650699 | -1.97845988023952 | 0.254373413173653 |
| 5374 | 0.0110566153846154 | 0.181440039920160 | -1.15938073852295 | 0.0285885828343313 |
| 5375 | -0.000208615384615385 | -0.146191616766467 | 2.52139930139721 | -0.281987385229541 |
| 5376 | 0.0354646153846154 | 0.0596136926147705 | -0.0799993013972056 | -0.0412585229540918 |
| 5377 | 0.0786480000000000 | 0.797718922155689 | -2.61276906187625 | 0.160973213572854 |
| 5378 | 0.0692603076923077 | 0.129135928143713 | 0.622938722554890 | -0.146678922155689 |
| 5379 | 0.0456867692307692 | -0.350535009980040 | 2.00607385229541 | -0.220586906187625 |
| 5380 | 0.0241993846153846 | 0.449295568862275 | -1.01359520958084 | 0.136445508982036 |
| 5381 | -0.00166892307692308 | 0.248688183632735 | -1.04120918163673 | 0.122800958083832 |
| 5382 | -0.0156461538461538 | -0.520767025948104 | 1.54029111776447 | -0.206455049900200 |
| 5383 | -0.0346301538461538 | -0.147166227544910 | 0.0519792415169661 | 0.0220911776447106 |
| 5384 | -0.0809427692307692 | 0.367590698602794 | -0.891768862275449 | 0.255185588822355 |
| 5385 | -0.136017230769231 | -0.113704590818363 | 0.962022055888224 | -0.0953494211576846 |
| 5386 | -0.159382153846154 | -0.365641477045908 | 0.124262874251497 | -0.268180399201597 |
| 5387 | -0.117241846153846 | 0.138557165668663 | -1.20729910179641 | 0.110780758483034 |
| 5388 | -0.0191926153846154 | 0.190211536926148 | 0.718369361277445 | 0.159186427145709 |
| 5389 | 0.0652966153846154 | -0.225297524950100 | 1.28689231536926 | -0.244627305389222 |
| 5390 | 0.0838633846153846 | -0.138882035928144 | -1.32100369261477 | -0.100060039920160 |
| 5391 | 0.0577864615384615 | 0.0540908982035928 | -0.926286327345309 | 0.260545948103792 |
| 5392 | 0.0256596923076923 | 0.0214414371257485 | 1.72830978043912 | 0.0792683433133733 |
| 5393 | 0.0110566153846154 | 0.135470898203593 | 0.728521556886228 | -0.216850898203593 |
| 5394 | 0.000625846153846154 | 0.103796047904192 | -1.80627864271457 | 0.0844662674650699 |
| 5395 | -0.0373421538461538 | 0.0131572455089820 | -0.557964670658683 | 0.300992295409182 |
| 5396 | -0.0909563076923077 | 0.173805588822355 | 1.50617974051896 | -0.136607944111776 |
| 5397 | -0.142901538461538 | 0.129785668662675 | 0.0442635728542914 | -0.285885828343313 |
| 5398 | -0.151872000000000 | -0.244952175648703 | -1.29460798403194 | 0.172668542914172 |
| 5399 | -0.108271384615385 | -0.145379441117764 | 0.0385783433133733 | 0.245114610778443 |
| 5400 | -0.0730153846153846 | 0.162760000000000 | 0.942935928143713 | -0.204830698602794 |
| 5401 | -0.0732240000000000 | -0.0110455888223553 | -0.0633497005988024 | -0.102496566866267 |
| 5402 | -0.104307692307692 | -0.0635121357285429 | -0.379286027944112 | 0.261358123752495 |
| 5403 | -0.122457230769231 | 0.0956742914171657 | 0.343550299401198 | 0.0602634331337325 |
| 5404 | -0.103681846153846 | -0.129460798403194 | 0.183551696606786 | -0.176242115768463 |
| 5405 | -0.0648793846153846 | -0.271104231536926 | -0.244058782435130 | 0.0639994411177645 |
| 5406 | -0.0340043076923077 | 0.0503548902195609 | 0.110455888223553 | 0.128161317365269 |
| 5407 | -0.0227390769230769 | 0.0706592814371258 | -0.0105582834331337 | -0.137095249500998 |
| 5408 | -0.0100135384615385 | -0.233581716566866 | -0.514919361277445 | -0.0363854690618763 |
| 5409 | 0.0150203076923077 | -0.178028902195609 | -0.0247713572854291 | 0.128811057884232 |
| 5410 | 0.0598726153846154 | 0.0942123752495010 | 0.363854690618762 | -0.129948103792415 |
| 5411 | 0.113278153846154 | 0.0467813173652695 | -0.0280200598802395 | -0.143105349301397 |

| | | | | |
|---|---|---|---|---|
| 5412 | 0.146239384615385 | -0.0341113772455090 | -0.446696606786427 | 0.265906307385230 |
| 5413 | 0.119536615384615 | 0.132871936127745 | 0.127511576846307 | 0.101359520958084 |
| 5414 | 0.0665483076923077 | 0.132709500998004 | 0.432889620758483 | -0.298393333333333 |
| 5415 | 0.0415144615384615 | -0.137420119760479 | -0.190049101796407 | 0.0297256287425150 |
| 5416 | 0.0415144615384615 | -0.0523041117764471 | -0.324870259481038 | 0.310738403193613 |
| 5417 | 0.0565347692307692 | 0.207916966067864 | 0.234312674650699 | -0.157562075848303 |
| 5418 | 0.0388024615384615 | -0.0911261077844311 | 0.362636427145709 | -0.309601357285429 |
| 5419 | -0.0175236923076923 | -0.157562075848303 | -0.434513972055888 | 0.224647784431138 |
| 5420 | -0.0667569230769231 | 0.135470898203593 | -0.242028343313373 | 0.236180678642715 |
| 5421 | -0.0665483076923077 | 0.181277604790419 | 0.421519161676647 | -0.239916686626747 |
| 5422 | -0.0110566153846154 | -0.0245277045908184 | 0.219693512974052 | -0.150739800399202 |
| 5423 | 0.0561175384615385 | -0.291408622754491 | -0.390656487025948 | 0.262657604790419 |
| 5424 | 0.0815686153846154 | -0.0869027944111777 | -0.189236926147705 | 0.0323245908183633 |
| 5425 | 0.0796910769230769 | 0.197845988023952 | 0.472280139720559 | -0.275814850299401 |
| 5426 | 0.0736412307692308 | -0.0368727744510978 | 0.100709780439122 | 0.0688724950099800 |
| 5427 | 0.0721809230769231 | -0.172181237524950 | -0.410148702594810 | 0.218800119760479 |
| 5428 | 0.104933538461538 | -0.0662735329341317 | -0.106801097804391 | -0.0579893413173653 |
| 5429 | 0.105768000000000 | -0.111105628742515 | 0.269236227544910 | -0.0362230339321357 |
| 5430 | 0.0657138461538462 | -0.208241836327345 | 0.0243652694610778 | 0.145541876247505 |
| 5431 | 0.00771876923076923 | -0.249987664670659 | -0.105582834331337 | -0.0407712175648703 |
| 5432 | -0.0294147692307692 | 0.260545948103792 | -0.107207185628743 | -0.112729980039920 |
| 5433 | -0.00354646153846154 | 0.764419720558882 | 0.0381722554890220 | 0.0938875049900200 |
| 5434 | 0.0106393846153846 | 0.476097365269461 | 0.237561377245509 | 0.0537660279441118 |
| 5435 | 0.0120996923076923 | 0.113054850299401 | 0.164871656686627 | -0.132709500998004 |
| 5436 | -0.00166892307692308 | 0.0508421956087824 | -0.108831536926148 | -0.00341113772455090 |
| 5437 | -0.0264941538461538 | -0.468625349301397 | -0.228627445109780 | 0.277276766467066 |
| 5438 | -0.0216960000000000 | -0.482594770459082 | 0.110455888223553 | 0.0214414371257485 |
| 5439 | 0.00166892307692308 | 0.584604031936128 | 0.0978671656686627 | -0.233419281437126 |
| 5440 | 0.0367163076923077 | -0.239916686626747 | -0.185988223552894 | 0.0969737724550898 |
| 5441 | 0.0335870769230769 | -0.826145069860280 | -0.287916267465070 | 0.223348303393214 |
| 5442 | -0.0179409230769231 | 1.07938143712575 | 0.0491366267465070 | -0.0989229940119761 |
| 5443 | -0.0433920000000000 | 0.808277205588822 | 0.259084031936128 | -0.216850898203593 |
| 5444 | -0.00897046153846154 | -1.04656954091816 | -0.110455888223553 | 0.0523041117764471 |
| 5445 | 0.0707206153846154 | -0.302616646706587 | -0.194109980039920 | 0.184039001996008 |
| 5446 | 0.134556923076923 | 0.862043233532934 | 0.211571756487026 | -0.00129948103792415 |
| 5447 | 0.145404923076923 | -0.396179281437126 | 0.278170159680639 | -0.116303552894212 |
| 5448 | 0.119328000000000 | -0.980296007984032 | -0.233500499001996 | -0.0149440319361277 |
| 5449 | 0.0788566153846154 | 0.398940678642715 | -0.145379441117764 | 0.0583142115768463 |
| 5450 | 0.0619587692307692 | 0.401052335329341 | 0.260302295409182 | -0.0581517764471058 |
| 5451 | 0.0502763076923077 | -0.407224870259481 | 0.0150252495009980 | -0.112405109780439 |
| 5452 | 0.0323353846153846 | 0.365154171656687 | -0.217663073852295 | -0.0415833932135729 |
| 5453 | 0.00312923076923077 | 0.587365429141717 | 0.0393905189620759 | -0.0240403992015968 |
| 5454 | -0.0362990769230769 | -0.477396846307385 | 0.246089221556886 | -0.00357357285429142 |
| 5455 | -0.0446436923076923 | 0.0583142115768463 | 0.0889332335329341 | 0.0484056686626747 |

| | | | | |
|---|---|---|---|---|
| 5456 | -0.0296233846153846 | 0.823058802395210 | -0.0795932135728543 | -0.00454818363273453 |
| 5457 | 0.0133513846153846 | -0.218800119760479 | -0.0560401197604790 | -0.0782937325349302 |
| 5458 | 0.0448523076923077 | -0.599060758483034 | 0.0300504990019960 | 0.0453194011976048 |
| 5459 | 0.0400541538461538 | 0.380747944111776 | 0.119795908183633 | 0.0562025548902196 |
| 5460 | 0.0490246153846154 | 0.408361916167665 | -0.0308626746506986 | -0.116465988023952 |
| 5461 | 0.0784393846153846 | -0.371164271457086 | -0.307002395209581 | -0.0940499401197605 |
| 5462 | 0.103890461538462 | -0.139369341317365 | -0.0755323353293413 | 0.149602754491018 |
| 5463 | 0.0974233846153846 | 0.188424750499002 | 0.287916267465070 | 0.241053732534930 |
| 5464 | 0.0598726153846154 | -0.158699121756487 | 0.0527914171656687 | -0.0289134530938124 |
| 5465 | 0.00959630769230769 | -0.0229033532934132 | -0.312687624750499 | -0.139856646706587 |
| 5466 | -0.0210701538461538 | 0.125075049900200 | 0.0856845309381238 | 0.157237205588822 |
| 5467 | -0.00187753846153846 | -0.251936886227545 | 0.385377345309381 | 0.268180399201597 |
| 5468 | 0.0377593846153846 | -0.156262594810379 | 0.0751262475049900 | -0.0259896207584830 |
| 5469 | 0.0627932307692308 | 0.129948103792415 | -0.124262874251497 | -0.149277884231537 |
| 5470 | 0.0521538461538462 | -0.0929128942115768 | 0.0755323353293413 | 0.0284261477045908 |
| 5471 | 0.0225304615384615 | -0.0391468662674651 | 0.244464870259481 | 0.133521676646707 |
| 5472 | 0.0287889230769231 | 0.130435409181637 | -0.0938062874251497 | 0.0146191616766467 |
| 5473 | 0.0602898461538462 | -0.168607664670659 | -0.133196806387226 | -0.156749900199601 |
| 5474 | 0.0798996923076923 | -0.118740079840319 | -0.0552279441117765 | -0.0912885429141717 |
| 5475 | 0.0582036923076923 | 0.357032415169661 | -0.0544157684630739 | 0.0612380439121757 |
| 5476 | 0.00396369230769231 | 0.249987664670659 | -0.0649740518962076 | -0.00113704590818363 |
| 5477 | -0.0454781538461538 | -0.270454491017964 | 0.000406087824351297 | -0.116303552894212 |
| 5478 | -0.0680086153846154 | -0.0261520558882236 | 0.0637557884231537 | -0.0597761277445110 |
| 5479 | -0.0375507692307692 | 0.299692814371258 | -0.118577644710579 | 0.0211165668662675 |
| 5480 | 0.00897046153846154 | -0.0730958083832335 | 0.554715968063872 | 0.0425580039920160 |
| 5481 | 0.0402627692307692 | -0.0895017564870260 | 0.125075049900200 | -0.0243652694610778 |
| 5482 | 0.0546572307692308 | 0.279063552894212 | -1.17521816367265 | -0.0527914171656687 |
| 5483 | 0.0542400000000000 | 0.0693598003992016 | 0.160810778443114 | 0.00341113772455090 |
| 5484 | 0.0673827692307692 | -0.197196247504990 | 1.57886946107784 | 0.0121826347305389 |
| 5485 | 0.0767704615384615 | 0.0961615968063872 | -0.822327844311377 | 0.0371976447105788 |
| 5486 | 0.0521538461538462 | 0.0375225149700599 | -1.85825788423154 | 0.0136445508982036 |
| 5487 | -0.00584123076923077 | -0.351834491017964 | 1.69704101796407 | -0.0407712175648703 |
| 5488 | -0.0901218461538462 | -0.163897045908184 | 1.28729840319361 | 0.0162435129740519 |
| 5489 | -0.154375384615385 | 0.248525748502994 | -1.84201437125749 | 0.0930753293413174 |
| 5490 | -0.163554461538462 | -0.0347611177644711 | -0.423143512974052 | 0.0172181237524950 |
| 5491 | -0.141858461538462 | -0.340951337325349 | 1.45907355289421 | -0.0998976047904192 |
| 5492 | -0.122665846153846 | 0.00243652694610778 | -0.00730958083832335 | -0.118090339321357 |
| 5493 | -0.115572923076923 | 0.0974610778443114 | -1.01197085828343 | -0.0419082634730539 |
| 5494 | -0.0993009230769231 | -0.137095249500998 | 0.588015169660679 | 0.0276139720558882 |
| 5495 | -0.0650880000000000 | -0.0891768862275449 | 0.0962428143712575 | 0.0206292614770459 |
| 5496 | -0.0133513846153846 | 0.0459691417165669 | -0.774003393213573 | -0.0495427145708583 |
| 5497 | 0.0204443076923077 | 0.00487305389221557 | 0.827606986027944 | -0.0579893413173653 |
| 5498 | -0.0110566153846154 | -0.0207916966067864 | 0.441011377245509 | 0.112729980039920 |
| 5499 | -0.0872012307692308 | 0.0818673053892216 | -1.12567544910180 | 0.0773191217564870 |

| | | | | |
|---|---|---|---|---|
| 5500 | -0.151246153846154 | 0.130922714570858 | -0.105988922155689 | -0.162110259481038 |
| 5501 | -0.152706461538462 | 0.0802429540918164 | 1.28811057884232 | -0.0605883033932136 |
| 5502 | -0.111817846153846 | 0.130760279441118 | -0.0925880239520958 | 0.180790299401198 |
| 5503 | -0.0748929230769231 | 0.104283353293413 | -1.13542155688623 | 0.0825170459081836 |
| 5504 | -0.0630018461538462 | 0.0167308183632735 | 0.137663772455090 | -0.0675730139720559 |
| 5505 | -0.0671741538461538 | -0.0896641916167665 | 0.920601097804391 | 0.0662735329341317 |
| 5506 | -0.0544486153846154 | 0.0652989221556886 | -0.217663073852295 | 0.163409740518962 |
| 5507 | -0.0221132307692308 | 0.175429940119760 | -1.00994041916168 | 0.0198170858283433 |
| 5508 | 0.0171064615384615 | -0.0334616367265469 | 0.128729840319361 | -0.0531162874251497 |
| 5509 | 0.0239907692307692 | -0.166333572854291 | 0.577456886227545 | 0.0472686227544910 |
| 5510 | 0 | -0.315936327345309 | -0.194516067864271 | -0.00519792415169661 |
| 5511 | -0.0260769230769231 | -0.0987605588822355 | -0.433295708582834 | -0.0769942514970060 |
| 5512 | -0.0365076923076923 | 0.0428828742514970 | 0.147815968063872 | 0.0550655089820359 |
| 5513 | -0.0141858461538462 | -0.0488929740518962 | 0.328931137724551 | 0.0107207185628743 |
| 5514 | 0.0146030769230769 | -0.162272694610778 | -0.231876147704591 | -0.0906388023952096 |
| 5515 | 0.0292061538461538 | 0.194434850299401 | -0.0718775449101796 | 0.00990854291417166 |
| 5516 | 0.0166892307692308 | 0.738917405189621 | 0.245277045908184 | 0.0716338922155689 |
| 5517 | 0.0175236923076923 | 0.187287704590818 | -0.0799993013972056 | -0.0545782035928144 |
| 5518 | 0.0481901538461539 | -0.325032694610778 | -0.205886526946108 | -0.0930753293413174 |
| 5519 | 0.0713464615384615 | -0.0566898602794411 | 0.245683133732535 | 0.127836447105788 |
| 5520 | 0.0778135384615385 | 0.169419840319361 | 0.0812175648702595 | 0.128161317365269 |
| 5521 | 0.0523624615384615 | -0.0173805588822355 | -0.217663073852295 | -0.0758572055888224 |
| 5522 | 0.00625846153846154 | -0.101846826347305 | 0.172181237524950 | -0.00909636726546906 |
| 5523 | -0.0319181538461538 | 0.129460798403194 | 0.225784830339321 | 0.183714131736527 |
| 5524 | -0.0333784615384615 | 0.140019081836327 | -0.0763445109780439 | 0.0393093013972056 |
| 5525 | 0.00208615384615385 | 0.0639994411177645 | -0.262332734530938 | -0.147653532934132 |
| 5526 | 0.0400541538461538 | 0.0914509780439122 | 0.135633333333333 | 0.0524665469061876 |
| 5527 | 0.0475643076923077 | -0.163897045908184 | 0.137257684630739 | 0.159836167664671 |
| 5528 | 0.0325440000000000 | -0.579406107784431 | -0.241216167664671 | -0.0817048702594810 |
| 5529 | 0.0250338461538462 | -0.416158802395210 | -0.156343812375250 | -0.0914509780439122 |
| 5530 | 0.0373421538461538 | 0.197358682634731 | 0.218069161676647 | 0.200120079840319 |
| 5531 | 0.0546572307692308 | 0.309276487025948 | 0.201825648702595 | 0.0898266267465070 |
| 5532 | 0.0319181538461538 | -0.0583142115768463 | -0.204262175648703 | -0.117603033932136 |
| 5533 | -0.0156461538461538 | 0.111917804391218 | 0.0345174650698603 | 0.0107207185628743 |
| 5534 | -0.0525710769230769 | 0.429965788423154 | 0.304565868263473 | 0.0129948103792415 |
| 5535 | -0.0634190769230769 | 0.192972934131737 | 0.144567265469062 | -0.107694491017964 |
| 5536 | -0.0383852307692308 | 0.0238779640718563 | -0.239591816367265 | -0.0246901397205589 |
| 5537 | 0.000625846153846154 | -0.0160810778443114 | -0.106395009980040 | 0.174292894211577 |
| 5538 | 0.0254510769230769 | -0.0412585229540918 | 0.230251796407186 | 0.0601009980039920 |
| 5539 | 0.0254510769230769 | -0.110293453093812 | -0.0576644710578842 | -0.143592654690619 |
| 5540 | 0.0173150769230769 | 0.158536686626747 | -0.218475249500998 | 0.0690349301397206 |
| 5541 | 0.0429747692307692 | 0.430290658682635 | -0.0479183632734531 | 0.213927065868264 |
| 5542 | 0.0813600000000000 | -0.0120201996007984 | 0.145379441117764 | -0.0614004790419162 |
| 5543 | 0.0830289230769231 | -0.155937724550898 | 0.123450698602794 | -0.165521397205589 |

| | | | | |
|---|---|---|---|---|
| 5544 | 0.0655052307692308 | 0.0570147305389222 | -0.0479183632734531 | 0.0945372455089820 |
| 5545 | 0.0350473846153846 | 0.00438574850299401 | -0.0605070858283433 | 0.0734206786427146 |
| 5546 | 0.0267027692307692 | -0.363854690618762 | 0.130354191616766 | -0.184363872255489 |
| 5547 | 0.0519452307692308 | -0.417945588822355 | 0.153907285429142 | -0.0756947704590818 |
| 5548 | 0.0840720000000000 | -0.0940499401197605 | -0.0905575848303393 | 0.0921007185628743 |
| 5549 | 0.107854153846154 | -0.0159186427145709 | -0.219693512974052 | -0.0350859880239521 |
| 5550 | 0.0773963076923077 | 0.0898266267465070 | 0.00203043912175649 | -0.0953494211576846 |
| 5551 | 0.0271200000000000 | 0.173480718562874 | 0.170556886227545 | -0.0201419560878244 |
| 5552 | 0.0129341538461538 | -0.0909636726546906 | 0.00365479041916168 | -0.0477559281437126 |
| 5553 | 0.0469384615384615 | -0.219449860279441 | -0.0775627744510978 | -0.0769942514970060 |
| 5554 | 0.0803169230769231 | 0.137257684630739 | -0.0312687624750499 | -0.0233906586826347 |
| 5555 | 0.0711378461538462 | 0.186800399201597 | 0.183145608782435 | 0.0479183632734531 |
| 5556 | 0.0688430769230769 | -0.0155937724550898 | 0.238373552894212 | 0.0614004790419162 |
| 5557 | 0.0542400000000000 | -0.0181927345309381 | -0.173805588822355 | -0.0389844311377246 |
| 5558 | 0.0415144615384615 | 0.133846546906188 | -0.227815269461078 | -0.0857657485029940 |
| 5559 | 0.0786480000000000 | 0.160973213572854 | 0.193703892215569 | -0.0129948103792415 |
| 5560 | 0.111192000000000 | -0.0461315768463074 | 0.0812175648702595 | 0.0565274251497006 |
| 5561 | 0.115990153846154 | -0.111592934131737 | -0.309032834331337 | -0.0116953293413174 |
| 5562 | 0.0815686153846154 | -0.207104790419162 | -0.103958483033932 | -0.102171696606786 |
| 5563 | 0.0452695384615385 | -0.136607944111776 | 0.123044610778443 | 0.00129948103792415 |
| 5564 | 0.0400541538461538 | -0.0311875449101796 | -0.00771566866267465 | 0.121014171656687 |
| 5565 | 0.0463126153846154 | -0.0719587624750499 | -0.0134008982035928 | -0.0232282235528942 |
| 5566 | 0.0780221538461538 | -0.0115328942115768 | 0.118983732534930 | -0.117927904191617 |
| 5567 | 0.0886615384615385 | 0.0990854291417166 | 0.132384630738523 | 0.0730958083832335 |
| 5568 | 0.0832375384615385 | 0.0761820758483034 | 0.0856845309381238 | 0.100384910179641 |
| 5569 | 0.0917907692307692 | -0.0412585229540918 | 0.00527914171656687 | 0.0183551696606786 |
| 5570 | 0.109940307692308 | 0.00389844311377246 | -0.0377661676646707 | 0.0763445109780439 |
| 5571 | 0.133722461538462 | -0.128648622754491 | -0.0268017964071856 | 0.0562025548902196 |
| 5572 | 0.112235076923077 | -0.120039560878244 | -0.0629436127744511 | -0.0352484231536926 |
| 5573 | 0.0567433846153846 | 0.175592375249501 | -0.223348303393214 | 0.0511670658682635 |
| 5574 | -0.00479815384615385 | 0.112892415169661 | -0.181927345309381 | 0.119227385229541 |
| 5575 | -0.0181495384615385 | -0.0998976047904192 | 0.162435129740519 | -0.106395009980040 |
| 5576 | 0.0319181538461538 | 0.0453194011976048 | 0.0917758483033932 | -0.205642874251497 |
| 5577 | 0.0715550769230769 | 0.281500079840319 | -0.149034231536926 | 0.0646491816367266 |
| 5578 | 0.0723895384615385 | 0.0102334131736527 | -0.0369539920159681 | 0.118740079840319 |
| 5579 | 0.0400541538461538 | -0.0865779241516966 | 0.226190918163673 | -0.154638243512974 |
| 5580 | 0.0202356923076923 | 0.148628143712575 | 0.0938062874251497 | -0.0674105788423154 |
| 5581 | 0.0323353846153846 | 0.0534411576846307 | -0.150658582834331 | 0.185013612774451 |
| 5582 | 0.0644621538461539 | 0.382047425149701 | -0.0913697604790419 | -0.0573396007984032 |
| 5583 | 0.0811513846153846 | 0.211815409181637 | 0.0357357285429142 | -0.252749061876248 |
| 5584 | 0.0709292307692308 | -0.958854570858284 | -0.00609131736526946 | 0.0446696606786427 |
| 5585 | 0.0417230769230769 | -0.655750618762475 | -0.0292383233532934 | 0.0467813173652695 |
| 5586 | 0.00751015384615385 | 0.531162874251497 | 0.0568522954091816 | -0.281500079840319 |
| 5587 | 0.0250338461538462 | 0.351022315369262 | -0.0402026946107784 | -0.0987605588822355 |

| | | | | |
|---|---|---|---|---|
| 5588 | 0.0690516923076923 | -0.0831667864271457 | 0.0694410179640719 | 0.251287145708583 |
| 5589 | 0.0911649230769231 | 0.0713090219560878 | 0.0869027944111777 | 0.0349235528942116 |
| 5590 | 0.0746843076923077 | 0.0932377644710579 | 0.101521956087824 | -0.165846267465070 |
| 5591 | 0.0463126153846154 | 0.558127105788423 | -0.242028343313373 | 0.172181237524950 |
| 5592 | 0.0371335384615385 | 0.433376926147705 | 0.285885828343313 | 0.208404271457086 |
| 5593 | 0.0348387692307692 | -0.924093453093812 | 0.573396007984032 | -0.141643433133733 |
| 5594 | 0.0350473846153846 | -0.636420838323353 | -0.795119960079840 | 0.0781312974051896 |
| 5595 | 0.0116824615384615 | 0.925392934131737 | -1.39085079840319 | 0.394392495009980 |
| 5596 | -0.0312923076923077 | 0.544969860279441 | 0.626593512974052 | -0.0151064670658683 |
| 5597 | -0.0663396923076923 | -0.626674730538922 | 1.92851107784431 | -0.249013053892216 |
| 5598 | -0.0700947692307692 | 0.0232282235528942 | -1.68445229540918 | 0.269155009980040 |
| 5599 | -0.0229476923076923 | 0.740379321357286 | -1.33927764471058 | 0.364504431137725 |
| 5600 | 0.0181495384615385 | 0.0147815968063872 | 2.37561377245509 | -0.180465429141717 |
| 5601 | 0.0385938461538462 | -0.343225429141717 | 1.17724860279441 | -0.221074211576846 |
| 5602 | 0.0475643076923077 | -0.102009261477046 | -2.20586906187625 | 0.233256846307385 |
| 5603 | 0.0262855384615385 | -0.451407225548902 | -0.713496307385230 | 0.254210978043912 |
| 5604 | 0.0210701538461538 | -0.367103393213573 | 2.38982684630739 | -0.0893393213572854 |
| 5605 | 0.0294147692307692 | 0.407062435129741 | -0.390656487025948 | -0.130272974051896 |
| 5606 | 0.0135600000000000 | -0.0284261477045908 | -1.24181656686627 | 0.0183551696606786 |
| 5607 | -0.0369249230769231 | -0.466351257485030 | 0.499894111776447 | 0.0878774051896208 |
| 5608 | -0.113486769230769 | 0.413722275449102 | 0.315530239520958 | 0.0180302994011976 |
| 5609 | -0.141649846153846 | 0.389032135728543 | -0.458067065868263 | -0.185176047904192 |
| 5610 | -0.103056000000000 | -0.472036487025948 | 0.529944610778443 | -0.237967465069860 |
| 5611 | -0.0342129230769231 | -0.165358962075848 | 0.566492514970060 | 0.0763445109780439 |
| 5612 | 0.0183581538461538 | 0.453518882235529 | -1.45298223552894 | 0.260870818363273 |
| 5613 | 0.0235735384615385 | -0.0682227544910180 | 0.383752994011976 | -0.0846287025948104 |
| 5614 | 0.0110566153846154 | -0.447508782435130 | 1.53582415169661 | -0.233256846307385 |
| 5615 | 0.000208615384615385 | 0.251449580838323 | -0.686288423153693 | 0.188262315369261 |
| 5616 | 0.0198184615384615 | 0.534736447105789 | -1.72790369261477 | 0.324382954091816 |
| 5617 | 0.0358818461538462 | -0.0133196806387226 | 0.733394610778443 | -0.124750179640719 |
| 5618 | 0.00438092307692308 | -0.125075049900200 | 1.69744710578842 | -0.211977844311377 |
| 5619 | -0.0490246153846154 | 0.255185588822355 | -1.17440598802395 | 0.147003792415170 |
| 5620 | -0.0961716923076923 | 0.0620502195608782 | -1.13907634730539 | 0.108506666666667 |
| 5621 | -0.0967975384615385 | -0.268342834331337 | 1.06232574850299 | -0.155450419161677 |
| 5622 | -0.0732240000000000 | -0.250962275449102 | 1.14679201596806 | -0.0726085029940120 |
| 5623 | -0.0433920000000000 | -0.285885828343313 | -0.676948403193613 | 0.0862530538922156 |
| 5624 | -0.0179409230769231 | -0.0352484231536926 | -0.495833233532934 | 0.00519792415169661 |
| 5625 | -0.0189840000000000 | 0.256160199600798 | 0.782531237524950 | 0.00763445109780439 |
| 5626 | -0.00542400000000000 | -0.0384971257485030 | 0.0134008982035928 | 0.113704590818363 |
| 5627 | 0.0154375384615385 | -0.406575129740519 | -0.436950499001996 | -0.0730958083832335 |
| 5628 | 0.0310836923076923 | 0.0648116167664671 | -0.172587325349301 | -0.234393892215569 |
| 5629 | 0.0289975384615385 | 0.393417884231537 | 0.0446696606786427 | -0.0164059481037924 |
| 5630 | -0.0160633846153846 | -0.0532787225548902 | -0.319591117764471 | 0.0648116167664671 |
| 5631 | -0.0650880000000000 | -0.108506666666667 | 0.0560401197604790 | -0.101359520958084 |

| | | | | |
|---|---|---|---|---|
| 5632 | -0.0932510769230769 | 0.0644867465069860 | 0.379692115768463 | -0.0396341716566866 |
| 5633 | -0.0748929230769231 | -0.0298880638722555 | -0.401214770459082 | 0.0929128942115768 |
| 5634 | -0.0212787692307692 | -0.00617253493013972 | 0.0925880239520958 | -0.0537660279441118 |
| 5635 | 0.0250338461538462 | 0.113704590818363 | 0.601009980039920 | -0.160485908183633 |
| 5636 | 0.0496504615384615 | -0.0540908982035928 | 0.110455888223553 | 0.0672481437125748 |
| 5637 | 0.0504849230769231 | -0.0618877844311377 | -0.530350698602794 | 0.186150658682635 |
| 5638 | 0.0458953846153846 | 0.298230898203593 | -0.123044610778443 | -0.0175429940119760 |
| 5639 | 0.0216960000000000 | 0.235368502994012 | 0.307408483033932 | -0.0240403992015968 |
| 5640 | -0.00333784615384615 | -0.236992854291417 | -0.203856087824351 | 0.196708942115768 |
| 5641 | -0.0540313846153846 | -0.0220911776447106 | -0.421519161676647 | 0.0693598003992016 |
| 5642 | -0.124334769230769 | 0.352159361277445 | 0.0682227544910180 | -0.231307624750499 |
| 5643 | -0.139146461538462 | -0.238617205588822 | 0.546188123752495 | -0.0726085029940120 |
| 5644 | -0.0993009230769231 | -0.384321516966068 | -0.0605070858283433 | 0.103796047904192 |
| 5645 | -0.0150203076923077 | 0.222373692614770 | -0.301317165668663 | -0.0873900998003992 |
| 5646 | 0.0394283076923077 | 0.0100709780439122 | 0.216444810379242 | -0.152689021956088 |
| 5647 | 0.0465212307692308 | -0.383671776447106 | 0.409742614770459 | -0.00438574850299401 |
| 5648 | 0.0473556923076923 | 0.135308463073852 | -0.207104790419162 | 0.0711465868263473 |
| 5649 | 0.0463126153846154 | 0.383509341317365 | -0.512482834331337 | -0.0552279441117765 |
| 5650 | 0.0557003076923077 | -0.139044471057884 | 0.0869027944111777 | -0.0443447904191617 |
| 5651 | 0.0396369230769231 | -0.191673453093812 | 0.142942914171657 | 0.152201716566866 |
| 5652 | 0.00396369230769231 | 0.0594512574850299 | -0.194109980039920 | 0.0440199201596806 |
| 5653 | -0.0536141538461538 | -0.166171137724551 | -0.153907285429142 | -0.189561796407186 |
| 5654 | -0.108062769230769 | -0.124425309381238 | 0.159186427145709 | -0.0513295009980040 |
| 5655 | -0.0940855384615385 | 0.352646666666667 | 0.196140419161677 | 0.183226826347305 |
| 5656 | -0.0388024615384615 | 0.0451569660678643 | 0.216850898203593 | 0.0313499800399202 |
| 5657 | 0.0348387692307692 | -0.00942123752495010 | 0.0328931137724551 | -0.118090339321357 |
| 5658 | 0.0665483076923077 | 0.507934650698603 | -0.233500499001996 | 0.0177054291417166 |
| 5659 | 0.0604984615384615 | -0.253398802395210 | 0.0414209580838323 | 0.0807302594810379 |
| 5660 | 0.0521538461538462 | -0.786673333333333 | 0.205886526946108 | -0.0350859880239521 |
| 5661 | 0.0342129230769231 | 0.0792683433133733 | -0.246901397205589 | -0.107694491017964 |
| 5662 | 0.0463126153846154 | 0.580543153692615 | -0.599791716566866 | 0.00422331337325349 |
| 5663 | 0.0196098461538462 | -0.0194922155688623 | -0.123450698602794 | 0.0799180838323353 |
| 5664 | -0.0404713846153846 | -0.151714411177645 | 0.234718762475050 | -0.00438574850299401 |
| 5665 | -0.0924166153846154 | 0.321946427145709 | -0.0194922155688623 | 0.00536035928143713 |
| 5666 | -0.126629538461538 | 0.0823546107784431 | -0.152282934131737 | 0.168932534930140 |
| 5667 | -0.0884529230769231 | -0.214414371257485 | 0.214008283433134 | 0.0919382834331337 |
| 5668 | -0.0342129230769231 | -0.313662235528942 | 0.435732235528942 | -0.0536035928143713 |
| 5669 | 0.0398455384615385 | -0.317560678642715 | 0.108019361277445 | 0.102009261477046 |
| 5670 | 0.0940855384615385 | 0.225459960079840 | -0.245683133732535 | 0.144079960079840 |
| 5671 | 0.103890461538462 | 0.607669820359282 | -0.0150252495009980 | -0.0186800399201597 |
| 5672 | 0.129758769230769 | 0.0362230339321357 | 0.250556187624750 | -0.0123450698602794 |
| 5673 | 0.128924307692308 | -0.289621836327345 | -0.230657884231537 | 0.125399920159681 |
| 5674 | 0.120579692307692 | 0.353458842315369 | -0.270860578842315 | 0.0693598003992016 |
| 5675 | 0.0728067692307692 | 0.497701237524950 | 0.260302295409182 | -0.0414209580838323 |

| | | | | |
|---|---|---|---|---|
| 5676 | -0.0112652307692308 | -0.172018802395210 | 0.272891017964072 | 0.0253398802395210 |
| 5677 | -0.0615415384615385 | -0.397803632734531 | -0.0552279441117765 | 0.0602634331337325 |
| 5678 | -0.0642535384615385 | -0.00113704590818363 | -0.0121826347305389 | -0.0830043512974052 |
| 5679 | -0.00625846153846154 | 0.271266666666667 | 0.278982335329341 | -0.124262874251497 |
| 5680 | 0.0371335384615385 | 0.217338203592814 | 0.0917758483033932 | 0.0825170459081836 |
| 5681 | 0.0492332307692308 | -0.138882035928144 | -0.0885271457085828 | 0.0524665469061876 |
| 5682 | 0.0431833846153846 | -0.367753133732535 | -0.105176746506986 | -0.169419840319361 |
| 5683 | 0.0304578461538462 | 0.0773191217564870 | -0.194516067864271 | -0.0843038323353293 |
| 5684 | 0.0465212307692308 | 0.267368223552894 | 0.0190861277445110 | 0.0953494211576846 |
| 5685 | 0.0552830769230769 | -0.317885548902196 | 0.114110678642715 | -0.0199795209580838 |
| 5686 | 0.0488160000000000 | -0.472848662674651 | -0.169338622754491 | -0.134496287425150 |
| 5687 | 0.00292061538461538 | 0.122151217564870 | -0.147003792415170 | 0.0233906586826347 |
| 5688 | -0.0575778461538462 | 0.287672614770459 | 0.478777544910180 | 0.0937250698602794 |
| 5689 | -0.0663396923076923 | -0.0976235129740519 | 0.396747804391218 | -0.00113704590818363 |
| 5690 | -0.0166892307692308 | 0.00909636726546906 | -0.197358682634731 | 0.00828419161676647 |
| 5691 | 0.0819858461538462 | 0.110293453093812 | -0.0974610778443114 | 0.113704590818363 |
| 5692 | 0.139772307692308 | -0.0631872654690619 | 0.252586626746507 | 0.0847911377245509 |
| 5693 | 0.129758769230769 | 0.146516487025948 | 0.145785528942116 | -0.0420706986027944 |
| 5694 | 0.0890787692307692 | 0.288484790419162 | -0.362230339321357 | -0.0644867465069860 |
| 5695 | 0.0458953846153846 | 0.0566898602794411 | -0.245683133732535 | -0.00714714570858283 |
| 5696 | 0.0444350769230769 | -0.0977859481037924 | 0.0763445109780439 | -0.0456442714570858 |
| 5697 | 0.0315009230769231 | 0.0857657485029940 | -0.0288322355289421 | -0.116953293413174 |
| 5698 | -0.0112652307692308 | 0.112080239520958 | -0.0962428143712575 | 0.000649740518962076 |
| 5699 | -0.0579950769230769 | -0.0552279441117765 | -0.0308626746506986 | 0.0550655089820359 |
| 5700 | -0.0919993846153846 | -0.0363854690618763 | 0.137663772455090 | -0.0229033532934132 |
| 5701 | -0.0728067692307692 | -0.0935626347305389 | -0.00365479041916168 | 0.0328118962075848 |
| 5702 | -0.0306664615384615 | -0.255510459081836 | 0.0296444111776447 | 0.0396341716566866 |
| 5703 | 0.0168978461538462 | -0.0927504590818363 | 0.0755323353293413 | -0.0614004790419162 |
| 5704 | 0.0427661538461539 | 0.134983592814371 | -0.176242115768463 | 0.0225784830339321 |
| 5705 | 0.0302492307692308 | -0.0839789620758483 | 0.438574850299401 | 0.0958367265469062 |
| 5706 | 0.0348387692307692 | -0.162922435129741 | 0.289134530938124 | -0.0722836327345309 |
| 5707 | 0.0479815384615385 | 0.0703344111776447 | -1.05339181636727 | -0.102983872255489 |
| 5708 | 0.0665483076923077 | -0.0362230339321357 | -1.13014241516966 | 0.156100159680639 |
| 5709 | 0.0596640000000000 | -0.188749620758483 | 1.68485838323353 | 0.225784830339321 |
| 5710 | 0.00667569230769231 | 0.0193297804391218 | 1.33440459081836 | -0.127024271457086 |
| 5711 | -0.0392196923076923 | 0.0797556487025948 | -2.50312534930140 | -0.221561516966068 |
| 5712 | -0.0544486153846154 | 0.0255023153692615 | -0.290352794411178 | 0.186800399201597 |
| 5713 | -0.00688430769230769 | 0.225947265469062 | 2.75489980039920 | 0.194272415169661 |
| 5714 | 0.0588295384615385 | 0.259246467065868 | 0.0276139720558882 | -0.232769540918164 |
| 5715 | 0.102012923076923 | -0.00373600798403194 | -2.81865558882236 | -0.146191616766467 |
| 5716 | 0.122248615384615 | -0.0415833932135729 | 0.252586626746507 | 0.169257405189621 |
| 5717 | 0.101178461538462 | 0.0818673053892216 | 1.99673383233533 | 0.0139694211576846 |
| 5718 | 0.0957544615384615 | -0.0898266267465070 | -0.981108183632735 | -0.153663632734531 |
| 5719 | 0.0986750769230769 | -0.178028902195609 | -0.689943213572854 | 0.0384971257485030 |

| | | | | |
|---|---|---|---|---|
| 5720 | 0.0725981538461538 | 0.0657862275449102 | 0.629436127744511 | 0.0726085029940120 |
| 5721 | 0.0300406153846154 | 0.0633497005988024 | 0.0129948103792415 | -0.216038722554890 |
| 5722 | -0.0448523076923077 | -0.172668542914172 | -0.208323053892216 | -0.0451569660678643 |
| 5723 | -0.0859495384615385 | -0.0501924550898204 | 0.940905489021956 | 0.297418722554890 |
| 5724 | -0.0534055384615385 | 0.121988782435130 | -0.343550299401198 | -0.0519792415169661 |
| 5725 | 0.00292061538461538 | -0.0622126546906188 | -1.50902235528942 | -0.315124151696607 |
| 5726 | 0.0663396923076923 | -0.172830978043912 | 0.925068063872256 | 0.0984356886227545 |
| 5727 | 0.0970061538461539 | -0.128648622754491 | 1.07044750499002 | 0.261845429141717 |
| 5728 | 0.104933538461538 | -0.154150938123753 | -1.17075119760479 | -0.168607664670659 |
| 5729 | 0.114738461538462 | 0.0670857085828343 | -1.36445508982036 | -0.174455329341317 |
| 5730 | 0.112235076923077 | 0.265906307385230 | 1.31856716566866 | 0.246738962075848 |
| 5731 | 0.0892873846153846 | 0.0329743313373254 | 1.32547065868263 | 0.141805868263473 |
| 5732 | 0.0258683076923077 | -0.0833292215568862 | -1.31085149700599 | -0.161135648702595 |
| 5733 | -0.0448523076923077 | -0.0573396007984032 | -0.807302594810379 | 0.0696846706586826 |
| 5734 | -0.0980492307692308 | 0.325195129740519 | 1.20973562874252 | 0.221561516966068 |
| 5735 | -0.102430153846154 | 0.498838283433134 | 0.848723552894212 | -0.0847911377245509 |
| 5736 | -0.0392196923076923 | 0.000649740518962076 | -0.915321956087824 | -0.0305378043912176 |
| 5737 | 0.0214873846153846 | 0.0227409181636727 | -0.298880638722555 | 0.217175768463074 |
| 5738 | 0.0452695384615385 | 0.0393093013972056 | 0.802023453093812 | -0.0170556886227545 |
| 5739 | 0.0146030769230769 | -0.114516766467066 | 0.0166496007984032 | -0.246738962075848 |
| 5740 | -0.0277458461538462 | 0.184526307385230 | -0.487711477045908 | 0.0961615968063872 |
| 5741 | -0.0312923076923077 | -0.129460798403194 | 0.223348303393214 | 0.276464590818363 |
| 5742 | -0.0189840000000000 | -0.761333453093812 | 0.445478343313373 | -0.191511017964072 |
| 5743 | -0.0131427692307692 | -0.0665984031936128 | -0.299286726546906 | -0.226921876247505 |
| 5744 | -0.0498590769230769 | 0.692136087824351 | 0.0592888223552894 | 0.279713293413174 |
| 5745 | -0.100761230769231 | -0.0843038323353293 | 0.397966067864271 | 0.166496007984032 |
| 5746 | -0.111609230769231 | -0.467488303393214 | -0.212790019960080 | -0.315936327345309 |
| 5747 | -0.0742670769230769 | 0.218800119760479 | -0.250150099800399 | -0.162272694610778 |
| 5748 | -0.0241993846153846 | 0.183714131736527 | 0.255429241516966 | 0.179003512974052 |
| 5749 | -0.000625846153846154 | -0.268992574850299 | 0.0527914171656687 | -0.0181927345309381 |
| 5750 | 0.00876184615384615 | -0.100384910179641 | -0.609537824351297 | -0.231307624750499 |
| 5751 | 0.0100135384615385 | -0.0878774051896208 | -0.182739520958084 | 0.0154313373253493 |
| 5752 | 0.0235735384615385 | -0.272566147704591 | 0.385377345309381 | 0.189074491017964 |
| 5753 | 0.0469384615384615 | 0.242840518962076 | 0.0613192614770459 | -0.0196546506986028 |
| 5754 | 0.0285803076923077 | 0.624075768463074 | -0.380910379241517 | -0.0685476247504990 |
| 5755 | -0.0194012307692308 | 0.0895017564870260 | 0.276951896207585 | 0.131410019960080 |
| 5756 | -0.0840720000000000 | -0.143592654690619 | 0.578675149700599 | 0.0984356886227545 |
| 5757 | -0.133096615384615 | 0.291246187624751 | -0.287916267465070 | -0.112892415169661 |
| 5758 | -0.122457230769231 | 0.174130459081836 | -0.434513972055888 | -0.0857657485029940 |
| 5759 | -0.0734326153846154 | -0.293682714570858 | 0.224160479041916 | 0.0388219960079840 |
| 5760 | -0.0166892307692308 | -0.135470898203593 | 0.420300898203593 | 0.00877149700598802 |
| 5761 | -0.00312923076923077 | 0.153663632734531 | -0.512076746506986 | -0.0367103393213573 |
| 5762 | -0.0221132307692308 | -0.0779688622754491 | -0.409742614770459 | 0.00958367265469062 |
| 5763 | -0.0179409230769231 | -0.182414650698603 | 0.549436826347305 | 0.0417458283433134 |

| | | | | |
|---|---|---|---|---|
| 5764 | 0.00166892307692308 | 0.125887225548902 | 0.444260079840319 | -0.00828419161676647 |
| 5765 | 0.0210701538461538 | 0.136445508982036 | -0.251774451097804 | 0.0607507385229541 |
| 5766 | 0.0139772307692308 | -0.129785668662675 | -0.224566566866267 | 0.139206906187625 |
| 5767 | -0.0340043076923077 | -0.183064391217565 | 0.518980239520958 | 0.0740704191616766 |
| 5768 | -0.0894960000000000 | 0.0190049101796407 | 0.201013473053892 | -0.0113704590818363 |
| 5769 | -0.111400615384615 | 0.129785668662675 | -0.292383233532934 | 0.0417458283433134 |
| 5770 | -0.0776049230769231 | 0.0246901397205589 | -0.196140419161677 | 0.0695222355289421 |
| 5771 | -0.0187753846153846 | -0.0510046307385230 | 0.0272078842315369 | -0.0791059081836327 |
| 5772 | 0.0377593846153846 | 0.123775568862275 | 0.0824358283433134 | -0.0998976047904192 |
| 5773 | 0.0625846153846154 | 0.244627305389222 | -0.00243652694610778 | 0.0680603193612775 |
| 5774 | 0.0611243076923077 | -0.0303753692614770 | -0.0117765469061876 | 0.110131017964072 |
| 5775 | 0.0815686153846154 | -0.126699401197605 | -0.239185728542914 | -0.0661110978043912 |
| 5776 | 0.0899132307692308 | 0.0555528143712575 | 0.0268017964071856 | -0.0851160079840319 |
| 5777 | 0.0878270769230769 | 0.0828419161676647 | 0.313093712574850 | 0.0685476247504990 |
| 5778 | 0.0425575384615385 | 0.0441823552894212 | -0.0438574850299401 | 0.123125828343313 |
| 5779 | -0.0304578461538462 | 0.104445788423154 | -0.379692115768463 | -0.0461315768463074 |
| 5780 | -0.0440178461538462 | 0.0717963273453094 | -0.0198983033932136 | -0.107856926147705 |
| 5781 | -0.0294147692307692 | -0.117440598802395 | 0.366697305389222 | 0.000162435129740519 |
| 5782 | 0.0227390769230769 | -0.167795489021956 | -0.204668263473054 | -0.0877149700598802 |
| 5783 | 0.0648793846153846 | -0.0294007584830339 | -0.531568962075848 | -0.0844662674650699 |
| 5784 | 0.0782307692307692 | -0.0458067065868264 | 0.134415069860279 | 0.0168932534930140 |
| 5785 | 0.0700947692307692 | -0.196384071856287 | 0.333398103792415 | -0.0423955688622755 |
| 5786 | 0.0502763076923077 | -0.146678922155689 | -0.306190219560878 | -0.0776439920159681 |
| 5787 | 0.0815686153846154 | -0.0397966067864271 | -0.216444810379242 | 0.102983872255489 |
| 5788 | 0.0899132307692308 | 0.112242674650699 | 0.449539221556886 | 0.142130738522954 |
| 5789 | 0.0652966153846154 | 0.143592654690619 | 0.249337924151697 | -0.0985981237524950 |
| 5790 | 0.00458953846153846 | 0.0656237924151697 | -0.446696606786427 | -0.126374530938124 |
| 5791 | -0.0688430769230769 | 0.0578269061876247 | -0.172181237524950 | 0.130760279441118 |
| 5792 | -0.0765618461538462 | -0.127674011976048 | 0.435732235528942 | 0.0914509780439122 |
| 5793 | -0.0400541538461538 | -0.0755323353293413 | 0.0710653692614771 | -0.207104790419162 |
| 5794 | 0.0229476923076923 | 0.142618043912176 | -0.406493912175649 | -0.0344362475049900 |
| 5795 | 0.0456867692307692 | -0.0435326147704591 | -0.0462940119760479 | 0.183714131736527 |
| 5796 | 0.0279544615384615 | -0.197033812375250 | 0.428422654690619 | -0.0536035928143713 |
| 5797 | 0.0104307692307692 | 0.181764910179641 | 0.174617764471058 | -0.111268063872255 |
| 5798 | 0.00897046153846154 | 0.299530379241517 | -0.0994915169660679 | 0.191835888223553 |
| 5799 | 0.0277458461538462 | -0.119877125748503 | -0.0767505988023952 | 0.151714411177645 |
| 5800 | 0.0118910769230769 | -0.157562075848303 | 0.128729840319361 | -0.157237205588822 |
| 5801 | -0.0221132307692308 | 0.116465988023952 | 0.280606686626747 | -0.0220911776447106 |
| 5802 | -0.0717636923076923 | 0.0985981237524950 | -0.0454818363273453 | 0.199957644710579 |
| 5803 | -0.0974233846153846 | -0.0662735329341317 | -0.402839121756487 | -0.00389844311377246 |
| 5804 | -0.0663396923076923 | 0.00942123752495010 | -0.348829441117764 | -0.102334131736527 |
| 5805 | -0.0210701538461538 | -0.121663912175649 | 0.198983033932136 | 0.143105349301397 |
| 5806 | 0.0252424615384615 | -0.362555209580838 | 0.222536127744511 | 0.0633497005988024 |
| 5807 | 0.0367163076923077 | -0.480645548902196 | -0.319185029940120 | -0.119389820359281 |

| | | | | |
|---|---|---|---|---|
| 5808 | 0.0319181538461538 | -0.0160810778443114 | -0.173399500998004 | 0.0428828742514970 |
| 5809 | 0.0317095384615385 | 0.882834930139721 | 0.252992714570858 | 0.0919382834331337 |
| 5810 | 0.0431833846153846 | 0.724135808383234 | 0.248931836327345 | -0.115653812375249 |
| 5811 | 0.0611243076923077 | 0.220586906187625 | -0.166496007984032 | -0.0563649900199601 |
| 5812 | 0.0306664615384615 | -0.186962834331337 | -0.204668263473054 | 0.158699121756487 |
| 5813 | -0.0227390769230769 | -0.523853293413174 | -0.0231470059880240 | -0.0968113373253493 |
| 5814 | -0.0715550769230769 | -0.203043912175649 | -0.0284261477045908 | -0.236505548902196 |
| 5815 | -0.0834461538461538 | 0.163247305389222 | 0.0799993013972056 | 0.127998882235529 |
| 5816 | -0.0444350769230769 | -0.412747664670659 | -0.0154313373253493 | 0.193947544910180 |
| 5817 | -0.00751015384615385 | -0.882185189620759 | 0.142942914171657 | -0.200932255489022 |
| 5818 | 0.00625846153846154 | 0.690186866267465 | 0.575426447105788 | -0.0807302594810379 |
| 5819 | -0.0173150769230769 | 1.29428311377246 | 0.153095109780439 | 0.282637125748503 |
| 5820 | -0.0292061538461538 | -0.535223752495010 | -1.29054710578842 | -0.0488929740518962 |
| 5821 | 0.00604984615384615 | -0.726409900199601 | 0.0531975049900200 | -0.240566427145709 |
| 5822 | 0.0502763076923077 | 0.653314091816367 | 2.03409391217565 | 0.162272694610778 |
| 5823 | 0.0696775384615385 | 0.192972934131737 | -0.964052495009980 | 0.201094690618762 |
| 5824 | 0.0396369230769231 | -0.926692415169661 | -2.29683273453094 | -0.319347465069860 |
| 5825 | 0.00604984615384615 | -0.279550858283433 | 1.82292824351297 | -0.150252495009980 |
| 5826 | -0.0221132307692308 | 0.249175489021956 | 2.08607315369261 | 0.399915289421158 |
| 5827 | -0.0104307692307692 | -0.0441823552894212 | -2.50759231536926 | -0.0133196806387226 |
| 5828 | 0.0536141538461538 | 0.375387584830339 | -1.26374530938124 | -0.414047145708583 |
| 5829 | 0.0736412307692308 | 0.531650179640719 | 2.54860718562874 | 0.150252495009980 |
| 5830 | 0.0705120000000000 | -0.266393612774451 | 0.516137624750499 | 0.422006467065868 |
| 5831 | 0.0500676923076923 | -0.0656237924151697 | -1.56506247504990 | -0.258109421157685 |
| 5832 | 0.0477729230769231 | 0.668420558882236 | 0.574614271457086 | -0.325520000000000 |
| 5833 | 0.0613329230769231 | -0.183714131736527 | 0.921413273453094 | 0.334291497005988 |
| 5834 | 0.0333784615384615 | -0.774328263473054 | -0.968925548902196 | 0.199632774451098 |
| 5835 | 0.0129341538461538 | 0.102496566866267 | 0.490960179640719 | -0.278738682634731 |
| 5836 | -0.0273286153846154 | 0.465863952095808 | 0.852784431137725 | -0.0151064670658683 |
| 5837 | -0.0458953846153846 | -0.291083752495010 | -1.56749900199601 | 0.239104510978044 |
| 5838 | -0.0189840000000000 | -0.323570778443114 | -0.524665469061876 | -0.0974610778443114 |
| 5839 | 0.0300406153846154 | 0.454818363273453 | 1.86719181636727 | -0.0664359680638723 |
| 5840 | 0.0792738461538462 | 0.180952734530938 | 0.0276139720558882 | 0.262982475049900 |
| 5841 | 0.0584123076923077 | -0.150577365269461 | -2.11937235528942 | 0.0178678642714571 |
| 5842 | 0.0308750769230769 | 0.153176327345309 | 0.423955688622755 | -0.255185588822355 |
| 5843 | 0.0154375384615385 | 0.00877149700598802 | 1.97845988023952 | 0.189561796407186 |
| 5844 | 0.0277458461538462 | -0.147653532934132 | -0.666796207584830 | 0.326007305389222 |
| 5845 | 0.0815686153846154 | 0.0763445109780439 | -1.68445229540918 | -0.283774171656687 |
| 5846 | 0.0788566153846154 | 0.0539284630738523 | 0.653801397205589 | -0.299043073852295 |
| 5847 | 0.0529883076923077 | -0.000812175648702595 | 1.08466057884232 | 0.219937165668663 |
| 5848 | 0.0114738461538462 | 0.252911497005988 | -0.864560978043912 | 0.105745269461078 |
| 5849 | -0.0229476923076923 | 0.0428828742514970 | -0.572177744510978 | -0.320809381237525 |
| 5850 | 0.000208615384615385 | -0.261033253493014 | 0.761008582834331 | -0.126699401197605 |
| 5851 | 0.00458953846153846 | 0.0917758483033932 | 0.274109281437126 | 0.233581716566866 |

| | | | | |
|---|---|---|---|---|
| 5852 | 0.0152289230769231 | 0.334453932135729 | -0.418676546906188 | 0.0657862275449102 |
| 5853 | 0.00417230769230769 | -0.0914509780439122 | 0.116141117764471 | -0.158699121756487 |
| 5854 | -0.00646707692307692 | -0.354271017964072 | 0.389844311377246 | 0.0261520558882236 |
| 5855 | 0.0216960000000000 | -0.0441823552894212 | -0.214008283433134 | 0.105095528942116 |
| 5856 | 0.0431833846153846 | 0.00877149700598802 | -0.122638522954092 | -0.113054850299401 |
| 5857 | 0.0763532307692308 | -0.182252215568862 | 0.127917664670659 | -0.0500300199600798 |
| 5858 | 0.0569520000000000 | 0.0177054291417166 | -0.252586626746507 | 0.124425309381238 |
| 5859 | 0.0413058461538462 | 0.173155848303393 | -0.111268063872255 | 0.0108831536926148 |
| 5860 | 0.0506935384615385 | -0.123288263473054 | 0.297662375249501 | -0.0534411576846307 |
| 5861 | 0.0611243076923077 | -0.0703344111776447 | 0.0515731536926148 | 0.103633612774451 |
| 5862 | 0.0924166153846154 | 0.172993413173653 | -0.300911077844311 | 0.132547065868263 |
| 5863 | 0.0771876923076923 | -0.0838165269461078 | 0.00324870259481038 | -0.0149440319361277 |
| 5864 | 0.0546572307692308 | -0.200769820359281 | 0.412991317365269 | -0.00601009980039920 |
| 5865 | 0.0181495384615385 | 0.150577365269461 | 0.0747201596806387 | 0.0935626347305389 |
| 5866 | -0.000834461538461538 | 0.223998043912176 | -0.420300898203593 | 0.0383346906187625 |
| 5867 | 0.0483987692307692 | -0.170394451097804 | -0.101521956087824 | 0.0373600798403194 |
| 5868 | 0.0919993846153846 | -0.0885271457085828 | 0.497863672654691 | 0.0888520159680639 |
| 5869 | 0.100969846153846 | 0.204830698602794 | 0.0601009980039920 | -0.0661110978043912 |
| 5870 | 0.0546572307692308 | -0.0217663073852295 | -0.646085728542914 | -0.108831536926148 |
| 5871 | 0.0181495384615385 | -0.197521117764471 | -0.171369061876248 | 0.0893393213572854 |
| 5872 | 0.0194012307692308 | 0.0717963273453094 | 0.496645409181637 | 0.0802429540918164 |
| 5873 | 0.0331698461538462 | 0.201744431137725 | 0.0747201596806387 | -0.151064670658683 |
| 5874 | 0.0627932307692308 | -0.221723952095808 | -0.507609780439122 | -0.110780758483034 |
| 5875 | 0.0538227692307692 | -0.243977564870259 | -0.0308626746506986 | 0.115978682634731 |
| 5876 | 0.0306664615384615 | 0.238779640718563 | 0.451163572854291 | 0.0102334131736527 |
| 5877 | 0.0237821538461538 | 0.150252495009980 | 0.0637557884231537 | -0.0706592814371258 |
| 5878 | 0.0400541538461538 | -0.177866467065868 | -0.245683133732535 | 0.124262874251497 |
| 5879 | 0.0732240000000000 | -0.0129948103792415 | -0.0799993013972056 | 0.0469437524950100 |
| 5880 | 0.0794824615384615 | 0.0172181237524950 | 0.0812175648702595 | -0.174130459081836 |
| 5881 | 0.0561175384615385 | -0.169907145708583 | 0.131978542914172 | -0.0251774451097804 |
| 5882 | -0.00646707692307692 | -0.151389540918164 | -0.0422331337325349 | 0.124100439121756 |
| 5883 | -0.0458953846153846 | 0.136932814371257 | -0.210353493013972 | -0.0976235129740519 |
| 5884 | -0.0271200000000000 | 0.553416487025948 | 0.0690349301397206 | -0.203531217564870 |
| 5885 | 0.00479815384615385 | 0.205318003992016 | 0.350047704590818 | 0.0155937724550898 |
| 5886 | 0.0300406153846154 | -0.0401214770459082 | 0.0592888223552894 | 0.108506666666667 |
| 5887 | 0.0100135384615385 | 0.0984356886227545 | -0.354108582834331 | -0.141156127744511 |
| 5888 | -0.00604984615384615 | -0.370676966067864 | -0.0795932135728543 | -0.201094690618762 |
| 5889 | -0.00208615384615385 | -0.262332734530938 | 0.201825648702595 | 0.0588015169660679 |
| 5890 | 0.00959630769230769 | 0.312525189620759 | -0.0462940119760479 | 0.0370352095808383 |
| 5891 | 0.0348387692307692 | -0.0646491816367266 | -0.259084031936128 | -0.130760279441118 |
| 5892 | 0.000417230769230769 | -0.211815409181637 | -0.0844662674650699 | -0.00373600798403194 |
| 5893 | -0.0536141538461538 | 0.621151936127745 | 0.250556187624750 | 0.126699401197605 |
| 5894 | -0.0874098461538462 | 0.419732375249501 | 0.240810079840319 | -0.0264769261477046 |
| 5895 | -0.0857409230769231 | -0.542695768463074 | -0.128729840319361 | -0.0518168063872256 |

| | | | | |
|---|---|---|---|---|
| 5896 | -0.0344215384615385 | -0.283124431137725 | -0.242840518962076 | 0.111917804391218 |
| 5897 | -0.0102221538461538 | 0.268505269461078 | 0.332585928143713 | 0.0349235528942116 |
| 5898 | -0.00751015384615385 | -0.154963113772455 | 0.382534730538922 | -0.119877125748503 |
| 5899 | -0.0287889230769231 | -0.421031856287425 | -0.229033532934132 | 0.000487305389221557 |
| 5900 | -0.0506935384615385 | 0.140506387225549 | -0.197358682634731 | 0.127511576846307 |
| 5901 | -0.0273286153846154 | 0.206130179640719 | 0.325276347305389 | 0.0467813173652695 |
| 5902 | -0.00542400000000000 | -0.0667608383233533 | 0.306596307385230 | 0.0302129341317365 |
| 5903 | -0.000834461538461538 | 0.285398522954092 | -0.367509481037924 | 0.0969737724550898 |
| 5904 | -0.0513193846153846 | 0.0693598003992016 | -0.287104091816367 | 0.100872215568862 |
| 5905 | -0.107436923076923 | -0.560401197604790 | 0.325276347305389 | 0.0199795209580838 |
| 5906 | -0.108480000000000 | 0.246089221556886 | 0.121014171656687 | 0.0118577644710579 |
| 5907 | -0.0707206153846154 | 0.826145069860280 | -0.300504990019960 | 0.0423955688622755 |
| 5908 | -0.0137686153846154 | -0.130272974051896 | -0.130354191616766 | -0.0956742914171657 |
| 5909 | 0.00292061538461538 | -0.362392774451098 | 0.149440319361277 | -0.0313499800399202 |
| 5910 | -0.0137686153846154 | 0.438412415169661 | 0.0158374251497006 | 0.195409461077844 |
| 5911 | -0.0494418461538462 | 0.413884710578842 | -0.229033532934132 | -0.0126699401197605 |
| 5912 | -0.0496504615384615 | -0.339002115768463 | -0.138475948103792 | -0.214251936127745 |
| 5913 | -0.00375507692307692 | -0.309114051896208 | 0.145785528942116 | 0.101846826347305 |
| 5914 | 0.0198184615384615 | 0.0422331337325349 | 0.118577644710579 | 0.279225988023952 |
| 5915 | 0.0100135384615385 | 0.00633497005988024 | -0.0690349301397206 | -0.0534411576846307 |
| 5916 | -0.0244080000000000 | 0.00909636726546906 | -0.0142130738522954 | -0.215876287425150 |
| 5917 | -0.0396369230769231 | 0.113217285429142 | 0.143349001996008 | 0.130435409181637 |
| 5918 | -0.0152289230769231 | -0.150252495009980 | 0.141724650698603 | 0.253073932135729 |
| 5919 | 0.0335870769230769 | -0.245601916167665 | -0.00649740518962076 | -0.129785668662675 |
| 5920 | 0.0690516923076923 | 0.111917804391218 | -0.0588827345309381 | -0.178841077844311 |
| 5921 | 0.0592467692307692 | -0.0510046307385230 | -0.0190861277445110 | 0.146678922155689 |
| 5922 | 0.0335870769230769 | -0.380910379241517 | 0.00609131736526946 | 0.0383346906187625 |
| 5923 | 0.0342129230769231 | -0.247226267465070 | -0.0881210578842315 | -0.198170858283433 |
| 5924 | 0.0519452307692308 | 0.117927904191617 | -0.0929941117764471 | -0.0789434730538922 |
| 5925 | 0.0602898461538462 | 0.0493802794411178 | -0.169744710578842 | 0.0402839121756487 |
| 5926 | 0.0421403076923077 | 0.0401214770459082 | -0.000406087824351297 | 0.0162435129740519 |
| 5927 | -0.00500676923076923 | 0.260058642714571 | 0.122232435129741 | 0.0519792415169661 |
| 5928 | -0.0575778461538462 | 0.0843038323353293 | 0.0572583832335329 | 0.119714690618762 |
| 5929 | -0.0786480000000000 | -0.101846826347305 | 0.00771566866267465 | 0.0217663073852295 |
| 5930 | -0.0408886153846154 | -0.0284261477045908 | 0.0154313373253493 | -0.0622126546906188 |
| 5931 | 0.0177323076923077 | 0.0112080239520958 | 0.584766467065868 | 0.0576644710578842 |
| 5932 | 0.0667569230769231 | -0.0497051497005988 | -0.0592888223552894 | 0.0761820758483034 |
| 5933 | 0.0627932307692308 | 0.110293453093812 | -0.553497704590818 | -0.0409336526946108 |
| 5934 | 0.0427661538461539 | 0.157237205588822 | 0.0775627744510978 | 0.0243652694610778 |
| 5935 | 0.0523624615384615 | 0.0849535728542914 | 0.828419161676647 | 0.116141117764471 |
| 5936 | 0.0554916923076923 | -0.0441823552894212 | -0.346799001996008 | 0.0297256287425150 |
| 5937 | 0.0573692307692308 | -0.0477559281437126 | -1.00466127744511 | -0.0168932534930140 |
| 5938 | 0.0250338461538462 | -0.0614004790419162 | 0.938062874251497 | 0.0623750898203593 |
| 5939 | -0.0279544615384615 | -0.237155289421158 | 0.411366966067864 | 0.00714714570858283 |

| | | | | |
|---|---|---|---|---|
| 5940 | -0.0559089230769231 | 0.0311875449101796 | -0.704562375249501 | -0.131247584830339 |
| 5941 | -0.0458953846153846 | 0.141643433133733 | -0.454006187624751 | -0.0558776846307385 |
| 5942 | -0.0271200000000000 | -0.0303753692614770 | 0.645679640718563 | 0.0480807984031936 |
| 5943 | -0.0118910769230769 | 0.0859281836327345 | 0.0552279441117765 | -0.0290758882235529 |
| 5944 | -0.0177323076923077 | 0.191186147704591 | -0.737455489021956 | -0.124425309381238 |
| 5945 | -0.0400541538461538 | -0.0188424750499002 | 0.279388423153693 | -0.0656237924151697 |
| 5946 | -0.0233649230769231 | -0.101034650698603 | -0.151064670658683 | 0.125399920159681 |
| 5947 | 0.00354646153846154 | -0.0420706986027944 | -0.189643013972056 | 0.0885271457085828 |
| 5948 | 0.0227390769230769 | -0.102983872255489 | 0.267205788423154 | -0.195571896207585 |
| 5949 | 0.0116824615384615 | -0.000162435129740519 | 0.279388423153693 | -0.0846287025948104 |
| 5950 | -0.0269113846153846 | 0.0588015169660679 | -0.395123453093812 | 0.200444950099800 |
| 5951 | -0.0477729230769231 | 0.0128323752495010 | -0.0816236526946108 | 0.000324870259481038 |
| 5952 | -0.0423489230769231 | 0.0472686227544910 | 0.821515668662675 | -0.235530938123752 |
| 5953 | -0.0104307692307692 | 0.187612574850299 | -0.0540096806387226 | -0.00487305389221557 |
| 5954 | 0.0248252307692308 | 0.130435409181637 | -0.653801397205589 | 0.188424750499002 |
| 5955 | 0.0233649230769231 | -0.155937724550898 | -0.0393905189620759 | -0.0575020359281437 |
| 5956 | -0.0137686153846154 | -0.114679201596806 | 0.704968463073852 | -0.218962554890220 |
| 5957 | -0.0410972307692308 | 0.368727744510978 | -0.141724650698603 | 0.00617253493013972 |
| 5958 | -0.0223218461538462 | 0.307977005988024 | -0.758572055888224 | 0.0843038323353293 |
| 5959 | 0.0256596923076923 | -0.656237924151697 | 0.0954306387225549 | -0.179165948103792 |
| 5960 | 0.0546572307692308 | -0.650390259481038 | 0.458473153692615 | -0.136932814371257 |
| 5961 | 0.0554916923076923 | 0.181602475049900 | 0.0154313373253493 | 0.160648343313373 |
| 5962 | 0.0300406153846154 | 0.209054011976048 | -0.399996506986028 | 0.0516543712574850 |
| 5963 | 0.0112652307692308 | 0.0795932135728543 | 0.138475948103792 | -0.153338762475050 |
| 5964 | 0.0183581538461538 | 0.110455888223553 | 0.351672055888224 | 0.0339489421157685 |
| 5965 | 0.0216960000000000 | -0.244952175648703 | -0.0994915169660679 | 0.223998043912176 |
| 5966 | 0.0256596923076923 | 0.251936886227545 | -0.130354191616766 | 0.0427204391217565 |
| 5967 | 0.0175236923076923 | 1.04445788423154 | 0.0824358283433134 | -0.0916134131736527 |
| 5968 | 0.0156461538461538 | -0.253723672654691 | 0.0856845309381238 | 0.101521956087824 |
| 5969 | 0.0285803076923077 | -1.03389960079840 | -0.0954306387225549 | 0.230657884231537 |
| 5970 | 0.0494418461538462 | 0.492990618762475 | 0.119795908183633 | 0.106395009980040 |
| 5971 | 0.0755187692307692 | 0.946509500998004 | 0.0714714570858283 | 0.0144567265469062 |
| 5972 | 0.0690516923076923 | -0.462777684630739 | -0.124262874251497 | 0.0303753692614770 |
| 5973 | 0.0527796923076923 | -0.582005069860279 | 0.110455888223553 | -0.0250150099800399 |
| 5974 | 0.0392196923076923 | 0.700257844311377 | 0.275733632734531 | -0.0115328942115768 |
| 5975 | 0.0563261538461538 | 0.539284630738523 | -0.0661923153692615 | 0.0808926946107784 |
| 5976 | 0.0821944615384615 | -0.258921596806387 | -0.295631936127745 | -0.00162435129740519 |
| 5977 | 0.0817772307692308 | -0.117278163672655 | 0.0917758483033932 | -0.0675730139720559 |
| 5978 | 0.0630018461538462 | -0.177866467065868 | 0.0284261477045908 | 0.0596136926147705 |
| 5979 | 0.0321267692307692 | -0.590451696606786 | -0.250962275449102 | 0.0459691417165669 |
| 5980 | 0.0354646153846154 | -0.143755089820359 | -0.102334131736527 | -0.127674011976048 |
| 5981 | 0.0659224615384615 | 0.174617764471058 | 0.270048403193613 | -0.0198170858283433 |
| 5982 | 0.112860923076923 | -0.337865069860279 | 0.0869027944111777 | 0.0946996806387226 |
| 5983 | 0.121205538461538 | -0.0573396007984032 | -0.132790718562874 | -0.0826794810379242 |

| | | | | |
|---|---|---|---|---|
| 5984 | 0.0930424615384616 | 0.479183632734531 | 0.130354191616766 | -0.133196806387226 |
| 5985 | 0.0617501538461538 | 0.0955118562874252 | 0.263957085828343 | 0.0183551696606786 |
| 5986 | 0.0340043076923077 | -0.294657325349301 | 0.0495427145708583 | -0.00617253493013972 |
| 5987 | 0.0529883076923077 | 0.113704590818363 | -0.272484930139721 | -0.148465708582834 |
| 5988 | 0.0715550769230769 | 0.334616367265469 | 0.0844662674650699 | -0.0110455888223553 |
| 5989 | 0.0604984615384615 | -0.239591816367265 | 0.154313373253493 | 0.146191616766467 |
| 5990 | 0.0423489230769231 | -0.259733772455090 | -0.0828419161676647 | 0.0116953293413174 |
| 5991 | 0.0308750769230769 | 0.309601357285429 | -0.162435129740519 | -0.165683832335329 |
| 5992 | 0.0406800000000000 | 0.300342554890220 | -0.0162435129740519 | -0.0237155289421158 |
| 5993 | 0.0700947692307692 | -0.0261520558882236 | 0.233094411177645 | 0.148140838323353 |
| 5994 | 0.0805255384615385 | 0.0602634331337325 | 0.0434513972055888 | 0.0464564471057884 |
| 5995 | 0.0669655384615385 | 0.148303273453094 | -0.0422331337325349 | 0.0575020359281437 |
| 5996 | 0.0454781538461538 | -0.0105582834331337 | -0.0982732534930140 | 0.0651364870259481 |
| 5997 | 0.0277458461538462 | -0.191998323353293 | 0.104364570858283 | -0.0622126546906188 |
| 5998 | 0.0346301538461538 | -0.416321237524950 | 0.174211676646707 | -0.0295631936127745 |
| 5999 | 0.0554916923076923 | -0.231794930139721 | -0.179896906187625 | 0.101359520958084 |
| 6000 | 0.0742670769230769 | 0.180790299401198 | -0.279794510978044 | 0.101359520958084 |
